# Predicting Solvation Free Energies Using Electronegativity-Equalization Atomic Charges and a Dense Neural Network: A Generalized-Born Approach

**DOI:** 10.1021/acs.jctc.3c00858

**Published:** 2023-11-14

**Authors:** Sergei F. Vyboishchikov

**Affiliations:** Institut de Química Computacional i Catàlisi and Departament de Química, Universitat de Girona, Carrer Maria Aurèlia Capmany 69, 17003 Girona, Spain

## Abstract

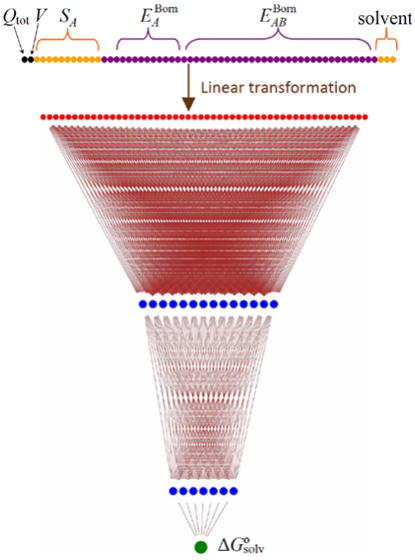

I propose a dense Neural Network, ESE-GB-DNN, for evaluation of solvation free energies Δ*G*°_solv_ for molecules and ions in water and nonaqueous solvents. As input features, it employs generalized-Born monatomic and diatomic terms, as well as atomic surface areas and the molecular volume. The electrostatics calculation is based on a specially modified version of electronegativity-equalization atomic charges. ESE-GB-DNN evaluates Δ*G*°_solv_ in a simple and highly efficient way, yet it offers a high accuracy, often challenging that of standard DFT-based methods. For neutral solutes, ESE-GB-DNN yields an RMSE between 0.7 and 1.3 kcal/mol, depending on the solvent class. ESE-GB-DNN performs particularly well for nonaqueous solutions of ions, with an RMSE of about 0.7 kcal/mol. For ions in water, the RMSE is larger (2.9 kcal/mol).

## Introduction

Evaluation of solvation free energy Δ*G*°_solv_ is an important quest in computational chemistry, since it makes a sizable contribution to the total Gibbs energy for chemical reactions in solutions, especially when ions are involved. Most practical calculations of Δ*G*°_solv_ for processes in solutions utilize Continuum Solvation models, which can be subdivided into the Polarizable Continuum Model (PCM)^1−14^ and the Generalized Born (GB)^15−21^ methods. In both approaches, Δ*G*°_solv_ is typically partitioned into the electrostatic energy *E*_elst_ and the nonelectrostatic correction term Δ*G*°_corr_:
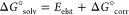
1

In the PCM-type methods, the solvent polarization is represented by a charge distribution on the surface of the cavity surrounding the solute molecule. On the other hand, the GB-type methods do not require an explicit construction of the molecular cavity, which makes them more computationally efficient. *E*_elst_ is then expressed directly through solute atomic charges {*Q*_*I*_} and effective Born radii {*R*_*I*_} as follows
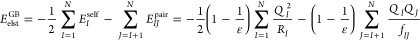
2where *N* is the number of atoms; *ε* is the dielectric constant of the solvent; and *f*_*IJ*_ is a function of atomic radii and interatomic distance *r*_*IJ*_. The monatomic terms (self-terms) *E*_*I*_^self^ = (1–1/ε)*Q*_*I*_^2^/*R*_*I*_ in eq 2 are identical to the expression of Born’s solvation theory^22^ for spherical ions. The choice of an analytical form of the *f*_*IJ*_ function in the pair term *E*_*IJ*_^pair^ = (1–1/ε)*Q*_*I*_*Q*_*J*_/*f*_*IJ*_ and of effective Born radii *R*_*I*_ is crucial to achieve an acceptable accuracy of the GB method.^23^ An often accepted form of *f*_*IJ*_ is
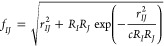
3where *c* can be set to 4 (as in the original work by Still et al.^15^) or to another value.^21^ Expressions alternative to eq 3 for *f*_*IJ*_ were also proposed.^17,24^ The GB approach was implemented in a wide number of solvation energy schemes,^16−19^ often in conjunction with a nonelectrostatic term (Δ*G*°_corr_ in eq 1). The simplest form of Δ*G*°_corr_ = ∑_*I*_κ_*I*_*S*_*I*_ term^15^ effectively describes the cavitation and dispersion energies through atomic surface areas {*S*_*I*_}; the element-dependent coefficients {κ_*I*_} are occasionally referred to as atomic surface tension.^2,12^ However, {κ_*I*_} are in fact treated as adjustable (semiempirical) parameters. More elaborated computational schemes involve charge- or atomic-position dependent {κ_*I*_} formulations.^25^

Taking a partly empirical (adjustable) character of parameters *R*_*I*_ and κ_*I*_ into account, one can conceive the GB approach as a linear or nonlinear regression problem in the space of terms {*E*_*I*_^self^}, {*E*_*IJ*_^pair^}, and {*S*_*I*_}. If a sufficiently large database is available, it is attractive to formulate a flexible Δ*G*°_solv_({*E*_*I*_^self^},{*E*_*IJ*_^pair^},{*S*_*I*_}) dependence without fixing a particular analytical form. This can be achieved by means of an artificial neural network (ANN).^26^ In this paper, I introduce a dense ANN that utilizes {*E*_*I*_^self^}, {*E*_*IJ*_^pair^}, {*S*_*I*_}, plus the molecular volume *V* and some extra parameters (*vide infra*) as input features to calculate Δ*G*°_solv_. The atomic charges {*Q*_*I*_} will be obtained by an appropriately modified Electronegativity-Equalization (EE) charge scheme, which is highly efficient computationally.

In recent years, several neural-network based solvation-energy schemes for Δ*G*°_solv_ evaluation have been developed. A generalized-Born approach for a graph ANN with atomistic embedding was employed by Chen et al.^27^ Vermeire and Green^28^ used textual molecular identifiers (SMILES and InChI) as input features for a directed message passing ANN. Lim and Jung^29^ proposed a graph convolutional ANN and a recurrent ANN based on atomic vectors. Alibakhshi and Hartke^30^ built a quite accurate ANN utilizing a self-consistent C-PCM calculated input. Other works in this field include Bernazzani et al.,^31^ Borhani et al.,^32^ Hutchinson and Kobayashi,^33^ Wang et al.,^34^ and Jaquis et al.^35^

In our previous works,^36−41^ we developed an efficient and accurate noniterative method for calculating Δ*G*°_solv_, named uESE (*universal Easy Solvation Energy*). It employs the COSMO^42,43^ electrostatics plus a number of additive correction terms that depend on {*S*_*I*_}, *V*, and atomic surface charges. Atomic charges needed as input for the COSMO calculation can be evaluated by various techniques^36,44−47^ including semiempirical^39^ methods.^47^ Importantly, EE charges are also suitable, resulting in the ESE-EE method.^40^ Nevertheless, a higher computational efficiency of the semiempirical versions of ESE comes at the cost of accuracy, with the DFT-based uESE performing noticeably better that ESE-EE, especially for ionic solutes.

The present work differs from the previous ones of the ESE family first in that an ANN rather than a linear function is employed, and second that the COSMO electrostatic energy term is replaced by GB-type terms {*E*_*I*_^self^} and {*E*_*IJ*_^pair^} (not by the total *E*^GB^_elst_ of eq 2). The expected advantage of this approach is that no explicit cavity surface has to be constructed, nor surface charges to be calculated. On the other hand, an appropriately trained ANN should provide sufficient flexibility to obtain accurate Δ*G*°_solv_. Therefore, we strive for a rapid yet accurate ANN-based solvation energy scheme. The idea of using the EE charges is encouraged by the simplicity and an extraordinary efficiency of the EE charge scheme and by a reasonable performance of the ESE-EE method for neutral solutes.^40^ An EE charge calculation does not require any quantum-mechanical input, just the molecular geometry. Thus, such a DNN will use physically sound GB-based input features. Therefore, Δ*G*°_solv_ will be geometry-dependent, such that the method can treat different molecular configurations. The details of our version of the EE method and of the GB term calculations, as well as the ANN construction and training, will be provided in the Methods section below.

## Methods

### Electronegativity Equalization

As explained in the Introduction, the atomic charges {*Q*_*I*_} for the GB-type calculation are evaluated by a specially modified version of the EE method. It is similar but not identical to that used within the ESE-EE method^40^ and to those by Svobodová Vařeková et al.,^48^ Ouyang et al.,^49^ and Menegon et al.^52^ The computation of EE charges can be conveniently expressed in matrix form as follows^50^
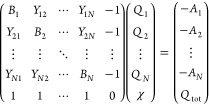
4where *A*_*I*_ and *B*_*I*_ are element-dependent parameters characterizing the intrinsic electronegativity and hardness of the *I*-th atom, correspondingly; *Q*_tot_ is the total charge of the molecule; *N* is the number of atoms; {*Q*_*I*_} are the resulting atomic charges obtained as the solution to the system by equations 4; and χ is the resulting equalized electronegativity. Various forms of the geometry-dependent off-diagonal matrix elements *Y*_*IJ*_ were proposed.^48,51−54^ In the present version, {*A*_*I*_} and {*B*_*I*_} are adjustable parameters depending not only on the element but also on the coordination number of the atom. The off-diagonal terms *Y*_*IJ*_ contain two more parameters κ and κ_2_:
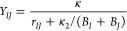
5

The least-squares fitting of the EE parameters {*A*_*I*_}, {*B*_*I*_}, κ, and κ_2_ was done using the downhill simplex algorithm^55^ available in the Python SciPy package,^56^ which does not require analytical derivatives. As the target values, CM5^46^ atomic charges of all atoms for 528 molecules from the Minnesota Solvation Database (MNSol)^57^ were used. The procedure remotely resembles that of Menegon et al.^52^ A root-mean-square error (RMSE) of about 0.03 electrons was reached. The resulting values of the EE parameters are provided in Table 1.

**Table 1:** Element- and Coordination-Number Dependent EE Parameters Optimized by Nonlinear Least-Squares Fitting^a^

Element	Coordination number	*A*_*i*_	*B*_*i*_	Element	Coordination number	*A*_*i*_	*B*_*i*_
H	1	2.364	0.961	P	1–3	2.448	0.705
	2	2.304	1.458		4	2.444	0.436
C	1	2.452	0.658		5	2.624	0.465
	2	2.452	0.658	S	1	2.501	0.562
	3	2.435	0.658		2	2.461	0.961
	4	2.422	0.672		3	2.382	0.556
N	1	2.628	0.872		4	2.304	0.789
	2	2.534	0.679		5	2.226	0.675
	3	2.502	0.634		6	2.941	0.601
	4	2.824	1.551	Cl		2.446	0.644
O	1	2.545	0.720	Br		2.420	0.412
	2, 3	2.502	0.675	I		2.426	0.746
F		2.577	1.478				
Si	3	2.3	0.600				
	4	2.3	0.605				

a κ = 0.991 Å; κ_2_ = 1.371 Å.

### Neural Network: Input Features and Hidden Layers

The ANN presented in this work – ESE-GB-DNN (*Easy Solvation Energy – Generalized Born – Dense Neural Network*) – is a dense ANN with two hidden layers. After a number of tests, the first hidden layer with 16 neurons and the second one with 8 neurons were chosen for aqueous solutions. For nonaqueous solutions, 14 × 7 neurons were used. In each case, biases and rectified linear unit (*ReLU*) activation functions were employed for the hidden layers. The output layer (also with a bias) has a linear activation function.

Initially, the following input features were included:

(1) the number of atoms in the solute molecule;

(2) the total charge of the solute *Q*_tot_;

(3) the molecular volume *V*_*tot*_ of the solute, which is the sum of atomic volumes, *V*_*tot*_ = ∑_*I*_*V*_*I*_ (*vide infra*);

(4) the total surface area *S*_*tot*_ of the solute (the sum of atomic surfaces, *S*_*tot*_ = ∑_*I*_*S*_*I*_, *vide infra*);

(5–13) Atomic surface areas *S*_*L*_ = ∑_*I*∈*L*_*S*_*I*_ for nine elements *L* = H, C, N, O, F, S, Cl, Br, I;

(14–22) the ε-dependent Born-type self-terms:
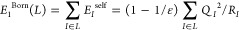
6for the same nine elements *L* calculated from EE charges;

(23–58) the ε-dependent Born-type pair terms:
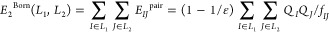
7Of 9·(9+1)/2 = 45 possible *E*_2_^Born^ terms, only 36 were used in fact. This is because some of the *L*_1_*–L*_2_ element combinations are scarcely represented in the training data set. The full list is given in the Supporting Information. I employed the form of *f*_*IJ*_ according to eq 3, with *c* = 4 and unmodified Bondi^58^ radii *R*_*I*_.

For nonaqueous solutions, I added three more features in order to describe the properties of the solvent:

(59) the dielectric constant ε of the solvent;

(60) the boiling point (BP) of the solvent;

(61) the number of non-hydrogen atoms in the solvent, which characterizes the solvent molecular size.

The features 59–61 indirectly represent solvent properties, albeit incompletely. They will allow ESE-GB-DNN to learn the difference between conventional solvent classes such as polar protic, polar aprotic, and nonpolar. This three-parameter solvent description is a much simpler one than that of Borhani et al.,^32^ who made use of as many as 12 solvent features.

### Surface and Volume Calculations

The input features 3–13 (*vide supra*) are geometric characteristics of the solute that must be evaluated from its molecular geometry and van der Waals radii. I adopted the following formulas for the atomic surface area *S*_*I*_ of the *I*-th atom
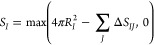
8where
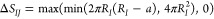
9
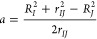
10Analogously,
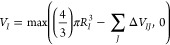
11where
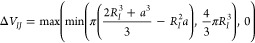
12

**Figure 1:**
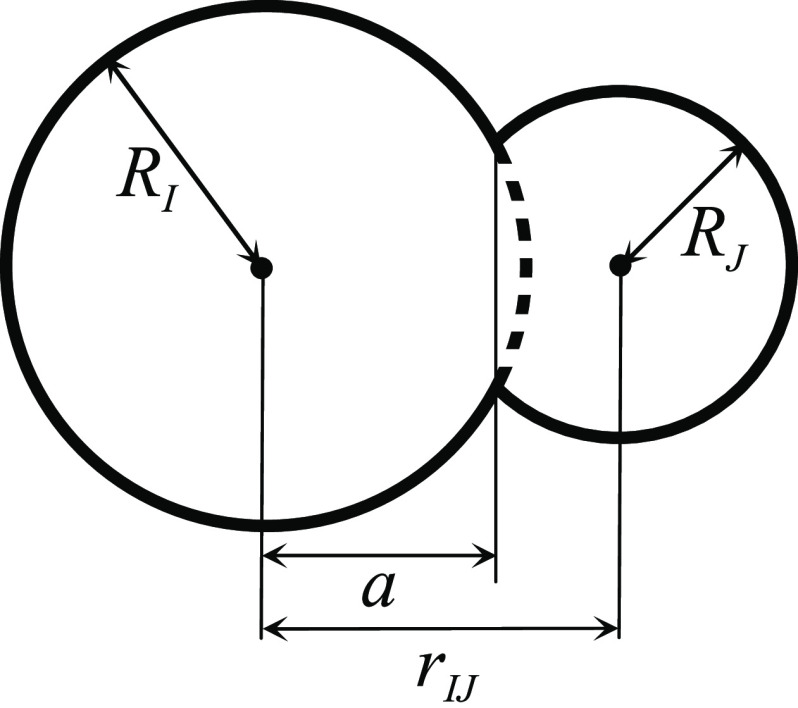
Estimation of the atomic surface area and volume (eqs 8–11). The dashed line indicates the surface of the spherical cap of atom *I* (Δ*S*_*IJ*_, eq 9), buried inside atom *J*. Parameter *a* (eq 10), which can be positive or negative, shows the position of the crossing plane between the spheres of atoms *I* and *J*.

The summation in eqs 8 and 11 runs over all the atoms *J* adjacent to the given atom *I*. Eqs 8 and 11 do not give the exact van der Waals surface area for complicated cases, when there is multiple atomic-sphere overlap. Nevertheless, they provide a good estimate suitable for the use as a DNN input. The derivation of eqs 8 and 11 based on elementary geometry is briefly illustrated in Figure 1. Δ*S*_*IJ*_ and Δ*V*_*IJ*_ is the area and the volume of the spherical cap of atom *I* buried inside the atom *J*, respectively, which is shown by a dashed line in Figure 1. Although Mongan et al.^59^ developed a more sophisticated version of the molecular volume calculation, in the context of the ANN, it is appealing to take advantage of the simplicity of eqs 8–11.

### Dimensionality Reduction

Calculation of the correlation matrix of the 58 input features revealed a substantial (in some cases, a very strong) correlation between some of them. In the MNSol database, 9 features turned out to have a correlation coefficient greater than 0.91. To decrease the number of the ANN parameters to be fitted and achieve a more stable behavior of ESE-GB-DNN, I used the principal component analysis to truncate the nine most correlated features. This was done by means of singular value decomposition as implemented in the sklearn.decomposition.PCA class. This procedure resulted in a 58 × 49 (or 61 × 52 for nonaqueous solutions) transformation matrix that produced a vector of the 49 (or 52) actual input features for the DNN.

### ANN Training

Two dense ANNs were independently trained: one (with 49 transformed input features) for aqueous solutions and the other (with 52 transformed input features) for nonaqueous ones. The resulting ESE-GB-DNN have, therefore, a total of (49+1)·16 + (16+1)·8 + (8+1) = 945 and (52+1)·14 + (14+1)·7 + (7+1) = 855 parameters to be trained for aqueous and nonaqueous solutions, correspondingly. The transformed input data are scaled and fed into the dense ANN described above. The fitting of ESE-GB-DNN was done using the Nesterov-accelerated^60^ Adaptive Moment Estimation algorithm^61^ as implemented in the tensorflow.keras.optimizers.Nadam class,^62^ with a suitably weighted mean squared error as the loss function. To avoid overparametrization, _2_ regularization with a strength λ = 0.01 was applied.

The EE charge calculation, data preprocessing, and the dense ANN training were implemented in a Python 3.7 code. Subsequently, the optimized ANN parameters (neuron weights and biases as well as the feature transformation matrix) were incorporated into a user-friendly Fortran code that reads a molecular geometry, evaluates the EE charges, calculates the *E*_1_^Born^(*L*) and *E*_2_^Born^(*L*_1_,*L*_2_) components, molecular volume, and atomic surfaces, and finally evaluates Δ*G*°_solv_ through ESE-GB-DNN.

Our training sets are partly based on the CombiSolv-QM,^28^ – a solvation free energy database calculated for neutral solutes by means of the COSMO-RS theory.^43^ For our study, the data at 298 K were chosen. In addition, the training set was expanded by a random half of the experimental Δ*G*°_solv_ values from MNSol,^57^ since MNSol includes both neutral and ionic solutes. Thus, in total there were 4242 data for aqueous solutions. No data for ions are available in the CombiSolv-QM database, whereas the collection of ionic data in MNSol is also limited. Therefore, in order to increase the stability of the resulting ANN for ionic solutes, the training database was extended by 10000 extrapolated ionic data that encompass a wide range of dielectric constants (10 < ε < 200) and boiling points (40 °C < BP < 210 °C). Specifically, from the existing data for ionic solutes in dimethyl sulfoxide, acetonitrile, and methanol, extrapolated Δ^ref^*G*°_solv_(ε_new_) target values corresponding to a dielectric constant ε_new_ (10 < ε_new_ < 200) were created according to the following formula
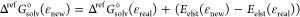
13where ε_real_ is the actual dielectric constant of the solvent. Eq 13 is based on the assumption that the nonelectrostatic component of the solvation energy (Δ*G*°_corr_ in eq 1) is independent of ε, like in our earlier ESE models.^36−41^ Additionally, the data were replicated to encompass a wide range of boiling points. In total, the training data set for the nonaqueous solutions contained 14640 entries that originate from MNSol, extrapolated MNSol, and CombiSolv-QM. For the sake of comparison, I also did a second training for nonaqueous solutions, in which CombiSolv-QM data were excluded (11716 data in total). For all the trainings, a validation split of 0.2 was applied, thus assigning 20% of the training data for validation. The learning rate was typically set to 0.001 or 0.0001.

## Results and Discussion

Since the CombiSolv-QM database provides SMILES codes rather than molecular geometries for the solutes, the geometries were created from the SMILES by the OpenBabel free online converter.^63^ For the MNSol database, PM7-optimized^47^ geometries were employed from my previous paper.^39^ From the databases used for testing (*vide infra*), the Cartesian coordinates were used as they are. With these geometries, atomic surfaces and volumes, EE atomic charges, and subsequently the *E*_1_^Born^(*L*_1_) (eq 6) and *E*_2_^Born^ (*L*_1_,*L*_2_) (eq 7) terms were calculated to generate the input necessary to train ESE-GB-DNN (see Methods section above, features 14–58). The general results of the training described in the Methods section above are summarized in Table 2. Subsequently, ESE-GB-DNN was tested on a number of data sets that include both neutral and ionic solutes. We checked it against other implicit-solvation methods, paying particular attention to SMD,^13^ which, cited more than 13 thousand times, can be regarded as a standard for routine Δ*G*°_solv_ evaluations in practical computational chemistry. The data sets used for the independent tests are as follows: the 141-solute reduced data set by Mobley et al.;^64^ Guthrie’s “blind challenge” data set with 63 pharmacologically relevant molecules;^65^ Guthrie’s 53-molecule reduced data set (SAMPL1);^65^ reduced Guthrie’s SAMPL4 data set^66^ (SAMPL4); and ionic C10 data set (6 cations and 4 anions).^67^

**Table 2:** Mean Signed Error (MSE), Mean Absolute Error (MAE), Root-Mean-Square Error (RMSE), Slope, Intercept, and Coefficient of Determination *R*^2^ for the Data Sets Used for Training and Validation of ESE-GB-DNN for Aqueous and Nonaqueous Solutions (in kcal/mol)

Training (number of solutes)	MSE	MAE	RMSE	Slope	Intercept	*R*^2^
Aqueous training (3394)	–0.01	0.91	1.41	0.981	–0.13	0.983
Validation (848)	0.04	0.98	1.56	0.965	–0.18	0.977
All (4242)	0.00	0.93	1.44	0.978	–0.14	0.982
Nonaqueous training I (11711)^a^	0.21	0.63	0.89	0.992	–0.17	0.999
Validation (2928)	0.25	0.67	0.97	0.992	–0.16	0.999
All (14639)	0.21	0.64	0.91	0.992	–0.17	0.999
Nonaqueous training II (9373)^b^	0.09	0.51	0.69	1.001	0.13	0.999
Validation (2343)	0.09	0.54	0.80	1.002	0.18	0.999
All (11716)	0.09	0.51	0.72	1.001	0.14	0.999

a Training including the CombiSolv-QM data.

b Training excluding the CombiSolv-QM data.

### Aqueous Solutions

Table 3 gives the RMSE for ESE-GB-DNN (split into the training and testing subsets) as well as for a number of other solvation methods. Compared to other semiempirical methods (our ESE-PM7^39^ and ESE-EE,^40^ as well as PM7/COSMO2,^67^ and the semiempirical versions^68^ of SMD), our ANN-based ESE-GB-DNN model is clearly superior for all examined databases, with the exception of the ionic C10 set, as for the latter, ESE-PM7 and PM7/COSMO2 are a little better.

**Table 3:** RMSE of the Hydration Free Energy in kcal/mol for Various Data Sets by the ESE-GB-DNN Method in Comparison with Other DFT-Based and Semiempirical Methods^a^

	ESE-GB-DNN											
Solute database (number of solutes in total/training/testing data sets)	total	training	testing	uESE/B3LYP/Def2TZVP	SMD/B3LYP/Def2TZVP	ESE-EE	ESE-PM7	ESE-PM7(SN)^b^	PM7/COSMO2	SMD/PM3^c^	SMD/PM6^c^	SMD/DFTB^c^
MNSol (528/207/321)^d^	1.84	1.72	1.92	2.24	4.19	3.34	2.79	2.62		5.0	7.5	4.1
Neutrals (389/141/248)	1.30	1.27	1.32	1.48	1.70	2.04	2.21	1.96		2.4	4.0	3.1
Cations (60/25/35)	2.59	1.68	3.09	3.43	5.08	5.09	3.91	4.20		9.2	10.3	4.9
Anions (82)	3.04	2.80	3.29	3.66	8.96	5.41	4.03	3.72		7.9	14.2	6.7
MNSol* (464/187/277)^d^^,^^e^	1.67	1.75	1.61	2.16	4.23	3.31	2.64	2.53	2.62^g^			
Neutrals (330/122/208)	1.09	1.24	0.98	1.25	1.38	2.29	1.90	1.72	2.24^g^			
Cations (59/25/34)	2.59	1.68	3.09	3.43	5.11	5.09	3.91	4.20	2.87^g^			
Anions (75/40/35)	2.60	2.80	2.35	3.56	9.05	5.38	3.91	3.56	3.69^g^			
Mobley 141 (141)^f^			1.30	3.38	3.02	2.22	1.72	1.65	2.54^h^			
Blind (63)^f^			2.15	2.95	3.54	3.42	3.49	2.94				
SAMPL 1(53)^f^			1.70	1.85	2.59	2.96	3.50	2.91	3.73^g^			
SAMPL 4(42)^f^			1.50	1.67	1.23	2.42	1.60	1.59	1.92^g^			
C10 (10)^f^			2.59	3.49	5.45	6.87	2.22	2.31	2.28^g^			

a The complete lists of solutes and the calculated hydration free energies and the reference values, as well as MSE and MAE, are given in the Supporting Information.

b ESE-PM7 with improved parameters for sulfur and nitrogen; see ref (39) for details.

c Data from ref (68) (Table 3).

d Training/testing set; for an explanation see text.

e MNSol* is Kříž and Řezáč’s data set of 464 solutes.^67^

f Testing set, hence no splitting into training/testing is shown.

g Data from ref (67).

h Data from ref (67). Mobley266 data set.

The present ESE-GB-DNN also definitely outperforms the DFT-based SMD^13^ and uESE^38^ methods for virtually all testing sets, even when considering only the testing-set data for MNSol. It is only for the SAMPL4 set that SMD yields a somewhat lower RMSE. Nevertheless, even for SAMPL4, the RMSE of 1.5 kcal/mol and an MAE about 1.1 kcal/mol produced by ESE-GB-DNN is an acceptable accuracy in many practical situations.

The results obtained by ESE-GB-DNN as well as by the DFT-based uESE and SMD methods for various chemical classes of neutral solutes are compiled in Table 4. For 7 of 13 of these classes, ESE-GB-DNN surpasses both the uESE and SMD methods: for alcohols, ethers, nitriles, nitro compounds/nitrates, and halogen-containing solutes (except for bromine-containing ones). For esters and amines, ESE-GB-DNN also yields good results. Only for small molecules the performance of ESE-GB-DNN is somewhat lower.

**Table 4:** RMSE of the Hydration Free Energy Calculated by the ESE-GB-DNN Method in Comparison with DFT-Based SMD and uESE Methods for Various Classes of the MNSol Database (in kcal/mol)^a^

	ESE-GB-DNN				
Solute class (number of solutes in total/training/testing data sets)	total	training	testing	uESE^b^	SMD^c^
Small molecules (24/6/18)^d^	1.23	1.31	1.20	0.68	0.63
Alcohols (18/8/10)	0.42	0.50	0.35	0.73	0.87
Aldehydes and ketones (22/8/14)	0.73	0.95	0.56	0.73	0.77
Ethers (10/8/2)	0.56	0.57	0.51	1.15	1.07
Esters (20/5/15)	0.72	0.91	0.64	0.64	0.87
Acids (10/5/5)	1.43	0.95	1.78	0.75	2.01
Amines (42/13/29)	1.12	1.50	0.90	1.48	0.95
Nitriles (4/1/3)	0.29	0.24	0.31	0.37	0.37
Nitro compounds and nitrates (17/5/12)	0.68	0.53	0.73	1.39	1.99
Fluorine compounds (33/12/21)	0.93	1.17	0.76	1.46	1.49
Chlorine compounds (74/25/49)	1.14	0.94	1.23	1.64	2.23
Bromine compounds (25/6/19)	1.94	3.37	1.18	1.30	1.60
Iodine (10/5/5)	0.53	0.49	0.58	1.40	1.35
Linear correlation^e^ (for all 389/141/248 neutral solutes):					
Slope	0.892	0.899	0.888	0.928	0.924
Intercept	–0.37	–0.37	–0.38	–0.46	0.23
*R*^2^	0.914	0.921	0.911	0.890	0.873

a The complete lists of molecules in all the subsets, as well as the mean signed errors (MSEs) and mean absolute errors (MAEs), are given in the Supporting Information.

b Data from ref (38). The total set.

c Data from ref (37). The total set.

d Molecules containing less than six atoms.

e Linear correlation between Δ*G*°_solv_ obtained within a given method and the reference Δ*G*°_solv_ value.

**Figure 2:**
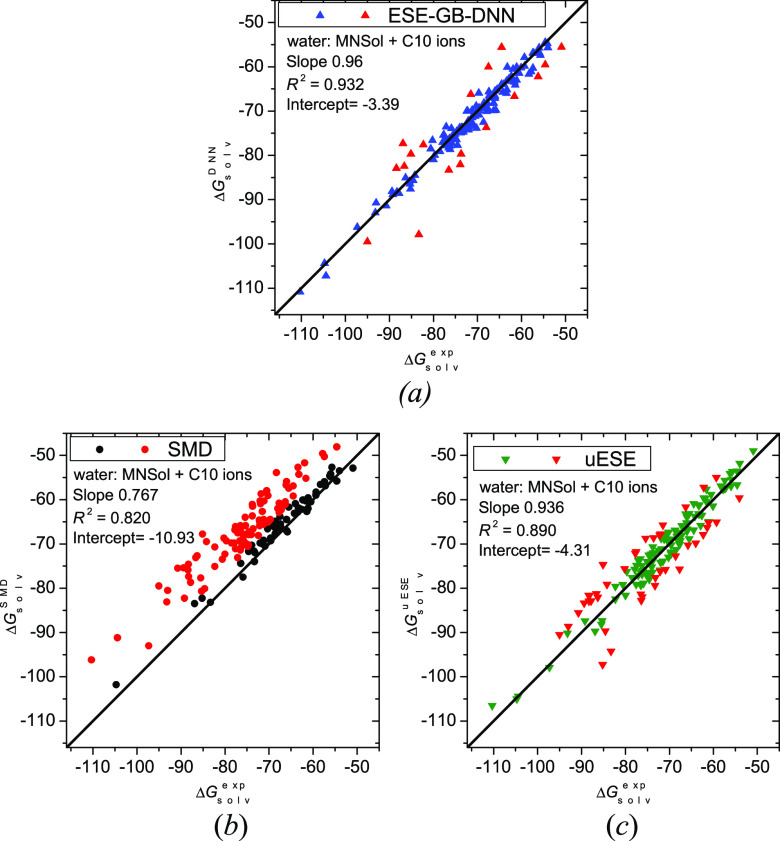
Hydration free energies (in kcal/mol) for ions from the MNSol and C10 data sets calculated by ESE-GB-DNN (*a*), SMD (*b*), and uESE (*c*) methods versus reference values. Red points denote outliers with a deviation greater than 4 kcal/mol. The sloping straight line is the identity line.

The performance of ESE-GB-DNN for ionic solutes is demonstrated in Figure 2, in which hydration energies for all the ions from MNSol plus C10 data sets are given. Problematic cases (|Δ^calc^*G*°_solv_ – Δ^ref^*G*°_solv_| > 4 kcal/mol) are indicated in red. For ESE-GB-DNN (Figure 2a), there are 18 such outliers out of 152 ions. The two worst cases (with a deviation > 9 kcal/mol) are “c089” (OH^–^·H_2_O) and “i091” (O_2_^–^). The former failure can be explained by unphysical EE charge redistribution between the OH^–^ and H_2_O fragments, rendering the water moiety not fully neutral. For the uESE method (Figure 2c), there are 42 deviations beyond 4 kcal/mol. The SMD method (Figure 2b) fails much more often (92 failures, i.e. the majority), with a substantially lower coefficient of determination *R*(2) and a clear trend of underestimating |Δ*G*°_solv_|.

### Nonaqueous Solutions

Two distinct trainings were done for nonaqueous solutions: one combining half of the MNSol database with the CombiSolv-QM database (nonaqueous training I in Table 2) and the other with the MNSol database only (nonaqueous training II). The details of the data sets used are given in the Methods section. Both nonaqueous trainings yield comparable quality (see Table 2). However, testing on the entire MNSol database produced more convincing results for the mixed database (nonaqueous training I). All the data and discussion in this section refer to nonaqueous training I. The results of alternative training II are given in Supporting Information.

In the discussion below, the solvents are subdivided into three standard classes: *polar protic* solvents (Table 5); *polar aprotic* solvents (Table 6); and *nonpolar* solvents. The latter class includes all those with ε < 9 regardless of their chemical nature, Table 7.

**Table 5:** RMSE of the Solvation Free Energy in kcal/mol for 14 *Polar Protic* Solvents Computed Using the ESE-GB-DNN Model in Comparison with DFT-Based uESE and SMD as well as with Semiempirical ESE-PM7 and ESE-EE^c^

Solvent^a^	ESE-GB-DNN	uESE	SMD	ESE-PM7	ESE-EE
Octanol (247)	1.10	1.13	1.72	1.40	1.61
Heptanol (12)	0.95	0.52	1.03	0.95	0.88
*m*-Cresol (7)	1.19	0.87	1.75	1.33	1.37
Benzyl alcohol (10)	0.65	0.38	0.87	1.00	0.79
Hexanol (14)	0.94	0.47	1.04	0.93	0.78
Pentanol (22)	1.07	0.82	0.90	1.17	1.11
*sec*-Butanol (9)	0.71	0.46	0.72	0.55	0.58
Isobutanol (17)	1.25	0.83	0.68	1.00	0.72
Methoxyethanol (6)	0.57	0.49	0.94	1.21	0.75
Butanol (21)	1.12	0.87	0.89	1.33	1.40
Iospropanol (7)	0.79	0.73	1.22	1.53	1.17
Propanol (7)	0.76	0.67	1.02	1.50	1.15
Ethanol (8)	1.08	1.03	1.77	1.65	1.60
Methanol cations (29)	1.13	3.03	2.94	2.86	6.49
Anions (51)	0.85	2.33	4.49	2.27	4.38
All ions (80)	0.96	2.61	4.00	2.50	5.25
**All neutral solutes(387)**	1.06	1.01	1.51	1.33	1.44
**All polar protic solvents(467)**	1.05	1.42	2.15	1.59	2.54
Slope	1.002	1.001	0.966	0.995	0.984
Intercept	0.17	0.03	0.18	–0.12	–0.34
*R*^2^	0.998	0.996	0.994	0.995	0.988
# bad solvents^b^	6	2	7	8	7

a The number of entries in the data set is given in parentheses.

b The number of solvents for which RMSE > 1 kcal/mol for neutral solutes.

c A total of 467 entries.

**Table 6:** RMSE of the Solvation Free Energy in kcal/mol for 20 *Polar Aprotic* Solvents Computed Using the ESE-GB-DNN Model in Comparison with uESE and SMD (B3LYP/Def2TZVP) as well as with Semiempirical ESE-PM7 and ESE-EE^c^

Solvent^a^	ESE-GB-DNN	uESE	SMD	ESE-PM7	ESE-EE
Bromoethane (7)	0.56	0.75	0.94	1.05	1.43
2-Methylpyridine (6)	0.64	0.75	0.86	0.71	1.12
*o*-Dichlorobenzene (11)	0.88	0.44	0.92	1.05	1.24
Dichloroethane (39)	0.58	0.77	0.64	0.77	1.32
4-Methyl-2-pentanone (13)	0.90	1.13	0.93	1.21	1.30
Pyridine (7)	0.64	0.70	0.86	0.91	1.02
Cyclohexanone (10)	1.18	1.43	1.08	1.28	1.05
Acetophenone (9)	0.63	0.91	0.78	0.87	0.94
Butanone (13)	0.65	1.11	1.57	1.16	1.01
Benzonitrile (7)	0.58	0.68	0.98	1.13	0.88
*o*-Nitrotoluene (6)	0.91	0.24	0.56	0.60	0.69
Nitroethane (7)	0.40	0.37	0.70	0.84	0.80
Nitrobenzene (15)	0.75	0.32	0.74	0.73	0.85
Acetonitrile neutral solutes (7)	0.44	1.00	0.93	1.21	1.35
Cations (39)	0.46	2.41	10.45	4.01	6.17
Anions (30)	0.81	2.50	3.47	1.96	3.87
All ions (69)	0.63	2.45	8.18	3.28	5.30
Nitromethane (7)	0.35	0.74	1.24	0.94	0.87
Dimethylformamide (7)	0.78	0.75	0.86	0.90	0.84
Dimethylacetamide (7)	0.80	0.82	0.94	0.89	0.87
Sulfolane (7)	0.65	0.65	1.64	1.04	1.03
Dimethyl sulfoxide neutral solutes (7)	0.95	0.94	1.04	2.59	1.90
Cations (4)	0.53	2.58	8.61	2.53	5.78
Anions (66)	0.47	2.71	4.41	3.95	6.37
Methyl formamide (7)	1.03	1.02	0.94	1.15	1.73
**All neutral solutes(199)**	0.73	0.83	0.96	1.07	1.17
**All polar*****aprotic(338)***	0.67	1.77	4.35	2.45	3.86
Slope	1.004	1.001	0.946	1.004	0.984
Intercept	0.07	0.05	–1.20	0.02	–0.34
*R*^2^	1.000	0.996	0.977	0.993	0.988
# bad solvents^b^	2	4	5	10	12

a The number of entries in the data set is given in parentheses.

b The number of solvents for which RMSE > 1 kcal/mol for neutral solutes.

c A total of 338 entries.

**Table 7:** RMSE of the Solvation Free Energy in kcal/mol for 57 *Nonpolar* Solvents Computed Using the ESE-GB-DNN Model in Comparison with uESE and SMD (B3LYP/Def2TZVP) as well as with Semiempirical ESE-PM7 and ESE-EE^c^

Solvent^a^	ESE-GB-DNN	uESE	SMD	ESE-PM7	ESE-EE
Pentane (26)	0.40	0.39	0.42	0.50	0.43
Hexane (59)	0.45	0.52	0.74	0.65	0.86
Heptane (69)	0.48	0.55	0.86	0.60	0.75
Isooctane (32)	0.34	0.48	0.56	0.55	0.61
Octane (38)	0.30	0.41	0.52	0.50	0.50
Nonane (26)	0.22	0.30	0.43	0.22	0.39
Decane (39)	0.30	0.48	0.52	0.47	0.57
Undecane (13)	0.48	0.41	0.65	0.46	0.56
Dodecane (8)	0.32	0.41	0.45	0.21	0.41
Cyclohexane (92)	0.63	0.66	0.79	0.68	1.03
Perfluorobenzene (15)	1.17	0.41	0.93	0.46	0.40
Pentadecane (9)	0.46	0.37	0.72	0.16	0.55
Hexadecane (198)	0.68	0.65	1.00	0.71	0.95
Decalin (27)	0.41	0.43	0.88	0.51	0.52
Carbon tetrachloride (79)	0.53	0.49	0.73	0.60	0.78
Isopropyltoluene (6)	0.37	0.32	0.57	0.17	0.16
Mesitylene (7)	0.65	0.37	0.66	0.50	0.30
Tetrachloroethene (10)	0.35	0.35	0.94	0.21	0.26
Benzene (75)	0.81	0.87	1.13	1.05	1.13
*sec*-Butylbenzene (5)	0.34	0.21	0.40	0.21	0.17
*tert*-Butylbenzene (14)	0.40	0.34	0.47	0.44	0.26
Butylbenzene (10)	0.48	0.32	0.62	0.45	0.27
Trimethylbenzene (11)	0.45	0.26	0.56	0.28	0.32
Isopropylbenzene (19)	0.58	0.34	0.49	0.46	0.59
Toluene (51)	0.56	0.40	0.73	0.52	0.58
Triethylamine (7)	0.63	0.68	1.12	0.82	0.70
Xylene (48)	0.60	0.46	0.75	0.53	0.51
Ethylbenzene (29)	0.54	0.40	0.60	0.46	0.49
Carbon disulfide (15)	0.64	0.59	0.88	1.16	0.85
Tetralin (9)	1.40	1.03	1.43	1.17	1.19
Dibutyl ether (15)	0.54	0.75	0.86	0.86	0.50
Diisopropyl ether (22)	0.93	1.07	1.05	1.23	0.85
Hexadecyl iodide (9)	0.34	0.26	0.48	0.22	0.60
Phenyl ether (6)	0.45	0.40	1.23	0.76	0.66
Fluoroctane (6)	0.41	0.07	0.58	0.18	0.12
Ethoxybenzene (7)	0.43	0.44	0.53	0.74	0.59
Anisole (8)	0.44	0.35	0.63	0.75	0.67
Diethyl ether (72)	0.92	1.00	1.14	1.13	1.30
Bromoform (12)	0.42	0.29	0.78	0.44	0.28
Iodobenzene (20)	0.41	0.54	0.49	0.75	0.47
Chloroform (109)	1.05	0.92	1.09	1.15	1.31
Dibromoethane (10)	0.36	0.45	0.79	0.47	0.21
Butyl acetate (22)	0.97	0.73	1.40	0.92	0.79
Bromooctane (5)	0.66	0.21	0.90	0.32	0.10
Bromobenzene (27)	0.39	0.49	0.64	0.70	0.38
Fluorobenzene (7)	0.40	0.58	0.95	0.96	0.67
Chlorobenzene (38)	0.55	0.50	0.79	0.66	0.51
Chlorohexane (11)	0.64	0.23	1.20	0.40	0.27
Ethyl acetate (24)	1.00	1.13	1.36	1.34	1.59
Acetic acid (7)	0.77	0.58	2.58	0.98	1.46
Aniline (10)	1.03	0.92	0.94	1.23	1.54
Dimethylpyridine (6)	0.67	0.71	0.88	0.62	1.06
Tetrahydrofuran (7)	0.72	0.68	0.86	0.97	0.81
Decanol (11)	0.94	0.68	1.48	1.00	0.71
Tributyl phosphate (16)	1.17	0.69	0.62	0.52	0.89
Nonanol (10)	0.78	0.88	0.99	1.44	1.22
Methylene chloride (11)	0.87	0.79	0.82	1.14	0.77
**All nonpolar *(1554)***	0.68	0.64	0.90	0.77	0.87
Slope	0.87	0.908	0.792	0.917	0.855
Intercept	–0.53	–0.47	–0.83	–0.41	–0.73
*R*^2^	0.892	0.899	0.815	0.859	0.818
# bad solvents^b^	5	3	12	10	10

a The number of entries in the data set is given in parentheses.

b The number of solvents for which RMSE > 1 kcal/mol.

c A total of 1554 entries.

For *polar protic* solvents, ESE-GB-DNN has a very good overall accuracy (see Table 5), with a total RMSE noticeably lower than that of all the other methods tested, both DFT-based (uESE and SMD) and the semiempirically based ones. This good average performance is partly due to ions, for which other methods are troublesome, in particular ESE-EE and SMD. Considering neutral solutes only, ESE-GB-DNN is second-best after uESE but still clearly better than the other methods. ESE-GB-DNN yields an RMSE below 1 kcal/mol for fewer solvents (six) than the other methods, with the exception of uESE (see the bottom of Table 5).

**Figure 3:**
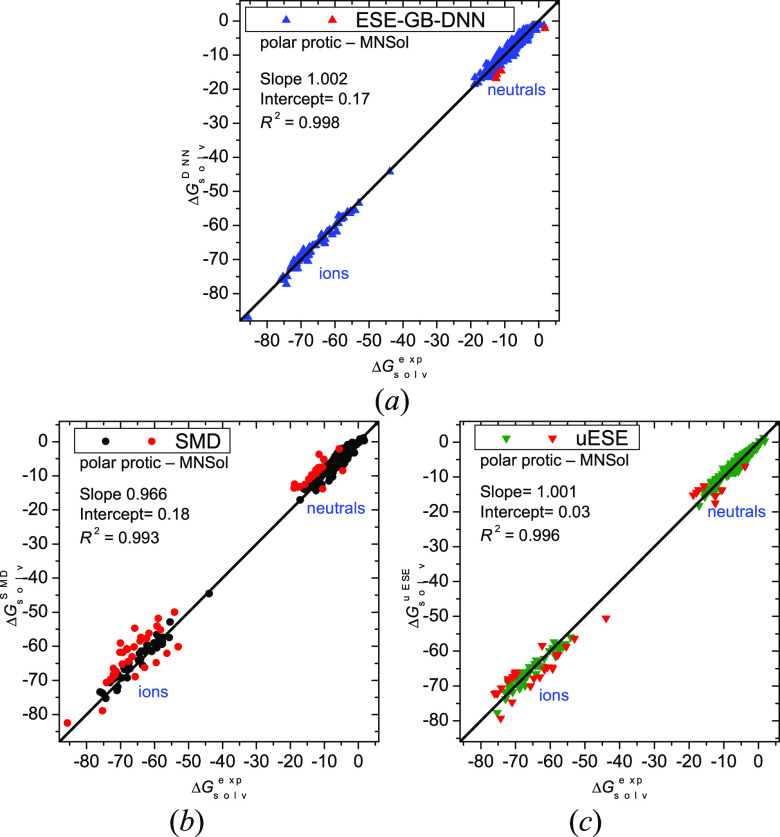
Solvation free energies (in kcal/mol) in nonaqueous *polar protic* solvents for 467 molecules and ions calculated by the ESE-GB-DNN (*a*), SMD (*b*), and uESE (*c*) methods versus reference values. Red points denote outliers with a deviation greater than 3 kcal/mol.

Complete ESE-GB-DNN results for the polar protic solvents are depicted in Figure 3a. Only for 4 entries out of 467 the deviation is beyond 3 kcal/mol. One of these cases is H_2_ with a positive experimental Δ*G*°_solv_. The other three solutes are large organic NO_2_-containing neutral species. The uESE method produces 34 failures (ΔΔ*G*°_solv_ > 3 kcal/mol, Figure 3c), of which 26 are ions and 8 are neutral solutes. The other DFT-based solvation scheme, SMD, fails for a larger number of ions and neutral molecules (as many as 34 ions and 22 neutral molecules, Figure 3b). Therefore, ESE-GB-DNN is much less prone to produce large errors in Δ*G*°_solv_ than SMD or uESE.

The data collected in Table 6 for the *polar aprotic* solvents show a clear superiority of ESE-GB-DNN both for neutral and ionic solutes over the other methods, including uESE. Apart from ions, for which the advantage of ESE-GB-DNN is overwhelming, ESE-GB-DNN works better than uESE and SMD also for neutral solutes for 12 and 16 of 20 solvents, respectively. Compared to ESE-PM7 and ESE-EE, ESE-GB-DNN performs better for nearly all the polar aprotic solvents.

The results concerning nonpolar solvents are summarized in Table 7. The performance of ESE-GB-DNN is convincing, with an RMSE below 0.7 kcal/mol, close to that of uESE. There are five solvents for which RMSE exceeds 1 kcal/mol (tetralin, tributyl phosphate, perfluorobenzene, chloroform, and aniline), as compared to three “bad” solvents in the case of uESE, and at least ten such failures for SMD, ESE-PM7, and ESE-EE. Still, ESE-GB-DNN outperforms uESE and SMD for 18 and 42 out of 57 solvents, correspondingly.

**Figure 4:**
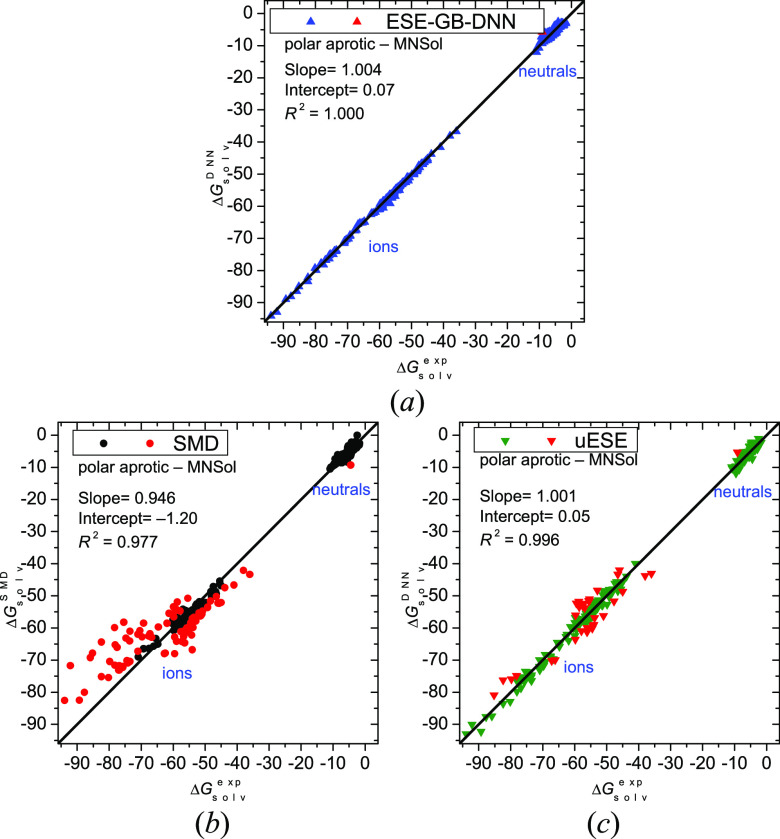
Solvation free energies (in kcal/mol) in nonaqueous *polar aprotic* solvents for 338 molecules and ions calculated by the ESE-GB-DNN (*a*), SMD (*b*), and uESE (*c*) methods versus reference values. Red points denote outliers with a deviation greater than 3 kcal/mol.

Figure 4a illustrates the good quality of ESE-GB-DNN results for the polar aprotic solvents. There is a single outlier only (H_2_O_2_ in cyclohexanone, ΔΔ*G*°_solv_ = 3.4 kcal/mol). In contrast, the uESE and SMD methods display 33 and 85 failures (ΔΔ*G*°_solv_ > 3 kcal/mol), correspondingly, which are mostly ions.

**Figure 5:**
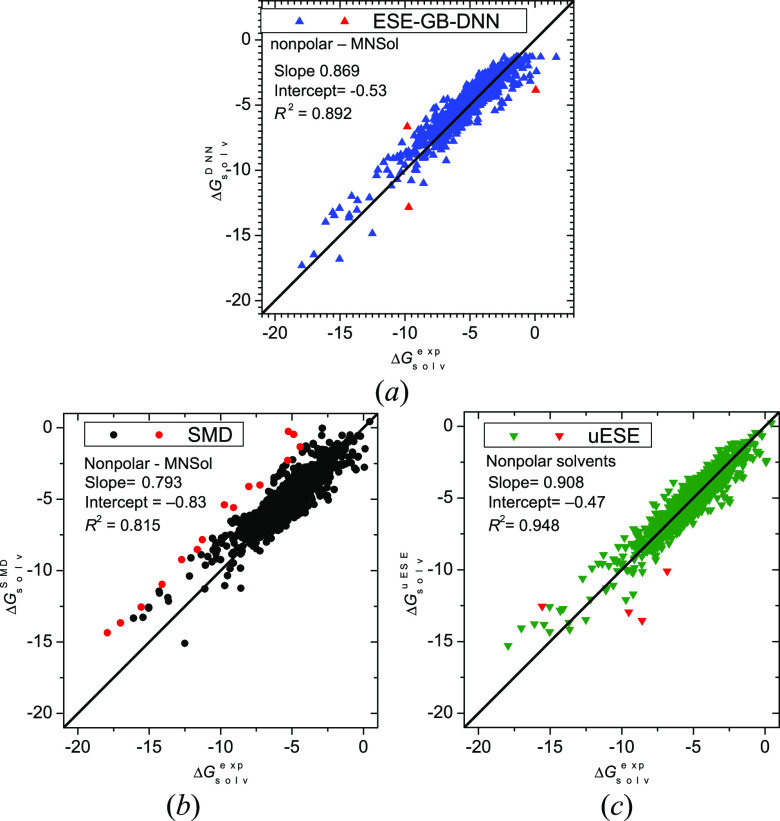
Solvation free energies (in kcal/mol) in nonpolar solvents for 1554 molecules calculated by ESE-GB-DNN (*a*), SMD (*b*), and uESE (*c*) methods versus reference values. Red points denote outliers with a deviation greater than 3 kcal/mol. SMD and uESE results are given for comparison.

Specific results for nonpolar solvent are shown in Figure 5. The present ESE-GB-DNN method exhibits just three problematic cases (ΔΔ*G*°_solv_ > 3 kcal/mol), which compares favorably to 4, 16, 10, and 19 failures for uESE, SMD, ESE-PM7, and ESE-EE methods, respectively. The three mentioned ESE-GB-DNN outliers are H_2_O in tetralin with a positive experimental Δ*G*°_solv_, as well as “0403thi” (1-methylthymine) and “186n” (*N*-methylpyrrolidone) in chloroform. The case of H_2_O, like that previously mentioned of H_2_, highlights a general problem that the current parametrization of ESE-GB-DNN exhibits with solutes with low Δ*G*°_solv_: all the neurons remain deactivated, and the calculated Δ*G*°_solv_ originates solely from the bias of the output layer, which is slightly negative (−1.3 kcal/mol). Therefore, this value is the upper bound of a Δ*G*°_solv_ that ESE-GB-DNN can yield. Upon careful examination, one can observe this fact in the upper-right section of Figure 5a. It should be noted that this problem only pertains to few poorly soluble solutes and thus poses minimal limitations on the practical use of ESE-GB-DNN.

## Conclusions

ESE-GB-DNN proposed in the present work is an uncomplicated, computationally efficient yet accurate technique for solvation free energy evaluation of molecules and ions both in aqueous and nonaqueous solutions, based on a dense neural network (DNN). The only input required for ESE-GB-DNN is the molecular geometry and the total charge of the solute. First, the atomic surfaces {*S*_*I*_} and molecular volume *V*_*tot*_ are estimated using simple geometric formulas, with no need of explicitly constructing the molecular surface. Subsequently, atomic charges {*Q*_*I*_} are computed by a modified version of the electronegativity-equalization (EE) method. Then, {*Q*_*I*_}, van der Waals radii, and interatomic distances are utilized to calculate monatomic and diatomic generalized-Born terms. The latter, together with {*S*_*I*_} and *V*, as well as three solvent features undergo a dimension-reducing linear transformation and are subsequently fed into a DNN that produces Δ*G*°_solv_. Independent DNN trainings were done for aqueous and nonaqueous solutions, respectively.

ESE-GB-DNN exhibits a good accuracy, typically similar or even superior to that of the DFT-based SMD and uESE methods. For neutral solutes in water, polar protic, polar aprotic, and nonpolar solvents, ESE-GB-DNN exhibits an RMSE of 1.30, 1.06, 0.73, and 0.68 kcal/mol, respectively (based on the MNSol database). ESE-GB-DNN is particularly valuable for nonaqueous solutions of ionic solutes, with an RMSE of 0.74 kcal/mol. For ions in water, the RMSE is larger (2.86 kcal/mol), but it is still lower that that produced by alternative methods.

The ESE-GB-DNN scheme is physically justified, since the ANN input features are generalized-Born terms describing the electrostatics, along with surface and volume terms for nonelectrostatic effects. The computational efficiency of ESE-GB-DNN comes first from the use of easily computable electronegativity-equalization atomic charges and second from an inexpensive calculation of the generalized-Born and surface terms.

The ESE-GB-DNN program is devised as a reliable standalone Δ*G*°_solv_ calculator. However, it should be noted that ESE-GB-DNN was not tested for unusual molecular configurations, such as untypical coordination numbers or strongly distorted bonds. Another limitation of the current version is that the elements parametrized are H, C–F, Si–Cl, Br, and I only. Nevertheless, it is extendable to other elements provided that a reliable training database is available.

## Data Availability

The ESE-GB-DNN executable program and a user guide are openly available for download: https://github.com/vyboishchikov/ESE-GB-DNN.

## Supporting Information Available

ESE-GB-DNN parameters (scaling factors, weights, and biases); ESE-GB-DNN-calculated solvation free energies in various solvents; extended statistical data (PDF)
