# Supplementary material for: Predicting Solvation Free Energies Using Electronegativity-Equalization Atomic Charges and a Dense Neural Network: A Generalized-Born Approach
Source: J Chem Theory Comput. 2023 Nov 14;19(22):8340–50. doi: 10.1021/acs.jctc.3c00858 (PMC10853938; doi:10.1021/acs.jctc.3c00858)
Supplement: Supplementary file 1 — ct3c00858_si_001.pdf [file ct3c00858_si_001.pdf]

Predicting Solvation Free Energies using Electronegativity-Equalization Atomic Charges and a Dense Neural Network: a Generalized-Born Approach

Sergei F. Vyboishchikov †

† Institut de Química Computacional i Catàlisi and Departament de Química
Universitat de Girona
Carrer Maria Aurèlia Capmany 69
17003 Girona, Spain.
E-mail: vyboishchikov@googlemail.com

Supporting Information

Content

Part 1. ESE-GB-DNN Dense Neural Network buildup....p. 2
- 61 initial input vector components....p. 2
- 58×49 dimensionality reduction linear transformation – aqueous solutions....p. 3
- 61×52 dimensionality reduction linear transformation – non-aqueous solutions....p. 7
- ESE-GB-DNN structure....p. 11
- ESE-GB-DNN weights and biases – aqueous solutions....p. 12
- ESE-GB-DNN weights and biases – non-aqueous solutions.... p. 15

Part 2. Statistical results....p. 17
- Aqueous solutions....p. 17
- Polar protic solvents....p. 19
- Polar aprotic solvents....p. 19
- Nonpolar solvents....p. 20

Part 3. Individual results....p. 21
- Aqueous solutions....p. 21
- Polar protic solvents....p. 28
- Polar aprotic solvents....p. 34
- Nonpolar solvents....p. 39

Part 4. Statistical results for nonaqueous solutions (training II)....p. 58

Part 1.
ESE-GB-DNN Dense Neural Network buildup

61 initial vector components:

(1) the number of atoms in the solute molecule N;
(2) the total charge of the solute Q_{tot};
(3) the molecular volume of the solute $V = \sum_I V_I$;
(4) the total surface are of the solute $S = \sum_I S_I$;
(5) atomic surface areas S_{H} summed over the hydrogen atoms.
(6) atomic surface areas S_{C} summed over the carbon atoms.
(7) atomic surface areas S_{N} summed over the nitrogen atoms.
(8) atomic surface areas S_{O} summed over the oxygen atoms.
(9) atomic surface areas S_{F} summed over the fluorine atoms.
(10) atomic surface areas S_{S} summed over the sulfur atoms.
(11) atomic surface areas S_{Cl} summed over the chlorine atoms.
(12) atomic surface areas S_{Br} summed over the bromine atoms.
(13) atomic surface areas S_{I} summed over the iodine atoms.
(14) Born-type self-term $E_1^{\text{Born}}(\text{H}) = \sum_{I\in\text{H}} E_I^{\text{self}} = (1-1/\varepsilon)\sum_{I\in\text{H}} Q_I^2/R_I$ for the hydrogen atoms.
(15) Born-type self-term $E_1^{\text{Born}}(\text{C}) = \sum_{I\in\text{C}} E_I^{\text{self}} = (1-1/\varepsilon)\sum_{I\in\text{C}} Q_I^2/R_I$ for the carbon atoms.
(16) Born-type self-term $E_1^{\text{Born}}(\text{N}) = \sum_{I\in\text{N}} E_I^{\text{self}} = (1-1/\varepsilon)\sum_{I\in\text{N}} Q_I^2/R_I$ for the nitrogen atoms.
(17) Born-type self self-term $E_1^{\text{Born}}(\text{O}) = \sum_{I\in\text{O}} E_I^{\text{self}} = (1-1/\varepsilon)\sum_{I\in\text{O}} Q_I^2/R_I$ for the oxygen atoms.
(18) Born-type self-term $E_1^{\text{Born}}(\text{F}) = \sum_{I\in\text{F}} E_I^{\text{self}} = (1-1/\varepsilon)\sum_{I\in\text{F}} Q_I^2/R_I$ for the fluorine atoms.
(19) Born-type self-term $E_1^{\text{Born}}(\text{S}) = \sum_{I\in\text{S}} E_I^{\text{self}} = (1-1/\varepsilon)\sum_{I\in\text{S}} Q_I^2/R_I$ for the sulfur atoms.
(20) Born-type self-term $E_1^{\text{Born}}(\text{Cl}) = \sum_{I\in\text{Cl}} E_I^{\text{self}} = (1-1/\varepsilon)\sum_{I\in\text{Cl}} Q_I^2/R_I$ for the chlorine atoms.
(21) Born-type self-term $E_1^{\text{Born}}(\text{Br}) = \sum_{I\in\text{Br}} E_I^{\text{self}} = (1-1/\varepsilon)\sum_{I\in\text{Br}} Q_I^2/R_I$ for the bromine atoms.
(22) Born-type self-term $E_1^{\text{Born}}(\text{I}) = \sum_{I\in\text{I}} E_I^{\text{self}} = (1-1/\varepsilon)\sum_{I\in\text{I}} Q_I^2/R_I$ for the iodine atoms.
(23–58) ε-dependent Born-type pair terms by elements: $E_2^{\text{Born}}(L_1,L_2) = (1-1/\varepsilon)\sum_{I\in L_1}\sum_{J\in L_2} Q_I Q_J / f_{IJ}$
(23) L_1 = H; L_2 = H
(24) L_1= C; L_2 = C
(25) L_1= C; L_2 = H
(26) L_1= N; L_2 = N
(27) L_1= N; L_2 = H
(28) L_1= N; L_2 = C
(29) L_1= O; L_2 = O
(30) L_1= O; L_2 = H
(31) L_1= O; L_2 = C
(32) L_1= O; L_2 = N
(33) L_1= F; L_2 = F
(34) L_1= F; L_2 = H
(35) L_1= F; L_2 = C
(36) L_1= F; L_2 = N
(37) L_1= F; L_2 = O
(38) L_1= S; L_2 = H
(39) L_1= S; L_2 = C
(40) L_1= S; L_2 = N
(41) L_1= S; L_2 = O
(42) L_1= Cl; L_2 = H
(43) L_1= Cl; L_2 = C
(44) L_1= Cl; L_2 = N
(45) L_1= Cl; L_2 = O
(46) L_1= Cl; L_2 = F
(47) L_1= Cl; L_2 = S
(48) L_1= Br; L_2 = H
(49) L_1= Br; L_2 = C
(50) L_1= Br; L_2 = N
(51) L_1= Br; L_2 = O
(52) L_1= Br; L_2 = F
(53) L_1= Br; L_2 = S

(54) L_1= Br; L_2 = Cl
(55) L_1= I; L_2 = H
(56) L_1= I; L_2 = C
(57) L_1= I; L_2 = N
(58) L_1= I; L_2 = O
(59) the dielectric constant ε of the solvent;
(60) the boiling point (BP) of the solvent;
(61) the number of non-hydrogen atoms in the solvent, which characterizes the solvent molecular size.
Components 59–61 are not used for aqueoues solutions.

58×49 dimensionality reduction linear transformation for aqueous solutions:

```
T(:,1)=(/8.96646e-2,7.70925e-5,5.33628e-1,6.71492e-1,4.36614e-1,2.48432e-1,-6.90174e-4,4.58152e-2,-4.01807e-2,-2.15558e-4,-
  1.49266e-2,-3.01379e-3,-1.69199e-3,-1.43367e-4,-1.09559e-4,-5.26332e-6,-6.89212e-5,1.07319e-5,-3.06893e-6,1.03264e-6,7.06005e-
  7,1.72012e-8,-1.81632e-4,-1.06263e-4,3.73646e-4,-9.77467e-6,1.79687e-5,-1.38237e-5,-2.00143e-5,9.90366e-5,-3.55323e-5,-
  5.35851e-6,4.04970e-6,-6.80254e-6,-1.11464e-5,8.36656e-7,2.19026e-6,-2.62402e-6,-2.43121e-7,3.36710e-6,4.10286e-6,-5.57603e-
  8,1.94451e-7,-1.79254e-7,-4.94773e-8,8.65337e-10,6.07717e-9,-4.22516e-8,1.30771e-7,-2.47541e-8,2.44621e-8,-1.00485e-7,-
  3.47702e-10,-1.60802e-9,-2.01448e-8,3.04953e-8,-1.81594e-9,-8.09561e-9/)
T(:,2)=(/-1.42215e-2,-7.11736e-5,2.01076e-1,2.63568e-1,-4.90593e-1,-1.47376e-1,-6.63080e-3,1.85905e-2,7.85860e-1,-3.20528e-
  3,9.71389e-2,9.22376e-3,-8.62517e-4,1.57198e-4,-8.88777e-5,8.42593e-5,6.14763e-5,-1.79625e-4,-1.58340e-5,-3.29004e-7,-4.79637e-
  7,4.72348e-9,1.75730e-4,6.69257e-7,-4.43320e-4,3.67078e-6,-9.58469e-5,2.74327e-5,-3.90442e-6,-5.60453e-5,7.06410e-5,-1.40194e-
  6,-1.33494e-4,8.61261e-5,3.69365e-4,-7.77564e-6,-2.99875e-5,-9.29589e-6,-1.64782e-6,1.00569e-5,1.77189e-5,1.21038e-6,-9.78408e-
  7,-2.91751e-7,-2.20000e-7,5.02936e-7,2.04215e-8,-3.18779e-7,-1.08611e-6,3.27239e-8,1.69844e-7,1.29478e-6,1.38578e-9,4.82974e-
  9,-4.35154e-8,2.61320e-8,-3.09388e-9,1.00577e-8/)
T(:,3)=(/-3.52122e-2,-3.45319e-4,2.25337e-1,4.45530e-2,-6.41585e-1,4.21483e-1,7.57299e-2,3.23105e-1,-4.31819e-1,2.92620e-
  2,2.42664e-1,1.90494e-2,3.73846e-3,-7.70495e-5,2.43938e-4,-1.86674e-4,-2.29741e-4,1.08838e-4,-5.34602e-5,-4.76758e-6,3.08315e-
  7,-6.10012e-8,2.77425e-4,3.00848e-4,-7.82286e-4,-4.84134e-5,1.23730e-4,1.36021e-4,-7.19733e-5,1.52443e-4,1.05940e-4,-6.93362e-
  5,7.55449e-5,-7.45827e-5,-1.79658e-4,4.48365e-6,1.29171e-5,-2.84362e-5,3.85145e-6,2.64718e-5,5.49418e-5,6.47480e-6,-2.25539e-
  6,-1.17169e-6,-1.67922e-6,-7.39530e-8,2.03293e-8,-7.50615e-8,3.02662e-7,1.98655e-7,1.23060e-7,-5.14893e-7,-3.14733e-
  10,6.75783e-9,-7.00161e-8,5.84081e-9,-4.48669e-9,1.05423e-7/)
T(:,4)=(/-5.42302e-3,-4.64640e-4,-6.61942e-2,1.66786e-1,9.20060e-2,-6.16284e-1,-5.56226e-2,7.32302e-1,-1.33352e-1,2.33322e-
  3,1.49200e-1,-9.41481e-3,1.12793e-4,-2.73961e-4,-1.37909e-5,1.91696e-4,-7.54014e-4,3.13441e-5,-7.19582e-5,-3.71939e-6,-
  3.46912e-7,-1.13322e-7,-2.42813e-4,6.94246e-5,-6.61302e-6,4.75666e-5,-1.20811e-4,1.68487e-5,-2.21751e-4,7.96045e-4,-4.03338e-
  6,-5.94093e-5,2.12997e-5,-1.67790e-5,-4.95306e-5,3.65683e-6,-8.99006e-6,-4.23304e-5,6.58304e-6,3.13193e-5,7.91123e-5,3.10853e-
  6,-7.38789e-7,-1.57919e-7,-1.21705e-6,-7.91199e-8,5.02539e-8,-3.20979e-7,2.31300e-7,2.23677e-7,-3.30102e-8,-9.48472e-
  8,5.75735e-10,5.54439e-9,-4.36472e-8,-7.16329e-8,-2.97955e-8,1.44193e-7/)
T(:,5)=(/-2.01113e-2,3.21006e-4,3.60042e-2,7.27801e-2,5.14823e-2,-2.49917e-1,-3.56577e-2,-4.31851e-1,-1.43614e-1,2.12582e-
  2,8.47933e-1,7.49069e-3,-1.65670e-3,2.66076e-4,8.03516e-5,7.79153e-5,4.31752e-4,3.01696e-5,3.26188e-5,-1.98998e-5,1.26369e-
  7,6.73294e-8,5.62151e-6,-9.50740e-5,3.30359e-4,-2.65092e-6,-6.17943e-5,-7.52745e-5,1.06848e-4,-3.86274e-4,-7.18843e-5,5.15056e-
  5,3.14019e-5,-1.70717e-5,-9.34980e-5,4.36481e-6,1.66434e-5,2.55392e-5,-5.03525e-6,-1.55333e-5,-4.21746e-5,2.51490e-5,-1.19666e-
  5,-3.69939e-6,-4.20588e-6,-4.22980e-7,-1.29786e-8,3.83576e-7,-5.66088e-8,-1.22046e-7,-9.63978e-8,-1.30068e-7,6.52761e-
  10,2.54766e-8,7.54320e-8,4.00816e-10,9.27909e-9,-1.09781e-7/)
T(:,6)=(/-2.77175e-2,1.28822e-4,1.28340e-1,1.30535e-1,-1.63424e-1,-3.32080e-1,3.56144e-1,-2.32665e-1,-2.00214e-1,6.99860e-1,-
  2.62145e-1,2.20565e-1,2.23871e-2,-3.34812e-4,-1.29778e-6,-1.39095e-3,4.83774e-4,5.44989e-5,-1.77891e-4,9.25872e-6,-1.11189e-5,-
  1.76912e-7,-8.32950e-5,9.78646e-5,-5.19860e-4,-3.94345e-4,1.37650e-3,4.33358e-4,6.13994e-5,-3.87911e-4,6.85451e-5,-1.21974e-
  4,2.21478e-5,-4.35685e-5,-4.67624e-5,-6.15048e-6,1.27318e-5,4.20668e-5,-2.63357e-5,2.47701e-5,6.85099e-5,-6.98859e-6,5.05747e-
  6,-1.12007e-6,9.31639e-7,5.15544e-8,-2.61532e-7,3.91361e-6,-6.93223e-6,1.20911e-6,2.44834e-7,2.73720e-6,1.53273e-8,1.37335e-
  8,3.08066e-7,-4.11365e-7,8.38931e-9,5.52230e-8/)
T(:,7)=(/1.26418e-2,5.28478e-4,1.80436e-2,6.23126e-2,-3.54440e-2,-1.44098e-1,6.61804e-1,-7.31979e-2,-6.73059e-2,-6.13144e-1,-
  5.49650e-2,3.82839e-1,1.38456e-2,-8.58915e-4,-1.55140e-4,-2.42133e-3,3.49091e-4,1.94053e-5,1.15365e-4,3.37099e-6,-2.05451e-5,-
  1.32403e-7,-3.91488e-4,5.97436e-5,-6.35484e-4,-6.32131e-4,2.37361e-3,6.57308e-4,6.22780e-5,-2.03860e-4,1.82542e-4,-2.04080e-
  4,1.26510e-5,-1.50884e-5,-2.14256e-5,-1.84408e-5,1.57634e-5,-7.56679e-5,1.66667e-5,3.89898e-5,-1.79056e-5,-2.62269e-6,2.85379e-
  6,-2.44693e-6,1.38416e-6,-8.12522e-8,3.01050e-7,7.33470e-6,-1.23958e-5,2.27508e-6,3.83543e-7,4.52474e-6,-1.53957e-9,3.08651e-
  8,1.71936e-7,-2.93310e-7,1.17538e-8,4.83961e-8/)
T(:,8)=(/-2.78310e-2,-3.92580e-4,4.88689e-2,3.37254e-3,-4.55239e-2,-2.10738e-2,-5.53869e-1,-3.65910e-2,-5.24061e-2,-5.97172e-2,-
  6.30141e-2,8.22024e-1,1.35151e-2,8.23565e-4,1.84786e-4,2.03782e-3,-2.16692e-4,7.16659e-7,6.27299e-5,6.46438e-9,-4.84797e-5,-
  1.05315e-7,3.15155e-4,-1.32718e-4,6.97161e-4,5.23894e-4,-2.03028e-3,-5.41065e-4,-2.27259e-5,1.37432e-4,-1.37788e-4,1.75994e-
  4,8.54658e-6,-1.89683e-5,-4.02763e-5,2.09999e-5,9.74896e-7,3.13796e-5,1.74355e-6,-4.29594e-5,-5.69415e-5,-1.25469e-6,-1.91243e-
  7,1.74998e-6,-4.94110e-8,-5.73436e-7,-7.56086e-8,1.81747e-5,-2.66423e-5,2.26908e-6,-1.46743e-7,1.05859e-5,1.89474e-8,7.44072e-
  8,1.96247e-7,-1.89127e-7,-8.04545e-9,6.52051e-8/)
T(:,9)=(/1.33173e-1,1.08369e-3,7.26167e-1,-5.02216e-1,2.14848e-3,-2.60661e-1,-9.91504e-2,-3.85649e-2,-5.00975e-2,-1.33362e-1,-
  8.87363e-2,-1.35928e-1,2.77147e-1,1.23335e-4,4.32553e-4,-2.32650e-5,1.44755e-4,2.27326e-5,-3.96614e-5,5.02319e-6,9.95090e-6,-
  2.45910e-6,3.47636e-4,-2.84744e-4,-2.92734e-4,1.13827e-5,9.11318e-5,-4.35524e-5,9.90813e-5,-3.18699e-4,1.19776e-4,-1.81754e-5,-
  4.04677e-6,-4.68424e-5,4.18910e-5,5.17334e-6,1.04119e-5,-7.13634e-5,1.54058e-5,3.59762e-5,8.88053e-5,-2.50810e-6,2.63081e-6,-
  3.72152e-6,2.43905e-6,6.55664e-7,4.22033e-7,-4.45052e-6,3.68949e-6,5.34487e-7,1.43459e-6,-1.53081e-6,-3.86369e-9,-1.77864e-
  8,3.62025e-6,-4.69770e-6,1.62200e-8,1.35307e-6/)
T(:,10)=(/-9.16562e-2,-1.21432e-4,-2.20810e-1,2.62418e-1,-1.00178e-1,-2.24845e-2,-9.29995e-2,-9.48501e-2,-8.70264e-2,-8.29952e-
  2,-7.81268e-2,-9.64827e-2,9.03281e-1,3.77500e-4,-9.43366e-5,6.28110e-4,1.23992e-4,2.01048e-5,7.64811e-5,8.50211e-7,5.58448e-6,-
  7.65741e-6,-2.09159e-5,-3.45959e-6,4.50115e-4,1.04389e-4,-6.50422e-4,-1.71502e-4,-2.48499e-5,1.59058e-5,-1.00523e-4,8.42984e-
  5,9.24982e-6,-9.34024e-6,-3.44267e-5,5.02733e-6,6.61005e-6,3.81872e-5,-7.68557e-6,-2.72834e-5,-8.01013e-5,-8.12331e-7,7.47462e-
  8,-1.67380e-6,-7.65889e-7,-1.53506e-7,-1.56052e-7,-1.29262e-6,3.45358e-6,-9.53711e-7,-6.15222e-7,-1.25915e-6,-3.67372e-9,-
  9.03248e-9,1.09213e-5,-1.45936e-5,1.70567e-7,4.11907e-6/)
T(:,11)=(/-1.59503e-1,1.19092e-3,1.86272e-2,3.06312e-1,-2.96663e-1,-3.07894e-1,-3.10271e-1,-3.09283e-1,-3.07526e-1,-3.23202e-1,-
  3.15988e-1,-3.16860e-1,-3.26109e-1,1.69975e-3,1.27365e-3,8.40626e-5,-4.34180e-4,1.03216e-5,4.39877e-4,-1.56028e-4,1.68321e-
  5,2.86246e-6,8.03625e-4,8.61430e-4,-3.35847e-5,-1.16997e-3,1.39633e-4,-3.27807e-3,-6.81564e-4,1.13410e-4,-1.80909e-3,6.99616e-
  4,-5.17988e-5,-2.16613e-4,1.27007e-4,1.67005e-5,8.21086e-5,3.51565e-4,-1.15794e-4,-1.81468e-4,-6.95658e-4,1.72850e-4,-6.54488e-
  5,-3.82967e-4,-4.02249e-5,8.38644e-7,-3.49850e-6,-6.14040e-6,1.37054e-5,-2.68710e-6,-1.49869e-6,-5.78087e-6,-1.77306e-8,-
  7.04113e-8,-4.13290e-6,5.59965e-6,4.84996e-8,-1.52683e-6/)
T(:,12)=(/3.98716e-1,-9.08051e-1,-6.13539e-2,3.81782e-2,-5.50792e-2,-1.86181e-2,-2.39232e-2,-2.50193e-2,-2.60719e-2,-6.80452e-
  3,-1.09546e-2,-7.32856e-3,-2.33125e-3,5.40059e-2,-8.86902e-3,1.28469e-2,-2.76683e-2,-2.10236e-3,5.42274e-3,-1.93249e-3,-
  1.46567e-3,6.98145e-6,2.82814e-2,-7.34472e-4,-3.33960e-3,-4.59494e-3,-2.80617e-2,2.07277e-3,-2.23636e-3,-3.59770e-3,-3.74698e-
  3,2.63239e-3,-3.51171e-4,3.59967e-4,1.42556e-5,2.89938e-4,-3.77741e-4,5.61512e-3,-1.25583e-3,-4.02446e-3,-9.17340e-3,2.67384e-
  4,-4.69464e-5,-2.39120e-4,-3.80610e-4,-3.64742e-6,-4.60832e-6,1.12364e-4,1.39016e-5,7.67359e-6,-1.43519e-4,-1.68688e-
  5,3.29473e-8,2.85500e-8,7.57776e-6,-3.79334e-6,-4.14624e-7,-1.32620e-5/)
T(:,13)=(/-8.79664e-1,-4.11723e-1,1.37430e-1,-8.47793e-2,1.21338e-1,3.94599e-2,5.37938e-2,5.27131e-2,5.67068e-2,1.32765e-
  2,2.32476e-2,1.47211e-2,4.66226e-3,2.72709e-3,-2.65607e-4,3.27013e-3,-2.39557e-2,-8.17228e-4,-1.87554e-2,-8.56527e-4,-3.18499e-
  4,-1.47823e-5,7.96446e-4,-2.32594e-2,4.48124e-2,9.78054e-3,-7.40352e-3,-9.52465e-3,-3.05838e-3,-1.45953e-3,-4.49359e-3,-
  3.69671e-3,2.24952e-4,-1.01118e-3,-6.78093e-4,-7.06706e-4,-7.80811e-4,-1.21807e-2,1.64807e-3,9.75993e-3,2.11552e-2,-1.86121e-
  4,-4.32150e-5,5.66810e-4,-8.94517e-5,5.75836e-6,1.31857e-5,5.44527e-5,1.57659e-5,-3.34266e-5,-6.68838e-5,1.50253e-5,-2.02903e-
  7,-4.71012e-7,-1.38976e-5,8.97664e-6,1.93064e-6,2.86219e-5/)
T(:,14)=(/9.36320e-4,4.79201e-2,3.00881e-4,1.10277e-5,-3.45647e-4,-5.78766e-4,4.96573e-3,2.20554e-4,-3.83555e-4,-2.28907e-4,-
  3.67599e-4,-3.75054e-4,-6.60508e-4,3.39655e-1,-9.70773e-3,5.32193e-1,1.31968e-1,3.51837e-3,1.57501e-2,1.46869e-3,9.63981e-4,-
  4.02892e-5,1.27846e-1,-9.14871e-2,2.84138e-1,1.03931e-1,-6.55502e-1,-8.26901e-2,1.96895e-2,-1.31661e-1,-4.82689e-2,8.95955e-
  2,1.74267e-3,-7.32829e-3,1.19068e-3,2.38908e-3,1.15723e-3,9.23889e-3,-7.54607e-4,-1.06945e-2,-1.37738e-2,-7.85677e-4,-7.82294e-
```

```
  5,1.20337e-3,5.12391e-4,4.94605e-5,-1.37423e-5,1.82752e-5,-8.01853e-5,-5.79346e-5,1.02719e-4,4.93134e-5,1.60939e-7,5.15239e-
  7,2.45813e-6,-3.26948e-5,-9.61872e-6,3.61241e-5/)
T(:,15)=(/-4.68404e-4,1.09245e-2,4.76074e-4,-3.54937e-4,9.16504e-5,-7.11070e-5,1.77725e-3,-1.34400e-3,1.04582e-4,1.12055e-
  4,4.88115e-5,2.10058e-5,1.15655e-4,-1.89429e-1,7.57467e-2,2.16273e-1,-5.27884e-1,-1.53953e-3,1.74268e-2,7.20134e-4,-6.59192e-
  4,1.14657e-4,-9.74291e-2,1.18394e-1,-1.55850e-1,2.70607e-2,-2.62865e-1,4.59908e-2,-1.20677e-1,6.90482e-1,-1.44495e-1,1.22012e-
  2,3.02428e-5,4.57862e-3,-7.59446e-3,9.67791e-4,1.98695e-3,5.93132e-3,9.24208e-4,-8.63393e-3,-1.46191e-2,2.30112e-4,5.75997e-
  4,8.73891e-4,-1.23859e-3,-4.49287e-5,2.43699e-6,1.97149e-4,3.03441e-4,-3.30249e-5,-5.41898e-4,-2.86379e-5,3.69167e-7,7.14970e-
  7,3.08350e-5,6.52268e-5,3.08695e-5,-1.50642e-4/)
T(:,16)=(/8.11674e-3,7.67669e-3,-1.18905e-3,1.64423e-3,-2.47143e-3,-1.29744e-3,-2.13720e-3,-1.58705e-3,-1.70070e-3,-1.11668e-3,-
  1.19016e-3,-1.19429e-3,-1.10031e-3,-1.02902e-1,-4.42114e-1,-2.73089e-2,-8.38655e-2,1.91151e-2,2.33215e-2,-6.47671e-4,-1.23184e-
  3,2.63313e-5,-2.28524e-2,-3.88299e-1,2.14818e-1,-1.55724e-1,-8.56770e-2,7.08584e-1,3.43264e-3,3.52459e-2,1.40505e-1,-1.03782e-
  1,-2.05805e-2,-6.57161e-2,1.29714e-1,4.03960e-4,-3.11832e-4,9.86777e-3,-4.35266e-3,-1.09848e-2,-1.99225e-2,-1.82328e-
  3,4.54045e-4,-1.26853e-4,6.75228e-5,5.23779e-4,-3.07332e-5,-5.92750e-4,-1.20793e-3,1.22668e-3,5.01355e-4,2.86175e-4,-8.71371e-
  7,2.64903e-7,-1.48902e-5,-2.40151e-5,5.02756e-5,-3.36567e-5/)
T(:,17)=(/4.43104e-2,-8.89125e-3,-6.55092e-3,3.71004e-3,-6.80639e-3,-1.66859e-3,-2.25645e-3,-2.42360e-3,-2.68061e-3,-2.82217e-
  4,-9.81924e-4,-5.76256e-4,-1.09958e-4,-2.15375e-1,-4.38748e-2,-7.42598e-2,-6.38402e-2,2.61920e-2,4.06351e-2,-6.73290e-3,-
  7.96928e-3,5.05074e-5,-3.54020e-1,-2.58919e-1,6.72399e-1,1.38134e-1,1.47389e-1,-4.02484e-1,-1.82521e-2,1.19614e-1,-9.25214e-
  2,1.00590e-1,-3.13415e-2,-1.10472e-1,1.93513e-1,3.88532e-3,8.30943e-3,2.69958e-2,1.93101e-2,-3.98794e-2,-5.87564e-2,-2.99386e-
  3,3.44694e-4,-1.71104e-3,1.26161e-4,1.25460e-3,-6.07946e-5,2.90696e-4,-5.46625e-4,-5.24822e-4,-8.05705e-4,1.37591e-3,2.52025e-
  6,1.33083e-5,-5.24083e-5,4.28577e-5,-2.84520e-5,-8.63034e-5/)
T(:,18)=(/3.53418e-2,2.10736e-2,-5.59847e-3,3.37738e-3,-4.91183e-3,-1.46265e-3,-2.14818e-3,-2.19185e-3,-2.18242e-3,-6.06756e-4,-
  8.89103e-4,-5.24522e-4,-1.24282e-4,1.64018e-1,5.86389e-3,-1.04829e-1,-1.94707e-1,1.77775e-2,-5.28003e-1,4.44241e-3,1.77887e-
  4,1.19080e-4,7.30693e-2,7.56087e-3,1.71638e-1,-6.35513e-2,1.46490e-2,1.74895e-2,-2.18044e-2,-4.68191e-2,-2.85471e-1,-1.43644e-
  1,3.26370e-3,-2.55371e-2,1.07310e-2,-4.67170e-3,-1.12021e-2,-3.29034e-1,8.08492e-2,2.43043e-1,5.69423e-1,-5.61923e-4,5.60924e-
  4,-3.55258e-3,7.95815e-7,2.47742e-4,5.25950e-4,-8.71608e-5,-4.26351e-4,7.43323e-5,-3.12652e-5,4.82179e-4,9.56582e-7,7.44583e-
  6,1.15883e-4,-4.64934e-5,5.40396e-6,-2.32155e-4/)
T(:,19)=(/-1.02412e-3,2.00389e-2,7.18575e-5,9.50492e-6,3.11473e-4,-4.02582e-5,5.01237e-4,-2.86246e-4,-2.14602e-4,-3.28103e-
  6,3.51819e-5,1.50216e-5,-7.58002e-6,1.63274e-1,-3.16690e-1,1.50754e-2,-2.58871e-1,5.36340e-2,-4.72192e-2,7.56399e-3,5.00904e-
  3,1.62839e-4,1.70800e-1,-6.85366e-2,-1.75621e-1,1.50180e-1,3.78142e-3,-3.75169e-1,-8.50117e-2,1.83881e-2,6.08283e-1,-2.34217e-
  1,-6.26275e-2,-1.34371e-1,3.24591e-1,1.54942e-2,-3.54542e-2,-4.12002e-2,-2.21891e-2,6.35103e-2,6.84101e-2,2.23489e-3,-7.79944e-
  4,-1.22809e-3,-2.13407e-3,1.03842e-3,1.31696e-4,6.46496e-4,-1.40943e-3,1.52827e-5,4.31079e-4,4.86374e-4,-4.32253e-6,-1.01204e-
  5,1.48865e-4,-3.06307e-5,5.97781e-5,-2.52654e-4/)
T(:,20)=(/-2.16633e-2,1.89310e-2,3.24318e-3,-2.69661e-3,3.92538e-3,1.72646e-3,1.74810e-3,2.18183e-3,1.86306e-3,1.04982e-
  3,1.30846e-3,1.09324e-3,8.06884e-4,3.62384e-1,-2.19379e-2,-8.39390e-2,-3.83350e-2,8.88496e-2,1.36470e-1,9.90553e-3,8.90833e-3,-
  2.43516e-4,1.55288e-1,-1.01622e-1,-2.00341e-1,-1.58544e-1,1.14173e-1,-5.15850e-3,4.88525e-2,5.11640e-2,-4.63370e-1,2.20292e-1,-
  1.33483e-1,-2.59816e-1,5.83757e-1,1.39837e-2,4.45376e-2,6.27313e-2,1.81380e-2,-7.79578e-2,-1.11526e-1,-1.00569e-3,-2.09050e-
  3,2.48038e-3,1.94833e-3,1.95837e-3,-1.36135e-4,-4.00655e-4,-2.22875e-3,-3.31189e-4,3.37425e-4,2.72564e-3,-4.40608e-6,-2.74273e-
  6,-5.94795e-5,-2.65278e-5,-3.92702e-5,3.49615e-4/)
T(:,21)=(/-6.89698e-3,1.20710e-2,1.34873e-3,-1.12526e-3,1.08308e-3,5.20992e-4,9.19763e-4,6.20333e-4,7.22182e-4,3.90030e-
  4,4.33419e-4,3.84820e-4,3.03775e-4,8.67472e-2,-3.35372e-1,3.89041e-1,-3.97492e-1,-1.39774e-2,6.93794e-2,7.46030e-3,4.53449e-3,-
  7.74836e-5,-2.01663e-1,3.65430e-1,9.50205e-2,-3.97162e-1,2.72246e-1,-5.49271e-2,6.75149e-2,-3.13002e-1,-2.39668e-2,1.54107e-
  1,2.67043e-2,3.58511e-2,-1.12529e-1,1.96781e-2,-2.07700e-2,2.24677e-2,1.19643e-2,-5.24447e-2,-3.76029e-2,-4.42443e-3,2.37764e-
  3,8.49844e-3,-3.21807e-3,1.64691e-4,-6.01781e-6,2.90596e-4,7.80023e-4,-5.82007e-4,-9.28453e-4,2.88310e-4,5.19270e-7,6.99253e-
  6,-7.71973e-5,4.23740e-5,-3.32834e-5,1.23448e-4/)
T(:,22)=(/-1.24132e-2,4.28714e-3,2.33208e-3,-1.54132e-3,1.65791e-3,5.37436e-4,4.29609e-4,8.59212e-4,8.39475e-4,2.22061e-
  4,3.58503e-4,2.23527e-4,7.74513e-5,2.56454e-1,-5.23245e-1,-3.76652e-1,1.90283e-1,-2.07442e-3,8.34372e-2,8.85576e-3,1.13339e-2,-
  1.26760e-4,-9.71849e-2,5.16581e-1,1.39321e-1,2.29773e-1,-1.64374e-1,4.54902e-3,-1.86169e-2,2.08510e-1,-1.60924e-1,-1.18568e-
  1,2.89684e-2,1.74346e-2,-8.66840e-2,-1.93769e-2,2.30210e-2,2.77560e-2,-1.09459e-2,-9.82530e-3,-7.12359e-2,-3.21935e-3,6.24088e-
  4,-4.39067e-3,4.76417e-3,5.15740e-4,-6.15977e-5,-2.54697e-3,3.80613e-4,-2.04266e-5,2.29029e-3,2.84371e-4,-1.16115e-6,9.54187e-
  6,-7.45820e-5,-1.05067e-4,3.27186e-5,1.48137e-4/)
T(:,23)=(/-5.31763e-3,-1.19985e-2,9.19703e-4,-8.04534e-4,8.70706e-4,5.08286e-4,3.05453e-4,5.30077e-4,5.12519e-4,2.20064e-
  4,2.89754e-4,2.37421e-4,1.10378e-4,2.82467e-1,-2.70392e-2,1.82782e-1,4.71397e-1,-9.97013e-3,-1.18495e-1,-1.45873e-2,-7.93876e-
  3,-1.05427e-4,-2.53800e-1,-7.32822e-2,-3.04562e-2,-4.18807e-1,1.73855e-1,-5.19861e-2,-3.32048e-1,4.29051e-1,2.07173e-
  1,1.07310e-1,3.20894e-2,-3.06814e-2,-5.23740e-2,1.50503e-2,-9.77886e-3,-5.55470e-2,-2.30271e-2,5.21813e-2,9.99830e-2,-3.03107e-
  3,-4.10665e-3,2.79004e-3,6.46313e-3,3.32908e-4,1.16262e-4,4.44429e-5,-5.87700e-4,-7.33762e-4,7.88760e-4,6.07559e-4,1.91248e-
  6,2.07845e-5,1.64166e-4,-1.72527e-4,-3.61153e-5,1.64588e-5/)
T(:,24)=(/-1.41091e-3,3.42227e-2,1.37753e-4,-4.16800e-4,5.13513e-4,4.76615e-4,1.70793e-4,4.28726e-4,6.65726e-4,4.85535e-
  4,3.79017e-4,3.65158e-4,3.58214e-4,4.07568e-1,2.17101e-1,-4.50847e-1,-3.68983e-1,5.10065e-2,1.21303e-1,3.95510e-2,2.92245e-2,-
  9.51244e-5,8.01420e-2,-1.16859e-1,1.75615e-1,-1.04479e-1,-1.05017e-1,4.48669e-2,-2.18264e-1,-2.65210e-2,2.28097e-1,3.78162e-
  1,1.21227e-1,2.10789e-2,-2.83576e-1,-1.69548e-2,-3.85990e-2,4.63024e-2,1.32408e-2,-8.65073e-2,-2.58067e-2,-8.12923e-3,6.70617e-
  3,-1.54486e-2,1.14989e-3,1.63759e-3,-3.19794e-5,-4.18977e-3,-7.44406e-5,1.65892e-3,1.95853e-3,9.10335e-4,1.00082e-5,4.00780e-
  5,-2.79475e-4,6.22877e-5,2.63827e-5,1.74827e-4/)
T(:,25)=(/1.59804e-3,-1.53574e-2,-2.20564e-4,1.90653e-4,-2.11328e-4,-2.15868e-4,-3.20089e-4,-2.23268e-4,-3.24634e-4,-1.68505e-
  4,-6.23506e-5,-4.47267e-5,-1.29673e-5,-4.49978e-1,-2.75408e-1,-1.11735e-1,1.27678e-1,-4.44587e-2,-1.87062e-1,-2.08895e-2,-
  7.57277e-3,2.64611e-4,1.83016e-1,1.05905e-1,-1.04755e-1,-7.26354e-2,-2.34659e-1,-9.73166e-2,-2.21825e-1,-8.74544e-2,4.96563e-
  2,6.24438e-1,-7.73415e-2,8.06431e-2,1.43490e-1,-7.36483e-3,-3.30251e-2,-4.14810e-2,7.68096e-2,-1.00903e-1,1.67232e-1,1.66366e-
  2,-5.14126e-3,-1.02047e-2,-2.75430e-3,-2.23520e-3,2.18334e-4,4.06681e-3,1.95072e-3,-1.39112e-3,-2.01949e-3,-2.96651e-3,-
  1.27123e-5,-5.76724e-5,3.36036e-4,6.95603e-5,-7.02730e-5,-3.65314e-4/)
T(:,26)=(/1.97261e-5,4.31603e-3,-3.55823e-5,-2.98065e-4,2.63012e-4,2.94386e-4,1.79251e-4,3.87295e-4,4.00211e-4,3.99485e-
  4,3.25551e-4,3.18667e-4,3.15672e-4,-1.50588e-1,-2.60721e-2,-1.20320e-1,6.26942e-2,2.82227e-2,1.27395e-1,1.98058e-2,6.39684e-3,-
  1.38767e-5,4.88237e-1,4.11697e-2,2.24569e-1,-4.93917e-1,-7.62432e-2,-2.02341e-1,4.33716e-1,2.47303e-1,4.48746e-2,-6.27212e-
  2,4.12634e-2,-6.73356e-4,-8.52845e-2,6.62691e-2,-7.47520e-2,-6.20340e-2,-7.12830e-2,2.53153e-1,-1.07859e-1,6.38177e-3,-
  2.60735e-3,-2.45126e-2,8.50255e-3,9.28816e-4,1.13040e-4,-1.12876e-3,-3.35883e-4,7.45596e-4,-4.29390e-4,1.11253e-3,6.92508e-6,-
  2.82992e-7,-3.04236e-4,2.41557e-4,-9.50944e-5,1.81713e-4/)
T(:,27)=(/-2.28293e-3,-4.40506e-3,3.74887e-4,-2.31849e-4,3.03657e-4,1.09205e-4,-3.73728e-5,1.42021e-4,-9.82715e-5,1.20807e-
  5,4.33585e-5,1.80941e-5,1.16905e-6,2.85964e-2,1.63158e-1,-2.10218e-1,-5.13483e-2,-4.60179e-1,-4.86629e-2,-9.73787e-2,-3.33488e-
  2,-1.92839e-4,-4.46611e-1,9.43213e-2,-7.79914e-2,-1.98538e-1,-3.19914e-1,2.13115e-2,1.32894e-1,-1.42863e-1,1.15452e-1,8.34371e-
  3,-1.64540e-1,6.50735e-2,2.75335e-1,3.57685e-2,-6.65023e-2,-1.39941e-2,-2.04484e-1,3.52610e-1,-1.09601e-1,2.35546e-2,-6.74622e-
  3,-3.10980e-2,-8.77386e-3,-2.51746e-3,-5.47764e-5,2.95007e-3,-2.82313e-3,7.64272e-4,-2.82193e-3,1.77163e-3,-1.24451e-5,-
  3.74669e-5,-1.19430e-4,1.22329e-4,2.93983e-5,3.18987e-4/)
T(:,28)=(/1.44849e-3,8.65986e-3,-2.39732e-4,3.40809e-5,-1.06299e-4,5.93349e-5,1.01597e-4,3.31207e-5,-1.72548e-4,1.92392e-
  4,8.58344e-5,9.10496e-5,1.02947e-4,3.92086e-2,-3.38707e-2,4.88500e-2,-1.33738e-2,-7.58467e-1,3.06946e-1,1.17484e-1,4.96047e-2,-
  1.87728e-5,2.37898e-1,-5.79968e-2,8.90687e-2,2.27450e-2,1.32540e-1,-2.56962e-2,-1.70344e-1,5.57186e-2,-5.31225e-2,-9.46632e-2,-
  9.84345e-2,1.12638e-1,-7.68342e-3,3.42285e-2,1.35745e-1,-1.53969e-1,2.78434e-1,-1.33878e-1,1.28669e-1,-1.55296e-2,9.77370e-
  3,1.03511e-2,8.79990e-3,-8.79022e-4,4.03955e-4,-6.11579e-3,-4.24692e-3,8.51122e-4,4.89313e-3,5.72125e-3,1.66783e-5,3.07283e-5,-
  1.21027e-4,-1.20551e-5,4.45385e-6,3.96072e-5/)
T(:,29)=(/3.26462e-3,-2.86143e-4,-4.93480e-4,1.65479e-4,-3.27403e-4,-3.30112e-5,-5.94799e-5,-9.59132e-5,-2.74697e-5,1.45074e-
  4,4.40171e-5,7.53273e-5,1.08719e-4,-1.08596e-1,9.46205e-3,5.97396e-2,-1.74359e-2,1.96354e-1,3.16184e-1,3.23981e-3,4.49195e-
  3,1.88783e-4,1.33090e-1,6.02014e-2,7.09536e-2,4.05801e-2,5.37756e-2,3.77795e-2,-5.26553e-1,-1.36643e-1,-1.01872e-1,-2.09762e-
  2,-7.94381e-2,3.87322e-2,1.66447e-2,-5.50262e-2,2.49398e-1,-1.58895e-1,-1.85387e-1,5.99722e-1,-1.17025e-1,4.27048e-4,-2.19553e-
  3,1.67009e-2,-2.09940e-3,-1.80483e-3,4.52405e-4,1.69117e-3,-7.78454e-4,-1.51005e-3,2.10383e-4,5.69185e-4,5.20311e-7,-1.59866e-
  5,3.37758e-4,-1.92409e-5,-7.66751e-5,-3.41583e-4/)
T(:,30)=(/-1.62459e-3,-5.30689e-3,2.51510e-4,-1.54418e-4,2.16079e-4,7.35794e-5,1.24234e-5,1.07218e-4,9.83501e-5,1.30261e-
  4,2.93581e-5,2.33798e-5,6.27736e-6,-1.87139e-2,2.35879e-2,-1.03186e-1,1.78727e-2,3.18472e-1,3.89161e-1,-3.93096e-1,-6.72359e-
  2,-1.44308e-4,-2.11920e-1,-4.36161e-2,-8.97217e-2,-1.83743e-1,-1.91467e-1,-8.15648e-2,6.27027e-2,-5.19867e-2,2.26721e-2,-
  1.41844e-1,-1.37103e-1,2.34967e-1,4.44498e-2,5.59132e-2,1.26756e-1,-2.71051e-1,4.20155e-1,-1.41236e-1,2.19272e-1,3.94115e-2,-
  1.67640e-2,-4.03966e-2,-1.37337e-2,-4.37250e-3,8.70155e-4,5.03199e-3,3.29710e-3,1.52151e-3,-4.06634e-3,-6.53362e-3,-3.86711e-
  6,-6.20940e-5,-1.21515e-4,7.79161e-5,6.45416e-5,2.82131e-4/)
T(:,31)=(/7.91237e-4,4.08747e-3,-1.30727e-4,-2.98822e-7,-1.11469e-5,3.79302e-5,7.69725e-5,2.86217e-5,1.05632e-4,4.00734e-
  5,3.95861e-5,7.08332e-5,8.08610e-5,-2.14155e-2,7.96958e-2,4.14454e-2,-9.14662e-3,-1.56481e-1,-4.55045e-2,-8.30725e-1,1.50888e-
  1,1.80753e-4,1.81074e-1,1.48734e-1,1.34286e-1,3.70961e-2,8.10240e-2,7.97568e-2,-9.77999e-2,1.32914e-2,3.70014e-2,-1.16203e-
```

```
 2,7.12436e-2,-3.05522e-1,-6.27357e-3,3.27968e-2,2.58118e-2,1.20051e-1,-1.66388e-1,-8.38395e-2,4.12609e-2,2.91380e-2,-2.82866e-
 2,-1.89999e-4,-1.92154e-2,4.90059e-3,-4.26894e-4,-9.66016e-3,-8.22500e-3,-4.40664e-4,1.06073e-2,1.03563e-2,1.49895e-5,-
 4.39517e-5,3.14480e-5,2.28055e-5,-3.51139e-5,-2.39505e-4/)
T(:,32)=(/-1.80779e-3,-1.01606e-3,2.88360e-4,-3.64129e-4,4.18950e-4,2.44283e-4,2.20211e-4,2.90039e-4,3.58063e-4,1.27363e-
 4,2.22096e-4,1.82841e-4,1.76309e-4,-8.67375e-2,1.98313e-1,-8.19790e-2,-5.89557e-3,8.56839e-2,-2.11810e-1,1.99809e-1,-9.47350e-
 2,-6.99203e-5,6.93256e-2,2.39595e-1,1.77075e-1,-2.63770e-1,-1.31482e-1,2.21816e-2,-2.01546e-1,-7.11632e-2,8.50994e-2,-2.72294e-
 1,-2.82279e-1,-1.03727e-1,8.78597e-2,1.90774e-1,4.88451e-1,2.11532e-1,-5.28986e-2,-3.35610e-1,-4.70615e-2,2.41484e-2,-9.11522e-
 3,-2.40756e-2,5.00776e-3,-1.56200e-3,-1.06069e-3,2.92797e-3,-1.55704e-2,2.57762e-3,-3.57791e-3,1.46551e-2,-2.97903e-6,-
 3.62911e-5,-3.42356e-4,3.89389e-4,5.22584e-5,3.73269e-4/)
T(:,33)=(/3.10299e-4,-2.58052e-3,-6.01127e-5,1.20139e-4,-9.47495e-5,-1.01426e-4,-9.92829e-5,-1.05389e-4,-2.55136e-5,-6.48255e-
 5,-8.96731e-5,-1.41354e-4,-7.53365e-5,-3.70183e-2,4.99377e-2,1.61402e-2,2.11673e-2,-5.42841e-2,1.93838e-1,2.79201e-2,-8.64273e-
 1,2.24144e-4,9.60515e-3,9.87149e-2,2.22837e-2,4.52915e-2,2.14841e-3,5.96762e-2,1.34057e-3,-1.11847e-2,3.96424e-2,4.05945e-
 2,1.31972e-1,-3.27164e-1,-8.77144e-3,3.76679e-2,-1.12534e-1,-4.38568e-2,-6.67652e-2,-6.87860e-3,1.94982e-1,1.67774e-3,-
 8.55212e-3,4.41618e-3,1.00434e-3,4.65006e-3,7.11444e-4,4.03064e-2,-1.79979e-2,-5.34108e-4,-4.85210e-2,1.93627e-2,8.11050e-6,-
 7.22324e-5,2.83279e-4,-8.31229e-5,-3.44861e-5,-3.73994e-4/)
T(:,34)=(/1.71145e-3,5.20270e-4,-2.43225e-4,1.44098e-4,-2.81358e-4,-6.57400e-5,-8.25667e-5,-9.99216e-5,-2.37378e-4,-3.72112e-5,-
 4.49301e-5,-3.06509e-5,-2.51378e-5,2.38986e-2,1.30831e-1,3.65265e-2,2.09126e-2,9.69433e-2,2.15818e-1,4.81372e-2,4.44912e-2,-
 3.48633e-5,9.80128e-2,1.50853e-1,1.79211e-1,6.63120e-3,8.41078e-2,9.57447e-2,-9.30640e-2,4.06142e-2,2.19300e-2,-7.91378e-2,-
 1.79144e-1,4.33564e-1,2.54200e-1,-1.34459e-2,-4.41766e-1,1.57838e-1,-4.00741e-1,-1.80411e-1,3.52123e-1,1.06649e-2,1.40049e-
 3,8.89448e-3,3.24769e-3,-6.86011e-3,2.89713e-4,-3.60439e-4,4.86312e-3,2.30188e-4,4.21553e-3,-8.92924e-3,-5.96388e-5,-3.35039e-
 5,7.56242e-5,2.43172e-4,-5.19914e-5,6.00269e-5/)
T(:,35)=(/-5.50613e-4,1.22705e-2,9.60464e-5,-1.04601e-4,9.36935e-5,9.43043e-5,1.51088e-4,9.42550e-5,-4.78084e-5,3.78462e-
 5,6.27534e-5,4.03450e-5,4.04154e-5,1.58290e-1,-2.65691e-2,4.59602e-2,-5.33189e-2,-1.88325e-2,-3.04218e-1,-2.52970e-1,-4.16078e-
 1,-5.29345e-4,1.52111e-1,-3.75609e-2,9.14159e-2,9.67711e-2,1.75117e-1,3.46974e-2,8.33308e-2,1.19403e-1,3.94875e-2,9.95523e-2,-
 1.92689e-1,5.39033e-1,7.05498e-2,-2.40212e-1,1.87638e-1,4.90313e-2,8.70088e-2,5.74567e-2,-3.01097e-1,-2.56357e-2,2.48931e-
 2,2.51014e-2,-4.59018e-4,-5.74348e-3,-1.26847e-3,1.58478e-2,3.26043e-4,3.45731e-3,-2.42456e-2,3.29949e-5,-1.86209e-5,3.95945e-
 5,-6.38346e-4,4.94245e-5,5.72148e-6,8.07904e-4/)
T(:,36)=(/-9.58694e-4,-1.13299e-3,1.61641e-4,-7.97010e-5,1.25020e-4,1.64618e-5,5.42980e-5,2.49352e-5,5.21214e-5,-7.84878e-6,-
 1.06870e-6,-1.80518e-5,-2.28158e-5,-7.52252e-3,1.53786e-1,3.22420e-2,2.04136e-2,6.35590e-2,-1.44364e-1,7.25590e-3,-5.73496e-
 3,9.24133e-6,7.31677e-2,2.10843e-1,1.39752e-1,4.89361e-2,4.84308e-2,1.42070e-1,-1.93230e-1,-2.09256e-2,5.94163e-2,-4.94708e-2,-
 6.20645e-2,-3.77339e-2,1.34034e-1,4.29460e-1,-4.92999e-1,-1.84631e-1,4.29516e-1,5.76735e-2,-3.70661e-1,1.11478e-2,-1.53901e-
 2,3.57056e-2,-2.05143e-4,9.67847e-4,-9.25452e-4,-1.64741e-3,-1.02717e-2,-3.06752e-6,6.90735e-3,6.61962e-3,1.25320e-5,-7.39699e-
 5,3.80228e-4,5.86158e-5,3.69220e-5,6.25511e-6/)
T(:,37)=(/-7.82697e-4,8.92136e-3,1.28332e-4,-2.47274e-4,2.53874e-4,2.08257e-4,2.33621e-4,2.22022e-4,1.89854e-4,1.66800e-
 4,1.95429e-4,1.86353e-4,1.76351e-4,4.01888e-2,-2.32984e-1,-4.67114e-2,-5.20247e-2,-3.78008e-2,-3.97297e-2,-1.24095e-1,-
 1.32266e-1,-6.73944e-4,-1.31292e-3,-3.19533e-1,-1.65197e-1,1.05760e-2,-1.20128e-2,-1.73052e-1,-7.85611e-2,-2.89406e-2,-
 2.21303e-1,-3.57208e-2,-1.54019e-1,1.62723e-1,-2.70268e-1,6.85497e-1,-3.21316e-2,1.82794e-1,-2.21136e-1,3.25209e-2,6.45488e-3,-
 7.44270e-2,4.62896e-2,-4.10805e-3,2.80840e-2,-1.74037e-3,-8.77742e-4,-8.67273e-3,1.15884e-2,4.39954e-3,-2.21397e-3,-7.07108e-
 3,-1.12737e-5,1.63579e-4,-1.07388e-3,-9.21390e-5,1.65617e-4,1.06660e-3/)
T(:,38)=(/2.90790e-3,-6.40341e-3,-4.73748e-4,6.98045e-4,-7.89131e-4,-5.10971e-4,-5.18064e-4,-5.77047e-4,-5.62143e-4,-4.13790e-
 4,-4.77188e-4,-4.49546e-4,-4.17952e-4,8.43019e-2,9.13038e-2,9.72961e-2,3.71649e-2,6.65001e-2,8.94850e-2,6.57996e-2,1.15733e-
 1,9.50476e-4,-3.48631e-2,1.54918e-1,7.61349e-2,2.83054e-1,1.61633e-1,2.04752e-1,3.65268e-1,1.45617e-1,2.42327e-1,3.60457e-1,-
 3.12105e-1,-7.89123e-2,-7.49094e-3,3.44806e-1,2.86842e-1,-2.39144e-1,-9.39924e-2,9.34840e-2,1.67301e-1,-5.62250e-2,3.58778e-
 2,8.44058e-2,-3.54579e-2,1.13440e-3,1.82606e-3,9.41628e-3,-8.55379e-3,-6.38793e-3,-1.17109e-2,1.57681e-2,-1.55704e-5,5.80546e-
 5,1.17962e-3,-8.21794e-4,-1.86328e-4,-2.11032e-3/)
T(:,39)=(/1.61848e-4,-1.74705e-3,-1.86169e-5,7.75761e-5,-8.91218e-5,-7.44959e-5,-6.93663e-5,-7.99267e-5,-7.22376e-5,-1.25379e-
 4,-7.34158e-5,-7.30309e-5,-7.17722e-5,6.79091e-3,2.69675e-2,2.12298e-2,1.61454e-2,1.70064e-2,5.75354e-2,1.51035e-2,2.08169e-
 2,3.97842e-4,-2.81267e-3,5.10545e-2,2.79793e-2,2.70385e-2,2.40555e-2,4.10804e-2,4.69003e-2,2.81379e-2,5.14537e-2,4.17272e-2,-
 8.97834e-2,-3.72990e-2,1.16013e-3,8.05802e-3,-4.76245e-4,7.30819e-1,4.50975e-1,3.85055e-1,2.85866e-1,-8.34220e-3,-5.95584e-
 3,5.44276e-2,-7.38337e-3,4.94033e-4,1.89064e-4,5.29699e-3,-1.32531e-2,-1.67429e-3,-2.23772e-3,1.26873e-2,6.11011e-5,-3.07933e-
 5,6.71376e-4,-2.58268e-4,-2.32645e-6,-8.33655e-4/)
T(:,40)=(/1.92750e-5,-2.00161e-3,-7.87853e-6,7.42072e-5,-6.35438e-5,-7.45563e-5,-9.78508e-5,-7.58018e-5,-6.82960e-5,-7.32339e-
 5,-3.99655e-5,-7.07664e-5,-6.82012e-5,-3.44503e-2,2.85488e-2,-2.01409e-2,8.43638e-3,1.37736e-2,8.48772e-3,-4.07363e-2,1.34859e-
 2,8.11784e-4,-1.18699e-2,3.52295e-2,3.61652e-3,-4.98384e-2,-4.59675e-2,-4.43889e-3,-2.21711e-2,-2.21189e-2,1.18425e-2,-
 3.50924e-2,3.94527e-3,-3.98140e-2,3.25605e-2,-7.26451e-2,-2.57641e-2,-1.54668e-2,2.05939e-2,-2.40597e-2,1.62014e-3,-8.10442e-
 1,4.64706e-1,1.96262e-1,2.56661e-1,1.72562e-3,-9.11944e-4,4.87202e-3,-1.30784e-3,-1.76990e-3,-1.27024e-3,-2.06315e-3,-3.84915e-
 5,8.96953e-4,9.79472e-4,4.10533e-4,3.01707e-5,-9.78059e-4/)
T(:,41)=(/2.79658e-4,-1.84780e-4,-4.50793e-5,3.47040e-5,-4.39004e-5,-1.73073e-5,-8.61121e-6,-2.57995e-5,-2.07678e-5,-5.91359e-
 6,-1.43268e-5,-1.13607e-5,-5.73901e-6,6.49772e-3,-2.81345e-3,1.12978e-2,-7.14418e-3,5.58394e-3,2.30953e-3,-7.73271e-3,7.41838e-
 3,7.49448e-4,-4.77552e-3,-2.97095e-3,-3.62359e-3,2.52591e-2,1.43952e-2,8.91726e-3,1.82472e-2,2.61825e-3,7.84716e-3,2.22194e-2,-
 1.31749e-1,-3.87290e-2,-5.05413e-2,-3.99690e-2,-2.50194e-2,-1.36854e-2,1.06626e-3,9.49546e-3,6.30155e-4,-4.86059e-3,-4.27430e-
 1,-1.49845e-1,8.73661e-1,5.08517e-2,-5.57798e-3,1.89052e-2,-3.51857e-2,-6.85402e-4,-1.24726e-2,3.18300e-2,-3.83188e-5,-
 6.77916e-4,1.70353e-4,6.40628e-5,1.13771e-4,-1.13256e-3/)
T(:,42)=(/-7.50687e-5,1.06578e-3,1.20967e-5,-6.24373e-5,6.36981e-5,5.80671e-5,6.31402e-5,6.02330e-5,7.60918e-5,6.23453e-
 5,5.89852e-5,3.76634e-5,5.51296e-5,3.95346e-4,-3.87152e-2,3.39843e-4,-1.52935e-3,1.42605e-2,9.09047e-4,-6.28215e-3,-1.49943e-
 2,-6.80310e-4,8.85798e-4,-5.00214e-2,-2.93571e-2,-5.69044e-3,-1.27959e-3,-3.09270e-2,-1.28617e-2,-2.94135e-3,-3.45463e-2,-
 1.15667e-2,-4.91881e-1,-1.76880e-1,-2.24758e-1,-2.17913e-1,-1.86291e-1,-3.37560e-2,-2.34555e-3,-1.32514e-2,-3.28465e-
 2,8.66627e-3,1.20151e-1,-1.84369e-1,-1.09742e-1,-1.39662e-2,3.37580e-3,-1.92510e-1,-4.77773e-1,6.82670e-2,2.30107e-1,4.47372e-
 1,1.31823e-4,3.28638e-4,-2.83479e-4,-4.43951e-4,4.52883e-5,8.65489e-4/)
T(:,43)=(/-3.74936e-4,-6.04191e-4,5.93082e-5,-9.95327e-5,1.19355e-4,8.06962e-5,1.12640e-4,8.39254e-5,8.93628e-5,6.71142e-
 5,7.48537e-5,7.12681e-5,6.28157e-5,8.45189e-3,1.23712e-2,3.77260e-2,7.52132e-3,2.06371e-3,-7.70350e-5,5.79833e-3,1.93347e-2,-
 7.64463e-4,1.25660e-2,2.78292e-2,1.57801e-2,4.50106e-2,4.26684e-2,3.39861e-2,1.42799e-3,1.18139e-2,1.50720e-2,2.42395e-2,-
 2.41369e-3,-1.12622e-2,1.54800e-2,2.21088e-2,-6.44032e-5,2.04774e-2,2.81625e-2,3.06577e-2,6.16085e-3,-3.04923e-2,3.25597e-1,-
 8.84673e-1,9.83695e-3,-3.87648e-2,5.96473e-3,1.83632e-1,1.26625e-1,-6.48771e-2,-1.73228e-1,-1.11771e-1,-9.13630e-5,4.62850e-
 4,1.43712e-3,-1.11435e-3,-2.42449e-4,3.60558e-4/)
T(:,44)=(/-1.14839e-5,1.44990e-3,4.79317e-6,-2.59182e-5,1.99462e-5,2.21313e-5,1.33727e-5,2.63964e-5,3.21304e-5,2.59362e-
 5,2.25909e-5,5.34008e-6,2.23827e-5,6.78368e-4,-2.58297e-2,-1.71672e-2,-4.88519e-3,-1.85074e-3,-4.40217e-3,-1.02417e-2,5.54896e-
 2,-1.37524e-3,1.86385e-3,-3.91218e-2,-1.78774e-2,-2.42250e-2,-1.63265e-2,-3.04205e-2,-2.06102e-2,-6.88773e-3,-3.01808e-2,-
 2.76820e-2,-2.42986e-1,-8.65357e-2,-1.22988e-1,-1.09427e-1,-1.00100e-1,-1.76165e-2,-9.58361e-3,-2.35619e-2,-2.84755e-
 2,9.09023e-3,-5.94841e-2,1.99052e-1,-8.06370e-2,2.64542e-2,-4.23369e-4,7.13466e-1,-1.58061e-1,-1.20234e-1,-5.33314e-1,3.52764e-
 2,-2.04929e-4,-1.93802e-3,-2.31436e-3,-2.12913e-5,2.69911e-4,2.41343e-3/)
T(:,45)=(/7.28705e-5,1.30778e-3,-1.13750e-5,-2.88406e-5,2.40027e-5,3.10198e-5,2.60855e-5,3.28502e-5,5.40006e-5,3.94983e-
 5,3.40748e-5,5.95857e-5,3.45763e-5,-2.97702e-4,-2.58248e-2,-8.92763e-3,-3.07544e-3,2.12833e-3,-2.80628e-4,-8.68437e-3,-
 2.84971e-2,-8.33771e-4,5.18214e-3,-3.65096e-2,-1.77308e-2,-1.76442e-2,-9.05322e-3,-2.69452e-2,-1.61790e-2,-3.98835e-3,-
 2.55946e-2,-1.98608e-2,-0.51039,-0.21091,-0.22444,-0.21764,-0.17632,-2.77120e-2,5.88702e-3,-1.77661e-2,-3.28574e-2,-1.02864e-
 2,-4.20094e-2,6.51369e-2,-8.31452e-2,5.03825e-2,1.59141e-3,-2.17839e-1,5.95734e-1,-4.26984e-2,-3.15399e-2,-3.70780e-1,-
 1.75503e-4,1.54202e-3,-7.66486e-4,-9.61064e-4,1.75983e-4,9.80288e-4/)
T(:,46)=(/-6.63872e-5,-9.57574e-5,1.09618e-5,-1.89051e-5,2.29344e-5,1.46361e-5,2.01676e-5,1.58750e-5,1.05542e-5,1.25364e-
 5,1.82171e-5,1.00007e-5,1.28433e-5,-1.46874e-3,2.59936e-3,4.07463e-3,1.48558e-3,-1.05409e-4,-1.43050e-3,9.02420e-3,-4.85966e-
 3,1.52717e-4,3.51094e-3,5.15169e-3,2.15879e-3,3.29406e-3,3.51205e-3,3.53439e-3,-1.11940e-3,7.76626e-4,1.37611e-3,1.29467e-
 3,1.59762e-2,2.42305e-2,1.48832e-2,9.97292e-3,7.76438e-3,3.87657e-3,3.76210e-4,1.40611e-3,1.32302e-3,-4.59677e-1,-5.27907e-1,-
 2.20743e-1,-3.24520e-1,5.38720e-1,1.08722e-2,8.06369e-2,-0.11787,7.80359e-2,0.14043,-0.134011,-2.41659e-4,-1.78492e-3,1.21528e-
 3,4.31654e-4,-1.92053e-4,-7.83782e-6/)
T(:,47)=(/3.64384e-5,2.16402e-4,-6.16173e-6,2.38063e-6,-4.75632e-6,4.59499e-7,1.77735e-9,-2.88340e-7,6.25017e-6,2.71901e-6,-
 5.06170e-7,-1.07869e-5,1.36494e-6,2.48469e-3,-3.64306e-3,1.36478e-4,-1.40432e-3,-2.33454e-3,8.37486e-4,-3.60888e-3,-1.58057e-
 2,-4.24283e-4,8.76133e-4,-4.46700e-3,-1.10397e-3,1.42196e-3,2.39322e-3,-2.55264e-3,3.30297e-3,1.90249e-3,-2.18324e-3,3.11340e-
 3,-7.91801e-2,-4.21752e-2,-3.83059e-2,-2.83662e-2,-1.97589e-2,-4.23977e-3,-3.71507e-4,3.25381e-4,-3.05692e-3,1.25066e-
 1,1.47207e-1,4.44263e-2,7.95306e-2,-1.20274e-1,-1.49410e-3,3.27703e-1,-3.16644e-1,3.12800e-1,4.64194e-1,-6.38617e-1,-8.32316e-
 4,-4.34878e-3,-1.80091e-3,4.58173e-5,-2.58696e-4,5.03868e-4/)
T(:,48)=(/-2.49340e-5,-1.94271e-4,3.76630e-6,-2.00423e-6,3.76120e-6,7.91679e-7,1.53638e-6,8.68276e-7,1.25545e-6,-2.63761e-
 7,4.49209e-7,1.61860e-6,-3.17307e-7,-1.36117e-3,1.11578e-3,2.34577e-3,2.20048e-3,9.11279e-4,1.02396e-3,1.25395e-3,1.94885e-
```

```
  2,2.09067e-3,-2.63986e-4,3.68246e-3,6.06796e-4,1.98572e-3,1.37360e-3,1.21271e-3,-1.83872e-3,-1.05983e-5,8.30454e-4,1.16474e-
  3,7.27333e-3,-7.37981e-4,4.76706e-3,1.38779e-3,-1.07226e-3,-2.91084e-4,1.56187e-3,1.85901e-3,1.40552e-3,-1.18127e-2,-6.20472e-
  3,-1.78407e-2,-6.92284e-3,-1.04539e-2,2.63424e-3,-2.42149e-1,-7.66096e-2,8.25801e-1,-4.99720e-1,-5.06614e-2,3.33049e-
  4,3.28603e-3,6.64986e-3,-2.14861e-3,-1.08810e-3,-5.05871e-3/)
T(:,49)=(/7.57274e-5,-7.35044e-5,-1.18172e-5,9.45135e-6,-1.23430e-5,-5.69660e-6,-6.11601e-6,-7.56664e-6,-6.83922e-6,-3.37464e-
  6,-4.04978e-6,-3.34670e-6,2.00582e-6,-5.12368e-4,5.86991e-4,7.82537e-4,3.40372e-4,3.65707e-4,4.73492e-4,5.66951e-4,7.04470e-4,-
  3.57579e-1,-2.97209e-5,1.25671e-3,3.97891e-4,1.41083e-3,5.53681e-4,1.03603e-3,5.80900e-4,4.03258e-4,1.35052e-3,1.33146e-
  3,1.45108e-3,-8.46436e-4,1.17050e-3,7.11016e-4,8.92860e-4,-3.15297e-4,5.56552e-4,1.11033e-3,1.57154e-3,-7.78171e-3,-7.94468e-
  3,-2.90374e-3,-1.95983e-3,-1.54857e-2,3.65914e-3,-4.11910e-3,-1.31315e-5,6.65520e-3,-3.04303e-3,4.97782e-5,2.25100e-5,1.36270e-
  4,-5.17003e-1,2.82743e-1,-1.14328e-2,7.24079e-1/)
```

61×52 dimensionality reduction linear transformation for non-aqueous solutions:

```
T(:,1)=(/8.81637e-2,1.69153e-3,5.27008e-1,6.54282e-1,4.24754e-1,2.65676e-1,-2.53428e-2,1.64434e-2,-8.95730e-3,-4.73598e-3,-
  5.24481e-3,-7.67821e-3,-1.03616e-3,-6.23030e-5,-9.18623e-5,7.81208e-5,4.08634e-5,3.92028e-6,9.67652e-6,3.43879e-5,2.45734e-
  5,4.49896e-9,-1.00015e-4,-8.27189e-5,2.96942e-4,1.41897e-5,-3.88421e-5,-1.97071e-5,-1.28668e-6,4.40203e-5,-1.21935e-5,8.71974e-
  6,1.49954e-6,2.39897e-7,-2.96949e-6,5.89547e-7,3.17688e-6,3.61746e-6,-2.23240e-6,4.56550e-7,-5.77906e-6,4.91834e-8,-6.28564e-
  7,-4.70271e-8,1.48559e-6,-3.11683e-9,4.68980e-9,-1.45935e-7,-3.96299e-9,2.35121e-8,2.41324e-7,-2.17785e-9,-4.55434e-11,-
  2.04645e-10,-1.25596e-8,1.24409e-8,1.30030e-10,8.90807e-11,-1.84790e-1,-1.02401e-2,6.92328e-3/)
T(:,2)=(/-5.19802e-3,-1.35532e-4,-4.36671e-2,-4.72055e-2,-6.55227e-4,-3.19750e-2,-1.55866e-3,-3.63024e-2,3.67732e-3,1.03467e-
  3,1.55639e-2,1.57562e-3,1.34540e-3,1.12904e-4,8.90560e-6,3.98662e-5,8.53916e-5,-3.47190e-7,5.10434e-7,3.70801e-6,1.53153e-6,-
  8.18112e-9,6.86064e-5,-1.02856e-5,6.88976e-6,-1.26336e-6,-7.53976e-5,5.79315e-6,1.72443e-5,-2.09561e-5,-1.04189e-5,-1.64804e-
  8,-3.72426e-7,1.30399e-7,6.29627e-7,-8.64101e-8,6.67537e-7,1.65793e-6,-1.10927e-6,-1.05472e-6,-3.86374e-6,1.09630e-7,-1.26321e-
  8,-2.10323e-8,1.36501e-6,2.77499e-9,1.05959e-9,1.18528e-7,-8.00418e-8,-2.33677e-8,3.89568e-8,7.22018e-9,-1.65048e-11,6.00424e-
  10,2.21271e-8,-2.44175e-8,1.22784e-9,8.41168e-10,-3.94742e-1,9.14443e-1,3.47306e-2/)
T(:,3)=(/1.41049e-2,-1.25974e-3,1.05889e-1,1.22669e-1,3.31579e-2,6.82834e-2,1.26813e-2,3.21292e-2,-4.06150e-3,1.94567e-3,-
  1.43136e-2,-4.29892e-3,-2.70295e-3,-9.63947e-5,-4.32981e-5,-9.74666e-5,-1.20107e-4,1.07906e-8,-9.28402e-6,1.49202e-6,1.95434e-
  6,1.16726e-8,-7.00781e-5,-1.02267e-5,3.06976e-5,-6.48665e-6,8.14157e-5,-7.27383e-7,-1.81872e-5,7.16684e-6,8.89647e-6,-1.11423e-
  5,3.25797e-7,6.57460e-8,-8.09346e-7,-3.21793e-7,-7.14664e-7,-1.83083e-6,1.73275e-6,3.40237e-6,8.77137e-6,-1.73218e-7,-1.04442e-
  7,2.28766e-8,-1.02859e-6,-5.27496e-9,5.12698e-10,-1.51402e-7,1.15627e-7,2.86433e-9,2.00086e-8,-7.01187e-9,-6.70460e-12,-
  4.50955e-10,-3.23477e-8,3.35176e-8,-4.34742e-10,-2.97833e-10,8.96659e-1,4.02825e-1,-8.25728e-3/)
T(:,4)=(/-3.20165e-2,-1.61711e-2,2.60028e-1,1.76191e-1,-7.60737e-1,2.09198e-1,1.92838e-2,4.93612e-1,5.76851e-2,-5.71235e-
  3,1.60308e-1,2.06414e-3,1.90750e-5,8.67466e-4,-3.55521e-5,2.48708e-4,-7.37423e-4,-1.97875e-5,-1.30695e-5,-1.31899e-5,8.77500e-
  6,5.27177e-9,7.47353e-4,9.92760e-5,-5.24883e-4,-3.39970e-6,-6.21797e-4,5.99712e-5,-1.61652e-4,9.35034e-5,1.34207e-4,-2.18694e-
  5,-8.23664e-6,2.22364e-6,1.52054e-5,-1.63057e-6,-1.10879e-5,-8.05242e-7,7.52721e-6,1.09368e-5,2.58089e-5,4.77377e-6,-1.28567e-
  6,-1.14524e-7,-1.26300e-5,1.94022e-8,1.77581e-8,1.67561e-7,5.31573e-8,1.94573e-8,-6.37067e-7,1.56482e-8,-6.73880e-11,1.25730e-
  9,-1.91352e-8,1.43536e-8,2.85337e-9,1.95479e-9,-6.47971e-2,1.68029e-2,-1.40405e-4/)
T(:,5)=(/-1.27517e-2,3.05602e-3,1.15946e-1,-1.23197e-1,-2.70465e-1,5.72075e-1,1.41357e-1,-7.27557e-1,-1.00694e-2,1.16235e-
  2,1.44081e-1,1.44905e-2,2.84685e-3,3.83804e-5,1.30511e-4,-5.30694e-4,1.18297e-3,7.15663e-6,4.19565e-5,-5.57924e-5,-2.14121e-5,-
  8.13775e-9,2.08499e-4,-3.44952e-5,-3.44064e-4,-9.31633e-5,1.39331e-4,5.64489e-5,2.52719e-4,-3.48790e-4,-5.74592e-5,3.18429e-
  5,2.90706e-6,1.03912e-6,-9.28005e-6,9.35490e-7,1.08771e-5,2.21477e-5,-2.89613e-5,-2.09620e-5,-8.45337e-5,1.78820e-6,-5.87385e-
  6,1.04027e-7,4.26963e-6,1.55655e-8,-7.43771e-9,-1.40669e-6,4.03878e-8,3.14074e-7,1.48209e-6,9.18770e-9,3.71638e-11,1.04930e-
  9,2.72985e-8,-2.44187e-8,-2.01352e-9,-1.37942e-9,1.61103e-3,-1.18763e-2,1.02689e-2/)
T(:,6)=(/-2.21222e-2,4.31006e-3,5.36202e-2,1.81371e-1,-2.66578e-2,-4.54001e-1,-6.50648e-2,-2.16302e-1,8.57118e-2,1.08604e-
  2,8.34114e-1,9.62539e-3,2.23692e-3,-4.74381e-5,7.53115e-5,1.30994e-4,3.24603e-4,-2.24938e-5,-2.19998e-5,-2.45361e-4,-1.47715e-
  5,-9.08689e-9,-2.06332e-4,-1.95304e-5,2.04109e-4,1.43672e-5,1.16556e-4,-2.19910e-5,5.32611e-5,-1.24396e-4,4.91751e-5,1.57303e-
  5,-1.19677e-5,1.46863e-6,2.59065e-5,-2.45243e-6,-6.99479e-6,-7.06242e-7,-5.37997e-6,-4.74823e-6,-8.81991e-6,1.80509e-5,-
  1.40011e-6,-1.58826e-8,-4.22684e-5,9.23331e-8,9.57167e-8,-1.13211e-7,-4.15445e-8,4.49414e-8,-7.21016e-8,6.80210e-8,-1.50407e-
  10,7.05412e-9,2.63589e-8,-2.58317e-8,-3.12451e-10,-2.14054e-10,3.38255e-2,-1.40932e-2,3.49559e-2/)
T(:,7)=(/2.83901e-3,-2.78685e-3,4.70510e-2,1.69568e-1,-1.40330e-1,-2.38418e-1,1.17163e-1,-2.05155e-1,8.59748e-1,3.57021e-2,-
  3.07505e-1,3.98084e-2,8.22461e-3,1.64045e-4,-5.88293e-5,-4.67777e-4,2.76883e-4,-2.79294e-4,-1.97903e-4,1.56477e-4,-4.61698e-5,-
  3.09144e-8,-5.04163e-7,-1.01233e-4,7.47818e-5,-8.95268e-5,1.29619e-4,1.06141e-4,6.65682e-5,-1.55171e-4,1.18560e-4,-5.81720e-5,-
  1.37581e-4,2.54897e-5,2.44729e-4,-2.90304e-5,-1.26640e-4,-2.58326e-7,-6.70369e-6,2.22779e-6,-1.25366e-6,-9.29524e-6,1.94071e-
  6,-4.49835e-8,2.42687e-5,2.48142e-7,-1.92268e-8,-7.96276e-7,-5.67954e-8,3.87077e-7,-6.37157e-7,2.31183e-7,-1.04717e-
  10,1.09104e-8,8.39190e-8,-9.02702e-8,3.30478e-9,2.26403e-9,8.60640e-4,-3.33910e-3,-3.31087e-6/)
T(:,8)=(/2.08273e-3,-1.51031e-2,7.25157e-2,1.35581e-1,-7.82324e-2,-2.86808e-1,8.83749e-1,-5.23021e-2,-2.96857e-1,6.41324e-2,-
  1.06561e-1,9.94000e-4,5.29258e-3,1.48596e-5,5.17069e-5,-2.99650e-3,5.46913e-4,1.00168e-4,-9.80721e-5,1.74987e-4,3.69901e-5,-
  1.56709e-8,-5.53710e-5,-7.77740e-6,4.97689e-5,-6.31136e-4,9.38699e-4,4.48607e-4,1.83267e-5,-1.22063e-4,-1.86329e-5,-2.79434e-
  4,4.51965e-5,-7.99708e-6,-6.78282e-5,-1.22336e-6,4.87116e-5,-2.59001e-5,2.35733e-5,6.35230e-5,1.23826e-4,-3.77307e-6,5.52084e-
  6,-2.55504e-7,9.51439e-6,-9.32868e-8,5.16910e-8,-8.05850e-7,1.62464e-7,4.11785e-7,-4.19356e-7,-4.34387e-8,-2.65438e-10,-
  1.78505e-9,2.74734e-8,-4.73034e-8,1.15555e-8,7.91644e-9,-1.48747e-2,-3.23537e-3,-1.16771e-2/)
T(:,9)=(/-3.69485e-2,-3.26993e-3,1.31647e-1,8.04526e-2,-1.52541e-1,-1.97918e-1,-2.46256e-1,-1.51070e-1,-2.26516e-1,5.03355e-1,-
  1.89270e-1,6.97756e-1,3.91726e-2,5.31790e-4,4.13736e-5,8.97649e-4,4.13002e-4,7.97003e-5,-4.81456e-4,1.29390e-4,-1.13377e-3,-
  1.50114e-7,4.00314e-4,-3.94509e-5,1.99379e-5,1.62482e-4,-5.51007e-4,-7.07131e-5,1.30932e-5,-6.58401e-5,-1.34962e-4,5.68238e-
  5,3.64457e-5,-6.51048e-6,-6.14695e-5,9.65287e-6,4.03392e-5,-6.42419e-5,-1.81219e-5,-1.29045e-5,1.27628e-4,-4.03160e-6,8.47587e-
  7,-1.80063e-7,1.19917e-5,-1.60233e-7,1.37289e-8,-5.98252e-6,1.28582e-6,5.54469e-6,-1.97804e-5,3.16669e-7,-1.35256e-9,2.11075e-
  8,4.11726e-7,-4.37216e-7,1.26342e-8,8.65541e-9,1.90687e-3,3.95297e-5,-3.46135e-3/)
T(:,10)=(/7.02685e-3,5.04295e-4,-2.09962e-2,-6.48353e-4,2.40041e-2,1.57094e-2,7.42477e-2,1.86184e-2,1.59006e-2,-7.80243e-
  1,2.30198e-2,6.19020e-1,-7.59475e-3,-1.91717e-4,1.25300e-4,-2.55796e-4,1.50873e-5,-4.09272e-6,8.26366e-4,-7.40834e-6,-1.01782e-
  3,2.81641e-8,-3.38176e-4,1.40071e-5,2.22267e-4,-1.01209e-4,3.07650e-4,3.33538e-5,7.14499e-6,2.58716e-5,1.73693e-4,3.77767e-6,-
  1.20345e-6,1.04322e-6,3.42081e-6,-1.75769e-6,-1.03109e-6,1.09779e-4,1.69670e-5,1.67937e-5,-2.18230e-4,5.21333e-8,1.40746e-
  7,1.31130e-7,-2.09219e-6,-7.32156e-8,-1.73469e-8,-5.58030e-6,1.18354e-6,4.98425e-6,-1.74955e-5,3.23428e-7,-8.99290e-
  11,2.29428e-8,-7.84693e-8,8.15631e-8,-1.35672e-9,-9.29458e-10,3.14147e-3,1.26543e-3,-2.67175e-3/)
T(:,11)=(/4.26078e-3,-5.38221e-3,2.27284e-2,-1.52724e-2,-7.68764e-3,-1.08695e-2,-1.61023e-4,6.33021e-3,-1.63615e-2,-1.56284e-2,-
  4.37333e-2,-1.13108e-2,8.02401e-2,1.99787e-3,2.37247e-4,8.83521e-4,7.32990e-4,2.27704e-5,3.32029e-5,2.85949e-4,6.16452e-5,-
  3.27558e-7,1.40452e-3,-2.28459e-5,-3.11075e-4,-1.91623e-5,-1.53970e-3,1.07434e-4,1.12954e-4,-5.42461e-5,-2.03341e-4,-4.05674e-
  6,8.81483e-6,-7.34793e-7,-8.30799e-6,2.98887e-6,1.74700e-5,1.66702e-5,9.11914e-6,8.80098e-6,1.42017e-5,-8.08645e-6,3.35019e-7,-
  9.11241e-7,5.09747e-5,-5.85258e-8,-1.20815e-7,4.08375e-6,-2.51045e-6,-1.26174e-6,4.20552e-6,4.98782e-8,4.64086e-11,2.48570e-
  9,9.01246e-7,-9.60516e-7,2.92994e-8,2.00724e-8,2.12583e-2,-2.76750e-2,9.94368e-1/)
T(:,12)=(/-5.59230e-3,1.15162e-2,1.92748e-1,-4.44531e-2,-8.08117e-2,-1.52373e-1,-1.11999e-1,-9.62379e-2,-9.82552e-2,-1.57655e-
  1,-1.06889e-1,-1.51556e-1,9.12142e-1,-4.93725e-4,1.48488e-4,1.85500e-4,8.97896e-5,3.92808e-5,1.64453e-4,-9.64003e-6,2.36307e-
  4,-3.63630e-6,-2.91432e-4,-2.03530e-5,-2.54354e-5,-2.23601e-5,4.01581e-4,5.66199e-5,-1.59798e-5,-2.02302e-5,3.06660e-
  5,1.67009e-5,1.57453e-5,-2.12062e-6,-1.24073e-5,1.36392e-6,1.79343e-5,4.74106e-6,5.31210e-6,1.87554e-5,-2.06559e-5,-2.40795e-
  6,-5.42158e-7,2.94429e-7,1.80505e-7,8.57023e-9,5.58181e-8,-3.26270e-7,4.07154e-7,-7.94847e-7,4.18066e-6,-1.00358e-7,2.44532e-
  10,-6.99688e-9,9.98190e-6,-1.05695e-5,2.86079e-7,1.95987e-7,1.18827e-3,2.75444e-3,-9.07694e-2/)
T(:,13)=(/1.59963e-1,3.18412e-2,7.34024e-1,-5.64299e-1,5.37146e-2,-2.11837e-1,-2.73156e-2,7.62265e-3,2.21454e-2,-6.87099e-2,-
  3.37024e-2,-6.01151e-2,-2.39780e-1,-3.12832e-3,7.52369e-4,-9.00201e-4,-1.31972e-4,5.68388e-5,-2.78550e-5,-9.31471e-5,7.84535e-
  5,9.69757e-7,-1.33570e-3,-1.45728e-4,-7.99475e-4,-4.16428e-4,2.83002e-3,5.69326e-4,-7.04424e-5,1.59156e-4,8.25506e-6,-6.55241e-
  5,8.35106e-6,4.54952e-6,8.36403e-5,-9.61106e-6,3.54309e-5,-8.66490e-5,2.50912e-5,1.00914e-4,1.31774e-4,-7.83686e-6,-5.14274e-
  6,9.66205e-7,2.20936e-5,1.51128e-7,3.12748e-7,-4.71567e-6,9.85319e-7,6.62369e-7,5.27197e-6,5.55725e-8,4.87837e-10,1.18837e-9,-
  2.69577e-6,2.81821e-6,-5.64376e-8,-3.86642e-8,3.10543e-4,1.30914e-3,-1.14009e-2/)
T(:,14)=(/7.60263e-2,-9.93496e-1,1.01675e-2,-2.27446e-2,1.20575e-2,-2.63058e-3,-1.24463e-2,-7.72654e-3,4.14576e-3,-1.37294e-
  3,7.28252e-3,-3.86683e-4,8.42832e-3,5.90113e-2,-1.04734e-2,8.23117e-3,-1.75885e-2,-1.54207e-4,-1.05836e-3,-4.33020e-3,-
  1.85664e-3,-1.75173e-7,2.99439e-2,-7.01093e-3,5.65057e-3,-8.38309e-4,-3.11300e-2,-5.99438e-5,-8.62071e-4,-7.98931e-3,-1.36517e-
  3,-1.19124e-3,2.87117e-6,1.05724e-5,-5.03421e-5,-3.44846e-5,-1.93520e-4,8.45317e-4,-1.23600e-4,3.81785e-5,6.52205e-4,8.35663e-
  5,-4.46266e-5,-4.39965e-6,-2.36232e-4,-4.50301e-7,-2.79685e-7,7.00353e-5,-1.28948e-5,-2.29003e-5,-8.66567e-6,-7.92230e-
  7,3.40872e-9,-5.46509e-8,7.10355e-7,-4.75958e-7,-1.39011e-7,-9.52336e-8,-3.35570e-5,-7.36444e-4,-6.70927e-3/)
T(:,15)=(/-5.63864e-1,-5.07727e-2,8.92660e-2,2.31287e-1,-2.09318e-1,-2.61228e-1,-2.55681e-1,-2.54057e-1,-2.51348e-1,-2.79502e-
  1,-2.71991e-1,-2.76433e-1,-2.83974e-1,-1.81519e-2,2.73782e-5,-1.84661e-2,-3.73896e-2,-5.13045e-4,-1.42179e-2,5.93065e-
  3,5.38434e-3,1.11996e-6,-1.05636e-2,-6.48818e-4,-3.83354e-4,6.18565e-3,2.12249e-2,-8.19108e-3,-5.51071e-3,9.14009e-3,-1.14607e-
  2,-7.11304e-3,-4.75900e-4,-1.74476e-4,-1.35648e-3,1.19083e-4,3.41254e-4,-4.62661e-3,6.37996e-3,7.00593e-3,1.73134e-2,8.12122e-
  5,1.15590e-5,4.19161e-6,-3.76246e-4,-1.38896e-6,-8.83146e-6,-4.64935e-5,2.61182e-5,2.34844e-5,-9.43155e-5,-7.70102e-7,-
  1.48834e-7,-2.44224e-7,-2.45282e-6,3.23905e-6,-4.34461e-7,-2.97640e-7,-9.55542e-5,-3.96588e-6,1.62977e-4/)
T(:,16)=(/7.86311e-1,5.66377e-2,-1.21908e-1,2.16689e-1,-2.49552e-1,-1.77536e-1,-1.86323e-1,-1.88385e-1,-1.91061e-1,-1.55465e-1,-
  1.62763e-1,-1.57502e-1,-1.47017e-1,4.93561e-2,9.12716e-3,4.27101e-2,7.74916e-2,1.08613e-3,3.55615e-2,-1.82210e-2,-1.13079e-
  2,5.62569e-7,3.22419e-2,4.96354e-3,-1.75712e-2,-1.26402e-2,-5.15868e-2,1.63988e-2,6.93502e-3,-1.00321e-2,4.30273e-3,1.61314e-
  2,9.94770e-4,2.81719e-4,2.40142e-3,-2.17500e-4,-6.72877e-4,1.20354e-2,-1.35832e-2,-1.46161e-2,-3.97731e-2,-7.85771e-5,-
  1.63796e-4,-2.73853e-5,9.40577e-4,2.64087e-6,-9.01468e-6,1.39741e-4,-6.70611e-5,-7.34894e-5,2.62866e-4,-9.04434e-7,-7.30542e-
  8,-2.22834e-7,-2.32312e-6,1.79638e-6,2.99997e-7,2.05522e-7,2.54460e-4,5.00123e-5,-2.65113e-3/)
T(:,17)=(/8.58704e-2,-5.35613e-2,-1.50896e-2,1.12564e-2,-1.38204e-2,-4.81744e-3,-9.68079e-3,-6.88201e-3,-7.16526e-3,-2.61954e-
  3,-3.92269e-3,-3.23478e-3,-1.56477e-3,-4.32843e-1,-1.25868e-2,-4.22433e-1,-1.01404e-3,-1.02541e-3,2.45263e-2,-1.71719e-2,-
  1.21016e-2,2.35278e-6,-2.75387e-1,1.92871e-2,-1.08611e-1,-6.87388e-3,7.18520e-1,3.15690e-2,-6.27216e-3,-1.13398e-1,4.37128e-2,-
```

```
 2.50383e-2,8.29302e-4,7.16477e-4,8.32248e-4,-7.83340e-4,-2.64411e-3,2.25556e-2,-1.12825e-2,-6.72382e-3,-2.23895e-2,-1.51612e-
 4,-1.86174e-4,-3.44474e-6,-7.48706e-4,-7.19784e-6,-3.82888e-6,-3.10903e-4,1.28725e-4,6.69157e-5,7.01366e-5,-7.78359e-
 6,2.45229e-9,-5.40120e-7,-1.03003e-5,6.13416e-6,2.47013e-6,1.69223e-6,-1.73728e-4,-2.97129e-5,2.22511e-3/)
T(:,18)=(/7.13627e-2,-1.11179e-2,-1.14281e-2,7.68762e-3,-1.06305e-2,-3.57785e-3,-3.63933e-3,-6.07397e-3,-5.05044e-3,-1.28444e-
 3,-2.38964e-3,-1.71364e-3,-6.84375e-4,-4.25702e-1,-3.58880e-2,2.03150e-1,-6.81557e-1,4.35398e-4,5.62459e-2,-1.90782e-3,-
 1.94290e-2,5.10270e-6,-2.29233e-1,-5.21053e-3,8.95031e-2,-3.24162e-2,-1.58508e-1,6.29413e-2,-4.16959e-2,4.56531e-1,9.25807e-
 3,9.99380e-3,2.61665e-3,1.04489e-3,6.03721e-3,-6.55066e-4,-5.69453e-3,1.56584e-2,-7.93367e-3,-1.67682e-2,-2.82742e-2,8.54707e-
 4,-2.80648e-4,-1.84124e-4,-1.11339e-3,-5.41232e-6,-6.97981e-6,8.58093e-4,-1.56398e-4,-2.93264e-4,-3.57850e-5,-3.25195e-6,-
 3.29790e-8,-2.06195e-7,-1.44125e-5,1.44756e-5,1.08127e-7,7.40752e-8,-8.48765e-5,-1.41737e-5,1.07545e-3/)
T(:,19)=(/-2.84046e-2,-3.28842e-2,4.76714e-3,-1.90683e-3,2.42447e-3,1.77565e-4,1.63705e-3,9.88023e-4,6.86775e-4,-6.40163e-4,-
 5.19752e-4,-9.41946e-4,-9.42986e-4,-3.22953e-1,3.00207e-6,5.17552e-1,4.53485e-1,-1.12158e-3,1.78495e-2,-1.96016e-1,-5.08975e-
 2,1.73573e-6,-3.67317e-1,-1.21361e-1,3.53610e-1,8.53172e-2,3.10648e-2,-2.42959e-1,1.95111e-2,-6.43730e-2,-9.01082e-2,1.46195e-
 1,-1.27420e-3,-7.00810e-4,-5.01940e-3,3.50063e-3,1.34499e-3,1.59273e-2,-1.35450e-2,-1.87093e-2,-4.60338e-2,1.52379e-5,-
 1.23470e-3,-2.05660e-5,5.10404e-4,-5.82943e-7,-1.51596e-5,1.28076e-3,-2.31865e-4,-5.38371e-4,4.18786e-4,-1.75119e-5,-2.33838e-
 10,-1.43569e-6,4.49651e-6,6.06448e-6,-6.03923e-6,-4.13735e-6,-2.53114e-5,-5.20148e-6,4.57648e-4/)
T(:,20)=(/-1.84714e-2,-1.33422e-2,2.83988e-3,-2.98620e-3,3.74424e-3,2.03865e-3,7.14399e-4,2.61196e-3,2.44952e-3,1.31485e-
 3,1.60003e-3,1.45367e-3,1.37173e-3,-1.64580e-1,1.99920e-2,-4.13122e-1,3.73226e-1,-1.12186e-4,-7.43283e-2,-5.10342e-1,-3.12770e-
 2,-3.50207e-7,3.26006e-2,-2.68304e-2,7.20724e-3,-7.19928e-2,-2.60925e-1,1.91771e-1,6.48944e-2,5.04774e-1,-8.83062e-2,-1.16372e-
 1,-2.97406e-3,8.52014e-4,-4.76280e-3,-4.11144e-3,1.00143e-2,-1.86266e-2,1.02158e-2,2.74998e-2,3.64149e-2,-2.65515e-3,-1.11612e-
 3,-1.06964e-4,1.00766e-2,-6.73489e-6,-1.02274e-7,2.73547e-4,-2.41828e-4,-1.90605e-4,1.18886e-3,-1.14807e-5,3.39372e-8,-
 1.24647e-6,-7.42833e-6,-1.71126e-6,5.30494e-6,3.63430e-6,3.70320e-6,-1.30855e-6,6.68074e-5/)
T(:,21)=(/2.28764e-2,2.36959e-2,-3.66949e-3,2.79349e-3,-3.78730e-3,-1.32659e-3,-1.13817e-3,-2.00447e-3,-1.93253e-3,-6.56559e-4,-
 1.32372e-3,-4.73427e-4,-6.09365e-4,2.33493e-1,8.74189e-2,1.46821e-2,-3.68219e-1,3.53533e-4,-6.66712e-2,-7.58913e-1,1.25998e-
 1,5.67291e-6,3.40414e-3,5.57759e-2,2.03780e-1,4.29978e-2,1.25183e-1,-1.06103e-1,-8.63338e-2,-2.96605e-1,7.74855e-2,-2.38514e-
 2,7.25321e-4,-4.98886e-4,5.63611e-3,1.87219e-3,-6.96834e-3,-4.66545e-2,4.71719e-2,5.06278e-2,1.48757e-1,-3.51440e-3,3.32574e-
 3,5.40179e-5,-9.18134e-5,-2.56310e-5,-2.90278e-5,-1.74346e-3,3.37452e-4,8.26453e-4,-1.30726e-3,2.73554e-5,-8.70328e-8,8.78729e-
 7,-1.25770e-5,1.58051e-5,-1.82845e-6,-1.25264e-6,-1.35381e-5,-8.77175e-8,3.24239e-4/)
T(:,22)=(/8.21783e-3,1.09190e-2,-7.98694e-4,3.12114e-3,-4.52673e-3,-3.05221e-3,-4.48517e-3,-3.17798e-3,-3.48618e-3,-2.64145e-3,-
 3.09922e-3,-3.24453e-3,-3.07669e-3,1.67011e-1,-7.71293e-2,-4.26432e-1,-3.66725e-2,-1.14722e-3,1.72211e-1,2.04972e-1,-5.25532e-
 2,3.62344e-6,-2.15044e-1,-1.16941e-1,7.23378e-1,-2.86186e-2,-1.57154e-1,7.63417e-3,-1.54451e-2,-6.66654e-2,2.58548e-1,-
 1.60846e-2,5.20048e-3,-1.98405e-3,1.97355e-2,-3.44757e-3,-2.21159e-2,-1.88296e-3,-3.29429e-2,-4.67038e-2,-1.52879e-1,1.86569e-
 3,2.26597e-3,6.55653e-5,-9.88206e-3,2.57028e-5,-5.37789e-5,1.18122e-3,-1.26326e-4,-4.27560e-4,-1.18859e-4,-2.94173e-5,1.77037e-
 8,-1.58536e-6,-8.18078e-6,1.34293e-5,-3.14912e-6,-2.15739e-6,-9.74737e-6,-1.26921e-5,3.82154e-4/)
T(:,23)=(/-7.01906e-2,-6.33337e-3,1.10207e-2,-6.81879e-3,9.94988e-3,3.01805e-3,4.45184e-3,4.24149e-3,4.41571e-3,1.74838e-
 3,1.79807e-3,1.70800e-3,4.64798e-4,-4.89787e-2,-8.08995e-2,1.35681e-1,8.38949e-2,-8.76207e-3,3.87173e-1,-2.14696e-1,1.90000e-
 1,-9.70180e-6,8.73326e-2,1.47019e-2,-2.36466e-1,-2.15707e-2,-2.38584e-2,1.49372e-1,-1.70963e-2,-1.41174e-2,5.74220e-1,8.61446e-
 2,7.10222e-4,-5.90390e-4,1.15214e-2,-3.64881e-3,-2.42899e-2,1.02899e-1,-1.45532e-1,-1.52551e-1,-4.96793e-1,2.73239e-3,7.63840e-
 3,8.01128e-5,-2.65688e-2,-5.85832e-5,-3.19302e-5,-2.09154e-4,3.59708e-4,8.48403e-4,-5.01466e-3,2.41031e-5,-2.66694e-7,1.03995e-
 6,1.70640e-5,-3.07462e-5,7.84045e-6,5.37132e-6,6.78168e-6,1.54709e-6,5.51084e-6/)
T(:,24)=(/-1.17419e-2,4.96301e-3,1.34012e-3,7.87407e-5,5.28101e-4,-1.65527e-4,-2.14120e-4,-6.25108e-5,-2.35578e-4,-7.76429e-4,-
 5.58571e-4,-5.05874e-4,-8.37357e-4,-5.25550e-2,-2.68868e-1,1.83894e-1,3.45885e-3,1.88330e-4,-5.86031e-2,-2.68441e-2,7.91156e-
 2,-7.26932e-6,5.54011e-2,-3.11788e-1,1.31499e-1,-3.58452e-1,6.98356e-2,7.34398e-1,1.26206e-2,-2.26977e-1,-1.30967e-1,5.75945e-
 2,-2.70414e-3,-2.07244e-3,1.67014e-3,-6.66429e-4,3.53608e-3,-3.94658e-2,6.16645e-2,-1.58312e-2,1.11677e-1,5.21046e-5,-1.15742e-
 3,1.85712e-4,-9.43532e-4,4.48988e-6,-3.11319e-5,-3.61210e-4,-3.27225e-4,4.55219e-4,-3.58131e-4,7.56344e-6,-6.04683e-8,7.82658e-
 7,-7.22266e-6,-2.27106e-5,1.70080e-5,1.16518e-5,2.42730e-6,-5.92477e-6,1.58188e-5/)
T(:,25)=(/1.60793e-3,-1.01461e-3,-9.40216e-5,-1.23433e-3,9.89052e-4,1.17436e-3,9.05755e-4,1.26248e-3,1.27530e-3,1.44438e-
 3,1.24854e-3,2.47826e-3,1.25748e-3,1.16319e-1,2.60131e-2,-9.10285e-2,-1.46725e-2,1.58359e-2,2.06307e-1,4.06921e-2,7.74980e-
 1,9.30967e-6,-1.45380e-1,8.69977e-2,3.51467e-2,-3.24126e-2,1.22960e-2,-5.05817e-2,1.56578e-1,7.38604e-2,-4.66132e-1,1.39570e-
 1,8.16692e-3,2.60554e-3,-6.57462e-3,2.75310e-3,2.35304e-2,5.24259e-2,-1.17193e-2,-8.54622e-2,-1.42152e-1,-3.01857e-3,-8.98599e-
 3,7.62246e-5,2.35283e-2,8.12952e-6,-4.22807e-6,-3.86395e-3,-6.54847e-4,2.96390e-3,-5.67471e-3,3.10914e-4,-6.35483e-7,2.09094e-
 5,-6.01387e-6,3.01840e-5,-1.37011e-5,-9.38632e-6,6.73625e-6,3.90724e-7,-7.26333e-5/)
T(:,26)=(/4.23802e-2,-1.40801e-3,-6.52852e-3,6.16465e-3,-8.06771e-3,-4.18069e-3,-4.63459e-3,-4.82717e-3,-5.01263e-3,-3.36473e-
 3,-3.29635e-3,-2.20514e-3,-2.47206e-3,-2.64942e-1,-3.17349e-1,-1.23723e-2,6.49127e-2,4.25885e-3,-2.79511e-1,1.06416e-
 1,5.20879e-1,-1.17885e-5,1.97731e-1,-2.38985e-1,4.02094e-2,1.87826e-1,-7.98171e-2,-1.92056e-1,-1.29486e-1,-1.00126e-2,3.37591e-
 1,-2.58797e-1,-2.67806e-4,-3.08748e-3,4.28496e-2,-5.76188e-3,-1.76834e-2,-5.65574e-2,2.85028e-2,1.30546e-1,2.53354e-1,3.03660e-
 3,6.30950e-3,-2.52321e-4,-1.36691e-2,-5.64999e-5,-7.57751e-6,1.80948e-4,-1.31533e-3,1.67598e-3,-5.71654e-3,1.59613e-4,-
 8.37131e-8,1.20465e-5,5.25832e-6,-3.21663e-5,1.52168e-5,1.04247e-5,-8.37494e-7,-7.21085e-6,9.60545e-5/)
T(:,27)=(/4.96028e-3,-5.35692e-3,-8.72823e-4,9.24368e-4,-1.04378e-3,-5.17313e-4,-1.02614e-3,-8.09156e-4,-8.18476e-4,-9.43756e-
 4,-5.49959e-4,-3.09718e-4,-4.49446e-4,1.54470e-1,2.30670e-1,-5.64872e-3,7.06421e-2,-4.43919e-2,-5.44660e-1,7.37208e-2,1.70687e-
 1,4.24356e-6,-2.72763e-1,1.28374e-1,-4.51470e-2,-3.32680e-1,1.03731e-2,3.18942e-2,-3.05709e-1,1.46596e-1,2.78936e-1,3.98355e-
 1,-2.86057e-2,-6.50884e-3,-8.85716e-2,2.01809e-2,-6.87628e-4,8.52385e-2,-7.46931e-2,-6.99714e-2,2.58848e-2,5.75151e-3,5.77569e-
 3,-1.22386e-4,-2.95341e-2,-1.51713e-4,-1.72624e-6,2.53671e-3,-2.41728e-4,-1.06626e-4,-5.03039e-3,-2.29062e-5,1.76210e-7,-
 3.73183e-7,1.63659e-5,1.37083e-5,-1.74692e-5,-1.19678e-5,2.05423e-6,2.71751e-6,1.26472e-5/)
T(:,28)=(/-1.35478e-3,4.39615e-3,4.76105e-4,-4.67575e-4,3.61721e-4,5.50348e-5,9.88420e-5,1.90572e-4,3.06718e-4,2.69955e-
 4,1.86851e-4,9.31581e-5,1.70080e-4,-9.20533e-2,-5.26125e-1,-1.79290e-1,3.25657e-3,5.96245e-2,2.24968e-1,-6.84896e-2,-1.11598e-
 1,-1.40037e-5,8.37686e-2,1.20777e-1,-9.59292e-2,-7.72518e-2,-1.35723e-1,-2.14775e-1,-2.74414e-1,-5.96680e-2,-7.66359e-
 2,5.61870e-1,2.09000e-2,-1.77592e-3,8.29223e-2,1.32613e-2,1.65168e-2,-3.54260e-2,1.76971e-1,-1.40803e-1,2.21983e-1,-3.09685e-
 3,3.91091e-3,3.82310e-4,1.94304e-3,1.65340e-4,3.31870e-5,-1.77033e-3,9.14008e-4,1.22255e-4,4.11979e-4,8.15148e-5,-5.36766e-
 8,4.06262e-6,5.51816e-5,-3.61237e-5,-1.08369e-5,-7.42416e-6,-1.16796e-5,-1.44038e-6,1.81556e-4/)
T(:,29)=(/1.18114e-3,1.19713e-2,-2.57166e-5,5.25740e-6,-3.43391e-4,-4.96764e-5,3.25157e-4,-2.02712e-4,-1.41734e-4,6.02714e-6,-
 9.71098e-5,-1.65672e-4,-1.63611e-4,4.73396e-1,-4.41809e-1,2.16424e-1,7.55792e-2,4.90352e-2,5.64270e-2,-9.40655e-3,-3.76124e-2,-
 4.82818e-6,-1.23378e-1,8.95732e-2,6.58059e-2,-9.30867e-5,4.34501e-1,-1.14548e-2,-7.09391e-2,4.95934e-1,5.77934e-2,-1.99725e-
 1,1.32849e-2,-1.42796e-4,4.16787e-2,4.05335e-3,3.75778e-2,-4.46145e-2,4.02701e-2,3.10767e-2,4.28140e-2,-4.08150e-3,-4.43654e-
 3,-3.42088e-4,1.97993e-2,2.07962e-4,1.62278e-5,-2.83239e-3,8.50713e-5,6.60226e-4,3.38215e-3,5.78937e-5,1.98322e-7,3.95096e-6,-
 2.84675e-5,-1.08128e-5,2.21860e-5,1.51992e-5,1.87602e-5,1.95215e-6,-1.13641e-4/)
T(:,30)=(/1.58346e-3,3.24280e-3,1.58734e-4,6.67011e-4,-1.05840e-3,-9.94193e-4,-7.38111e-4,-8.57415e-4,-9.65117e-4,-1.27038e-3,-
 8.67968e-4,-8.78316e-4,-8.24379e-4,-5.54554e-2,-4.58767e-1,-3.40481e-2,-5.74134e-2,-9.13226e-3,-4.79185e-1,-5.52746e-2,-
 7.16853e-2,-1.39290e-5,-1.00333e-1,3.98220e-1,9.59174e-3,1.49333e-1,-1.40251e-1,5.12980e-2,2.56653e-1,-1.98933e-1,-9.85228e-2,-
 1.21922e-1,-4.02899e-2,-3.45925e-3,-9.32607e-2,2.83127e-3,9.39206e-2,9.80288e-2,-7.07061e-2,-2.08537e-1,-3.51227e-1,3.77967e-
 4,-6.60075e-3,-2.47286e-4,8.06129e-3,-4.32267e-5,-4.36236e-5,-1.53008e-4,-1.01384e-4,4.94400e-5,8.64504e-4,-1.92469e-
 5,5.26448e-8,-8.53152e-7,-1.18673e-6,-4.02372e-5,2.38950e-5,1.63700e-5,-1.59658e-5,-4.25852e-6,1.93164e-4/)
T(:,31)=(/2.82989e-3,9.21353e-3,-6.10150e-4,-5.53033e-5,-2.85624e-5,3.21744e-4,5.00654e-4,2.76637e-4,2.64422e-4,2.01630e-
 4,4.04960e-4,3.41809e-4,4.31843e-4,-2.25372e-2,4.93895e-2,-1.50147e-2,-8.30468e-2,6.91219e-3,-2.42219e-1,-2.71031e-2,-5.54457e-
 2,7.89630e-6,5.50641e-1,-3.33324e-1,2.34722e-1,1.83522e-1,2.86588e-1,-8.66117e-2,-2.17900e-2,2.10823e-1,-1.43898e-1,3.32691e-
 1,-3.35472e-2,-1.40159e-2,-4.25485e-2,1.60778e-2,7.41945e-2,9.09454e-3,-8.07590e-2,-1.49321e-1,-3.44735e-1,-1.25558e-2,-
 4.66322e-4,1.29006e-3,3.65273e-2,1.10020e-4,-1.83468e-5,-1.01806e-2,2.39332e-3,2.20183e-3,5.61889e-3,-4.30060e-5,3.10073e-7,-
 1.04004e-6,-6.83720e-6,2.86731e-5,-1.23639e-5,-8.47024e-6,1.58871e-5,-8.70709e-8,-9.22688e-5/)
T(:,32)=(/8.37833e-4,7.13169e-3,-9.11299e-5,-3.75463e-4,3.16489e-4,3.95315e-4,6.43857e-4,4.25483e-4,3.12356e-4,4.14349e-
 4,4.18710e-4,4.11259e-4,4.31162e-4,1.97599e-1,-1.13762e-2,-7.92262e-2,-4.17024e-2,5.45782e-2,-4.89337e-2,-4.09048e-2,-4.79061e-
 2,4.59211e-5,-3.36601e-1,-4.49089e-1,-2.34283e-1,4.87305e-1,-9.26800e-2,1.39296e-1,-1.10201e-1,-1.42715e-2,-5.07947e-
 2,3.24955e-2,-1.42090e-2,-1.84087e-2,1.61437e-1,-3.44711e-2,2.74532e-2,4.06256e-1,-3.04162e-2,-2.89857e-1,1.13101e-1,-1.41971e-
 2,1.66925e-2,5.85124e-4,1.65466e-2,1.63360e-4,6.21103e-5,-3.69214e-3,6.59426e-4,8.94641e-4,3.14924e-3,-5.38941e-5,2.21001e-7,-
 7.46004e-7,-1.92895e-4,1.22447e-4,4.15218e-5,2.84457e-5,-2.48807e-6,8.56924e-7,-3.83783e-6/)
T(:,33)=(/4.63330e-3,7.66346e-3,-8.18208e-4,7.74368e-4,-8.94455e-4,-4.34996e-4,-5.41058e-4,-3.95846e-4,-6.03204e-4,-3.28532e-4,-
 3.59240e-4,-3.02415e-4,-2.20829e-4,1.35891e-1,-1.54417e-1,-8.62460e-2,-9.76023e-2,6.35587e-2,-1.22830e-1,-6.14884e-2,-6.32305e-
 2,5.50203e-5,-2.56499e-1,-4.65628e-1,-2.72628e-1,-2.31676e-1,-8.12203e-2,-2.61240e-1,3.41214e-1,-3.52287e-2,1.92830e-
 2,1.21180e-1,-2.42356e-2,-2.50852e-2,1.15894e-1,3.16920e-2,4.93024e-2,-3.56641e-1,-4.47339e-2,3.02013e-1,-2.36494e-1,-6.17736e-
 3,2.13428e-2,-2.13652e-4,-2.54367e-2,1.34459e-4,3.24510e-5,-1.91546e-3,1.33719e-3,4.80370e-4,-1.14143e-3,-5.05394e-5,1.58461e-
 7,-1.27154e-6,-1.90518e-4,1.52012e-4,2.38252e-5,1.63221e-5,-9.69251e-6,-7.45258e-7,9.99287e-5/)
T(:,34)=(/-7.58220e-3,4.70096e-3,1.15923e-3,-9.53037e-4,1.23103e-3,5.75109e-4,5.83389e-4,7.36954e-4,7.78110e-4,4.88808e-
 4,4.37794e-4,3.36163e-4,2.71595e-4,-2.52631e-2,-1.15116e-1,-1.17672e-2,-2.66016e-2,1.33064e-1,3.46015e-2,-3.21769e-2,-4.94599e-
```

```
2,-1.60908e-5,1.14785e-1,-2.52665e-2,4.68732e-2,-3.02316e-1,6.07475e-3,-1.58493e-1,2.58578e-2,-2.97494e-2,-6.54740e-2,-
  3.59583e-2,8.65505e-3,-1.66953e-2,5.52864e-2,5.71852e-2,1.46574e-1,7.21407e-1,-3.30476e-1,4.04636e-1,2.89572e-2,-9.37423e-
  3,7.85105e-4,1.03009e-3,2.39813e-2,4.72189e-4,-2.70746e-4,-6.81452e-3,2.02363e-3,1.45287e-3,1.90105e-3,1.86857e-4,-5.68795e-
  7,8.73537e-6,6.78161e-5,-3.72913e-5,-1.76970e-5,-1.21238e-5,7.50926e-7,-8.20438e-7,3.87042e-6/)
T(:,35)=(/-2.15435e-3,-7.85880e-4,3.11456e-4,-1.22994e-3,1.34085e-3,1.15089e-3,1.15526e-3,1.05641e-3,1.32865e-3,1.09275e-
  3,1.11498e-3,1.08625e-3,1.05193e-3,-1.64632e-2,-1.45810e-2,-2.91117e-2,2.38254e-2,1.73053e-1,2.59272e-2,-1.41301e-3,-1.38365e-
  3,-7.50734e-6,-9.18830e-2,1.32573e-2,-4.91712e-2,1.33446e-1,-8.74317e-2,1.06352e-1,-6.77190e-1,-9.60101e-2,-2.27511e-1,-
  1.45038e-1,2.40813e-2,4.99809e-3,-5.41640e-2,2.69568e-2,2.81399e-1,-2.49806e-1,-2.10990e-1,2.87484e-1,-3.05992e-1,-1.55061e-2,-
  2.53006e-2,6.84868e-4,1.07271e-1,5.78806e-4,-3.77263e-5,7.04506e-4,-1.35747e-3,-8.03751e-4,4.33621e-3,4.83850e-4,-1.15775e-
  7,2.67229e-5,7.27928e-5,-2.00329e-5,-3.06758e-5,-2.10153e-5,-5.45458e-6,-9.50722e-7,4.31317e-5/)
T(:,36)=(/-2.77290e-3,1.49789e-3,3.02244e-4,-4.20641e-4,6.21721e-4,4.20214e-4,2.60308e-4,4.37647e-4,5.98702e-4,3.60834e-
  4,3.33863e-4,3.05840e-4,2.89721e-4,-2.89286e-2,5.82180e-2,8.01159e-3,-1.19397e-2,1.99585e-1,2.00162e-2,-1.70366e-2,-2.07242e-
  2,4.43103e-5,2.76594e-2,-1.92104e-1,-4.63441e-2,-4.30202e-1,-1.71760e-2,-2.73843e-1,-1.25272e-1,-4.21904e-2,-3.85609e-2,-
  3.70632e-1,2.13572e-2,1.15782e-3,-1.47189e-1,1.10948e-1,3.25277e-1,4.80896e-2,2.21642e-1,-5.50988e-1,-4.63709e-2,-8.07994e-
  3,1.18344e-2,-1.10362e-3,2.18132e-2,6.35507e-4,-2.68577e-5,-3.42876e-4,1.09131e-4,-3.76356e-4,1.12860e-3,4.58053e-4,-1.14192e-
  7,2.10020e-5,-1.02025e-4,1.20551e-4,-9.89374e-6,-6.77799e-6,-1.96911e-6,-3.81908e-7,3.76744e-6/)
T(:,37)=(/-5.78948e-3,-3.04408e-3,8.07895e-4,-6.31146e-4,9.44008e-4,4.47944e-4,3.90722e-4,4.72164e-4,2.62640e-4,1.34471e-
  4,3.07361e-4,2.47859e-4,1.84653e-4,-4.96004e-2,8.16299e-2,3.07074e-2,1.85452e-2,1.16949e-1,-9.61080e-2,1.01229e-2,6.65433e-3,-
  2.46544e-5,6.42283e-2,1.28765e-1,5.34550e-2,-1.37085e-1,1.46956e-2,-1.39962e-2,-6.94223e-2,-1.72283e-2,-4.74421e-2,-9.60959e-
  2,-1.20704e-1,-1.05841e-1,8.99874e-1,-6.69172e-2,-1.14685e-1,-7.25023e-2,-8.45330e-2,-1.56837e-1,-7.33107e-2,1.07080e-2,-
  6.58872e-2,-3.54978e-4,8.42496e-2,5.25125e-5,1.70807e-5,2.90483e-3,1.30135e-4,-1.04474e-3,-9.52466e-4,-8.07130e-4,-4.39915e-8,-
  3.81543e-5,1.95376e-4,-6.99017e-5,-7.33217e-5,-5.02311e-5,-4.48998e-7,2.02578e-7,-7.88582e-6/)
T(:,38)=(/2.10246e-3,-3.65439e-3,-3.51314e-4,6.71426e-4,-7.30219e-4,-5.37944e-4,-5.60208e-4,-5.83327e-4,-4.01093e-4,-5.27160e-
  4,-5.55629e-4,-4.95682e-4,-4.70088e-4,-1.87006e-2,9.02330e-2,1.80831e-2,1.39090e-2,4.14922e-1,8.46848e-3,2.48923e-2,3.00823e-
  2,-3.05461e-5,2.05506e-2,1.18756e-1,5.76587e-2,1.45066e-1,2.35314e-2,1.22028e-1,1.77129e-1,2.96637e-2,1.03640e-1,1.29831e-
  1,7.82957e-2,1.86420e-2,1.19852e-1,1.03486e-2,5.13708e-1,-9.48770e-2,-4.51472e-2,1.90844e-2,1.38718e-1,7.65718e-2,1.58604e-1,-
  1.09098e-3,-5.96846e-1,9.05476e-4,-1.46180e-4,1.35446e-2,-2.95250e-3,-3.22714e-3,-8.87816e-3,7.37449e-4,5.70140e-7,1.87808e-
  5,9.48059e-5,-8.92599e-5,-4.33160e-6,-2.96749e-6,2.93906e-6,5.30426e-7,-1.12466e-5/)
T(:,39)=(/-1.56859e-3,-1.59520e-3,2.31274e-4,4.34138e-4,-3.50636e-4,-5.09202e-4,-4.74974e-4,-4.51983e-4,-3.35260e-4,-6.22022e-
  4,-5.16741e-4,-5.54930e-4,-5.59471e-4,-5.92178e-3,1.28578e-2,6.40926e-3,-3.89004e-3,2.49553e-1,3.77292e-2,6.53615e-3,4.42026e-
  3,-2.69224e-5,7.20319e-4,3.55392e-2,1.17748e-2,3.80750e-2,6.17888e-3,3.24222e-2,2.15352e-1,8.96175e-3,1.14067e-1,9.66946e-
  2,1.70901e-1,3.87988e-2,-8.91772e-2,3.86078e-2,1.62946e-1,-2.09001e-1,-5.36998e-1,-1.97967e-1,3.01140e-1,-2.76910e-2,-2.39311e-
  1,-5.55258e-4,5.24311e-1,1.03898e-3,9.58656e-4,2.02436e-2,8.55666e-4,-3.24996e-3,-3.49116e-2,7.79772e-4,2.86501e-7,3.98738e-5,-
  6.39413e-6,-8.36845e-5,5.21388e-5,3.57192e-5,9.59322e-7,2.54502e-7,-2.45954e-5/)
T(:,40)=(/2.83047e-3,-3.47055e-3,-4.65005e-4,6.34283e-4,-7.11717e-4,-4.72179e-4,-5.16195e-4,-5.24690e-4,-4.53258e-4,-3.56833e-
  4,-4.13065e-4,-3.98762e-4,-3.65418e-4,-1.26613e-2,9.51461e-2,1.37696e-2,1.24630e-2,2.39752e-1,-4.60411e-2,2.81854e-2,3.19187e-
  2,-5.24499e-5,-1.66853e-2,6.53641e-2,2.20031e-2,1.06002e-1,-1.50971e-4,9.71323e-2,1.13556e-1,1.23352e-2,1.48734e-1,1.15375e-
  1,3.28240e-2,-9.38110e-3,7.23130e-2,-7.85766e-3,2.72187e-1,1.43654e-1,6.36607e-1,2.52003e-1,-1.19896e-1,3.14108e-2,-2.89818e-
  1,-1.48143e-3,4.21083e-1,8.28350e-4,-8.48268e-4,6.07462e-2,-5.52267e-3,-1.21656e-2,-6.57749e-2,3.68417e-4,7.37787e-7,1.46277e-
  5,1.39850e-4,-1.53342e-4,5.80539e-6,3.97715e-6,3.08231e-6,4.94675e-7,-4.95300e-5/)
T(:,41)=(/-1.94579e-4,-5.52122e-4,4.13683e-5,-6.60342e-5,7.27907e-5,4.55949e-5,7.41110e-5,4.79716e-5,1.75182e-4,9.72191e-
  6,4.39497e-5,4.39807e-5,4.18981e-5,-2.49202e-3,1.09646e-2,9.16797e-5,5.27327e-3,3.57318e-1,-3.83775e-2,1.93046e-3,2.35459e-3,-
  1.81000e-5,-3.57821e-3,2.28534e-2,3.45341e-3,6.35328e-2,-2.85167e-3,4.49547e-2,-9.93711e-3,-3.42413e-4,-1.42453e-2,-2.43432e-
  3,1.94409e-1,-2.07417e-1,-2.50228e-2,7.84240e-1,-3.96866e-1,6.10251e-3,5.23605e-2,1.58178e-2,-4.64464e-2,-1.19796e-2,4.01832e-
  2,5.55802e-4,-4.73685e-2,1.41722e-3,-8.72955e-5,-9.27273e-3,-2.85508e-3,1.19979e-3,2.24123e-2,8.72014e-4,4.67122e-7,3.08632e-
  5,-2.30573e-5,-6.50176e-5,5.05233e-5,3.46125e-5,3.30584e-7,3.07550e-7,-8.91323e-6/)
T(:,42)=(/1.18740e-3,7.97806e-4,-1.63504e-4,6.12659e-5,-1.27298e-4,-2.66092e-5,-1.06622e-5,-3.24169e-5,-1.50526e-4,5.59275e-5,-
  1.51871e-5,1.99074e-5,2.74331e-5,9.56353e-3,-1.69299e-2,-7.19533e-3,-2.86765e-3,-3.10946e-1,5.69784e-2,-5.28788e-3,-1.58153e-
  3,2.08687e-5,-8.53764e-3,-2.31450e-2,-8.73726e-3,2.53161e-2,-1.96542e-3,-9.31979e-4,-6.59348e-3,3.68859e-3,-3.48653e-3,-
  2.14812e-3,-3.90832e-1,-6.01679e-2,1.00894e-2,3.57223e-1,1.96149e-1,-2.05585e-2,-7.83932e-2,-1.56108e-2,6.06174e-2,3.75590e-1,-
  6.19038e-1,-1.34270e-2,-1.92705e-1,-6.04380e-4,1.71507e-4,-3.79618e-2,1.53433e-3,8.88875e-3,4.51080e-2,-1.26438e-3,-6.39964e-
  7,-8.34921e-5,-2.33040e-4,5.51414e-5,1.03828e-4,7.11302e-5,-2.43738e-7,-7.33974e-8,1.77549e-5/)
T(:,43)=(/1.31967e-3,-1.43856e-3,-1.85523e-4,2.92581e-4,-3.57119e-4,-2.46005e-4,-2.60514e-4,-2.66308e-4,-3.89496e-4,-1.75571e-
  4,-2.00443e-4,-2.12720e-4,-1.94116e-4,-5.72715e-3,1.79745e-2,5.82456e-3,6.56640e-3,-2.96514e-1,5.48484e-2,1.39610e-2,1.25217e-
  2,-9.88224e-5,-4.93986e-3,4.32295e-2,1.45500e-2,4.98916e-2,1.90950e-3,4.13211e-2,5.44204e-2,7.55653e-3,5.05910e-2,3.47997e-2,-
  4.12726e-1,-4.01000e-2,8.62894e-2,3.87517e-1,3.21729e-1,-3.27520e-2,-7.13401e-3,1.91336e-2,8.07513e-2,-2.94492e-1,4.74163e-
  1,8.85168e-3,2.29409e-1,-1.29051e-3,6.92119e-4,1.96576e-1,-1.40312e-2,-3.83571e-2,-2.22540e-1,-1.41187e-3,-6.41376e-7,-
  2.38385e-5,1.67441e-4,-2.85104e-4,6.43513e-5,4.40857e-5,9.84751e-7,9.93513e-8,-1.66301e-5/)
T(:,44)=(/-4.16402e-4,1.30665e-3,6.60724e-5,-1.86824e-4,1.97029e-4,1.66652e-4,1.79378e-4,1.73238e-4,1.97954e-4,1.48112e-
  4,1.51406e-4,1.30382e-4,1.50072e-4,7.15842e-3,-2.06991e-2,-4.96885e-3,-4.91144e-3,5.04329e-2,-1.11268e-2,-9.69518e-3,-1.49346e-
  2,1.05546e-4,7.43057e-3,-2.96145e-2,-7.00326e-3,-2.24059e-2,3.37593e-3,-2.56967e-2,-4.12605e-2,-9.44974e-4,-4.45911e-2,-
  2.71650e-2,8.99320e-2,-1.52166e-2,-3.84462e-2,-8.66675e-2,-1.37991e-1,1.05087e-2,-4.08122e-2,-2.19887e-2,-2.81738e-2,1.07842e-
  1,-1.54105e-1,-2.42460e-3,-1.37774e-1,9.09390e-4,-9.32709e-5,5.58936e-1,-8.08995e-3,-1.05735e-1,-7.56620e-1,-1.14280e-3,-
  3.39318e-7,-1.00693e-4,-2.78138e-4,3.05301e-4,-1.29399e-5,-8.86485e-6,-3.95187e-7,-7.51952e-8,1.05952e-5/)
T(:,45)=(/3.43380e-4,-3.02109e-4,-5.38284e-5,5.13715e-5,-6.54391e-5,-3.35717e-5,-4.16541e-5,-4.12694e-5,-4.44783e-5,-2.63657e-
  5,-2.78185e-5,-6.33110e-5,-2.19751e-5,-1.94546e-3,4.42789e-3,1.19085e-3,2.25451e-3,-8.55256e-3,-1.03878e-3,2.53795e-3,-
  6.00274e-3,-4.96041e-5,-3.38544e-3,5.93763e-3,1.70089e-3,4.25856e-3,-2.11199e-3,5.64245e-3,-4.27308e-4,-9.97828e-5,2.64931e-3,-
  1.72587e-3,4.54481e-3,3.06875e-2,1.22516e-2,2.43662e-2,1.27873e-2,-2.56477e-3,1.20697e-2,4.23775e-3,-1.63808e-4,1.23397e-
  2,2.49565e-2,-3.72197e-3,1.06893e-2,2.13836e-4,-1.53406e-4,-6.99180e-1,3.76833e-1,2.18168e-1,-5.64213e-1,-2.78670e-3,1.09422e-
  5,-1.78983e-4,1.80109e-4,-1.37951e-4,-2.64652e-5,-1.81307e-5,-1.06907e-7,-1.37940e-7,4.92357e-6/)
T(:,46)=(/-9.52978e-4,3.22122e-4,1.37745e-4,-1.14951e-4,1.59573e-4,7.68451e-5,8.72496e-5,9.05034e-5,1.55012e-5,2.35516e-
  5,5.67441e-5,4.80756e-5,3.67717e-5,2.55692e-3,8.72475e-4,-1.39643e-3,-1.28258e-3,-1.20427e-1,-3.58225e-2,-2.42384e-3,-3.32062e-
  3,5.27706e-5,1.39749e-3,-1.12779e-2,-9.94111e-4,-4.36093e-3,-3.86585e-5,-2.08689e-3,-1.30307e-2,-2.59030e-3,-1.16532e-2,-
  4.91025e-3,2.18597e-1,9.17749e-1,1.66291e-1,2.45148e-1,2.13867e-3,1.85527e-2,2.15105e-2,2.65073e-4,-4.16079e-2,3.25910e-2,-
  1.12223e-2,3.31799e-3,-1.06929e-2,-1.19777e-2,-8.98640e-4,3.92772e-2,-1.36704e-2,-1.15429e-2,5.72876e-3,-4.56363e-4,1.72354e-
  6,3.94967e-5,-3.12592e-6,1.60303e-4,-8.90245e-5,-6.09888e-5,-7.98955e-8,-9.21351e-8,1.31871e-6/)
T(:,47)=(/-1.77169e-4,4.15664e-5,2.96695e-5,-2.85948e-5,3.34377e-5,1.85933e-5,1.94073e-5,2.13076e-5,2.85558e-5,1.21828e-
  5,8.55778e-6,1.31140e-5,1.12124e-5,1.27016e-3,-6.63564e-4,-7.84440e-5,-5.88658e-4,3.04700e-2,5.51771e-4,-2.11998e-3,-2.80866e-
  4,7.66270e-5,5.23626e-4,-7.86398e-5,6.04823e-4,1.59858e-3,1.72566e-3,1.16588e-3,3.74550e-3,3.89769e-4,-6.65096e-4,4.13465e-3,-
  2.14916e-2,-8.37998e-3,-9.78247e-3,-1.28368e-2,-1.35168e-2,-1.25066e-5,-4.73098e-3,1.12300e-3,7.23614e-4,8.55494e-1,4.36807e-
  1,-1.41843e-1,2.33701e-1,-6.98147e-3,3.37319e-3,1.88742e-2,-8.65742e-3,-5.89180e-3,9.65910e-3,3.99066e-4,-1.79362e-7,-3.32723e-
  5,-2.09016e-4,2.27469e-4,-9.80584e-6,-6.71777e-6,3.47233e-7,2.68343e-8,-5.05205e-6/)
T(:,48)=(/3.18033e-4,-1.10177e-4,-5.00231e-5,3.95114e-5,-5.31058e-5,-2.33066e-5,-2.88026e-5,-2.90688e-5,1.87901e-5,-2.64677e-6,-
  1.55609e-5,-1.36551e-5,-1.14644e-5,-4.68065e-4,-1.92768e-4,1.94651e-4,8.35458e-4,5.10674e-1,1.78237e-2,1.97921e-3,2.10358e-3,-
  1.98071e-4,6.23377e-5,3.67729e-3,1.94179e-3,4.29796e-3,5.24931e-4,2.62157e-3,2.92748e-3,1.44097e-3,2.04476e-3,2.74127e-3,-
  7.24245e-1,3.04722e-1,-1.68697e-1,-1.14944e-1,-2.74688e-1,-7.12829e-3,-1.32319e-2,1.96146e-3,1.79141e-2,-3.78777e-2,-3.22446e-
  3,1.67228e-2,2.40324e-3,-4.22298e-2,1.42479e-3,-6.75005e-3,5.34782e-3,6.16994e-3,1.28903e-3,-1.12993e-2,-4.69079e-6,-8.17673e-
  4,5.66588e-4,-4.84051e-4,-5.39458e-5,-3.69571e-5,1.75633e-7,7.52905e-9,-1.74284e-6/)
T(:,49)=(/-2.99043e-5,-8.30350e-6,4.50805e-6,-4.18490e-6,5.52681e-6,2.94643e-6,2.73070e-6,3.14247e-6,3.23653e-6,2.03503e-
  6,2.26210e-6,-1.47562e-5,1.71900e-6,-6.13028e-5,5.47845e-4,-2.82003e-5,-1.57815e-5,3.12391e-4,-1.00635e-4,1.12502e-6,-4.94121e-
  3,3.56930e-4,-7.59446e-6,-1.66389e-5,2.64358e-5,-6.59516e-4,-6.14118e-5,-4.44225e-4,-4.89680e-4,-2.73943e-5,-4.78701e-4,-
  1.25252e-4,7.71730e-4,1.36272e-3,8.44390e-4,-9.64183e-4,-7.57013e-4,1.47444e-5,4.34720e-5,-2.39700e-4,-5.14404e-4,3.85261e-
  4,5.74564e-4,1.64497e-3,-2.50125e-4,-3.53098e-3,-3.07889e-5,-1.23619e-1,-7.98779e-1,5.65986e-1,-1.61430e-1,1.49154e-2,1.99007e-
  5,-2.17140e-4,-7.24460e-5,1.05658e-3,-5.59453e-4,-3.83270e-4,6.05753e-10,1.14410e-8,3.13474e-8/)
T(:,50)=(/-3.76122e-5,2.71931e-6,5.96753e-6,-6.02523e-6,7.49240e-6,3.73715e-6,4.22016e-6,4.40028e-6,4.27074e-6,2.69118e-
  6,4.62811e-6,2.74163e-6,2.10201e-6,-2.76214e-4,2.11920e-4,-1.01855e-4,7.29234e-5,5.19954e-3,-1.26941e-5,3.69947e-4,-9.17864e-
  6,-2.22548e-3,6.93727e-4,1.57484e-5,3.53622e-4,4.18911e-4,-1.46523e-5,3.34431e-4,-8.56979e-4,-2.04036e-4,-2.36282e-4,5.83685e-
  4,-8.08317e-3,1.04844e-2,-6.53256e-4,-2.25463e-4,-3.76117e-3,7.92328e-4,2.79948e-4,8.30292e-4,-5.16819e-4,-1.31122e-1,-
  4.92310e-2,-9.88587e-1,-2.86656e-2,3.53662e-2,2.53658e-2,6.45631e-4,-1.54323e-3,1.33821e-3,-7.00966e-6,-6.24792e-3,1.21141e-
  5,7.14782e-5,7.43949e-3,-6.19092e-3,-7.71886e-4,-5.28803e-4,1.14253e-8,1.69116e-8,-7.40422e-7/)
```

```
T(:,51)=(/6.68684e-6,7.41588e-6,-1.04071e-6,3.14011e-7,-6.40481e-7,8.76242e-8,4.47224e-8,6.38185e-9,-9.56330e-8,3.06365e-
  7,1.51565e-7,2.58793e-7,-1.60711e-5,1.21242e-4,-3.24178e-5,-5.41933e-5,-7.59328e-5,-4.98569e-4,-4.82024e-5,-5.72327e-5,-
  4.70075e-5,-2.36330e-1,-1.67642e-4,-3.59804e-4,-1.31759e-4,-4.73599e-5,-1.14447e-5,-1.41301e-4,1.97748e-5,1.37877e-5,-1.03739e-
  4,-9.18891e-5,7.15328e-4,-2.11023e-4,-5.53800e-5,1.54654e-5,4.87236e-5,-8.79819e-6,-6.00217e-5,-1.64234e-4,-6.51774e-
  5,1.80983e-3,3.53455e-4,9.78488e-3,1.19837e-4,4.15243e-4,2.62050e-4,4.82912e-4,-4.07449e-4,9.46533e-4,-1.07910e-4,6.47836e-5,-
  1.20452e-6,-3.96948e-6,6.96803e-1,-6.76814e-1,-1.71932e-2,-1.17787e-2,-2.16718e-9,-8.17916e-9,5.60178e-8/)
T(:,52)=(/-2.87371e-6,4.10577e-6,5.19166e-7,-2.41938e-7,3.02773e-7,3.74599e-8,1.48586e-7,1.16638e-7,-3.28500e-7,-1.32388e-
  7,4.26357e-8,-1.10857e-6,-9.01728e-8,4.50246e-5,-3.34346e-5,9.54194e-6,-1.29194e-5,-1.12558e-2,-1.66466e-4,-1.20026e-5,-
  3.54483e-4,-2.74754e-4,9.81098e-6,-1.14599e-4,-2.29046e-5,-7.37167e-5,2.96426e-5,-5.09868e-5,1.34585e-6,2.12435e-5,1.52686e-5,-
  8.21212e-5,1.62306e-2,-1.58613e-2,3.28165e-3,1.26621e-3,6.94429e-3,1.18549e-4,1.23734e-4,-8.80581e-5,-1.38398e-4,-7.99089e-3,-
  4.49174e-3,-3.05806e-2,-2.18940e-3,-7.78941e-1,1.01002e-3,-1.03777e-1,-1.22663e-1,-2.28043e-1,-4.50493e-2,5.57448e-1,4.62989e-
  5,3.04153e-2,2.21650e-4,-8.60946e-4,3.59220e-4,2.46094e-4,-4.67738e-9,-4.78919e-11,-4.32717e-8/)
```

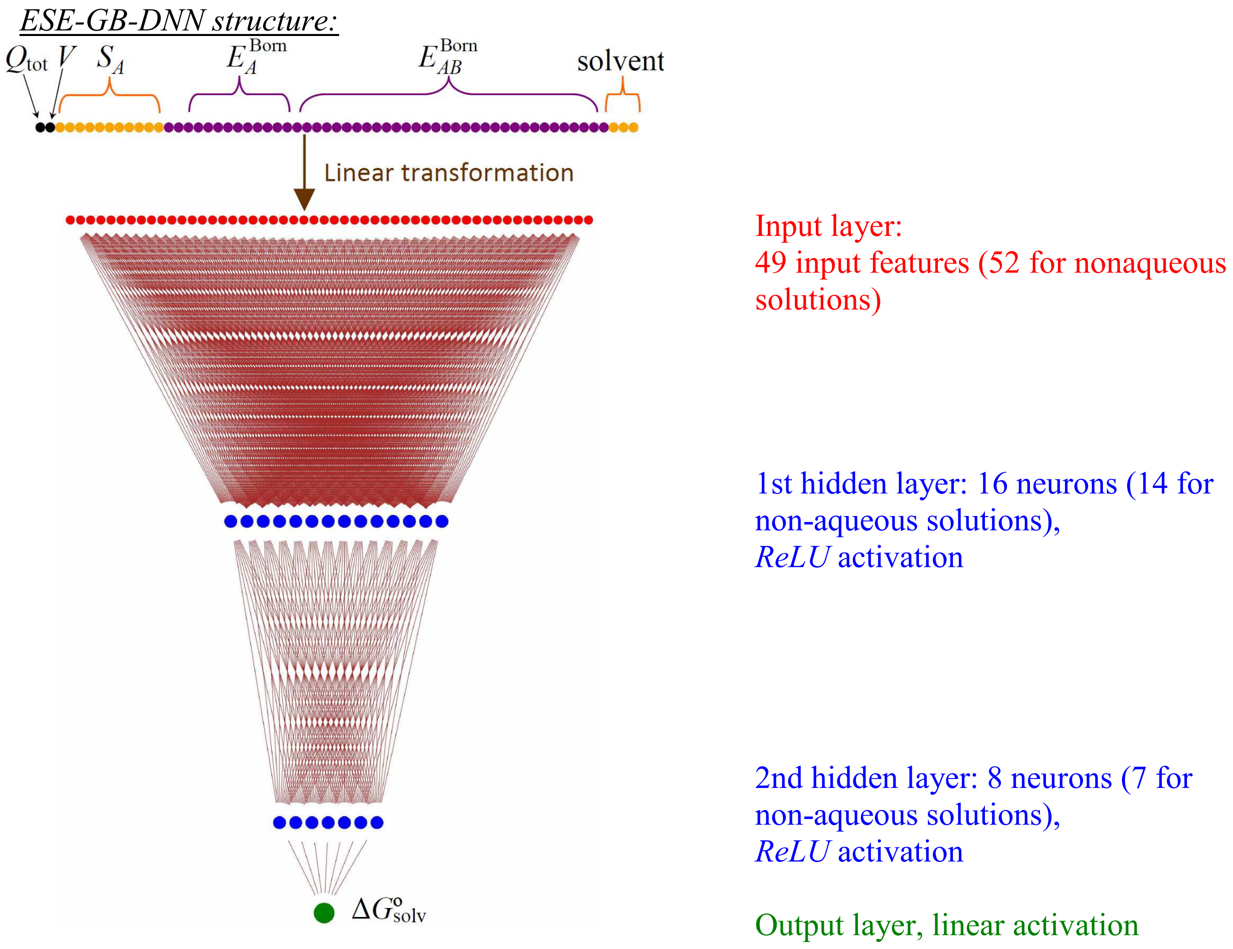
ESE-GB-DNN structure:
Q_{tot}
V
S_A
E_A^{Born}
E_{AB}^{Born}
solvent
Linear transformation
Input layer:
49 input features (52 for nonaqueous solutions)
1st hidden layer: 16 neurons (14 for non-aqueous solutions),
ReLU activation
2nd hidden layer: 8 neurons (7 for non-aqueous solutions),
ReLU activation
ΔG^{o}_{solv}
Output layer, linear activation

ESE-GB-DNN weights and biases – aqueous solutions

Normalizing input features:

```
InputFeaturesNormalized(1:49) = InputFeatures(1:49)/NormDivisor(1:49)
```

where

```
NormDivisor=(/1403.66359, 545.84132, 395.07050, 577.79512, 394.07041, 123.5138, 135.37684,
153.59692, 86.341928, 93.246352, 18.067127, 2.2207542, 3.3086802, 0.8249658, 1.0456705,
0.2813675, 0.3194059, 0.2880499, 0.1575286, 0.2333831, 0.2446127, 0.1296748, 0.2762627,
0.1245198, 0.0883491, 0.0810542, 0.1371979, 0.2143074, 0.0974264, 0.1351170, 0.1546696,
0.0644944, 0.1528315, 0.0787142, 0.0675893, 0.0423616, 0.0345638, 0.0356998, 0.0443300,
0.0278893, 0.0392096, 0.0237092, 0.0270278, 0.0287954, 0.0180279, 0.0093377, 0.0131260,
0.0087289, 0.0095011/)
```

1st hidden layer weights and biases:

```
Weights1(:,1)=(/0.13212422,-0.41911966,-0.11539493,0.02044838,0.18052265,-0.001429,-
 0.03932058,0.06418,0.10243363,0.02713941,0.02977092,-0.9195392,-0.7065884,-0.2757262,-
 0.3810858,-0.11653661,0.01665478,0.14888144,-0.16298854,-
 0.07626038,0.05313623,0.19435827,0.0879399,0.16173401,0.07617078,0.00617771,0.19983414,-
 0.06873269,-0.12202761,0.13523558,-0.00180787,0.05303482,0.01023318,-0.01620149,-0.0716126,-
 0.1366027,-0.0279597,-0.00396092,-0.06261156,-0.14646566,0.05594379,-0.11425094,0.03862128,-
 0.03845054,-0.12653871,-0.0181628,-0.06820507,0.08176014,0.03912159/)
Weights1(:,2)=(/-0.08226077,0.03971612,-0.1684947,0.69,-0.08987,-0.50133324,-
 0.5129117,0.37331924,0.03382277,0.02179703,0.28986794,0.72491705,0.7087946,-
 0.58612317,0.16167627,-0.07950083,0.00430508,0.06535669,-0.00544014,0.17343193,-0.19217223,-
 0.08011922,-0.19577488,-0.04327673,-0.12408924,0.03613532,0.05612348,-0.01131305,0.30434445,-
 0.07282054,0.01171998,0.08553721,0.05448883,0.43746006,-0.31855062,0.14467092,-0.07590342,-
 0.14605147,-0.30714133,0.04055666,-0.27768543,0.09204907,-0.11698295,-0.02248231,0.10376322,-
 0.19363569,0.06753549,-0.05565612,-0.01994/)
Weights1(:,3)=(/0.1140471,-5.84218837e-2,-0.52105284,-0.2278528,0.39603388,-5.963982e-2,-
 8.7788571e-3,3.8530171e-2,0.3146197,-0.14434286,0.12567517,-0.121417,-8.73986557e-2,0.1008118,-
 0.110561,-7.88170546e-2,-0.4443731,0.18437487,0.13026479,0.6482811,0.171130478,-
 0.10497285,0.49101061,0.4429681,6.13982715e-2,0.38244784,-0.16542593,0.20597233,0.16356564,-
 0.1641537,0.24413584,0.14483766,9.7907737e-2,0.266061544,5.57683818e-2,-4.6640113e-2,-
 2.34484836e-3,-0.291809648,-0.177811012,-0.2409468,-1.9174625e-1,1.39084607e-1,2.09302783e-
 1,9.2428714e-2,0.107478984,-3.84665318e-2,7.42486343e-2,-6.15123771e-2,3.36501194e-4/)
Weights1(:,4)=(/-0.0552557,-0.15369558,-0.01456453,-0.10513085,-0.02216036,0.1068028,0.1068881,-
 0.19255675,0.50007665,-0.214,0.2867658,-0.143302187,-
 0.12537,0.083534166,0.14973536,0.07202391,0.105124295,0.082018,0.0510675,-0.029,0.41481882,-
 0.0304377,0.1975627,-0.02975345,0.3987879,0.28510526,0.59404528,-0.247689,2.81505962e-
 3,0.20123127,-0.18197845,0.040807072,-0.01871094,-0.23034197,-2.42360838e-5,-
 0.099351644,6.69155717e-2,-0.31357533,-8.93513262e-2,-3.45653796e-4,-0.13486852,3.19178961e-3,-
 0.100168,0.0923,2.04636864e-2,1.5892135e-2,-0.10829777,3.62371318e-2,7.81704858e-3/)
Weights1(:,5)=(/-0.08801451,0.11888569,-0.32699734,-0.01633595,0.2035664,-0.10394885,-
 0.09924952,0.188322,0.34005338,0.07667182,0.26639315,0.15142892,-0.20955354,0.34279117,-
 0.5242721,0.12070019,0.271317,0.11880621,-
 0.2769835,0.14059699,0.40538073,0.13782476,0.09699672,0.11105592,0.394618,-0.29740173,-
 0.06219565,0.01211345,-0.03172431,0.07400402,-0.21228516,-0.13786146,-0.01560461,0.00683901,-
 0.02359028,-0.19821002,-0.1842105,0.24272572,0.3498288,-0.08333982,-0.23224203,-0.06862497,-
 0.11471479,0.07000755,-0.0690144,0.1205651,-0.06441657,0.05741957,-0.00242756/)
Weights1(:,6)=(/0.38332465,0.19846803,0.08203278,0.03846467,-0.28670827,-0.2580486,0.09277075,-
 0.01254324,0.02321831,-0.03381582,0.01546713,-0.19956584,0.26322186,0.41879842,-
 0.02649899,0.21219005,-0.16400698,-0.13973987,-0.03515531,0.27598298,0.09365747,-0.04157788,-
 0.0578048,-0.3105087,-0.04827464,0.29611203,-0.15280584,0.02374559,0.04112334,-
 0.03490833,0.19428964,0.11752813,0.02175987,0.03835513,-0.04058621,0.39898634,-0.10344344,-
 0.2166548,-0.08876757,0.29095012,-0.05593716,-0.05752352,0.02971162,0.00363989,-
 0.01695033,0.20105587,-0.06733573,0.03760991,-0.01226398/)
Weights1(:,7)=(/0.05274509,-0.32494807,-0.03598604,-0.04522964,-0.09749544,-0.3233026,-
 0.05300232,0.01581936,-0.00534503,-0.01952865,-0.29115808,-0.01020298,0.16806765,-0.12758183,-
 0.14182033,-0.1309198,-0.09067334,-0.03467274,0.02913816,-0.02885022,0.1851336,-0.02413099,-
 0.6847534,-0.3034366,0.15296164,-0.2762394,0.21284464,-0.11126976,-0.2442129,-
 0.03041556,0.21403444,0.26919493,0.06175859,-0.04294329,-0.13792337,0.01105944,-
 0.09343975,0.15460694,-0.05635246,0.2430953,0.0733528,-0.10235623,0.02113528,-0.05624525,-
 0.07716646,0.2795519,-0.09490003,0.04560892,-0.02238454/)
Weights1(:,8)=(/-0.05803213,-0.14941935,-0.05259439,0.14174497,-0.1023269,-0.15113196,-
 0.21447709,0.12857237,0.02200485,-0.06087433,-0.60808355,-0.31264985,0.12795258,-
 0.0625762,0.08442567,0.15282162,0.12005385,-0.16184652,0.04910361,-0.15537614,0.20363262,-
 0.1307211,-0.15085493,-0.31524065,0.15247354,-0.57401115,0.2846606,-0.16538648,0.16375048,-
 0.06541184,0.03478033,-0.31959158,0.06290145,-0.12847723,-0.24595924,-
 0.12319018,0.07405879,0.53155977,0.06070305,-0.06334665,-0.02125533,-0.06782059,0.24503583,-
 0.12233281,-0.17269783,0.01301835,-0.03473412,0.05414624,0.01517976/)
Weights1(:,9)=(/-0.10412553,-0.13276377,-0.1573642,-0.18418987,-0.0199584,-0.10652985,-
 0.1620908,0.11247204,0.02033024,-0.02809203,-0.44547704,0.05549695,0.07750761,-
 0.0647474,0.7031132,0.15873489,0.10592724,-0.0552848,-0.10728838,-0.22970793,0.00396172,-
 0.01727509,-0.51565,0.03517681,0.02307752,0.05782448,0.28571516,-0.18702099,-
 0.2047554,0.13908707,-0.20628569,-0.20634179,0.00991546,-0.37093443,-0.04212806,-
```

0.11377688,0.28133455,-0.36265165,-0.5323336,0.05975164,0.07483839,0.27108258,-
0.13453583,0.19049084,0.2669062,0.0844321,0.01900861,0.04980435,-0.041806/)
Weights1(:,10)=(/-0.38300505,0.12910333,-0.14445794,0.01912307,-0.04103856,-0.11277289,-
0.09203198,0.0329902,-0.24842125,0.01083095,-
0.08590092,0.08900455,0.0935027,0.14879145,0.07707642,0.21609737,0.22966772,-
0.08887384,0.0391213,-0.18877238,-0.41750088,0.0691181,-0.05642332,-0.1241743,-0.46981427,-
0.01127185,-0.05292799,0.04120445,0.08553178,-0.06050952,0.02672406,-0.09064043,-
0.02895061,0.01845668,-0.01725007,0.1905483,-0.23541899,0.54667306,-0.16011377,0.04768407,-
0.17689268,-0.08637591,-0.00354902,-0.09431585,-0.10266083,0.11451584,-0.030475,-
0.01764598,0.00452661/)
Weights1(:,11)=(/0.13034652,-0.16320674,0.06891795,0.47930345,-
0.18128897,0.02321196,0.27210858,-0.24693348,0.11842404,-0.08645843,0.08704672,-0.22693314,-
0.13631825,0.12239894,0.0923852,-0.18450038,-0.17154872,0.01396863,-
0.05805064,0.25047415,0.10067119,-0.18890098,-0.189189,-0.00506749,-0.06406035,-
0.01133212,0.16394167,-0.07330277,-0.28221545,-0.00353114,-0.05455244,-0.00117446,0.04613731,-
0.33819902,-0.18686906,-0.40224364,-0.3480168,0.09901976,0.1154,0.16646156,-0.01417269,-
0.17931631,-0.12024027,0.04011194,-0.07925185,0.21440321,-0.05942878,-0.01927163,0.02095801/)
Weights1(:,12)=(/-0.11789138,0.18481193,0.12421633,0.38302454,-0.1611938,0.09581784,0.24488574,-
0.23945145,-0.33479455,0.204797,-0.335872,0.06331426,0.04221674,0.539664,-0.01225367,-
0.13708445,-0.29951987,0.03011453,-0.24920301,-0.05691066,0.17739792,-0.02131511,-0.50424397,-
0.07242468,0.10400425,-0.01598547,-0.07992388,0.04541118,-0.0739353,-
0.20616162,0.24710463,0.20824808,-0.06968328,0.33990106,0.25669938,0.15724175,-
0.38035402,0.07383002,0.04809281,0.23044018,0.03241165,-0.19119333,0.05237355,-0.21601835,-
0.11869211,0.04583658,-0.07461631,-0.02148947,-0.01059983/)
Weights1(:,13)=(/0.02494168,0.02878706,0.17382982,0.35343418,-0.02512072,0.04941596,0.25077838,-
0.13517638,0.36401302,-0.08785538,0.07247818,0.09987034,0.07234938,0.07011916,0.18771215,-
0.17815909,0.08521568,-0.08102245,-0.13064982,-0.12009542,0.02468003,-0.266047,-0.08601038,-
0.07869684,-0.09593315,-0.02704624,0.21880239,-0.12078259,0.05501874,-
0.03703563,0.01633614,0.04679911,0.00692864,-0.04049009,0.0822802,-0.08177163,-
0.38360333,0.10125124,-0.08576416,0.07767453,0.05443002,-0.05536793,0.02621718,-0.02299514,-
0.0539625,0.01222268,0.0176341,-0.02503129,0.01158067/)
Weights1(:,14)=(/-0.24074772,2.75732148e-2,0.234605536,0.20951365,-
0.109704256,0.136011615,0.25623858,-0.259372264,-9.47102383e-2,1.85212283e-3,-
0.2975661,1.03859222,0.56615877,-0.341507465,0.14841485,9.661378e-4,-0.434383154,-9.97951487e-
4,-1.89954098e-2,0.18614896,0.11503,-0.137127,-0.19904205,-0.31953371,6.27306551e-2,-
0.249845415,4.70259227e-2,-2.8725611e-2,1.79603342e-2,1.35856532e-2,-6.08685277e-2,-
7.55923316e-2,4.10419963e-2,-0.31236872,-0.14649297,6.4310074e-2,0.20624904,5.40288948e-
2,3.14191612e-3,-8.45232308e-2,1.97352711e-
2,0.11811192,0.12699419,0.127869472,0.1296927,2.18357425e-2,-4.11554845e-3,2.56694551e-3,-
3.54718557e-3/)
Weights1(:,15)=(/-0.1437251,-0.17338218,-0.12237176,-0.21833721,-1.956221e-2,-0.16819435,-
0.11395697,0.10801233,0.0199205,-1.7162092e-2,-0.313266784,5.11546321e-2,0.189500138,-
0.27335647,0.2815517,8.68056566e-2,0.11888243,9.2929192e-2,-2.68388633e-2,-5.03523648e-
3,0.30993244,-0.23234102,-0.485507816,-3.68245505e-2,4.13874984e-2,-0.46690282,0.1710339,-
4.45197187e-2,-2.08396893e-2,2.47920398e-2,-1.77285587e-3,1.58632137e-2,5.28942607e-2,-
0.264354676,6.92000103e-5,-7.10147321e-2,3.71763781e-2,-9.1352798e-2,-
0.503904045,0.158592314,8.01256597e-2,9.04467925e-2,-
0.0152113233,0.042057812,0.127713,0.18766676,-0.010794469,-3.39084608e-3,6.92640152e-3/)
Weights1(:,16)=(/-0.2149978,-0.09429916,0.16863161,-0.4666432,-0.04083424,0.0104782,-
0.04218455,-0.04501119,0.07008757,-0.03087826,-0.34951058,-0.19537969,-0.38946253,-0.09411,-
0.7755562,0.04685082,-0.21827264,0.12790619,-0.22233385,0.18795134,-0.19608162,0.01190267,-
0.61289585,-0.09421796,0.04687165,-0.36382344,0.5272329,-0.28992504,0.04901671,-
0.07795319,0.07220788,-0.02270439,0.0569473,-0.4033211,-0.40451747,0.00494363,0.16103475,
-0.40521356,-
0.5500779,0.24400589,0.08501278,0.14546506,0.0764162,0.05910161,0.20618102,0.04153986,-
0.0354287,-0.03527899,0.0959208/)
Biases1=(/0.0198,-0.0112,0.0922,0.0842,0.1059,0.0972,-0.071,-0.0102,0.0042,0.092,0.0077,-
0.0696,0.095,-0.0599,-0.0014,-0.0374/)

2nd hidden layer weights:

Weights2(:,1)=(/,0.56510586,0.7182803,-0.4039164,0.44951114,-
0.39260137,0.08420291,0.41602594,0.4665283,0.50707775,-0.3990527,0.3968,-
0.576461,0.05713014,0.5395586,0.4263016,0.6448816/)
Weights2(:,2)=(/-0.14556369,-0.08165083,0.2635351,-0.07939805,0.22869709,0.3818262,-0.29685637,-
0.28730863,-0.10358241,0.247904,-0.19871649,0.18266587,-0.3594234,-0.0384645,-0.15523623,-
0.12263589/)
Weights2(:,3)=(/,0.4962543,0.7272149,-0.4730078,0.31616044,-
0.47268206,0.02922746,0.397271,0.4147811,0.49833098,-0.45908675,0.29257706,-
0.41795516,0.20943739,0.5890934,0.4551705,0.6076437/)
Weights2(:,4)=(/,0.50519574,0.80428946,-0.46115148,0.5946093,-
0.33336765,0.20906512,0.40214998,0.39744234,0.48405042,-0.3580484,0.36880016,-0.5419065,-
0.00967289,0.59760064,0.41217554,0.62513506/)
Weights2(:,5)=(/,0.22722723,0.05693208,0.5742575,0.44967934,0.42292875,-
0.8721391,0.05060035,0.65913373,0.49391347,-0.00502491,-0.3875583,-0.066555,-
0.20724575,0.43251187,0.28574702,0.7202434/)

```
Weights2(:,6)=(/-0.24833477,-0.10700491,0.241886,-0.16309491,0.24101017,0.34948954,-0.25769982,-
 0.3283426,-0.24965039,0.26570767,-0.20620637,0.23217678,-0.30143878,-0.01564024,-0.20401917,-
 0.18877688/)
Weights2(:,7)=(/,0.49930125,0.6276411,-0.5414536,0.26754367,-
 0.34239572,0.0427918,0.3528969,0.39625016,0.47843835,-0.33756718,0.24804689,-
 0.40437603,0.22543725,0.5123259,0.40358508,0.5800927/)
Weights2(:,8)=(/,0.4807646,0.6820421,-0.5492519,0.27094343,-
 0.3297589,0.03269373,0.36214525,0.39481148,0.50176674,-0.32402012,0.24954608,-
 0.4270434,0.23700689,0.56597257,0.417618,0.5754994/)
Biases2= (/0.0327,0.1617,0.0742,-0.0018,-0.4517,0.1708,0.1037,0.1044/)
```

Output layer weights and bias:

```
Weights3(:,1)=(/-17.141348,11.107002,-16.472565,-17.844854,38.116474,11.913367,-15.732109,-15.85938/)
Biases3=(/-3.24/)
```

ESE-GB-DNN weights and biases – non-aqueous solutions

Normalizing input features:

```
InputFeaturesNormalized(1:52) = InputFeatures(1:52)/NormDivisor(1:52)
```

where

```
NormDivisor=
 (/934.4,430.09,309.68,295.93,143.25,310.35,328.3,83.107,105.0,111.13,58.12,89.91,33.643,
 4.4951,5.4796,2.1397,0.43471,0.27142,0.25536,0.16547,0.15545,0.23095,0.14512,0.1625,0.18388,0.1
 2344,0.19715,0.12193,0.087574,0.14772,0.079527,0.074393,0.084211,0.054373,0.041536,0.037823,0.1
 08814,0.0525072,0.0280037,0.0208638,0.023599,0.024583,0.028834,0.017004,0.013399,0.016807,0.010
 306,0.0089631,0.0043172,0.0027051,0.0027619,0.002607/)
```

1st hidden layer weights and biases:

```
Weights1(:,1)=(/-0.05453372,-0.01498948,-0.05620975,-0.20848791,0.19362341,-0.03210346,-0.038236,-
 0.28411973,0.01801342,0.02149714,-0.33293748,0.11678406,-0.21577416,0.10030062,-
 0.03172165,0.06478438,0.06286266,-0.24235758,0.01861281,-0.05151421,-
 0.08090021,0.1688948,0.04536343,0.18959953,-
 0.06855453,0.29636472,0.11359198,0.03976344,0.02407823,-
 0.2308061,0.23446001,0.05551448,0.15825179,0.1589722,-0.05521064,0.20429516,-0.02477232,-
 0.06735907,0.09202351,0.1863467,-0.15577145,-0.01794506,0.12630416,-0.07364978,-
 0.05598577,0.03508407,0.05643088,-0.02962044,-0.00439072,0.05831053,-0.02384328,-0.01139633/)
Weights1(:,2)=(/0.2660111,0.512714207,-2.55231969e-2,-0.11444175,2.59534456e-2,0.35701868,-
 0.19280811,-0.13373764,-7.62668401e-2,0.1080558,2.17237473,-0.21215138,-0.11927091,-0.22836979,-
 2.55448334e-2,-0.20099738,0.10738437,0.1350091,0.11337963,-1.45381596e-3,2.74114721e-
 2,1.30958471e-3,0.158222511,-4.31180298e-2,1.91327941e-3,-0.19374597,6.45776466e-2,3.08302254e-3,-
 0.24446306,0.11414784,-0.10848605,4.87648286e-2,0.2814748,6.14092015e-2,0.176676646,-9.8900661e-
 2,0.10151872,-9.86961871e-2,0.125851586,-0.25048962,2.62721651e-3,4.0133195e-3,-
 0.163960546,0.122144625,-2.21638544e-3,4.9467436e-3,-0.10976039,2.34462358e-2,4.24383767e-2,-
 7.85175711e-3,-8.47598538e-3,-2.00786665e-2/)
Weights1(:,3)=(/5.1160574e-2,2.95252144e-1,-1.94930956e-1,-2.07904428e-1,5.64065054e-2,1.09846786e-
 1,2.08919942e-1,-6.64022043e-2,-1.5787898e-1,9.72051546e-2,8.27844322e-1,-2.15978865e-
 2,2.00690944e-2,1.17857277e-1,-4.20225382e-1,1.8476072e-1,-4.47324544e-1,-3.07860464e-1,-
 1.10581972e-1,-9.21026766e-2,-1.46576427e-2,1.35232762e-1,1.34421393e-1,-2.31998652e-
 1,1.09216059e-2,1.61337361e-1,-6.41986802e-2,2.43645728e-1,2.51859218e-1,-9.42847207e-
 2,3.26040894e-1,1.81107908e-1,-5.08844078e-1,1.46456867e-1,1.52067482e-1,-2.32098624e-1,-
 1.80464089e-1,1.46604273e-2,-3.05620581e-1,2.21978091e-2,1.67585805e-1,-2.49188114e-2,5.72245277e-
 4,8.98764133e-2,-4.52100188e-2,1.20446064e-1,3.95267457e-2,8.70948136e-2,-3.30571495e-
 2,1.40837774e-1,8.62544985e-4,9.28246044e-3/)
Weights1(:,4)=(/7.0556033e-1,3.5331991e-1,-2.2440536e-1,-1.8922079e-2,8.7055452e-2,2.0782518e-
 1,8.7334299e-3,-4.9948938e-2,3.987157e-2,5.002635e-2,1.1008016,-5.014471e-2,8.1305139e-
 2,4.7874479e-3,-2.1020088e-1,2.7388811e-1,-3.3171067e-1,-2.4622913e-1,-2.27498e-2,1.7625749e-1,-
 8.3508626e-2,-1.5071957e-1,1.1335479e-1,-1.6950171e-2,-5.2759688e-2,4.472905e-2,6.8009533e-2,-
 1.161057e-1,0.5749924,-0.40609,0.4744941,-0.1533429,-0.7039304,6.8282202e-2,-3.0495655e-2,-
 0.192988,6.5568477e-2,0.231523,-0.1718837,0.1781014,1.5625658e-2,1.5262666e-2,2.22759e-
 2,6.9765948e-2,-4.8552528e-2,3.9490428e-2,3.7058515e-3,0.1385307,-8.7382803e-3,8.4150784e-
 2,1.0352969e-3,-2.3822628e-3/)
Weights1(:,5)=(/-0.123711,9.9430695e-2,-0.263243,-6.2056735e-2,-0.2138815,6.8323739e-2,1.3306155e-
 2,4.749085e-2,-2.7879504e-2,-6.1529856e-2,-4.6882622e-2,-3.1596649e-2,-3.7587751e-2,8.2272394e-3,-
 8.0628604e-2,-5.2683357e-2,6.5889157e-2,-3.0162636e-2,-0.1482493,-4.1613836e-2,3.1834632e-2,-
 0.1356085,-0.132855,-0.1036654,2.5858611e-2,-0.1189896,0.141667,-6.5352172e-3,0.2165559,-
 0.1204647,-6.8491176e-2,2.0934699e-2,4.1019078e-2,-7.7881508e-2,3.1327721e-2,3.6077346e-3,-
 1.2665356e-2,6.0028356e-2,-2.89669e-2,5.5823172e-3,4.2775948e-2,-2.033761e-2,2.9878015e-2,-
 1.612499e-2,1.711404e-2,1.6528454e-2,1.2616759e-2,-4.8660528e-2,-2.1062131e-4,5.3879309e-
 2,1.557876e-2,-5.4777153e-3/)
Weights1(:,6)=(/-0.2669669,-0.474368155,8.04488137e-2,0.14066623,1.47929247e-2,-
 0.3611088,2.35875938e-2,-3.06852367e-2,4.41393517e-2,-2.08907034e-2,-1.78095448,1.03680804e-
 1,2.08133413e-3,5.6259796e-2,-1.5860633e-3,1.71404824e-1,0.26261652,-0.17038021,-8.28043446e-2,-
 0.29558423,0.1449277,-0.41815367,0.12440316,-7.08977357e-2,-0.12221456,-5.17618582e-2,-
 0.13923354,-6.47505894e-2,-2.82980185e-2,6.57531479e-3,0.30317503,9.5264297e-3,0.203232154,-
 0.24796185,6.0889177e-2,9.16822478e-2,-2.69431956e-2,9.2041254e-2,0.1554075,-4.62782383e-
 2,6.69787005e-2,9.74240899e-2,-2.44945753e-2,-1.8326778e-2,-2.96438038e-2,-2.13143486e-
 3,5.27838729e-2,2.63322107e-2,1.55597329e-2,7.09137842e-2,4.19397559e-3,-2.04094686e-3/)
Weights1(:,7)=(/-0.17957346,-0.30869505,0.03094378,-0.14364469,-0.16295858,-0.24139479,-0.1744137,-
 0.09706963,-0.06839578,0.0245449,-1.2724223,0.11262248,0.204075,0.13073598,-
 0.00930762,0.01515187,-0.05111494,-0.02794358,0.00864384,-0.0069008,-
 0.03665242,0.11411826,0.03216991,-0.1673619,0.01550685,0.20581149,-
 0.06942538,0.12389652,0.14491712,0.20280784,0.0139856,-0.06806564,-0.0409781,-
 0.03453795,0.19550683,-0.13762431,0.04624464,0.11766555,0.1975842,0.15070887,0.02812315,-
 0.11883412,0.09604182,-0.09883226,-0.02407771,-
 0.05922855,0.06010196,0.13365033,0.01266871,0.03550606,0.00152793,-0.01725087/)
Weights1(:,8)=(/0.239089,0.57986194,-0.198983,0.1436641,1.27937235e-2,0.2907345,-0.12030625,-
 0.20102365,-3.98310274e-3,9.21763182e-2,2.11794996,-0.12798087,-5.4690022e-2,7.19254687e-2,-
 0.1294174,0.12414458,-0.2777753,-3.57863568e-2,-4.76912446e-2,-5.41759729e-2,1.97821893e-2,-
 6.35759681e-2,0.14143543,0.106353,2.70236488e-2,-7.70508572e-2,-3.34735401e-3,-5.1211793e-
 2,0.11646863,-5.84529489e-2,0.271749,-8.18771422e-2,-0.10588867,0.15437582,-0.17702374,-
 0.2445084,2.58367625e-4,0.258523,0.114288,0.1283432,-2.31576189e-2,-8.45959503e-3,3.90924551e-2,-
```

9.35835242e-2,-1.34289274e-2,0.1244624,4.26040702e-2,9.2520982e-2,-1.70976855e-3,-4.11799597e-3,-1.07934349e-3,-1.80972777e-2/)

Weights1(:,9)=(/0.10651229,0.11565,-1.63323488e-2,-0.4534247,0.11675715,0.13211972,1.5213565e-2,0.138571,-9.38119516e-2,2.19363589e-2,0.4547426,-9.65507627e-2,-9.50603653e-3,-0.2446161,5.24223857e-2,-2.63675656e-2,7.48308434e-4,0.26325616,0.15851168,-0.1247327,0.3604253,7.16537535e-2,-3.49583924e-2,-0.369939,-7.25066066e-2,-6.17377646e-2,5.45675419e-2,-1.129171e-3,-0.10977326,-2.29003634e-2,8.40250403e-2,-0.10724634,-0.211693,3.46155874e-2,8.39977758e-4,-6.12039603e-2,1.21197039e-2,5.69684431e-2,-7.1284771e-3,9.25147086e-2,2.90245889e-3,-1.26163457e-2,-7.29445321e-3,-2.62173582e-2,7.29922131e-2,1.16449306e-2,-1.1100596e-4,1.16225835e-2,7.60477921e-3,-1.72007247e-3,5.56048378e-3,8.04856606e-3/)

Weights1(:,10)=(/-0.07194883,0.04669157,0.01080701,0.02042615,0.08625676,-0.17197502,-0.13009432,-0.15878664,0.0835073,-0.03902385,0.33316875,0.10106674,0.59812534,-0.27433145,0.04681928,-0.10464077,0.02279309,0.38241646,0.0649581,0.23272356,-0.20224515,0.05603916,0.26224828,-0.13889903,-0.0132769,0.06739067,-0.0187351,0.06778862,-0.14781584,0.11054512,0.09666921,0.09718454,0.2510629,-0.01107379,-0.17364863,-0.06017489,-0.07504316,0.1181514,0.10778715,-0.05543452,-0.0434122,-0.00245821,0.03223608,-0.03323244,0.00633287,-0.10282937,-0.1381818,-0.05950256,0.02388211,-0.098362,-0.02855028,0.01687189/)

Weights1(:,11)=(/0.1544647,0.3298142,-0.4462749,4.8232298e-2,1.998532e-2,5.5832304e-2,-2.4155201e-2,-1.6089788e-2,2.3459585e-2,5.311335e-3,0.14929,-1.7133554e-2,-1.5342945e-2,0.140897,-1.4068723e-3,-6.8396866e-2,6.3214555e-2,1.9204764e-2,3.8126563e-3,3.6512353e-3,-5.5927515e-2,-4.5042813e-2,1.1219042e-2,-8.6657666e-3,1.0473699e-2,8.3238915e-2,-8.519236e-3,6.6452965e-2,-1.842927e-2,-7.209225e-2,-5.6666233e-2,-9.2829779e-2,-5.3361263e-2,-2.9481011e-2,2.8134996e-2,1.4141335e-2,1.875402e-2,5.1203296e-3,2.0081941e-2,3.0208301e-2,3.7673265e-2,-5.3731322e-2,-2.2712661e-2,-8.7189659e-2,4.6175946e-2,3.389575e-2,-7.5633176e-2,2.0269892e-4,2.7355654e-2,-7.1187191e-2,-1.5659926e-3,-8.9396145e-3/)

Weights1(:,12)=(/4.85510349e-1,1.44695878e-1,-1.6080229e-1,1.97820038e-1,5.14298752e-2,1.27450928e-1,1.07763529e-1,-8.59113708e-2,2.78581679e-2,-1.48687121e-2,3.50286752e-1,-2.00751871e-2,-3.09595585e-1,-6.41439296e-3,-2.80071795e-1,0.3914177,-0.6473637,-0.48495147,-0.2406,0.32344,2.02463493e-1,4.90139127e-1,-0.22027,-0.2713693,0.13283144,-0.14056127,5.03720418e-2,-0.2275987,0.3209953,-7.95914307e-2,-0.10016161,0.10216542,-0.2518949,0.299008,-0.2583233,2.54080705e-2,3.77107561e-2,4.91108857e-2,-3.79674286e-2,8.45643431e-2,-7.55496025e-2,8.64805505e-2,0.1100778,1.30895935e-2,-6.64937496e-2,6.80346712e-2,0.143581,6.41650637e-4,-4.0694721e-2,-4.36829627e-2,2.20214725e-2,1.41927116e-2/)

Weights1(:,13)=(/0.387372,0.04712003,-0.05761131,0.25998044,0.15885241,0.03439786,0.0232806,0.29811338,-0.02439706,0.0625618,0.17823234,-0.01833663,0.18992962,-0.08157422,0.14137453,0.08901384,0.2685681,0.12393,0.247489,0.40308115,-0.14394628,0.11169865,0.00875409,-0.05598452,0.04840746,-0.01244922,-0.18758416,-0.19658521,0.05387965,0.11172066,-0.15871881,0.0746055,0.00366294,-0.2620015,-0.13090219,0.11711968,-0.02527752,-0.06034221,-0.19307572,-0.03371379,0.07543067,-0.06168343,-0.09615686,0.06207946,0.03983985,-0.00263716,-0.04950107,0.10570107,0.05016094,0.06526279,-0.02496891,-0.06142782/)

Weights1(:,14)=(/0.80821,0.27991387,-0.2378547,0.09735874,-0.1842106,0.3725269,-0.06952686,-0.40927103,0.22914107,-0.1114485,0.82839394,-0.03543518,-0.07131948,-0.03737136,-0.29760697,0.3934191,-0.24996646,-0.4324659,-0.3756315,0.15594043,0.05322234,-0.10043797,-0.07247844,0.15309025,0.03102183,-0.10606016,-0.08800026,-0.3294748,0.40991786,-0.21050623,0.54898214,-0.26581168,-0.6073325,0.16498765,-0.25220048,-0.1612754,0.07866817,0.29293865,-0.01367818,0.10773782,-0.03694266,0.08262341,0.03641693,0.10196839,-0.09086677,-0.02326503,0.03416013,0.03516447,-0.0353443,0.12025614,0.00939402,0.03256761/)

Biases1= (/0.1178,-0.273,0.2638,0.1123,0.0533,-0.0238,0.2269,0.0227,-0.123,0.0444,0.0619,0.2094,-0.035,0.0709/)

2nd hidden layer weights:

Weights2(:,1)=(/-0.38693306,0.18658678,-0.45609334,-0.2389182,-0.33136576,0.39285508,-0.18664764,-0.14555362,0.18505295,0.216647,0.09366871,-0.4235578,0.55084264,-0.33202717/)

Weights2(:,2)=(/-0.15559159,0.8638379,-0.4087239,-0.7435897,0.01640199,0.6478928,0.48329693,-0.771969,0.23865731,0.2646615,-0.15782519,-0.39841506,-0.19582884,-0.692267/)

Weights2(:,3)=(/-0.12182322,0.8105745,-0.40534014,-0.7512441,0.05342652,0.6463024,0.45652393,-0.7428306,0.21660905,0.17300613,-0.14460607,-0.39012882,-0.17552723,-0.68775094/)

Weights2(:,4)=(/-0.1408059,0.8185763,-0.40263718,-0.74316984,0.03978247,0.6402057,0.4378228,-0.7783392,0.20984173,0.21157964,-0.15058032,-0.37032557,-0.16698633,-0.6829547/)

Weights2(:,5)=(/-0.13869366,0.829773,-0.4,-0.7552143,0.02692816,0.5899541,0.46034804,-0.77175534,0.209,0.18743114,-0.1629915,-0.34404477,-0.16925767,-0.6919108/)

Weights2(:,6)=(/-0.38591522,0.19551378,-0.44863015,-0.2600623,-0.33275536,0.42614153,-0.17956327,-0.18431185,0.22611403,0.28317463,0.06683218,-0.4090219,0.60961896,-0.34868616/)

Weights2(:,7)=(/-0.11509894,0.83782065,-0.39452338,-0.7001322,0.0611072,0.6361319,0.4206644,-0.7523181,0.21509257,0.20277354,-0.16487151,-0.35395896,-0.19229048,-0.64563715/)

Biases2= (/0.3256,0.2778,0.2551,0.2505,0.2660,0.3456,0.2538/)

Output layer weights and bias:

Weights3(:,1)= (/-19.79797,-36.600742,-36.534344,-36.56224,-36.815292,-20.882866,-36.958862/)

Biases3=(/-1.31/)

Part 2 – Statistical results

Table S1. Mean signed error (MSE), mean absolute error (MAE), and the root-mean-square error (RMSE) of the hydration free energy in kcal/mol for various datasets by the ESE-GB-DNN method in comparison with other semi-empirical methods.

Solutes[a]	ESE-GB-DNN			uESE			SMD			ESE-EE		ESE-PM7		ESE-PM7(SN)		PM7/COSMO2
	MSE	MAE	RMSE	MSE	MAE	RMSE	MSE	MAE	RMSE	MAE	RMSE	MAE	RMSE	MAE	RMSE	RMSE
MNSol(528)	-0.034	1.073	1.843	-0.063	1.472	2.237	2.064	2.528	4.187	2.37	3.34	2.00	2.79	1.90	2.62	
neutrals(390)	0.102	0.802	1.302	-0.144	0.992	1.482	0.571	1.152	1.697	1.53	2.04	1.62	2.21	1.48	1.96	
cations(60)	-0.533	1.793	2.587	0.116	2.734	3.431	3.442	3.758	5.081	4.15	5.09	3.13	3.91	3.37	4.20	
anions(82)	-0.326	1.861	3.041	0.192	2.831	3.663	8.087	8.104	8.965	4.36	5.41	3.01	4.03	2.85	3.72	
MNSol*(464)	-0.035	1.021	1.668	0.024	1.408	2.159	2.211	2.501	4.233	2.32	3.31	1.91	2.64	1.82	2.53	2.62
neutrals (330)	0.057	0.732	1.087	-0.102	0.847	1.254	0.616	0.962	1.384	1.70	2.29	1.46	1.90	1.33	1.72	2.24
cations (59)	-0.532	1.794	2.587	0.175	2.724	3.432	3.451	3.773	5.110	4.15	5.09	3.13	3.91	3.37	4.20	2.87
anions (75)	-0.046	1.683	2.601	0.461	2.843	3.558	8.249	8.268	9.049	4.34	5.38	2.91	3.91	2.73	3.56	3.69
Mobley141(141)	-0.125	0.846	1.296	-0.892	1.421	3.381	0.068	1.502	3.015	1.64	2.22	1.25	1.72	1.19	1.65	2.54
Blind(63)	0.360	1.254	2.153	-0.774	2.056	2.948	-0.455	2.370	3.542	2.69	3.42	2.53	3.49	2.30	2.94	
SAMPL1(53)	0.713	1.121	1.702	-0.335	1.421	1.854	0.422	1.617	2.594	2.36	2.96	2.35	3.50	2.30	2.91	3.73
SAMPL4(42)	-0.344	1.075	1.499	-0.388	1.283	1.666	0.519	1.027	1.226	1.85	2.42	1.28	1.60	1.34	1.59	1.92
C10(10)	-1.182	1.936	2.587	2.170	3.010	3.486	3.999	3.999	5.452	6.18	6.87	1.65	2.22	1.77	2.31	2.28

[a] The number of entries in the dataset is given in parentheses.

Table S2. Mean signed error (MSE), mean absolute error (MAE), and root-mean-square error (RMSE) of the hydration free energy calculated by the ESE-GB-DNN method in comparison with DFT-based SMD and uESE methods for various classes of neutral solutes from the MNSol database[1] (in kcal/mol)[a].

**Solute class**		**Solvation scheme**		
		ESE-GB-DNN	uESE[b]	SMD[c]
Small molecules(24)[b]	MSE	–0.474	–0.40	–0.05
	MAE	0.879	0.53	0.46
	RMSE	1.227	0.68	0.63
Alcohols(18)	MSE	0.066	–0.15	0.82
	MAE	0.331	0.61	0.82
	RMSE	0.423	0.73	0.87
Aldehydes and ketones(22)	MSE	0.419	0.42	0.26
	MAE	0.569	0.60	0.50
	RMSE	0.729	0.73	0.77
Ethers(10)	MSE	0.152	–0.88	0.90
	MAE	0.456	1.02	0.99
	RMSE	0.561	1.15	1.07
Esters(21)	MSE	–0.325	–0.20	0.54
	MAE	0.657	0.42	0.62
	RMSE	0.719	0.64	0.87
Acids(10)	MSE	0.712	–0.64	1.82
	MAE	0.926	0.64	1.82
	RMSE	1.426	0.75	2.01
Amines(42)	MSE	0.110	–0.28	0.81
	MAE	0.831	0.94	0.87
	RMSE	1.120	1.48	0.95
Nitriles(4)	MSE	0.103	0.01	0.21
	MAE	0.253	0.32	0.26
	RMSE	0.291	0.37	0.37
Nitro compounds and nitrates(15)	MSE	–0.051	1.06	1.46
	MAE	0.540	1.15	1.60
	RMSE	0.688	1.39	1.99
Fluorine compounds(33)	MSE	0.226	–0.23	0.31
	MAE	0.686	1.02	1.08
	RMSE	0.928	1.46	1.49
Chlorine compounds(74)	MSE	0.216	0.03	0.75
	MAE	0.727	1.06	1.63
	RMSE	1.084	1.64	2.23
Bromine compounds(25)	MSE	0.454	–0.06	0.30
	MAE	0.920	0.70	0.97
	RMSE	1.945	1.30	1.60
Iodine compounds(10)	MSE	–0.236	0.15	0.14
	MAE	0.458	0.73	0.81
	RMSE	0.534	1.40	1.35
Linear correlation[c] (for all 389 neutral solutes):	Slope	0.892	0.928	0.921
	Intercept	–0.37	–0.46	0.23
	R^2	0.914	0.8903	0.8728

[b] Molecules containing less than six atoms.
[c] Linear correlation between $\Delta G^{\circ}_{\text{solv}}$ obtained within a given method and the reference $\Delta G^{\circ}_{\text{solv}}$ value.

Small molecules: 0001met, 0030eth, 0153flu, 0160chl, 0161dic, 0162tri, 0177bro, 0178dib, 0179tri, 0197bro, 0198chl, 0199chl, 0200tet, 0216amm, 0217wat, 0218pho, 0219hyd, 0400hyd, 0421dfl, 0422ftc, 0423brt, n017, test4002, test4003

Alcohols: 0044met, 0045eth, 0046eth, 0047pro, 0048pro, 0049but, 050met, 0051cyc, 0052pen, 0053phe, 0054hex, 0055ocr, 0056mcr, 0057pcr, 0058hep, 0145pro, 0146met, 0236oct.

Aldehydes and ketones: 0070eth, 0071proa, 0072but, 0073pen, 0074ben, 0145pro, 0150mhy, 0151phy, 0237oct, test2001, 0075pro, 0076but, 0077cyc, 0078pen, 0079pen, 0080hex, 0081dim, 0082hep, 0083hep, 0084met, 0085non, 0239oct.

Ethers: 0060dim, 0063die, 0064met, 0065met, 0067but, 0242dii, 0246eth, 0062dio, 0068ani, 0061tet.

Esters: 0091met, 0092ethb, 0093met, 0094met, 0095eth, 0096met, 0097pro, 0098met, 0099but, 0100met, 0101pen, 0238met, 0240met, test0011, test0013, test0016, test2003, test2011, test2020, test2026, test0013.

Acids: test2021, 0086eth, 0087pro, 0088but, 0089pen, 0090hex, test2001, test3007, test3014, test3015.

Amines: 0103eth, 0104dim, 0105aze, 0106pro, 0107tri, 0108pyr, 0109pip, 0110but, 0111die, 0112Nme, 0113pen, 0114NNd, 0115dip, 0116pyr, 0117met, 0118ani, 0119met, 0120met, 0121met, 0122Nme, 0123dim, 0124dim, 0125dim, 0216amm, 0225pipa, 0228met, 0229hyd, 0230eth, 0402adn, 0471dim, 0571dim, 0574eth, n005, n006, n009, n010, n011, n013, n014, n015, n016, test0017.

Nitriles: 0126eth, 0127pro, 0128butb, 0129ben.

Nitro compounds and nitrates: 0130nit, 0131nit, 0132nit, 0133nit, 0134nit, 0135met, 0506nit, test1001, test1002, test1028, test1041, test1058, test2022, test1003, test1004, test1005, test1006.

Fluorine compounds: 0153flu, 0154dif, 0157flu, 0197bro, 0198chl, 0199chl, 0200tet, 0201bro, 0203bro, 0205chl, 0206tri, 0207tri, 0209chl, 0211tri, 0212hex, 0214tri, 0405hex, 0406oct, 0421dfl, 0422ftc, 0424clp, n200, n201, test0004, test1011, test1027, test1046, test1056, test2010, test2013, test2023, test2029, test3021.

Chlorine compounds: 0160chl, 0161dic, 0162tri, 0163chl, 0165tri, 0166tri, 0167chla, 0168chl, 0169chl, 0170chl, 0171Zdi, 0172Edi, 0173tri, 0174chl, 0175odi, 0176pdi, 0198chl, 0199chl, 0201bro, 0202bro, 0204tet, 0205chl, 0206tri, 0209chl, 0213bis, 0407tet, 0408hex, 0409clb, 0410clp, 0411chp, 0412clt, 0413clt, 0414dcl, 0415dcl, 0416dcl, 0421dfl, 0422ftc, 0423brt, 0424clp, 0426dcl, 0427dcl, 0428ami, 0433pho, 0438pho, 0440pho, 0442pho, 0444pho, 0445pho, n202, test0007, test1007, test1014, test1017, test1018, test1019, test1020, test1021, test1022, test1023, test1025, test1029, test1030, test1033, test1035, test1048, test1049, test1050, test1052, test1055, test2006, test2015, test2024, test3019, test3020.

Bromine compounds: 0177bro, 0178dib, 0179tri, 0180bro, 0182bro, 0183bro, 0184bro, 0185bro, 0186bro, 0187dib, 0197bro, 0201bro, 0202bro, 0203bro, 0215pbr, 0417brp, 0418bri, 0419brt, 0420pbr, 0423brt, 0425dbr, 0442pho, 0445pho, n203, test1013.

Iodine compounds: 0939tet, test2018, test4001, test4002, test4003, test4004, test4006, test4007, test4008, test4009.

Table S3. MSE, MAE, and RMSE of the solvation free energy in kcal/mol for 14 *polar protic* solvents (MNSol database[1]) computed using the ESE-GB-DNN model in comparison with DFT-based uESE and SMD as well as with semi-empirical ESE-PM7 and ESE-EE (a total of 467 entries).

**Solvent**[a]	**uESE**			**SMD**			**ESE-PM7**			**ESE–EE**			**ESE-GB-DNN**		
	MSE	*MAE*	*RMSE*	*MSE*	*MAE*	*RMSE*	*MSE*	*MAE*	*RMSE*	*MSE*	*MAE*	*RMSE*	*MSE*	*MAE*	*RMSE*
octanol(247)	-0.047	0.820	1.097	0.62	1.24	1.72	–0.03	1.02	1.40	–0.12	1.18	1.61	–0.047	0.820	1.097
heptanol(12)	0.772	0.825	0.949	0.72	0.74	1.03	0.20	0.85	0.95	0.03	0.71	0.88	0.772	0.825	0.949
m-cresol(7)	0.949	0.988	1.190	1.56	1.56	1.76	–0.09	1.13	1.33	0.55	1.16	1.37	0.949	0.988	1.190
benzyl alcohol(10)	0.450	0.526	0.652	0.42	0.66	0.87	–0.34	0.67	1.00	–0.11	0.54	0.79	0.450	0.526	0.652
hexanol(14)	0.754	0.814	0.940	0.69	0.77	1.04	0.12	0.82	0.93	–0.06	0.64	0.78	0.754	0.814	0.940
pentanol(22)	0.777	0.935	1.065	0.40	0.72	0.90	0.21	0.97	1.17	–0.27	0.84	1.11	0.777	0.935	1.065
sec-butanol(9)	0.510	0.586	0.713	–0.23	0.53	0.72	–0.16	0.39	0.55	–0.18	0.44	0.58	0.510	0.586	0.713
isobutanol(17)	0.843	1.048	1.250	–0.01	0.56	0.68	0.60	0.75	1.00	–0.28	0.62	0.72	0.843	1.048	1.250
methoxyethanol(6)	0.083	0.494	0.565	0.20	0.83	0.94	–0.99	0.99	1.21	–0.34	0.45	0.75	0.083	0.494	0.565
butanol(21)	0.754	0.963	1.121	0.12	0.64	0.89	0.07	0.94	1.33	0.05	0.89	1.40	0.754	0.963	1.121
isopropanol(7)	0.006	0.719	0.791	–0.91	1.02	1.22	–1.03	1.03	1.53	–0.44	0.80	1.17	0.006	0.719	0.791
propanol(7)	0.060	0.666	0.763	–0.66	0.81	1.02	–0.97	0.99	1.50	–0.36	0.82	1.15	0.060	0.666	0.763
ethanol(8)	-0.587	0.859	1.080	–1.21	1.29	1.77	–1.33	1.33	1.65	–1.01	1.21	1.60	–0.587	0.859	1.080
methanol – cations(29)	-0.518	0.825	1.128	–0.32	2.44	2.94	0.14	2.18	2.86	0.93	5.77	6.49	–0.518	0.825	1.128
methanol – anions(51)	0.277	0.637	0.852	3.50	3.70	4.49	0.09	1.69	2.27	–0.19	3.21	4.38	0.277	0.637	0.852
methanol – all ions(80)	-0.011	0.705	0.961	2.11	3.24	4.00	0.11	1.87	2.50	0.22	4.14	5.25	–0.011	0.705	0.961
All polar protic solvents (467)	0.143	0.805	1.046	0.74	1.44	2.15	–0.03	1.13	1.59	–0.08	1.56	2.54	0.143	0.805	1.056
Slope		1.002			0.966			0.995			0.984			1.002	
Intercept		0.17			0.18			–0.12			–0.34			0.17	
R^2		0.998			0.994			0.995			0.988			0.998	

[a] The number of entries in the dataset is given in parentheses.

Table S4. MSE, MAE, and RMSE of the solvation free energy in kcal/mol for 20 polar aprotic solvents (MNSol database[1]) computed using the ESE-GB-DNN model in comparison with DFT-based uESE and SMD as well as with semi-empirical ESE-PM7 and ESE-EE (a total of 338 entries).

**Solvent**[a]	**ESE-GB-DNN**			**uESE**	**SMD**	**ESE-PM7**	**ESE-EE**
	MSE	*MAE*	*RMSE*	*RMSE*	*RMSE*	*RMSE*	*RMSE*
bromoethane(7)	0.071	0.472	0.560	0.750	0.940	1.05	1.43
2-methylpyridine(6)	0.335	0.447	0.636	0.753	0.856	0.71	1.12
o-dichlorobenzene(11)	–0.672	0.830	0.878	0.443	0.924	1.05	1.24
dichloroethane(39)	–0.367	0.478	0.580	0.770	0.644	0.77	1.32
4-methyl-2-pentanone (MIBK) (13)	0.327	0.652	0.900	1.134	0.930	1.21	1.30
pyridine(7)	0.388	0.476	0.640	0.699	0.856	0.91	1.02
cyclohexanone(10)	0.612	0.760	1.177	1.431	1.076	1.28	1.05
acetophenone(9)	0.402	0.473	0.626	0.906	0.777	0.87	0.94
butanone(13)	0.143	0.587	0.654	1.115	1.566	1.16	1.01
benzonitrile (PhCN) (7)	0.473	0.515	0.577	0.677	0.977	1.13	0.88
o-nitrotoluene(6)	–0.411	0.807	0.907	0.241	0.561	0.60	0.69
nitroethane(7)	0.220	0.267	0.398	0.372	0.698	0.84	0.80
nitrobenzene(15)	–0.111	0.669	0.750	0.323	0.744	0.73	0.85
acetonitrile (MeCN)							
neutral solutes (7)	0.077	0.419	0.443	0.995	0.933	1.21	1.35
cations(39)	0.044	0.368	0.456	2.409	10.450	4.01	6.17
anions(30)	–0.515	0.584	0.808	2.500	3.466	1.96	3.87
all ions(69)	–0.061	0.412	0.543	2.449	8.182	3.28	5.30
nitromethane ($MeNO_2$)(7)	0.082	0.259	0.354	0.739	1.244	0.94	0.87
dimethyl formamide (DMF) (7)	0.533	0.626	0.781	0.749	0.860	0.90	0.84
dimethyl acetamide (DMA) (7)	0.542	0.620	0.801	0.818	0.940	0.89	0.87
sulfolane(7)	0.069	0.520	0.646	0.653	1.642	1.04	1.03
dimethyl sulfoxide (DMSO)	0.350	0.877	0.952				
neutral solutes (7)				0.935	1.037	2.59	1.90
cations (4)	–0.386	0.386	0.529	2.577	8.606	2.53	5.78
anions (66)	–0.226	0.348	0.466	2.711	4.410	3.95	6.37
methyl formamide (NMF)(7)	0.585	0.759	1.025	1.017	0.939	1.15	1.73
**All polar *aprotic(338)***	–0.043	0.504	0.667	1.774	4.345	2.45	3.86
Slope	1.004	1.000		1.001	0.946	1.004	0.984
Intercept	0.07	0.06		0.05	–1.20	0.02	–0.34
R^2	0.9995	0.999		0.996	0.977	0.993	0.988

[a] The number of entries in the dataset is given in parentheses.

Table S5. MSE, MAE, and RMSE of the solvation free energy in kcal/mol for 57 nonpolar solvents (MNSol database[1]) computed using the ESE-GB-DNN model in comparison with DFT-based uESE and SMD as well as with semi-empirical ESE-PM7 and ESE-EE (a total of 1554 entries).

**Solvent**[a]	**ESE-GB-DNN**			**uESE**	**SMD**	**ESE-PM7**	**ESE-EE**
	MSE	*MAE*	*RMSE*	*RMSE*	*RMSE*	*RMSE*	*RMSE*
acetic acid(7)	0.410	0.560	0.774	0.585	2.577	0.98	1.46
aniline(10)	0.616	0.806	1.026	0.918	0.938	1.23	1.54
anisole(8)	0.319	0.337	0.435	0.351	0.631	0.75	0.67
benzene(75)	0.551	0.679	0.812	0.866	1.126	1.05	1.13
bromobenzene(27)	-0.167	0.328	0.391	0.489	0.635	0.70	0.38
bromoform(12)	-0.385	0.385	0.420	0.288	0.780	0.44	0.28
bromooctane(5)	-0.652	0.652	0.664	0.213	0.903	0.32	0.10
butyl acetate(22)	0.664	0.772	0.971	0.728	1.400	0.92	0.79
butylbenzene(10)	0.445	0.445	0.484	0.318	0.622	0.45	0.27
carbon disulfide(15)	-0.465	0.554	0.637	0.586	0.877	1.16	0.85
carbon tetrachloride(79)	0.131	0.400	0.527	0.490	0.728	0.60	0.78
chlorobenzene(38)	-0.208	0.363	0.551	0.503	0.793	0.66	0.51
chloroform(109)	0.253	0.806	1.051	0.917	1.094	1.15	1.31
chlorohexane(11)	-0.590	0.590	0.642	0.227	1.199	0.40	0.27
cyclohexane(92)	-0.224	0.463	0.627	0.656	0.787	0.68	1.03
decalin(27)	-0.185	0.343	0.412	0.432	0.878	0.51	0.52
decane(39)	0.040	0.227	0.295	0.482	0.515	0.47	0.57
decanol(11)	0.765	0.774	0.942	0.680	1.475	1.00	0.71
dibromoethane(10)	-0.134	0.283	0.356	0.446	0.792	0.47	0.21
dibutyl ether(15)	0.255	0.416	0.536	0.754	0.859	0.86	0.50
diethyl ether(72)	0.271	0.678	0.917	1.004	1.136	1.13	1.30
diisopropyl ether(22)	0.492	0.696	0.929	1.070	1.052	1.23	0.85
dimethylpyridine(6)	0.430	0.507	0.666	0.715	0.878	0.62	1.06
dodecane(8)	0.112	0.229	0.320	0.405	0.445	0.21	0.41
ethoxybenzene(7)	0.344	0.344	0.427	0.439	0.531	0.74	0.59
ethyl acetate(24)	0.687	0.784	1.000	1.129	1.362	1.34	1.59
ethylbenzene(29)	0.196	0.395	0.543	0.403	0.602	0.46	0.49
fluorobenzene(7)	0.293	0.304	0.397	0.584	0.950	0.96	0.67
fluoroctane(6)	-0.387	0.387	0.405	0.071	0.580	0.18	0.12
heptane(69)	-0.056	0.356	0.477	0.552	0.862	0.60	0.75
hexadecane(198)	0.064	0.485	0.679	0.645	0.998	0.71	0.95
hexadecyl iodide(9)	0.143	0.303	0.339	0.256	0.483	0.22	0.60
hexane(59)	-0.192	0.334	0.448	0.519	0.740	0.65	0.86
iodobenzene(20)	-0.164	0.291	0.407	0.542	0.486	0.75	0.47
isooctane(32)	-0.057	0.271	0.336	0.482	0.558	0.55	0.61
isopropylbenzene(19)	0.118	0.415	0.581	0.339	0.495	0.46	0.59
isopropyltoluene(6)	0.315	0.323	0.368	0.323	0.570	0.17	0.16
mesitylene(7)	0.613	0.613	0.650	0.366	0.664	0.50	0.30
methylene chloride(11)	-0.124	0.543	0.865	0.792	0.825	1.14	0.77
nonane(26)	-0.063	0.197	0.222	0.302	0.430	0.22	0.39
nonanol(10)	0.539	0.618	0.778	0.877	0.990	1.44	1.22
octane(38)	-0.135	0.228	0.303	0.408	0.516	0.50	0.50
pentadecane(9)	0.255	0.292	0.464	0.369	0.716	0.16	0.55
pentane(26)	-0.313	0.347	0.401	0.385	0.417	0.50	0.43
perfluorobenzene(15)	1.141	1.141	1.171	0.414	0.932	0.46	0.40
phenyl ether(6)	0.327	0.383	0.445	0.395	1.225	0.76	0.66
sec-butylbenzene(5)	0.328	0.328	0.344	0.212	0.403	0.21	0.17
tert-butylbenzene(14)	0.370	0.370	0.396	0.338	0.466	0.44	0.26
tetrachloroethene(10)	0.089	0.252	0.347	0.355	0.944	0.21	0.26
tetrahydrofuran(7)	0.536	0.579	0.717	0.681	0.865	0.97	0.81
tetralin(9)	-0.717	0.833	1.403	1.029	1.434	1.17	1.19
toluene(51)	0.335	0.452	0.559	0.395	0.728	0.52	0.58
tributyl phosphate(16)	0.851	1.082	1.174	0.688	0.618	0.52	0.89
triethylamine(7)	0.431	0.443	0.628	0.676	1.121	0.82	0.70
trimethylbenzene(11)	0.361	0.361	0.453	0.261	0.558	0.28	0.32
undecane(13)	0.318	0.418	0.480	0.414	0.650	0.46	0.56
xylene(48)	0.349	0.463	0.602	0.463	0.749	0.53	0.51
**All non–polar** ***(1554)***	0.127	0.490	0.677	0.642	0.897	0.77	0.87
Slope		0.869		0.908	0.792	0.917	0.855
Intercept		–0.53		-0.468	–0.828	–0.409	-0.727
R^2		0.892		0.899	0.815	0.859	0.818

Part 3. Solvation free energies in kcal/mol. Individual results
Table S6. Aqueous solutions

Solute MNSol code[1]	Solute formula	Solvent	$\Delta^{ref}G^{\circ}_{solv}$	$\Delta^{calc}G^{\circ}_{solv}$
0001met	CH_4	water	2.00	1.14
0002eth	C_2H_6	water	1.83	1.09
0003pro	C_3H_8	water	1.96	1.10
0004nbu	C_4H_{10}	water	2.08	1.06
0005npe	C_5H_{12}	water	2.33	1.14
0006nhe	C_6H_{14}	water	2.49	1.41
0007nhe	C_7H_{16}	water	2.62	1.54
0008noc	C_8H_{18}	water	2.89	1.65
0010met	C_4H_{10}	water	2.32	1.09
0011dim	C_5H_{12}	water	2.50	1.38
0012met	C_6H_{14}	water	2.52	1.42
0013dim	C_7H_{16}	water	2.88	1.67
0014tri	C_8H_{18}	water	2.85	1.55
0016cyc	C_3H_6	water	0.75	0.07
0017cyc	C_5H_{10}	water	1.20	0.00
0018cyc	C_6H_{12}	water	1.23	0.35
0019met	C_7H_{14}	water	1.71	0.98
0020cis	C_8H_{16}	water	1.58	1.36
0021eth	C_2H_4	water	1.27	0.78
0022pro	C_3H_6	water	1.27	0.84
0023str	C_4H_6	water	0.61	0.32
0024met	C_4H_8	water	1.16	0.86
0025buta	C_4H_8	water	1.38	0.84
0026cyc	C_5H_8	water	0.56	-0.18
0027pen	C_5H_{10}	water	1.66	0.74
0028Epe	C_5H_{10}	water	1.34	0.72
0029hex	C_6H_{12}	water	1.68	0.59
0030eth	C_2H_2	water	-0.01	0.09
0031pro	C_3H_4	water	-0.31	0.42
0032but	C_4H_6	water	-0.16	0.35
0033pen	C_5H_8	water	0.01	0.19
0034hex	C_6H_{10}	water	0.29	-0.01
0035ben	C_6H_6	water	-0.87	-0.49
0036tol	C_7H_8	water	-0.89	-0.61
0037eth	C_8H_{10}	water	-0.80	-0.81
0038oxy	C_8H_{10}	water	-0.90	-0.86
0039mxy	C_8H_{10}	water	-0.84	-0.78
0040pxy	C_8H_{10}	water	-0.81	-0.78
0041nap	$C_{10}H_8$	water	-2.39	-2.19
0042ant	$C_{14}H_{10}$	water	-4.23	-3.65
0044met	CH_4O	water	-5.11	-4.44
0045eth	C_2H_6O	water	-5.01	-4.42
0046eth	$C_2H_6O_2$	water	-9.30	-10.27
0047pro	C_3H_8O	water	-4.83	-4.38
0048pro	C_3H_8O	water	-4.76	-4.58
0049but	$C_4H_{10}O$	water	-4.72	-4.43
0050met	$C_4H_{10}O$	water	-4.51	-4.40
0051cyc	$C_5H_{10}O$	water	-5.49	-5.69
0052pen	$C_5H_{12}O$	water	-4.47	-4.61
0053phe	C_6H_6O	water	-6.62	-5.82
0054hex	$C_6H_{14}O$	water	-4.36	-4.46
0055ocr	C_7H_8O	water	-5.87	-6.04
0056mcr	C_7H_8O	water	-5.49	-5.95
0057pcr	C_7H_8O	water	-6.14	-5.97
0058hep	$C_7H_{16}O$	water	-4.24	-4.22
0060dim	C_2H_6O	water	-1.92	-1.01
0061tet	C_4H_8O	water	-3.47	-2.79
0062dio	$C_4H_8O_2$	water	-5.05	-5.31
0063die	$C_4H_{10}O$	water	-1.76	-1.62
0064met	$C_4H_{10}O$	water	-1.66	-1.47
0065met	$C_4H_{10}O$	water	-2.01	-1.80
0066dim	$C_4H_{10}O_2$	water	-4.84	-5.01
0067but	$C_5H_{12}O$	water	-2.21	-1.46
0068ani	C_7H_8O	water	-2.45	-2.29
0070eth	C_2H_4O	water	-3.50	-2.30
0071proa	C_3H_6O	water	-3.44	-2.49
0072but	C_4H_8O	water	-3.18	-2.79
0073pen	$C_5H_{10}O$	water	-3.03	-3.11
0074ben	C_7H_6O	water	-4.02	-4.36
0075pro	C_3H_6O	water	-3.85	-2.72

0076but	C_4H_8O	water	-3.64	-2.98
0077cyc	C_5H_8O	water	-4.68	-4.67
0078pen	$C_5H_{10}O$	water	-3.53	-2.97
0079pen	$C_5H_{10}O$	water	-3.41	-2.95
0080hex	$C_6H_{12}O$	water	-3.29	-2.90
0081dim	$C_6H_{12}O$	water	-2.89	-2.19
0082hep	$C_7H_{14}O$	water	-3.04	-2.81
0083hep	$C_7H_{14}O$	water	-2.93	-2.73
0084met	C_8H_8O	water	-4.58	-4.82
0085non	$C_9H_{18}O$	water	-2.67	-2.48
0086eth	$C_2H_4O_2$	water	-6.70	-7.46
0087pro	$C_3H_6O_2$	water	-6.47	-6.30
0088but	$C_4H_8O_2$	water	-6.36	-5.88
0089pen	$C_5H_{10}O_2$	water	-6.16	-5.56
0090hex	$C_6H_{12}O_2$	water	-6.21	-5.42
0091met	$C_2H_4O_2$	water	-2.78	-3.39
0092ethb	$C_3H_6O_2$	water	-2.65	-3.21
0093met	$C_3H_6O_2$	water	-3.32	-3.83
0094met	$C_4H_8O_2$	water	-2.93	-3.21
0095eth	$C_4H_8O_2$	water	-3.10	-3.61
0096met	$C_5H_{10}O_2$	water	-2.83	-3.13
0097pro	$C_5H_{10}O_2$	water	-2.86	-3.58
0098met	$C_6H_{12}O_2$	water	-2.57	-3.02
0099but	$C_6H_{12}O_2$	water	-2.55	-3.51
0100met	$C_7H_{14}O_2$	water	-2.49	-2.93
0101pen	$C_7H_{14}O_2$	water	-2.45	-3.42
0103eth	C_2H_7N	water	-4.50	-4.94
0104dim	C_2H_7N	water	-4.29	-3.33
0105aze	C_3H_7N	water	-5.56	-4.04
0106pro	C_3H_9N	water	-4.39	-4.77
0107tri	C_3H_9N	water	-3.23	-2.01
0108pyr	C_4H_9N	water	-5.48	-3.61
0109pip	$C_4H_{10}N_2$	water	-7.40	-6.72
0110but	$C_4H_{11}N$	water	-4.29	-4.57
0111die	$C_4H_{11}N$	water	-4.07	-2.93
0112Nme	$C_5H_{12}N_2$	water	-7.77	-5.99
0113pen	$C_5H_{13}N$	water	-4.10	-4.38
0114NNd	$C_6H_{14}N_2$	water	-7.58	-5.71
0115dip	$C_6H_{15}N$	water	-3.66	-3.10
0116pyr	C_5H_5N	water	-4.70	-4.31
0117met	$C_5H_6N_2$	water	-5.57	-7.24
0118ani	C_6H_7N	water	-5.49	-6.67
0119met	C_6H_7N	water	-4.63	-4.70
0120met	C_6H_7N	water	-4.77	-4.59
0121met	C_6H_7N	water	-4.94	-4.62
0122Nme	C_7H_9N	water	-4.68	-4.47
0123dim	C_7H_9N	water	-4.86	-4.44
0124dim	C_7H_9N	water	-4.72	-4.45
0125dim	C_7H_9N	water	-4.60	-4.42
0126eth	C_2H_3N	water	-3.89	-3.42
0127pro	C_3H_5N	water	-3.85	-3.61
0128butb	C_4H_7N	water	-3.64	-3.70
0129ben	C_7H_5N	water	-4.10	-4.34
0130nit	$C_2H_5NO_2$	water	-3.71	-3.26
0131nit	$C_3H_7NO_2$	water	-3.34	-2.99
0132nit	$C_3H_7NO_2$	water	-3.14	-3.37
0133nit	$C_4H_9NO_2$	water	-3.08	-3.16
0134nit	$C_6H_5NO_2$	water	-4.12	-5.09
0135met	$C_7H_7NO_2$	water	-3.59	-5.14
0136met	CH_4S	water	-1.24	-0.69
0137ethb	C_2H_6S	water	-1.30	-0.99
0138pro	C_3H_8S	water	-1.05	-1.23
0139thi	C_6H_6S	water	-2.55	-2.16
0140dim	C_2H_6S	water	-1.54	-0.65
0141dim	$C_2H_6S_2$	water	-1.83	-1.61
0142die	$C_4H_{10}S$	water	-1.43	-0.85
0143dip	$C_6H_{14}S$	water	-1.27	-0.32
0144thi	C_7H_8S	water	-2.73	-2.11
0145pro	C_3H_6O	water	-5.08	-5.43
0146met	$C_3H_8O_2$	water	-6.77	-6.71
0147met	C_3H_9NO	water	-6.55	-7.61
0148but	C_4H_4	water	0.04	-0.30
0149mor	C_4H_9NO	water	-7.17	-6.98

0150mhy	$C_7H_6O_2$	water	-9.51	-9.09
0151phy	$C_7H_6O_2$	water	-10.48	-9.12
0153flu	CH_3F	water	-0.22	0.38
0154dif	$C_2H_4F_2$	water	-0.11	0.20
0157flu	C_6H_5F	water	-0.78	-0.68
0160chl	CH_3Cl	water	-0.56	-0.08
0161dic	CH_2Cl_2	water	-1.36	-0.37
0162tri	$CHCl_3$	water	-1.07	-0.47
0163chl	C_2H_5Cl	water	-0.63	-0.32
0165tri	$C_2H_3Cl_3$	water	-0.25	-0.91
0166tri	$C_2H_3Cl_3$	water	-1.95	-1.90
0167chla	C_3H_7Cl	water	-0.27	-0.72
0168chl	C_3H_7Cl	water	-0.25	-0.93
0169chl	C_2H_3Cl	water	-0.59	-0.07
0170chl	C_3H_5Cl	water	-0.57	-0.46
0171Zdi	$C_2H_2Cl_2$	water	-1.17	-0.33
0172Edi	$C_2H_2Cl_2$	water	-0.76	-0.50
0173tri	C_2HCl_3	water	-0.39	-0.48
0174chl	C_6H_5Cl	water	-1.12	-1.17
0175odi	$C_6H_4Cl_2$	water	-1.36	-1.58
0176pdi	$C_6H_4Cl_2$	water	-1.01	-1.79
0177bro	CH_3Br	water	-0.82	-0.62
0178dib	CH_2Br_2	water	-2.11	-2.11
0179tri	$CHBr_3$	water	-1.98	-2.83
0180bro	C_2H_5Br	water	-0.70	-0.74
0182bro	C_3H_7Br	water	-0.56	-0.87
0183bro	C_3H_7Br	water	-0.48	-0.81
0184bro	C_4H_9Br	water	-0.41	-0.74
0185bro	$C_5H_{11}Br$	water	-0.08	-0.57
0186bro	C_6H_5Br	water	-1.46	-2.05
0187dib	$C_6H_4Br_2$	water	-2.30	-3.00
0197bro	CF_3Br	water	1.79	1.21
0198chl	CH_2FCl	water	-0.77	-0.25
0199chl	CH_1F_2Cl	water	-0.50	0.70
0200tet	CF_4	water	3.16	2.87
0201bro	$C_2H_1F_3ClBr$	water	-0.13	0.28
0202bro	C_2H_4ClBr	water	-1.95	-2.05
0203bro	$C_2H_1F_4Br$	water	0.52	0.87
0204tet	C_2Cl_4	water	0.05	-0.49
0205chl	$C_2H_2F_3Cl$	water	0.06	0.75
0206tri	$C_2F_3Cl_3$	water	1.77	0.83
0207tri	$C_2H_3OF_3$	water	-4.31	-2.86
0209chl	$C_3H_2OF_5Cl$	water	0.11	0.91
0211tri	$C_3H_5OF_3$	water	-4.16	-3.46
0212hex	$C_3H_2OF_6$	water	-3.77	-3.24
0213bis	$C_4H_8SCl_2$	water	-3.92	-3.13
0214tri	$C_4H_5OF_3$	water	-0.12	0.41
0215pbr	C_6H_5OBr	water	-7.13	-6.27
0216amm	H_3N	water	-4.29	-5.96
0217wat	H_2O	water	-6.31	-9.35
0218pho	H_3P	water	0.60	-1.03
0219hyd	H_2S	water	-0.70	-0.75
0220tri	$C_3H_9O_4P$	water	-8.70	-10.48
0221tri	$C_6H_{15}O_4P$	water	-7.80	-7.65
0222tri	$C_9H_{21}O_4P$	water	-6.10	-5.36
0223die	$C_4H_{10}S_2$	water	-1.63	-1.54
0225pipa	$C_5H_{11}N$	water	-5.11	-3.59
0227Nme	$C_5H_{11}NO$	water	-6.34	-6.76
0228met	CH_5N	water	-4.56	-4.48
0229hyd	H_4N_2	water	-6.26	-8.26
0230eth	$C_6H_8N_2$	water	-5.51	-6.64
0233ethb	C_2H_5NO	water	-9.71	-9.28
0234ENmb	C_3H_7NO	water	-10.00	-7.02
0235ZNmb	C_3H_7NO	water	-10.00	-6.82
0236oct	$C_8H_{18}O$	water	-4.09	-3.86
0237oct	$C_8H_{16}O$	water	-2.29	-2.93
0238met	$C_9H_{18}O_2$	water	-2.04	-2.65
0239oct	$C_8H_{16}O$	water	-2.88	-2.71
0240met	$C_8H_8O_2$	water	-3.91	-5.16
0242dii	$C_6H_{14}O$	water	-0.53	-1.55
0244tet	$C_5H_{10}O$	water	-3.12	-2.91
0245thi	C_4H_4S	water	-1.42	-1.15
0246eth	$C_8H_{10}O$	water	-2.22	-2.46

0400hyd	H_2	water	2.33	-0.74
0401amia	$C_9H_{12}N_2O$	water	-9.63	-9.12
0402adn	$C_6H_7N_5$	water	-13.60	-17.16
0403thi	$C_6H_8N_2O_2$	water	-10.40	-11.19
0405hex	C_2F_6	water	3.94	3.51
0406oct	C_3F_8	water	4.28	3.97
0407tet	$C_2H_2Cl_4$	water	-1.15	-1.26
0408hex	C_2Cl_6	water	-1.40	-1.05
0409clb	C_4H_9Cl	water	0.07	-0.90
0410clp	$C_5H_{11}Cl$	water	0.07	-0.64
0411chp	$C_5H_{11}Cl$	water	0.07	-0.69
0412clt	C_7H_7Cl	water	-1.92	-1.60
0413clt	C_7H_7Cl	water	-1.15	-1.56
0414dcl	$C_{12}H_8Cl_2$	water	-2.73	-2.58
0415dcl	$C_{12}H_8Cl_2$	water	-2.45	-2.93
0416dcl	$C_{12}H_7Cl_3$	water	-1.99	-2.96
0417brp	C_3H_5Br	water	-0.86	-0.97
0418bri	C_4H_9Br	water	-0.03	-0.67
0419brt	C_7H_7Br	water	-2.37	-2.24
0420pbr	C_7H_7Br	water	-1.39	-1.87
0421dfl	CF_2Cl_2	water	1.69	1.15
0422ftc	$CFCl_3$	water	0.82	0.31
0423brt	C_1Cl_3Br	water	-0.93	-1.21
0424clp	C_2F_5Cl	water	2.86	2.75
0425dbr	$C_7H_3NOBr_2$	water	-9.00	-5.14
0426dcl	$C_7H_3NCl_2$	water	-5.22	-4.28
0427dcl	$C_7H_5NSCl_2$	water	-10.81	-8.97
0428ami	$C_6H_3N_2O_2Cl_3$	water	-11.96	-12.48
0433pho	$C_4H_7O_4PCl_2$	water	-6.61	-7.64
0437pho	$C_9H_{13}O_3PS_2$	water	-6.92	-4.77
0438pho	$C_{10}H_{13}O_3PSCl_2$	water	-3.86	-3.56
0440pho	$C_9H_{12}O_4PCl$	water	-7.28	-7.94
0441pho	$C_8H_{10}NO_5PS$	water	-7.62	-7.17
0442pho	$C_{11}H_{15}O_3PSClBr$	water	-4.09	-1.32
0444pho	$C_8H_8O_3PSCl_3$	water	-5.06	-5.14
0445pho	$C_8H_8O_3PSCl_2Br$	water	-5.70	-5.35
0447pho	$C_{10}H_{14}NO_5PS$	water	-6.27	-5.86
0449pho	$C_{15}H_{14}NO_2PS$	water	-5.10	-5.39
0471dim	C_7H_9N	water	-5.22	-4.46
0506nit	CH_3NO_2	water	-3.95	-3.25
0571dim	C_7H_9N	water	-4.84	-4.49
0574eth	C_7H_9N	water	-4.74	-4.52
0939tet	$C_4H_{12}Si$	water	3.04	2.26
n005	CH_6N_2	water	-5.31	-5.53
n006	$C_2H_8N_2$	water	-4.48	-4.90
n007	CH_4N_2O	water	-13.80	-14.27
n008	C_7H_7NO	water	-10.90	-9.23
n009	C_7H_9N	water	-5.56	-5.56
n010	C_7H_9N	water	-5.67	-5.95
n011	C_7H_9N	water	-5.55	-5.97
n013	$C_8H_{11}N$	water	-4.62	-3.89
n014	$C_8H_{11}N$	water	-3.58	-3.20
n015	$C_6H_8N_2$	water	-9.92	-10.80
n016	$C_2H_8N_2$	water	-9.72	-9.99
n017	H_2O_2	water	-8.58	-10.93
n018	CH_4O_2	water	-5.28	-5.85
n019	$C_2H_6O_2$	water	-5.32	-5.65
n191	$C_4H_4N_2O_2$	water	-16.59	-13.92
n200	$C_4H_3N_2O_2F$	water	-16.92	-13.57
n201	$C_5H_3N_2O_2F_3$	water	-15.46	-14.44
n202	$C_4H_3N_2O_2Cl$	water	-17.74	-13.95
n203	$C_4H_3N_2O_2Br$	water	-18.17	-9.95
test0001	$C_9H_{14}O_6$	water	-8.84	-8.97
test0004	$C_8H_4F_6$	water	1.07	0.37
test0005	$C_{10}H_{13}NO_2$	water	-11.01	-10.76
test0006	$C_{10}H_{13}NO$	water	-9.76	-7.26
test0007	$C_4H_8OCl_2$	water	-4.23	-5.25
test0008	$C_6H_{10}O_4$	water	-4.97	-5.09
test0009	$C_6H_{14}O_2$	water	-3.28	-2.37
test0011	$C_7H_{12}O_4$	water	-6.00	-6.42
test0012	$C_3H_8O_2$	water	-2.93	-3.15
test0013	$C_6H_{10}O_4$	water	-6.34	-5.43
test0014	$C_6H_{14}O_2$	water	-3.54	-5.07

test0016	$C_7H_6O_2$	water	-3.82	-5.05
test0017	$C_3H_4N_2$	water	-9.81	-7.67
test1001	$C_2H_4N_2O_6$	water	-5.70	-5.22
test1002	$C_3H_6N_2O_6$	water	-5.00	-4.21
test1003	$C_4H_9NO_3$	water	-2.10	-1.93
test1004	$C_4H_9NO_3$	water	-1.80	-1.83
test1005	$C_4H_9NO_3$	water	-1.90	-1.96
test1006	$C_2H_5NO_4$	water	-8.20	-7.26
test1007	$C_{14}H_{20}NO_2Cl$	water	-8.20	-7.84
test1008	$C_7H_{14}N_2O_2S$	water	-9.80	-6.49
test1009	$C_9H_{17}N_5S$	water	-7.70	-8.26
test1010	$C_{10}H_{12}N_3O_3PS_2$	water	-10.00	-9.27
test1011	$C_{13}H_{16}N_3O_4F_3$	water	-3.50	-2.79
test1012	$C_{16}H_{18}N_4O_7S$	water	-17.20	-17.06
test1013	$C_9H_{13}N_2O_2Br$	water	-9.70	-9.68
test1014	$C_9H_8NO_2SCl_3$	water	-9.00	-8.26
test1015	$C_{12}H_{11}NO_2$	water	-9.50	-7.36
test1016	$C_{12}H_{15}NO_3$	water	-9.60	-8.30
test1017	$C_{11}H_{16}O_2PS_3Cl$	water	-6.50	-6.13
test1018	$C_{10}H_6Cl_8$	water	-3.40	-3.30
test1019	$C_{12}H_{14}O_4PCl_3$	water	-7.10	-4.38
test1020	$C_{15}H_{15}N_4O_6SCl$	water	-14.00	-14.00
test1021	CNO_2Cl_3	water	-1.50	-1.42
test1022	$C_9H_{11}NO_3PSCl_3$	water	-5.00	-5.20
test1023	$C_{14}H_{17}NO_4PS_2Cl$	water	-5.70	-6.00
test1024	$C_{12}H_{21}N_2O_3PS$	water	-6.50	-4.57
test1025	$C_8H_6O_3Cl_2$	water	-9.90	-7.05
test1027	$C_{11}H_{13}N_4O_4F_3$	water	-5.70	-5.56
test1028	$C_{10}H_{12}N_2O_5$	water	-6.20	-5.82
test1029	$C_9H_6O_3SCl_6$	water	-4.20	-5.21
test1030	$C_{12}H_8OCl_6$	water	-5.50	-5.57
test1031	$C_9H_{22}O_4P_2S_4$	water	-6.10	-5.75
test1033	$C_{10}H_5Cl_7$	water	-2.60	-3.15
test1034	$C_9H_{14}O$	water	-5.20	-4.58
test1035	$C_6H_6Cl_6$	water	-5.40	-5.69
test1036	$C_{10}H_{19}O_6PS_2$	water	-8.20	-6.61
test1037	$C_5H_{10}N_2O_2S$	water	-10.70	-7.20
test1039	$C_{14}H_{15}N_5O_6S$	water	-15.50	-15.38
test1040	$C_{13}H_{19}N_3O_6S$	water	-8.00	-8.27
test1041	$C_3H_5NO_4$	water	-6.00	-6.39
test1043	$C_{10}H_{14}NO_5PS$	water	-6.70	-5.86
test1044	$C_{10}H_{21}NOS$	water	-3.60	-3.74
test1045	$C_7H_{17}O_2PS_3$	water	-4.40	-4.67
test1046	$C_{14}H_{16}N_3O_4F_3$	water	-2.50	-4.43
test1047	$C_{10}H_{19}N_5S$	water	-8.40	-7.34
test1048	$C_9H_9NOCl_2$	water	-7.80	-8.33
test1049	$C_{10}H_8N_3OCl$	water	-16.40	-12.17
test1050	$C_7H_{12}N_5Cl$	water	-10.20	-9.70
test1051	$C_{15}H_{16}N_4O_5S$	water	-20.30	-12.56
test1052	$C_9H_{13}N_2O_2Cl$	water	-11.10	-10.42
test1053	$C_{10}H_{19}N_5S$	water	-6.70	-7.36
test1054	$C_{12}H_{13}N_5O_6S_2$	water	-16.20	-17.27
test1055	$C_4H_8O_4PCl_3$	water	-12.70	-9.40
test1056	$C_{13}H_{16}N_3O_4F_3$	water	-3.30	-2.94
test1057	$C_{10}H_{21}NOS$	water	-4.10	-3.92
test1058	$C_{12}H_{10}N_4O_2$	water	-11.20	-10.62
test1059	$C_{20}H_{14}N_2O_2$	water	-7.40	-9.94
test1060	$C_{14}H_{12}N_4O_2$	water	-8.90	-18.95
test1061	$C_{14}H_9NO_2$	water	-8.00	-10.63
test1063	$C_{11}H_{18}N_4O_2$	water	-9.40	-9.80
test2001	$C_9H_8O_4$	water	-9.94	-8.09
test2003	$C_{11}H_{14}O_3$	water	-8.72	-8.03
test2004	$C_8H_{10}N_4O_2$	water	-12.64	-15.28
test2006	$C_4H_3N_2O_2Cl$	water	-15.83	-15.38
test2007	$C_3H_3N_3O_3$	water	-18.06	-20.19
test2010	$C_{13}H_8O_3F_2$	water	-9.40	-9.03
test2011	$C_9H_{10}O_3$	water	-9.20	-8.71
test2013	$C_{15}H_{13}O_2F$	water	-8.42	-7.56
test2015	C_6Cl_6	water	-2.33	-2.06
test2017	$C_{13}H_{18}O_2$	water	-7.00	-7.24
test2018	$C_4H_3N_2O_2I$	water	-18.72	-17.78
test2019	$C_{16}H_{14}O_3$	water	-10.78	-10.27
test2020	$C_8H_8O_3$	water	-9.51	-9.26

test2021	$C_{14}H_{14}O_3$	water	-10.21	-10.52
test2022	$C_6H_6N_2O_2$	water	-9.45	-10.57
test2023	C_4F_8	water	3.43	3.41
test2024	$C_6NO_2Cl_5$	water	-5.22	-3.32
test2025	$C_8H_5NO_2$	water	-9.61	-10.46
test2026	$C_{10}H_{12}O_3$	water	-9.37	-8.39
test2027	$C_4H_8O_2S$	water	-8.61	-9.60
test2029	$C_5H_9O_3F_3$	water	-0.80	-0.75
test3001	$C_8H_9NO_2$	water	-14.83	-12.44
test3002	$C_8H_9NO_2$	water	-13.93	-12.39
test3003	$C_{16}H_{14}O_3$	water	-12.75	-11.22
test3004	$C_8H_9NO_2$	water	-11.61	-10.83
test3005	$C_{10}H_{13}NO_2$	water	-10.91	-11.33
test3007	$C_8H_8O_3$	water	-10.32	-6.46
test3014	$C_8H_8O_3$	water	-9.15	-9.00
test3015	$C_8H_8O_3$	water	-8.93	-8.64
test3019	$C_{14}H_{12}NO_2Cl$	water	-6.71	-7.58
test3020	$C_{14}H_{11}NO_2Cl_2$	water	-6.30	-7.20
test3021	$C_{14}H_{10}NO_2F_3$	water	-5.68	-6.92
test4001	C_6H_5I	water	-1.73	-2.53
test4002	CH_2I_2	water	-2.49	-2.32
test4003	CH_3I	water	-0.89	-1.40
test4004	C_2H_5I	water	-0.72	-1.14
test4006	C_3H_7I	water	-0.59	-0.82
test4007	C_4H_9I	water	-0.25	-0.52
test4008	$C_5H_{11}I$	water	-0.12	-0.25
test4009	C_3H_7I	water	-0.46	-0.79
c050	$CH_7O_2^+$	water	-76.60	-76.58
c051	$C_2H_9O_2^+$	water	-73.60	-73.70
c052	$C_2H_9O_2^+$	water	-66.30	-68.24
c053	$C_4H_{13}O_2^+$	water	-62.20	-63.03
c054	$C_3H_9O_2^+$	water	-66.30	-69.87
c056	$C_8H_{11}O_2^+$	water	-55.70	-57.38
c088	$H_5O_2^+$	water	-87.80	-88.53
i003	CH_6N^+	water	-76.40	-77.59
i004	$C_3H_{10}N^+$	water	-71.50	-72.68
i005	$C_3H_{10}N^+$	water	-69.60	-70.86
i006	$C_4H_{12}N^+$	water	-67.30	-66.53
i007	$C_6H_{14}N^+$	water	-68.70	-69.44
i008	$C_3H_8N^+$	water	-72.00	-74.11
i009	$C_2H_8N^+$	water	-68.60	-72.53
i010	$C_4H_{12}N^+$	water	-63.40	-65.99
i011	$C_6H_{16}N^+$	water	-60.50	-61.45
i012	$C_6H_{12}N^+$	water	-61.60	-63.44
i013	$C_3H_{10}N^+$	water	-61.10	-62.99
i014	$C_6H_{16}N^+$	water	-54.60	-59.54
i015	$C_9H_{22}N^+$	water	-50.90	-55.51
i018	$C_6H_8N^+$	water	-72.40	-69.99
i019	$C_7H_{10}N^+$	water	-70.30	-68.84
i020	$C_7H_{10}N^+$	water	-69.60	-69.29
i021	$C_7H_{10}N^+$	water	-69.80	-69.44
i023	$C_6H_9N_2^+$	water	-65.80	-69.79
i024	$C_7H_{10}N^+$	water	-62.60	-62.93
i025	$C_8H_{12}N^+$	water	-62.20	-62.29
i026	$C_8H_{12}N^+$	water	-57.20	-56.91
i027	$C_9H_{14}N^+$	water	-55.90	-56.82
i028	$C_{10}H_{16}N^+$	water	-54.00	-55.67
i029	$C_{10}H_{10}N^+$	water	-67.40	-65.64
i030	$C_2H_6N^+$	water	-70.90	-73.22
i031	$C_3H_8N^+$	water	-67.70	-69.31
i032	$C_4H_{10}N^+$	water	-66.00	-66.63
i033	$C_5H_{12}N^+$	water	-64.20	-65.70
i034	$C_6H_{14}N^+$	water	-63.30	-65.16
i035	$C_4H_6N^+$	water	-61.40	-64.11
i036	$C_5H_6N^+$	water	-61.10	-60.06
i037	$C_9H_8N^+$	water	-56.00	-55.72
i039	$C_4H_{11}N_2^+$	water	-66.00	-67.12
i040	$C_2H_4N^+$	water	-75.30	-75.77
i047	H_4N^+	water	-85.20	-87.59
i048	$H_5N_2^+$	water	-84.60	-85.66
i050	CH_5O^+	water	-93.00	-90.74
i051	$C_2H_7O^+$	water	-88.40	-82.92
i052	$C_2H_7O^+$	water	-79.70	-79.98

i053	$C_4H_{11}O^+$	water	-71.50	-66.22
i054	$C_3H_7O^+$	water	-77.10	-73.56
i056	$C_8H_9O^+$	water	-64.50	-55.58
i088	H_3O^+	water	-110.30	-110.83
i093	$C_7H_{10}NO^+$	water	-71.20	-71.27
i094	$C_6H_7N_2O_2^+$	water	-75.90	-73.93
i095	$C_4H_{10}NO^+$	water	-69.60	-69.89
i098	$C_2H_6NO^+$	water	-73.90	-82.06
i099	$C_7H_8NO^+$	water	-67.20	-66.99
i106	$C_2H_7S^+$	water	-64.50	-63.91
i112	$C_2H_7OS^+$	water	-67.70	-68.23
i125	$C_6H_7NCl^+$	water	-74.70	-74.59
i126	$C_6H_7NCl^+$	water	-74.10	-73.95
c001	$C_2H_3O^-$	water	-67.90	-70.05
c046b	CH_2NO^-	water	-64.00	-63.95
c065	$CH_5O_2^-$	water	-80.00	-80.95
c066	$C_2H_7O_2^-$	water	-78.50	-79.11
c067	$C_3H_9O_2^-$	water	-75.80	-77.26
c068	$C_3H_9O_2^-$	water	-76.00	-76.32
c069	$C_4H_{11}O_2^-$	water	-76.30	-76.17
c070	$C_4H_{11}O_2^-$	water	-72.10	-74.24
c071	$C_3H_7O_2^-$	water	-75.10	-75.22
c072	$C_7H_9O_2^-$	water	-75.40	-75.14
c073	$C_3H_9O_3^-$	water	-77.90	-77.05
c078	$C_2H_7O_3^-$	water	-73.30	-73.87
c082	$CH_5O_3^-$	water	-80.60	-78.54
c083	$C_2H_7O_3^-$	water	-77.10	-76.21
c089	$H_3O_2^-$	water	-86.90	-77.32
c091	$H_2O_3^-$	water	-73.20	-74.72
c114	H_2OF^-	water	-85.50	-85.92
c115	H_2OCl^-	water	-67.50	-60.01
c116	H_2OBr^-	water	-63.20	-60.05
c121	$C_2H_4O_2F_3^-$	water	-68.00	-73.71
c122	$C_3H_3O_2F_6^-$	water	-61.60	-66.64
i001	$C_2H_1^-$	water	-76.50	-77.22
i043	CHN_2^-	water	-72.20	-69.99
i044	$C_6H_6N^-$	water	-62.90	-62.80
i045	$C_{12}H_{10}N^-$	water	-54.60	-54.46
i046	CN^-	water	-70.20	-70.11
i058	CHO_2^-	water	-76.20	-78.64
i059	$C_2H_3O_2^-$	water	-77.60	-76.84
i060	$C_3H_5O_2^-$	water	-76.20	-76.79
i061	$C_6H_{11}O_2^-$	water	-74.60	-74.80
i062	$C_3H_3O_2^-$	water	-74.00	-73.76
i063	$C_3H_3O_3^-$	water	-68.50	-68.17
i064	$C_7H_5O_2^-$	water	-71.20	-69.79
i065	CH_3O^-	water	-95.00	-99.52
i066	$C_2H_5O^-$	water	-90.70	-91.38
i067	$C_3H_7O^-$	water	-88.30	-88.34
i068	$C_3H_7O^-$	water	-86.30	-85.07
i069	$C_4H_9O^-$	water	-84.20	-84.50
i070	$C_4H_9O^-$	water	-82.30	-77.60
i071	$C_3H_5O^-$	water	-86.60	-82.44
i072	$C_7H_7O^-$	water	-85.10	-79.67
i073	$C_3H_7O_2^-$	water	-89.40	-88.14
i074	$C_6H_5O^-$	water	-71.90	-73.60
i075	$C_7H_7O^-$	water	-70.20	-73.85
i076	$C_7H_7O^-$	water	-71.10	-72.74
i077	$C_7H_7O^-$	water	-72.00	-73.12
i078	$C_2H_5O_2^-$	water	-85.30	-86.43
i080	$C_6H_5O_2^-$	water	-73.80	-74.90
i081	$C_6H_5O_2^-$	water	-77.60	-75.39
i082	$CH_3O_2^-$	water	-93.20	-92.96
i083	$C_2H_5O_2^-$	water	-89.20	-88.84
i084	$C_2H_3O^-$	water	-76.50	-83.33
i085	$C_3H_5O^-$	water	-76.20	-77.88
i086	$C_5H_9O^-$	water	-73.70	-79.70
i089	HO^-	water	-104.70	-104.36
i090	HO_2^-	water	-97.30	-96.22
i091	O_2^-	water	-83.30	-97.85
i100	$C_6H_4NO_3^-$	water	-60.10	-60.17
i101	$C_6H_4NO_3^-$	water	-61.90	-60.53
i102	$C_6H_4NO_3^-$	water	-57.80	-61.09

i103	$CH_2NO_2^-$	water	-76.50	-78.28
i104	$C_6H_5N_2O_2^-$	water	-57.40	-56.83
i105	$C_2H_4NO^-$	water	-80.20	-76.59
i107	CH_3S^-	water	-73.80	-74.27
i108	$C_2H_5S^-$	water	-71.80	-72.65
i109	$C_3H_7S^-$	water	-70.50	-71.12
i110	$C_6H_5S^-$	water	-63.40	-63.24
i111	HS^-	water	-72.10	-72.19
i113	$C_2H_5OS^-$	water	-67.70	-69.76
i114	F^-	water	-104.40	-107.21
i115	Cl^-	water	-74.50	-77.73
i116	Br^-	water	-68.30	-68.81
i117	$C_1Cl_3^-$	water	-54.10	-54.74
i118	$C_2O_2F_3^-$	water	-59.30	-59.83
i119	$C_2H_2O_2Cl^-$	water	-69.70	-65.93
i120	$C_2HO_2Cl_2^-$	water	-62.30	-62.09
i121	$C_2H_2OF_3^-$	water	-77.50	-77.80
i122	$C_3HOF_6^-$	water	-65.50	-65.08
i123	$C_6H_4OCl^-$	water	-66.10	-66.41
i124	$C_6H_4OCl^-$	water	-66.00	-65.46

Table S7. Polar protic solvents

Solute MNSol code[1]	Solute formula	Solvent	$\Delta^{ref}G^{\circ}_{solv}$	$\Delta^{calc}G^{\circ}_{solv}$
0001met	CH_4	octanol	0.51	-1.31
0002eth	C_2H_6	octanol	-0.64	-1.31
0003pro	C_3H_8	octanol	-1.26	-1.33
0004nbu	C_4H_{10}	octanol	-1.86	-1.75
0005npe	C_5H_{12}	octanol	-2.45	-2.24
0006nhe	C_6H_{14}	octanol	-3.01	-2.80
0007nhe	C_7H_{16}	octanol	-3.74	-3.50
0008noc	C_8H_{18}	octanol	-4.18	-4.21
0010met	C_4H_{10}	octanol	-1.45	-1.56
0011dim	C_5H_{12}	octanol	-1.74	-1.55
0016cyc	C_3H_6	octanol	-1.60	-1.65
0017cyc	C_5H_{10}	octanol	-2.65	-3.53
0018cyc	C_6H_{12}	octanol	-3.46	-4.20
0019met	C_7H_{14}	octanol	-3.21	-4.64
0021eth	C_2H_4	octanol	-0.27	-1.31
0022pro	C_3H_6	octanol	-1.14	-1.32
0023str	C_4H_6	octanol	-2.10	-1.83
0024met	C_4H_8	octanol	-2.03	-1.59
0025buta	C_4H_8	octanol	-1.89	-1.75
0029hex	C_6H_{12}	octanol	-2.94	-3.02
0030eth	C_2H_2	octanol	-0.51	-1.31
0031pro	C_3H_4	octanol	-1.59	-1.58
0033pen	C_5H_8	octanol	-2.79	-2.82
0034hex	C_6H_{10}	octanol	-3.43	-3.61
0035ben	C_6H_6	octanol	-3.72	-3.67
0036tol	C_7H_8	octanol	-4.55	-4.23
0037eth	C_8H_{10}	octanol	-5.08	-4.91
0038oxy	C_8H_{10}	octanol	-5.07	-4.79
0039mxy	C_8H_{10}	octanol	-5.25	-4.76
0040pxy	C_8H_{10}	octanol	-5.19	-4.76
0041nap	$C_{10}H_8$	octanol	-6.97	-7.15
0042ant	$C_{14}H_{10}$	octanol	-10.47	-10.64
0044met	CH_4O	octanol	-3.87	-3.16
0045eth	C_2H_6O	octanol	-4.36	-3.53
0046eth	$C_2H_6O_2$	octanol	-7.44	-6.61
0047pro	C_3H_8O	octanol	-5.02	-4.17
0048pro	C_3H_8O	octanol	-4.62	-3.69
0049but	$C_4H_{10}O$	octanol	-5.71	-4.92
0050met	$C_4H_{10}O$	octanol	-4.78	-3.35
0052pen	$C_5H_{12}O$	octanol	-6.40	-5.75
0053phe	C_6H_6O	octanol	-8.69	-7.24
0054hex	$C_6H_{14}O$	octanol	-7.06	-6.38
0055ocr	C_7H_8O	octanol	-8.49	-7.54
0056mcr	C_7H_8O	octanol	-8.20	-7.57
0057pcr	C_7H_8O	octanol	-8.84	-7.59
0058hep	$C_7H_{16}O$	octanol	-7.75	-7.03
0059dec	$C_{10}H_{22}O$	octanol	-9.88	-8.94
0060dim	C_2H_6O	octanol	-2.06	-2.25
0061tet	C_4H_8O	octanol	-3.93	-4.12
0062dio	$C_4H_8O_2$	octanol	-4.89	-5.13
0063die	$C_4H_{10}O$	octanol	-2.89	-3.45
0064met	$C_4H_{10}O$	octanol	-3.63	-3.72
0065met	$C_4H_{10}O$	octanol	-4.64	-3.27
0066dim	$C_4H_{10}O_2$	octanol	-4.55	-5.04
0067but	$C_5H_{12}O$	octanol	-3.49	-3.54
0068ani	C_7H_8O	octanol	-5.47	-6.39

0069met	CH_2O	octanol	-3.23	-2.27
0071proa	C_3H_6O	octanol	-4.13	-4.06
0072but	C_4H_8O	octanol	-4.62	-4.90
0074ben	C_7H_6O	octanol	-6.13	-7.23
0075pro	C_3H_6O	octanol	-3.15	-3.62
0076but	C_4H_8O	octanol	-3.78	-4.23
0077cyc	C_5H_8O	octanol	-5.01	-5.67
0078pen	$C_5H_{10}O$	octanol	-4.35	-4.87
0079pen	$C_5H_{10}O$	octanol	-4.36	-4.80
0080hex	$C_6H_{12}O$	octanol	-5.02	-5.54
0081dim	$C_6H_{12}O$	octanol	-4.53	-5.34
0082hep	$C_7H_{14}O$	octanol	-5.65	-6.21
0084met	C_8H_8O	octanol	-6.74	-7.71
0086eth	$C_2H_4O_2$	octanol	-6.35	-5.87
0087pro	$C_3H_6O_2$	octanol	-6.86	-6.24
0088but	$C_4H_8O_2$	octanol	-7.58	-6.80
0089pen	$C_5H_{10}O_2$	octanol	-8.22	-7.44
0090hex	$C_6H_{12}O_2$	octanol	-8.82	-8.09
0091met	$C_2H_4O_2$	octanol	-2.82	-4.19
0093met	$C_3H_6O_2$	octanol	-3.54	-4.42
0094met	$C_4H_8O_2$	octanol	-4.06	-4.83
0095eth	$C_4H_8O_2$	octanol	-4.06	-4.63
0096met	$C_5H_{10}O_2$	octanol	-4.59	-5.53
0097pro	$C_5H_{10}O_2$	octanol	-4.55	-5.31
0098met	$C_6H_{12}O_2$	octanol	-5.13	-6.30
0099but	$C_6H_{12}O_2$	octanol	-4.96	-6.09
0103eth	C_2H_7N	octanol	-4.09	-3.20
0106pro	C_3H_9N	octanol	-4.77	-3.99
0107tri	C_3H_9N	octanol	-3.60	-2.87
0109pip	$C_4H_{10}N_2$	octanol	-5.80	-6.98
0110but	$C_4H_{11}N$	octanol	-5.33	-4.85
0111die	$C_4H_{11}N$	octanol	-4.75	-3.81
0115dip	$C_6H_{15}N$	octanol	-6.02	-5.49
0116pyr	C_5H_5N	octanol	-5.34	-5.21
0117met	$C_5H_6N_2$	octanol	-5.87	-6.55
0118ani	C_6H_7N	octanol	-6.71	-7.23
0119met	C_6H_7N	octanol	-6.14	-5.43
0120met	C_6H_7N	octanol	-6.40	-5.69
0121met	C_6H_7N	octanol	-6.60	-5.64
0122Nme	C_7H_9N	octanol	-6.94	-6.71
0126eth	C_2H_3N	octanol	-3.15	-3.00
0127pro	C_3H_5N	octanol	-3.66	-3.77
0128butb	C_4H_7N	octanol	-4.25	-4.57
0129ben	C_7H_5N	octanol	-6.09	-6.52
0130nit	$C_2H_5NO_2$	octanol	-3.93	-4.96
0131nit	$C_3H_7NO_2$	octanol	-4.44	-5.69
0132nit	$C_3H_7NO_2$	octanol	-4.23	-5.44
0133nit	$C_4H_9NO_2$	octanol	-5.11	-6.45
0134nit	$C_6H_5NO_2$	octanol	-6.63	-7.56
0135met	$C_7H_7NO_2$	octanol	-6.80	-8.28
0138pro	C_3H_8S	octanol	-3.52	-3.44
0139thi	C_6H_6S	octanol	-5.99	-6.31
0141dim	$C_2H_6S_2$	octanol	-4.24	-4.47
0142die	$C_4H_{10}S$	octanol	-4.09	-4.13
0144thi	C_7H_8S	octanol	-6.47	-6.91
0145pro	C_3H_6O	octanol	-5.27	-4.95
0146met	$C_3H_8O_2$	octanol	-5.83	-5.46
0149mor	C_4H_9NO	octanol	-5.99	-6.32
0150mhy	$C_7H_6O_2$	octanol	-11.39	-10.14
0151phy	$C_7H_6O_2$	octanol	-12.36	-10.12
0152eth	$C_7H_{10}N_2O$	octanol	-6.85	-9.60
0154dif	$C_2H_4F_2$	octanol	-1.13	-1.66
0157flu	C_6H_5F	octanol	-3.87	-4.23
0161dic	CH_2Cl_2	octanol	-3.07	-2.44
0162tri	$CHCl_3$	octanol	-3.81	-3.35
0163chl	C_2H_5Cl	octanol	-2.58	-2.22
0165tri	$C_2H_3Cl_3$	octanol	-3.69	-4.26
0166tri	$C_2H_3Cl_3$	octanol	-4.53	-4.65
0167chla	C_3H_7Cl	octanol	-3.06	-2.77
0168chl	C_3H_7Cl	octanol	-2.84	-2.71
0171Zdi	$C_2H_2Cl_2$	octanol	-3.71	-3.13
0172Edi	$C_2H_2Cl_2$	octanol	-3.61	-3.17
0173tri	C_2HCl_3	octanol	-3.75	-3.97
0174chl	C_6H_5Cl	octanol	-5.00	-4.84
0175odi	$C_6H_4Cl_2$	octanol	-6.01	-5.95
0176pdi	$C_6H_4Cl_2$	octanol	-5.67	-6.03
0177bro	CH_3Br	octanol	-2.43	-2.22
0178dib	CH_2Br_2	octanol	-4.18	-3.94
0179tri	$CHBr_3$	octanol	-5.62	-5.44
0180bro	C_2H_5Br	octanol	-2.90	-2.91
0182bro	C_3H_7Br	octanol	-3.42	-3.61
0183bro	C_3H_7Br	octanol	-3.40	-3.58
0184bro	C_4H_9Br	octanol	-4.16	-4.39

0185bro	$C_5H_{11}Br$	octanol	-4.68	-5.13
0186bro	C_6H_5Br	octanol	-5.46	-5.90
0187dib	$C_6H_4Br_2$	octanol	-7.47	-7.82
0197bro	CF_3Br	octanol	-0.75	-2.71
0199chl	CH_1F_2Cl	octanol	-1.97	-1.62
0200tet	CF_4	octanol	1.50	-1.31
0201bro	$C_2H_1F_3ClBr$	octanol	-3.27	-3.52
0204tet	C_2Cl_4	octanol	-4.24	-4.39
0206tri	$C_2F_3Cl_3$	octanol	-2.54	-2.88
0207tri	$C_2H_3OF_3$	octanol	-4.81	-3.87
0210dic	$C_3H_4OF_2Cl_2$	octanol	-4.02	-5.32
0211tri	$C_3H_5OF_3$	octanol	-5.12	-4.30
0212hex	$C_3H_2OF_6$	octanol	-5.76	-3.65
0215pbr	C_6H_5OBr	octanol	-10.59	-9.10
0217wat	H_2O	octanol	-4.43	-5.43
0220tri	$C_3H_9O_4P$	octanol	-7.81	-6.96
0221tri	$C_6H_{15}O_4P$	octanol	-8.88	-7.66
0222tri	$C_9H_{21}O_4P$	octanol	-8.65	-9.29
0225pipa	$C_5H_{11}N$	octanol	-6.27	-5.36
0228met	CH_5N	octanol	-3.78	-2.73
0229hyd	H_4N_2	octanol	-3.44	-5.86
0230eth	$C_6H_8N_2$	octanol	-6.40	-7.31
0236oct	$C_8H_{18}O$	octanol	-8.13	-7.69
0239oct	$C_8H_{16}O$	octanol	-6.38	-6.90
0240met	$C_8H_8O_2$	octanol	-7.26	-8.68
0244tet	$C_5H_{10}O$	octanol	-4.21	-4.70
0245thi	C_4H_4S	octanol	-3.89	-5.04
0246eth	$C_8H_{10}O$	octanol	-5.65	-6.90
0400hyd	H_2	octanol	1.76	-2.12
0401amia	$C_9H_{12}N_2O$	octanol	-13.12	-12.70
0402adn	$C_6H_7N_5$	octanol	-13.56	-13.49
0414dcl	$C_{12}H_8Cl_2$	octanol	-9.41	-10.30
0415dcl	$C_{12}H_8Cl_2$	octanol	-9.23	-10.41
0416dcl	$C_{12}H_7Cl_3$	octanol	-9.12	-11.40
0417brp	C_3H_5Br	octanol	-3.30	-3.44
0419brt	C_7H_7Br	octanol	-6.36	-6.91
0421dfl	CF_2Cl_2	octanol	-1.25	-1.59
0422ftc	$CFCl_3$	octanol	-2.63	-2.63
0426dcl	$C_7H_3NCl_2$	octanol	-9.18	-6.79
0428ami	$C_6H_3N_2O_2Cl_3$	octanol	-12.37	-15.73
0433pho	$C_4H_7O_4PCl_2$	octanol	-8.59	-9.47
0437pho	$C_9H_{13}O_3PS_2$	octanol	-12.55	-11.59
0438pho	$C_{10}H_{13}O_3PSCl_2$	octanol	-10.87	-10.69
0441pho	$C_8H_{10}NO_5PS$	octanol	-11.70	-10.91
0442pho	$C_{11}H_{15}O_3PSClBr$	octanol	-10.49	-11.96
0444pho	$C_8H_8O_3PSCl_3$	octanol	-11.69	-10.63
0445pho	$C_8H_8O_3PSCl_2Br$	octanol	-12.30	-10.75
0447pho	$C_{10}H_{14}NO_5PS$	octanol	-11.31	-11.81
0449pho	$C_{15}H_{14}NO_2PS$	octanol	-11.06	-14.61
0506nit	CH_3NO_2	octanol	-3.51	-4.25
0515dim	C_3H_7NO	octanol	-6.14	-5.58
0517met	C_2H_5NO	octanol	-6.97	-5.79
0519dim	C_4H_9NO	octanol	-7.48	-6.04
0574eth	C_7H_9N	octanol	-7.80	-6.36
0579pyy	C_4H_5N	octanol	-5.28	-4.65
0582qui	C_9H_7N	octanol	-8.43	-7.78
0648gbu	$C_4H_6O_2$	octanol	-6.83	-5.44
0939tet	$C_4H_{12}Si$	octanol	-1.79	-1.31
n005	CH_6N_2	octanol	-3.88	-5.44
n007	CH_4N_2O	octanol	-10.93	-10.71
n008	C_7H_7NO	octanol	-11.77	-11.26
n009	C_7H_9N	octanol	-7.36	-7.69
n010	C_7H_9N	octanol	-7.57	-7.73
n011	C_7H_9N	octanol	-7.44	-7.75
n127	CH_3NO	octanol	-7.80	-6.90
n185	C_4H_7NO	octanol	-8.85	-7.32
n186	C_5H_9NO	octanol	-8.63	-6.87
n191	$C_4H_4N_2O_2$	octanol	-15.13	-14.05
n200	$C_4H_3N_2O_2F$	octanol	-15.71	-14.66
n201	$C_5H_3N_2O_2F_3$	octanol	-15.51	-14.35
n202	$C_4H_3N_2O_2Cl$	octanol	-17.26	-15.34
n203	$C_4H_3N_2O_2Br$	octanol	-17.88	-18.12
test1035	$C_6H_6Cl_6$	octanol	-10.71	-9.78
test2001	$C_9H_8O_4$	octanol	-11.56	-11.82
test2003	$C_{11}H_{14}O_3$	octanol	-13.59	-12.63
test2004	$C_8H_{10}N_4O_2$	octanol	-12.54	-16.89
test2007	$C_3H_3N_3O_3$	octanol	-18.89	-18.57
test2010	$C_{13}H_8O_3F_2$	octanol	-15.46	-15.72
test2011	$C_9H_{10}O_3$	octanol	-12.57	-11.52
test2013	$C_{15}H_{13}O_2F$	octanol	-14.10	-15.18
test2015	C_6Cl_6	octanol	-10.15	-9.10
test2017	$C_{13}H_{18}O_2$	octanol	-12.42	-12.72
test2018	$C_4H_3N_2O_2I$	octanol	-18.77	-16.61

test2019	$C_{16}H_{14}O_3$	octanol	-15.04	-16.57
test2020	$C_8H_8O_3$	octanol	-12.18	-11.30
test2021	$C_{14}H_{14}O_3$	octanol	-14.55	-15.20
test2022	$C_6H_6N_2O_2$	octanol	-11.35	-10.77
test2023	C_4F_8	octanol	0.31	-1.31
test2024	$C_6NO_2Cl_5$	octanol	-11.55	-8.75
test2025	$C_8H_5NO_2$	octanol	-11.18	-13.03
test2026	$C_{10}H_{12}O_3$	octanol	-13.52	-12.05
test2027	$C_4H_8O_2S$	octanol	-7.56	-10.35
test3001	$C_8H_9NO_2$	octanol	-15.46	-12.60
test3002	$C_8H_9NO_2$	octanol	-14.93	-12.76
test3003	$C_{16}H_{14}O_3$	octanol	-17.12	-16.95
test3004	$C_8H_9NO_2$	octanol	-12.59	-13.32
test3005	$C_{10}H_{13}NO_2$	octanol	-13.07	-11.98
test3007	$C_8H_8O_3$	octanol	-12.49	-10.28
test3014	$C_8H_8O_3$	octanol	-11.82	-11.41
test3015	$C_8H_8O_3$	octanol	-11.69	-11.37
test3019	$C_{14}H_{12}NO_2Cl$	octanol	-13.76	-16.38
test3020	$C_{14}H_{11}NO_2Cl_2$	octanol	-12.45	-14.11
test3021	$C_{14}H_{10}NO_2F_3$	octanol	-12.84	-15.28
test4001	C_6H_5I	octanol	-6.18	-6.80
test4002	CH_2I_2	octanol	-5.63	-6.47
test4003	CH_3I	octanol	-3.07	-2.99
test4004	C_2H_5I	octanol	-3.45	-3.70
test4009	C_3H_7I	octanol	-4.40	-4.20
0035ben	C_6H_6	heptanol	-3.73	-3.65
0036tol	C_7H_8	heptanol	-4.33	-4.18
0037eth	C_8H_{10}	heptanol	-4.58	-4.90
0053phe	C_6H_6O	heptanol	-8.69	-7.19
0055ocr	C_7H_8O	heptanol	-8.78	-7.59
0057pcr	C_7H_8O	heptanol	-9.16	-7.65
0058hep	$C_7H_{16}O$	heptanol	-7.84	-7.09
0086eth	$C_2H_4O_2$	heptanol	-6.70	-5.98
0103eth	C_2H_7N	heptanol	-4.15	-3.10
0106pro	C_3H_9N	heptanol	-4.80	-3.97
0110but	$C_4H_{11}N$	heptanol	-5.40	-4.84
0215pbr	C_6H_5OBr	heptanol	-10.49	-9.24
0008noc	C_8H_{18}	m-cresol	-4.02	-4.16
0036tol	C_7H_8	m-cresol	-4.58	-4.12
0045eth	C_2H_6O	m-cresol	-5.58	-3.62
0056mcr	C_7H_8O	m-cresol	-8.40	-7.59
0062dio	$C_4H_8O_2$	m-cresol	-6.82	-5.23
0076but	C_4H_8O	m-cresol	-5.98	-4.39
0506nit	CH_3NO_2	m-cresol	-4.73	-4.36
0008noc	C_8H_{18}	benzyl alcohol	-3.77	-4.15
0036tol	C_7H_8	benzyl alcohol	-4.46	-4.11
0045eth	C_2H_6O	benzyl alcohol	-4.84	-3.62
0062dio	$C_4H_8O_2$	benzyl alcohol	-5.39	-5.23
0069met	CH_2O	benzyl alcohol	-3.48	-2.39
0076but	C_4H_8O	benzyl alcohol	-4.57	-4.38
0086eth	$C_2H_4O_2$	benzyl alcohol	-6.96	-5.99
0110but	$C_4H_{11}N$	benzyl alcohol	-5.15	-4.82
0506nit	CH_3NO_2	benzyl alcohol	-4.53	-4.36
0533ben	C_7H_8O	benzyl alcohol	-8.61	-8.21
0035ben	C_6H_6	hexanol	-3.68	-3.66
0036tol	C_7H_8	hexanol	-4.27	-4.25
0037eth	C_8H_{10}	hexanol	-4.54	-4.96
0053phe	C_6H_6O	hexanol	-8.76	-7.18
0054hex	$C_6H_{14}O$	hexanol	-7.05	-6.56
0055ocr	C_7H_8O	hexanol	-8.76	-7.65
0056mcr	C_7H_8O	hexanol	-8.42	-7.68
0057pcr	C_7H_8O	hexanol	-9.21	-7.71
0069met	CH_2O	hexanol	-3.42	-2.40
0086eth	$C_2H_4O_2$	hexanol	-6.51	-5.98
0103eth	C_2H_7N	hexanol	-4.20	-3.08
0106pro	C_3H_9N	hexanol	-4.83	-3.94
0110but	$C_4H_{11}N$	hexanol	-5.50	-4.81
0215pbr	C_6H_5OBr	hexanol	-10.51	-9.25
0035ben	C_6H_6	pentanol	-3.53	-3.71
0036tol	C_7H_8	pentanol	-4.25	-4.29
0037eth	C_8H_{10}	pentanol	-4.48	-5.00
0052pen	$C_5H_{12}O$	pentanol	-7.92	-6.05
0053phe	C_6H_6O	pentanol	-8.55	-7.19
0055ocr	C_7H_8O	pentanol	-8.57	-7.77
0057pcr	C_7H_8O	pentanol	-9.25	-7.78
0069met	CH_2O	pentanol	-3.44	-2.55
0086eth	$C_2H_4O_2$	pentanol	-6.65	-5.83
0087pro	$C_3H_6O_2$	pentanol	-7.09	-6.46
0088but	$C_4H_8O_2$	pentanol	-7.74	-7.04
0089pen	$C_5H_{10}O_2$	pentanol	-8.17	-7.73
0090hex	$C_6H_{12}O_2$	pentanol	-8.99	-8.46
0103eth	C_2H_7N	pentanol	-4.27	-2.96
0106pro	C_3H_9N	pentanol	-4.88	-3.87

0110but	$C_4H_{11}N$	pentanol	-5.55	-4.79
0111die	$C_4H_{11}N$	pentanol	-5.30	-3.72
0118ani	C_6H_7N	pentanol	-6.44	-7.03
0215pbr	C_6H_5OBr	pentanol	-10.62	-9.27
0216amm	H_3N	pentanol	-3.13	-3.53
0228met	CH_5N	pentanol	-3.95	-2.45
n017	H_2O_2	pentanol	-7.46	-5.65
0069met	CH_2O	sec-butanol	-2.86	-2.67
0086eth	$C_2H_4O_2$	sec-butanol	-6.81	-5.65
0087pro	$C_3H_6O_2$	sec-butanol	-7.00	-6.39
0088but	$C_4H_8O_2$	sec-butanol	-7.34	-6.97
0089pen	$C_5H_{10}O_2$	sec-butanol	-7.61	-7.66
0090hex	$C_6H_{12}O_2$	sec-butanol	-8.11	-8.41
0110but	$C_4H_{11}N$	sec-butanol	-5.52	-4.60
0217wat	H_2O	sec-butanol	-5.71	-5.27
0509sec	$C_4H_{10}O$	sec-butanol	-5.48	-4.23
0072but	C_4H_8O	isobutanol	-4.82	-5.20
0086eth	$C_2H_4O_2$	isobutanol	-6.80	-5.65
0087pro	$C_3H_6O_2$	isobutanol	-6.98	-6.39
0088but	$C_4H_8O_2$	isobutanol	-7.62	-6.97
0089pen	$C_5H_{10}O_2$	isobutanol	-8.06	-7.66
0090hex	$C_6H_{12}O_2$	isobutanol	-8.77	-8.41
0095eth	$C_4H_8O_2$	isobutanol	-4.27	-5.08
0104dim	C_2H_7N	isobutanol	-4.43	-2.85
0107tri	C_3H_9N	isobutanol	-3.90	-3.17
0109pip	$C_4H_{10}N_2$	isobutanol	-6.58	-7.13
0111die	$C_4H_{11}N$	isobutanol	-4.86	-3.54
0115dip	$C_6H_{15}N$	isobutanol	-5.87	-5.34
0116pyr	C_5H_5N	isobutanol	-5.87	-5.26
0225pipa	$C_5H_{11}N$	isobutanol	-6.17	-4.97
0228met	CH_5N	isobutanol	-4.56	-2.46
0507iso	$C_4H_{10}O$	isobutanol	-5.79	-3.38
n017	H_2O_2	isobutanol	-7.93	-5.48
0008noc	C_8H_{18}	methoxyethanol	-3.71	-4.10
0036tol	C_7H_8	methoxyethanol	-4.49	-4.33
0045eth	C_2H_6O	methoxyethanol	-4.71	-3.65
0062dio	$C_4H_8O_2$	methoxyethanol	-4.91	-5.32
0076but	C_4H_8O	methoxyethanol	-4.28	-4.71
0506nit	CH_3NO_2	methoxyethanol	-5.06	-4.55
0008noc	C_8H_{18}	butanol	-4.45	-4.11
0035ben	C_6H_6	butanol	-3.39	-3.76
0036tol	C_7H_8	butanol	-4.50	-4.34
0037eth	C_8H_{10}	butanol	-4.46	-5.05
0044met	CH_4O	butanol	-4.73	-3.20
0045eth	C_2H_6O	butanol	-5.02	-3.64
0046eth	$C_2H_6O_2$	butanol	-8.69	-7.05
0049but	$C_4H_{10}O$	butanol	-6.03	-5.14
0069met	CH_2O	butanol	-3.49	-2.71
0076but	C_4H_8O	butanol	-4.12	-4.72
0086eth	$C_2H_4O_2$	butanol	-6.81	-5.65
0087pro	$C_3H_6O_2$	butanol	-7.17	-6.39
0088but	$C_4H_8O_2$	butanol	-7.66	-6.97
0089pen	$C_5H_{10}O_2$	butanol	-8.02	-7.66
0090hex	$C_6H_{12}O_2$	butanol	-8.75	-8.40
0103eth	C_2H_7N	butanol	-4.50	-2.85
0106pro	C_3H_9N	butanol	-5.04	-3.70
0111die	$C_4H_{11}N$	butanol	-5.15	-3.55
0506nit	CH_3NO_2	butanol	-3.93	-4.56
n017	H_2O_2	butanol	-7.94	-5.47
n191	$C_4H_4N_2O_2$	butanol	-16.05	-15.14
0008noc	C_8H_{18}	isopropanol	-4.50	-4.04
0036tol	C_7H_8	isopropanol	-4.38	-4.52
0045eth	C_2H_6O	isopropanol	-4.84	-3.77
0048pro	C_3H_8O	isopropanol	-4.82	-3.81
0062dio	$C_4H_8O_2$	isopropanol	-4.49	-5.57
0076but	C_4H_8O	isopropanol	-4.07	-4.66
0506nit	CH_3NO_2	isopropanol	-4.00	-4.69
0008noc	C_8H_{18}	propanol	-4.39	-3.99
0036tol	C_7H_8	propanol	-4.47	-4.47
0045eth	C_2H_6O	propanol	-5.01	-3.80
0047pro	C_3H_8O	propanol	-5.29	-4.37
0062dio	$C_4H_8O_2$	propanol	-4.61	-5.56
0076but	C_4H_8O	propanol	-4.15	-4.69
0506nit	CH_3NO_2	propanol	-4.04	-4.68
0008noc	C_8H_{18}	ethanol	-4.23	-4.13
0036tol	C_7H_8	ethanol	-4.57	-4.63
0045eth	C_2H_6O	ethanol	-5.04	-4.05
0062dio	$C_4H_8O_2$	ethanol	-4.68	-5.78
0076but	C_4H_8O	ethanol	-4.32	-5.01
0174chl	C_6H_5Cl	ethanol	-3.30	-5.55
0506nit	CH_3NO_2	ethanol	-4.34	-4.90
0648gbu	$C_4H_6O_2$	ethanol	-4.58	-5.71
i003	CH_6N^+	methanol	-74.30	-77.24

i013	$C_3H_{10}N^+$	methanol	-59.50	-61.85
i014	$C_6H_{16}N^+$	methanol	-53.00	-53.40
i018	$C_6H_8N^+$	methanol	-74.10	-74.97
i019	$C_7H_{10}N^+$	methanol	-72.10	-71.37
i020	$C_7H_{10}N^+$	methanol	-71.30	-72.61
i021	$C_7H_{10}N^+$	methanol	-71.70	-72.70
i024	$C_7H_{10}N^+$	methanol	-63.50	-63.67
i029	$C_{10}H_{10}N^+$	methanol	-69.60	-70.02
i033	$C_5H_{12}N^+$	methanol	-62.90	-64.01
i036	$C_5H_6N^+$	methanol	-60.80	-62.69
i037	$C_9H_8N^+$	methanol	-57.50	-57.59
i047	H_4N^+	methanol	-85.60	-86.93
i093	$C_7H_{10}NO^+$	methanol	-72.10	-70.87
i094	$C_6H_7N_2O_2^+$	methanol	-75.30	-75.05
i125	$C_6H_7NCl^+$	methanol	-76.00	-76.02
i126	$C_6H_7NCl^+$	methanol	-75.50	-75.82
i130	$C_6H_{14}N^+$	methanol	-56.30	-56.05
i133	$C_7H_{10}N^+$	methanol	-55.60	-56.46
i140	$C_6H_8N^+$	methanol	-57.70	-57.52
i141	$C_6H_7NBr^+$	methanol	-75.20	-75.38
i144	$C_7H_{10}NO^+$	methanol	-68.00	-70.42
i145	$C_6H_8N^+$	methanol	-58.50	-58.14
i148	$C_6H_5N_2^+$	methanol	-72.10	-71.55
i149	$C_7H_{10}N^+$	methanol	-57.60	-57.75
i151	$C_6H_8N^+$	methanol	-58.10	-58.15
i153	$C_{11}H_{10}N^+$	methanol	-62.30	-61.78
i163	$C_2H_8N^+$	methanol	-71.00	-72.58
i176	$C_4H_{12}N^+$	methanol	-70.80	-70.42
i059	$C_2H_3O_2^-$	methanol	-72.90	-71.94
i060	$C_3H_5O_2^-$	methanol	-72.00	-71.27
i062	$C_3H_3O_2^-$	methanol	-68.80	-68.29
i064	$C_7H_5O_2^-$	methanol	-67.50	-66.27
i074	$C_6H_5O^-$	methanol	-69.30	-67.00
i079	$C_7H_7O_2^-$	methanol	-70.00	-69.33
i101	$C_6H_4NO_3^-$	methanol	-58.90	-57.13
i102	$C_6H_4NO_3^-$	methanol	-54.10	-55.56
i115	Cl^-	methanol	-71.50	-70.91
i116	Br^-	methanol	-65.80	-65.69
i119	$C_2H_2O_2Cl^-$	methanol	-64.70	-64.76
i120	$C_2HO_2Cl_2^-$	methanol	-59.30	-59.29
i123	$C_6H_4OCl^-$	methanol	-64.20	-63.77
i191	$C_6H_3N_2O_5^-$	methanol	-43.90	-44.24
i194	$C_7H_4O_2Cl^-$	methanol	-64.10	-63.61
i197	$C_7H_4O_2F^-$	methanol	-67.50	-67.55
i198	$C_6H_4OF^-$	methanol	-66.00	-65.88
i199	$C_7H_5O_3^-$	methanol	-55.40	-55.87
i205	$C_7H_4NO_4^-$	methanol	-62.20	-62.05
i207	$C_{10}H_{13}O^-$	methanol	-61.60	-61.24
i209	$C_9H_9O_2^-$	methanol	-72.10	-70.21
i213	$C_7H_4O_2Cl^-$	methanol	-63.90	-63.34
i215	$C_6H_4OCl^-$	methanol	-64.00	-62.61
i217	$C_8H_4NO_2^-$	methanol	-59.00	-59.35
i218	$C_7H_5O_3^-$	methanol	-68.40	-67.78
i219	$C_8H_7O_2^-$	methanol	-68.20	-68.27
i220	$C_7H_4NO_4^-$	methanol	-59.30	-58.98
i221	$C_8H_4O_2F_3^-$	methanol	-60.20	-60.43
i224	$C_7H_6NO_2^-$	methanol	-73.10	-72.72
i226	$C_7H_4O_2Cl^-$	methanol	-63.90	-63.51
i228	$C_7H_4O_2F^-$	methanol	-64.40	-64.46
i229	$C_7H_5O_2^-$	methanol	-59.20	-59.36
i231	$C_7H_5O_3^-$	methanol	-65.70	-65.83
i232	$C_8H_7O_3^-$	methanol	-69.00	-68.35
i233	$C_8H_7O_2^-$	methanol	-68.60	-68.31
i234	$C_7H_4NO_4^-$	methanol	-58.20	-57.56
i235	$C_{11}H_{13}O_2^-$	methanol	-67.40	-67.93
i236	$C_{10}H_{13}O^-$	methanol	-68.00	-67.78
i242	$C_2H_2O_2Br^-$	methanol	-63.00	-65.30
i243	$C_4H_7O_2^-$	methanol	-71.40	-70.59
i245	$C_3H_2NO_2^-$	methanol	-61.70	-61.98
i246	$C_5H_7O_2^-$	methanol	-71.30	-71.32
i247	$C_7H_{11}O_2^-$	methanol	-70.80	-69.30
i250	$C_4H_5O_2^-$	methanol	-71.70	-72.19
i253	$C_2H_2O_2F^-$	methanol	-67.20	-67.65
i255	$C_2H_3O_3^-$	methanol	-66.60	-65.86
i259	$C_7H_7O^-$	methanol	-69.80	-68.94
i266	$C_7H_7O^-$	methanol	-68.10	-68.83
i267	$C_7H_7O^-$	methanol	-70.20	-68.84
i269	$C_8H_7O_2^-$	methanol	-69.50	-69.24
i281	$C_8H_7O_3^-$	methanol	-67.70	-68.68

Table S8. Polar aprotic solvents

Solute MNSol code[1]	Solute formula	Solvent	$\Delta^{ref}G^{\circ}_{solv}$	$\Delta^{calc}G^{\circ}_{solv}$
0008noc	C_8H_{18}	bromoethane	-5.54	-4.60
0036tol	C_7H_8	bromoethane	-5.55	-4.94
0062dio	$C_4H_8O_2$	bromoethane	-5.39	-5.70
0076but	C_4H_8O	bromoethane	-5.13	-4.77
0087pro	$C_3H_6O_2$	bromoethane	-5.52	-6.34
0180bro	C_2H_5Br	bromoethane	-3.67	-3.74
0506nit	CH_3NO_2	bromoethane	-4.54	-4.75
0008noc	C_8H_{18}	2-methylpyridine	-4.73	-4.42
0036tol	C_7H_8	2-methylpyridine	-5.06	-4.33
0045eth	C_2H_6O	2-methylpyridine	-5.01	-3.70
0062dio	$C_4H_8O_2$	2-methylpyridine	-5.01	-5.30
0076but	C_4H_8O	2-methylpyridine	-4.52	-4.56
0119met	C_6H_7N	2-methylpyridine	-5.71	-5.71
0044met	CH_4O	*o*-dichlorobenzene	-1.73	-3.12
0045eth	C_2H_6O	*o*-dichlorobenzene	-2.34	-3.60
0047pro	C_3H_8O	*o*-dichlorobenzene	-3.47	-4.23
0049but	$C_4H_{10}O$	*o*-dichlorobenzene	-3.90	-4.96
0052pen	$C_5H_{12}O$	*o*-dichlorobenzene	-4.93	-5.80
0054hex	$C_6H_{14}O$	*o*-dichlorobenzene	-5.70	-6.42
0058hep	$C_7H_{16}O$	*o*-dichlorobenzene	-6.50	-7.05
0103eth	C_2H_7N	*o*-dichlorobenzene	-2.59	-3.08
0106pro	C_3H_9N	*o*-dichlorobenzene	-3.44	-3.94
0110but	$C_4H_{11}N$	*o*-dichlorobenzene	-4.13	-4.81
0175odi	$C_6H_4Cl_2$	*o*-dichlorobenzene	-6.89	-6.02
0044met	CH_4O	dichloroethane	-2.53	-3.22
0045eth	C_2H_6O	dichloroethane	-2.83	-3.60
0047pro	C_3H_8O	dichloroethane	-3.85	-4.26
0049but	$C_4H_{10}O$	dichloroethane	-4.92	-5.03
0052pen	$C_5H_{12}O$	dichloroethane	-5.45	-5.84
0053phe	C_6H_6O	dichloroethane	-7.48	-7.27
0054hex	$C_6H_{14}O$	dichloroethane	-6.02	-6.72
0055ocr	C_7H_8O	dichloroethane	-7.73	-7.88
0056mcr	C_7H_8O	dichloroethane	-6.91	-7.75
0057pcr	C_7H_8O	dichloroethane	-7.75	-7.75
0058hep	$C_7H_{16}O$	dichloroethane	-6.79	-7.45
0074ben	C_7H_6O	dichloroethane	-7.23	-7.29
0084met	C_8H_8O	dichloroethane	-7.83	-8.06
0086eth	$C_2H_4O_2$	dichloroethane	-4.89	-5.48
0087pro	$C_3H_6O_2$	dichloroethane	-5.12	-6.25
0088but	$C_4H_8O_2$	dichloroethane	-5.83	-6.74
0089pen	$C_5H_{10}O_2$	dichloroethane	-6.47	-7.42
0090hex	$C_6H_{12}O_2$	dichloroethane	-7.33	-8.15
0093met	$C_3H_6O_2$	dichloroethane	-4.55	-4.78
0094met	$C_4H_8O_2$	dichloroethane	-4.87	-5.39
0095eth	$C_4H_8O_2$	dichloroethane	-4.93	-5.12
0097pro	$C_5H_{10}O_2$	dichloroethane	-5.40	-5.73
0098met	$C_6H_{12}O_2$	dichloroethane	-5.97	-6.84
0099but	$C_6H_{12}O_2$	dichloroethane	-5.93	-6.55
0100met	$C_7H_{14}O_2$	dichloroethane	-6.57	-7.68
0101pen	$C_7H_{14}O_2$	dichloroethane	-6.64	-7.39
0103eth	C_2H_7N	dichloroethane	-3.19	-2.92
0106pro	C_3H_9N	dichloroethane	-4.04	-3.76
0110but	$C_4H_{11}N$	dichloroethane	-4.34	-4.60
0111die	$C_4H_{11}N$	dichloroethane	-4.00	-3.68
0116pyr	C_5H_5N	dichloroethane	-5.53	-5.53
0118ani	C_6H_7N	dichloroethane	-7.39	-7.02
0150mhy	$C_7H_6O_2$	dichloroethane	-10.11	-10.73
0151phy	$C_7H_6O_2$	dichloroethane	-10.70	-10.71
0215pbr	C_6H_5OBr	dichloroethane	-9.10	-9.29
0220tri	$C_3H_9O_4P$	dichloroethane	-8.55	-8.78
0221tri	$C_6H_{15}O_4P$	dichloroethane	-9.59	-9.06
0521dic	$C_2H_4Cl_2$	dichloroethane	-4.69	-4.53
n008	C_7H_7NO	dichloroethane	-10.90	-12.05
0041nap	$C_{10}H_8$	4-methyl-2-pentanone	-7.45	-7.14
0053phe	C_6H_6O	4-methyl-2-pentanone	-9.38	-7.26
0056mcr	C_7H_8O	4-methyl-2-pentanone	-8.79	-7.76
0086eth	$C_2H_4O_2$	4-methyl-2-pentanone	-6.33	-6.03
0087pro	$C_3H_6O_2$	4-methyl-2-pentanone	-6.85	-6.52
0088but	$C_4H_8O_2$	4-methyl-2-pentanone	-7.44	-7.08
0107tri	C_3H_9N	4-methyl-2-pentanone	-2.86	-2.96
0111die	$C_4H_{11}N$	4-methyl-2-pentanone	-3.63	-3.80
0116pyr	C_5H_5N	4-methyl-2-pentanone	-5.33	-5.34
0118ani	C_6H_7N	4-methyl-2-pentanone	-7.54	-7.18
0216amm	H_3N	4-methyl-2-pentanone	-2.52	-3.82
0228met	CH_5N	4-methyl-2-pentanone	-4.14	-2.58
0508met	$C_6H_{12}O$	4-methyl-2-pentanone	-5.23	-5.76
0008noc	C_8H_{18}	pyridine	-4.50	-4.36
0036tol	C_7H_8	pyridine	-5.10	-4.37
0045eth	C_2H_6O	pyridine	-5.08	-3.72
0062dio	$C_4H_8O_2$	pyridine	-5.14	-5.31
0076but	C_4H_8O	pyridine	-4.61	-4.74

0116pyr	C_5H_5N	pyridine	-5.47	-5.30
0506nit	CH_3NO_2	pyridine	-5.11	-4.49
0008noc	C_8H_{18}	cyclohexanone	-4.57	-4.21
0036tol	C_7H_8	cyclohexanone	-5.05	-4.21
0045eth	C_2H_6O	cyclohexanone	-4.41	-3.75
0062dio	$C_4H_8O_2$	cyclohexanone	-4.95	-5.36
0076but	C_4H_8O	cyclohexanone	-4.42	-4.58
0086eth	$C_2H_4O_2$	cyclohexanone	-6.43	-6.05
0087pro	$C_3H_6O_2$	cyclohexanone	-7.18	-6.54
0506nit	CH_3NO_2	cyclohexanone	-5.09	-4.50
0523cyc	$C_6H_{10}O$	cyclohexanone	-6.25	-6.42
n017	H_2O_2	cyclohexanone	-9.11	-5.73
0008noc	C_8H_{18}	acetophenone	-4.24	-3.99
0036tol	C_7H_8	acetophenone	-4.90	-4.12
0045eth	C_2H_6O	acetophenone	-4.12	-3.61
0062dio	$C_4H_8O_2$	acetophenone	-5.03	-5.22
0076but	C_4H_8O	acetophenone	-4.39	-4.19
0084met	C_8H_8O	acetophenone	-7.59	-7.72
0086eth	$C_2H_4O_2$	acetophenone	-6.20	-6.03
0506nit	CH_3NO_2	acetophenone	-4.92	-4.37
n017	H_2O_2	acetophenone	-7.30	-5.82
0008noc	C_8H_{18}	butanone	-4.64	-4.17
0036tol	C_7H_8	butanone	-5.06	-4.41
0045eth	C_2H_6O	butanone	-4.46	-3.61
0062dio	$C_4H_8O_2$	butanone	-5.02	-5.37
0069met	CH_2O	butanone	-1.77	-2.68
0076but	C_4H_8O	butanone	-4.50	-4.75
0086eth	$C_2H_4O_2$	butanone	-6.88	-5.69
0087pro	$C_3H_6O_2$	butanone	-7.05	-6.43
0088but	$C_4H_8O_2$	butanone	-7.34	-7.01
0089pen	$C_5H_{10}O_2$	butanone	-7.54	-7.70
0090hex	$C_6H_{12}O_2$	butanone	-8.07	-8.45
0506nit	CH_3NO_2	butanone	-5.24	-4.60
0648gbu	$C_4H_6O_2$	butanone	-4.47	-5.30
0008noc	C_8H_{18}	benzonitrile	-4.34	-3.89
0036tol	C_7H_8	benzonitrile	-4.95	-3.95
0045eth	C_2H_6O	benzonitrile	-4.05	-3.67
0062dio	$C_4H_8O_2$	benzonitrile	-5.14	-5.29
0076but	C_4H_8O	benzonitrile	-4.58	-4.26
0129ben	C_7H_5N	benzonitrile	-7.28	-6.57
0506nit	CH_3NO_2	benzonitrile	-5.05	-4.45
0053phe	C_6H_6O	*o*-nitrotoluene	-7.79	-7.21
0086eth	$C_2H_4O_2$	*o*-nitrotoluene	-4.68	-5.99
0087pro	$C_3H_6O_2$	*o*-nitrotoluene	-5.30	-6.37
0088but	$C_4H_8O_2$	*o*-nitrotoluene	-5.76	-6.92
0135met	$C_7H_7NO_2$	*o*-nitrotoluene	-8.04	-8.15
0215pbr	C_6H_5OBr	*o*-nitrotoluene	-9.57	-8.96
0008noc	C_8H_{18}	nitroethane	-3.89	-3.89
0036tol	C_7H_8	nitroethane	-4.88	-4.18
0045eth	C_2H_6O	nitroethane	-3.98	-3.76
0062dio	$C_4H_8O_2$	nitroethane	-5.28	-5.33
0076but	C_4H_8O	nitroethane	-4.73	-4.70
0130nit	$C_2H_5NO_2$	nitroethane	-5.53	-5.65
0506nit	CH_3NO_2	nitroethane	-5.35	-4.61
0044met	CH_4O	nitrobenzene	-2.93	-3.38
0053phe	C_6H_6O	nitrobenzene	-7.86	-7.11
0055ocr	C_7H_8O	nitrobenzene	-8.16	-7.38
0056mcr	C_7H_8O	nitrobenzene	-7.29	-7.41
0057pcr	C_7H_8O	nitrobenzene	-8.13	-7.44
0086eth	$C_2H_4O_2$	nitrobenzene	-4.78	-6.05
0087pro	$C_3H_6O_2$	nitrobenzene	-5.38	-6.42
0088but	$C_4H_8O_2$	nitrobenzene	-5.84	-6.90
0089pen	$C_5H_{10}O_2$	nitrobenzene	-6.47	-7.43
0090hex	$C_6H_{12}O_2$	nitrobenzene	-7.26	-8.00
0118ani	C_6H_7N	nitrobenzene	-7.15	-7.07
0134nit	$C_6H_5NO_2$	nitrobenzene	-7.94	-7.44
0215pbr	C_6H_5OBr	nitrobenzene	-9.76	-8.93
0506nit	CH_3NO_2	nitrobenzene	-4.90	-4.34
n017	H_2O_2	nitrobenzene	-5.45	-5.66
i059	$C_2H_3O_2^-$	acetonitrile	-58.80	-60.54
i064	$C_7H_5O_2^-$	acetonitrile	-55.40	-56.00
i074	$C_6H_5O^-$	acetonitrile	-55.10	-56.84
i101	$C_6H_4NO_3^-$	acetonitrile	-46.70	-46.98
i102	$C_6H_4NO_3^-$	acetonitrile	-45.00	-45.71
i115	Cl^-	acetonitrile	-62.40	-62.12
i116	Br^-	acetonitrile	-59.30	-59.15
i119	$C_2H_2O_2Cl^-$	acetonitrile	-54.60	-55.18
i120	$C_2HO_2Cl_2^-$	acetonitrile	-51.20	-51.54
i189	$C_{10}H_{11}O_2^-$	acetonitrile	-54.40	-54.88
i191	$C_6H_3N_2O_5^-$	acetonitrile	-36.00	-36.74
i194	$C_7H_4O_2Cl^-$	acetonitrile	-53.50	-53.49

i199	$C_7H_5O_3^-$	acetonitrile	-46.50	-46.72
i205	$C_7H_4NO_4^-$	acetonitrile	-51.90	-52.09
i208	$C_6H_2OCl_3^-$	acetonitrile	-43.80	-43.76
i209	$C_9H_9O_2^-$	acetonitrile	-59.70	-59.91
i215	$C_6H_4OCl^-$	acetonitrile	-50.60	-51.10
i220	$C_7H_4NO_4^-$	acetonitrile	-48.30	-49.17
i222	$C_7H_4OF_3^-$	acetonitrile	-46.90	-46.72
i227	$C_7H_4NO^-$	acetonitrile	-46.50	-46.94
i231	$C_7H_5O_3^-$	acetonitrile	-53.80	-55.03
i234	$C_7H_4NO_4^-$	acetonitrile	-48.00	-48.42
i240	$C_6H_6NO_2S^-$	acetonitrile	-55.40	-55.23
i243	$C_4H_7O_2^-$	acetonitrile	-56.70	-59.14
i245	$C_3H_2NO_2^-$	acetonitrile	-50.80	-51.10
i247	$C_7H_{11}O_2^-$	acetonitrile	-55.60	-56.48
i255	$C_2H_3O_3^-$	acetonitrile	-54.70	-54.48
i261	$CH_3O_3S^-$	acetonitrile	-54.00	-54.22
i263	NO_3^-	acetonitrile	-51.00	-52.10
i277	$C_2O_2F_3^-$	acetonitrile	-45.60	-45.84
0008noc	C_8H_{18}	acetonitrile	-3.57	-4.03
0036tol	C_7H_8	acetonitrile	-4.68	-4.44
0045eth	C_2H_6O	acetonitrile	-4.43	-4.14
0062dio	$C_4H_8O_2$	acetonitrile	-5.33	-5.75
0076but	C_4H_8O	acetonitrile	-4.73	-5.04
0126eth	C_2H_3N	acetonitrile	-4.85	-4.33
0506nit	CH_3NO_2	acetonitrile	-5.62	-4.93
i003	CH_6N^+	acetonitrile	-80.20	-79.26
i004	$C_3H_{10}N^+$	acetonitrile	-75.70	-76.31
i006	$C_4H_{12}N^+$	acetonitrile	-70.90	-70.80
i010	$C_4H_{12}N^+$	acetonitrile	-67.50	-67.00
i013	$C_3H_{10}N^+$	acetonitrile	-66.10	-65.22
i014	$C_6H_{16}N^+$	acetonitrile	-59.80	-59.16
i015	$C_9H_{22}N^+$	acetonitrile	-57.90	-57.57
i018	$C_6H_8N^+$	acetonitrile	-76.80	-76.81
i021	$C_7H_{10}N^+$	acetonitrile	-74.40	-75.01
i032	$C_4H_{10}N^+$	acetonitrile	-71.00	-71.51
i033	$C_5H_{12}N^+$	acetonitrile	-69.20	-69.02
i036	$C_5H_6N^+$	acetonitrile	-66.70	-65.63
i047	H_4N^+	acetonitrile	-89.30	-88.96
i048	$H_5N_2^+$	acetonitrile	-87.70	-88.11
i050	CH_5O^+	acetonitrile	-92.10	-92.94
i053	$C_4H_{11}O^+$	acetonitrile	-71.00	-71.16
i054	$C_3H_7O^+$	acetonitrile	-77.50	-78.20
i056	$C_8H_9O^+$	acetonitrile	-69.20	-69.34
i095	$C_4H_{10}NO^+$	acetonitrile	-74.10	-73.96
i099	$C_7H_8NO^+$	acetonitrile	-69.70	-70.15
i112	$C_2H_7OS^+$	acetonitrile	-73.50	-73.80
i132	$C_7H_{10}N^+$	acetonitrile	-61.90	-62.12
i150	$C_8H_9O_2^+$	acetonitrile	-75.40	-74.93
i154	$C_2H_5O_2^+$	acetonitrile	-85.90	-86.45
i157	$C_7H_7O_2^+$	acetonitrile	-78.30	-77.83
i158	$C_7H_{10}N^+$	acetonitrile	-78.20	-77.77
i159	$C_8H_{20}N^+$	acetonitrile	-65.40	-65.18
i161	$C_8H_{20}N^+$	acetonitrile	-64.80	-64.74
i163	$C_2H_8N^+$	acetonitrile	-76.80	-76.77
i164	H_3S^+	acetonitrile	-100.20	-100.05
i165	$C_4H_{12}N^+$	acetonitrile	-73.50	-73.69
i167	$C_7H_9O^+$	acetonitrile	-70.40	-70.43
i175	$C_4H_{11}O^+$	acetonitrile	-85.20	-85.02
i176	$C_4H_{12}N^+$	acetonitrile	-75.70	-75.29
i178	$C_6H_7O^+$	acetonitrile	-78.10	-78.10
i179	$C_4H_{11}O^+$	acetonitrile	-82.40	-82.15
i180	$C_4H_9O^+$	acetonitrile	-74.70	-74.81
i181	$C_2H_6NS^+$	acetonitrile	-73.60	-74.09
i184	$C_{12}H_{28}N^+$	acetonitrile	-57.40	-57.18
0008noc	C_8H_{18}	nitromethane	-3.15	-3.70
0036tol	C_7H_8	nitromethane	-4.52	-4.20
0045eth	C_2H_6O	nitromethane	-4.16	-3.94
0062dio	$C_4H_8O_2$	nitromethane	-5.46	-5.52
0076but	C_4H_8O	nitromethane	-4.72	-4.71
0506nit	CH_3NO_2	nitromethane	-5.38	-4.73
0648gbu	$C_4H_6O_2$	nitromethane	-5.45	-5.45
0008noc	C_8H_{18}	dimethyl formamide	-3.77	-3.66
0036tol	C_7H_8	dimethyl formamide	-4.88	-3.95
0045eth	C_2H_6O	dimethyl formamide	-5.23	-3.87
0062dio	$C_4H_8O_2$	dimethyl formamide	-5.03	-5.26
0076but	C_4H_8O	dimethyl formamide	-4.56	-4.66

0506nit	CH_3NO_2	dimethyl formamide	-5.66	-4.59
0515dim	C_3H_7NO	dimethyl formamide	-6.47	-5.89
0008noc	C_8H_{18}	dimethyl acetamide	-3.94	-3.73
0036tol	C_7H_8	dimethyl acetamide	-4.94	-3.87
0045eth	C_2H_6O	dimethyl acetamide	-5.40	-3.98
0062dio	$C_4H_8O_2$	dimethyl acetamide	-5.01	-5.26
0076but	C_4H_8O	dimethyl acetamide	-4.52	-4.54
0506nit	CH_3NO_2	dimethyl acetamide	-5.62	-4.55
0519dim	C_4H_9NO	dimethyl acetamide	-6.77	-6.47
0008noc	C_8H_{18}	sulfolane	-2.44	-3.50
0036tol	C_7H_8	sulfolane	-4.23	-3.49
0045eth	C_2H_6O	sulfolane	-4.30	-4.00
0062dio	$C_4H_8O_2$	sulfolane	-4.90	-5.20
0076but	C_4H_8O	sulfolane	-4.09	-4.26
0110but	$C_4H_{11}N$	sulfolane	-4.25	-4.29
0506nit	CH_3NO_2	sulfolane	-5.28	-4.27
i043	CHN_2^-	dimethyl sulfoxide	-59.50	-59.77
i044	$C_6H_6N^-$	dimethyl sulfoxide	-52.90	-52.79
i045	$C_{12}H_{10}N^-$	dimethyl sulfoxide	-47.70	-47.38
i046	CN^-	dimethyl sulfoxide	-54.00	-54.32
i059	$C_2H_3O_2^-$	dimethyl sulfoxide	-59.20	-59.64
i064	$C_7H_5O_2^-$	dimethyl sulfoxide	-55.60	-55.48
i065	CH_3O^-	dimethyl sulfoxide	-67.60	-67.61
i066	$C_2H_5O^-$	dimethyl sulfoxide	-64.60	-64.94
i068	$C_3H_7O^-$	dimethyl sulfoxide	-60.00	-61.04
i070	$C_4H_9O^-$	dimethyl sulfoxide	-56.10	-56.96
i074	$C_6H_5O^-$	dimethyl sulfoxide	-54.20	-54.11
i085	$C_3H_5O^-$	dimethyl sulfoxide	-60.40	-61.09
i086	$C_5H_9O^-$	dimethyl sulfoxide	-59.00	-59.60
i089	HO^-	dimethyl sulfoxide	-77.00	-76.83
i102	$C_6H_4NO_3^-$	dimethyl sulfoxide	-45.40	-45.84
i103	$CH_2NO_2^-$	dimethyl sulfoxide	-61.30	-61.82
i104	$C_6H_5N_2O_2^-$	dimethyl sulfoxide	-47.40	-47.90
i105	$C_2H_4NO^-$	dimethyl sulfoxide	-58.00	-58.01
i110	$C_6H_5S^-$	dimethyl sulfoxide	-53.80	-53.85
i113	$C_2H_5OS^-$	dimethyl sulfoxide	-55.40	-55.70
i115	Cl^-	dimethyl sulfoxide	-62.70	-62.55
i116	Br^-	dimethyl sulfoxide	-57.80	-57.88
i120	$C_2HO_2Cl_2^-$	dimethyl sulfoxide	-49.20	-50.12
i121	$C_2H_2OF_3^-$	dimethyl sulfoxide	-56.10	-56.42
i186	$C_2H_2N_3^-$	dimethyl sulfoxide	-56.70	-56.65
i187	$C_2H_2N_3^-$	dimethyl sulfoxide	-57.20	-57.25
i191	$C_6H_3N_2O_5^-$	dimethyl sulfoxide	-38.00	-38.08
i194	$C_7H_4O_2Cl^-$	dimethyl sulfoxide	-53.60	-53.69
i200	$C_9H_7O^-$	dimethyl sulfoxide	-58.70	-58.39
i201	$C_3H_6NO_2^-$	dimethyl sulfoxide	-59.50	-59.47
i204	$C_{10}H_7O^-$	dimethyl sulfoxide	-51.80	-52.62
i205	$C_7H_4NO_4^-$	dimethyl sulfoxide	-52.60	-52.51
i206	$C_3H_6NO_2^-$	dimethyl sulfoxide	-59.90	-60.73
i209	$C_9H_9O_2^-$	dimethyl sulfoxide	-59.80	-59.34
i211	$C_7H_6NO_2^-$	dimethyl sulfoxide	-59.50	-60.25
i212	$C_5H_5N_2^-$	dimethyl sulfoxide	-51.50	-51.63
i218	$C_7H_5O_3^-$	dimethyl sulfoxide	-56.50	-57.40
i219	$C_8H_7O_2^-$	dimethyl sulfoxide	-56.10	-57.45
i225	$C_5H_5N_2^-$	dimethyl sulfoxide	-51.30	-50.95
i226	$C_7H_4O_2Cl^-$	dimethyl sulfoxide	-52.60	-52.79
i234	$C_7H_4NO_4^-$	dimethyl sulfoxide	-48.20	-48.89
i237	$C_5H_4N_5^-$	dimethyl sulfoxide	-56.40	-56.66
i238	$C_7H_6NO^-$	dimethyl sulfoxide	-55.60	-55.75
i239	$C_8H_8NO^-$	dimethyl sulfoxide	-54.00	-53.96
i240	$C_6H_6NO_2S^-$	dimethyl sulfoxide	-54.60	-54.57
i241	$C_9H_9O^-$	dimethyl sulfoxide	-55.40	-55.33
i244	$C_{12}H_8N^-$	dimethyl sulfoxide	-49.90	-50.13
i248	$C_5H_5^-$	dimethyl sulfoxide	-56.10	-56.16
i251	$C_2H_5O_2S^-$	dimethyl sulfoxide	-56.90	-56.52
i252	$C_2H_4NS^-$	dimethyl sulfoxide	-54.90	-54.58
i254	CH_2NO^-	dimethyl sulfoxide	-57.60	-57.56
i257	$C_3H_3N_2^-$	dimethyl sulfoxide	-56.70	-56.42
i258	$C_3HN_2^-$	dimethyl sulfoxide	-46.00	-46.31
i260	$CH_4NO_2S^-$	dimethyl sulfoxide	-57.50	-57.41
i262	$C_4H_9S^-$	dimethyl sulfoxide	-55.70	-56.56
i265	NO_2^-	dimethyl sulfoxide	-54.50	-55.18
i267	$C_7H_7O^-$	dimethyl sulfoxide	-53.90	-54.86
i270	$C_8H_5^-$	dimethyl sulfoxide	-56.40	-56.37
i271	$C_9H_5N_2^-$	dimethyl sulfoxide	-41.00	-41.60
i272	$C_3H_3N_2^-$	dimethyl sulfoxide	-54.20	-54.26

i274	$C_4H_4NO_2^-$	dimethyl sulfoxide	-58.60	-58.79
i275	CHN_4^-	dimethyl sulfoxide	-54.50	-54.45
i276	$C_2HNOF_3^-$	dimethyl sulfoxide	-49.20	-49.69
i277	$C_2O_2F_3^-$	dimethyl sulfoxide	-45.00	-45.24
i279	$CH_3N_2O^-$	dimethyl sulfoxide	-58.90	-58.43
i280	$C_9H_6NO^-$	dimethyl sulfoxide	-52.90	-53.18
0008noc	C_8H_{18}	dimethyl sulfoxide	-2.84	-3.65
0036tol	C_7H_8	dimethyl sulfoxide	-4.42	-3.88
0045eth	C_2H_6O	dimethyl sulfoxide	-5.25	-4.14
0062dio	$C_4H_8O_2$	dimethyl sulfoxide	-4.90	-5.44
0076but	C_4H_8O	dimethyl sulfoxide	-4.23	-4.72
0503dim	C_2H_6OS	dimethyl sulfoxide	-7.63	-6.05
0506nit	CH_3NO_2	dimethyl sulfoxide	-5.66	-4.60
i003	CH_6N^+	dimethyl sulfoxide	-82.40	-83.41
i018	$C_6H_8N^+$	dimethyl sulfoxide	-79.80	-79.91
i036	$C_5H_6N^+$	dimethyl sulfoxide	-67.20	-67.38
i047	H_4N^+	dimethyl sulfoxide	-93.90	-94.15
0008noc	C_8H_{18}	methyl formamide	-3.34	-3.56
0036tol	C_7H_8	methyl formamide	-4.34	-3.52
0045eth	C_2H_6O	methyl formamide	-5.12	-4.19
0062dio	$C_4H_8O_2$	methyl formamide	-4.86	-4.89
0076but	C_4H_8O	methyl formamide	-4.34	-4.70
0506nit	CH_3NO_2	methyl formamide	-5.11	-4.43
0517met	C_2H_5NO	methyl formamide	-8.27	-6.00

Table S9. Nonpolar solvents

Solute MNSol code[1]	Solute formula	Solvent	$\Delta^{ref}G^{\circ}_{solv}$	$\Delta^{calc}G^{\circ}_{solv}$
0008noc	C_8H_{18}	acetic acid	-3.93	-4.38
0036tol	C_7H_8	acetic acid	-4.53	-4.61
0045eth	C_2H_6O	acetic acid	-5.25	-3.45
0062dio	$C_4H_8O_2$	acetic acid	-5.80	-5.28
0076but	C_4H_8O	acetic acid	-4.80	-4.42
0086eth	$C_2H_4O_2$	acetic acid	-5.30	-5.16
0506nit	CH_3NO_2	acetic acid	-4.88	-4.32
0008noc	C_8H_{18}	aniline	-3.48	-4.43
0036tol	C_7H_8	aniline	-4.57	-4.25
0045eth	C_2H_6O	aniline	-4.45	-3.53
0062dio	$C_4H_8O_2$	aniline	-5.65	-5.13
0076but	C_4H_8O	aniline	-4.87	-4.42
0086eth	$C_2H_4O_2$	aniline	-6.30	-5.63
0087pro	$C_3H_6O_2$	aniline	-6.20	-6.16
0118ani	C_6H_7N	aniline	-7.61	-6.87
0506nit	CH_3NO_2	aniline	-5.11	-4.18
n017	H_2O_2	aniline	-7.80	-5.28
0008noc	C_8H_{18}	anisole	-4.62	-4.64
0036tol	C_7H_8	anisole	-4.95	-4.25
0045eth	C_2H_6O	anisole	-3.59	-3.25
0062dio	$C_4H_8O_2$	anisole	-5.06	-4.82
0068ani	C_7H_8O	anisole	-6.33	-6.14
0076but	C_4H_8O	anisole	-4.43	-4.12
0110but	$C_4H_{11}N$	anisole	-4.44	-4.49
0506nit	CH_3NO_2	anisole	-4.69	-3.84
0005npe	C_5H_{12}	benzene	-2.99	-3.19
0006nhe	C_6H_{14}	benzene	-3.62	-3.98
0008noc	C_8H_{18}	benzene	-5.35	-5.40
0018cyc	C_6H_{12}	benzene	-4.05	-3.95
0035ben	C_6H_6	benzene	-4.55	-3.71
0036tol	C_7H_8	benzene	-5.32	-4.39
0044met	CH_4O	benzene	-2.58	-2.00
0045eth	C_2H_6O	benzene	-3.42	-2.59
0047pro	C_3H_8O	benzene	-3.87	-3.30
0048pro	C_3H_8O	benzene	-3.48	-2.97
0049but	$C_4H_{10}O$	benzene	-4.45	-4.00
0050met	$C_4H_{10}O$	benzene	-3.70	-3.10
0052pen	$C_5H_{12}O$	benzene	-5.10	-4.74
0053phe	C_6H_6O	benzene	-7.12	-5.96
0054hex	$C_6H_{14}O$	benzene	-6.13	-5.43
0055ocr	C_7H_8O	benzene	-7.44	-6.65
0056mcr	C_7H_8O	benzene	-6.66	-6.59
0057pcr	C_7H_8O	benzene	-7.35	-6.60
0058hep	$C_7H_{16}O$	benzene	-6.85	-6.13
0062dio	$C_4H_8O_2$	benzene	-5.21	-4.21
0075pro	C_3H_6O	benzene	-3.79	-3.01
0076but	C_4H_8O	benzene	-4.46	-3.91
0078pen	$C_5H_{10}O$	benzene	-5.14	-4.63
0080hex	$C_6H_{12}O$	benzene	-5.76	-5.31
0082hep	$C_7H_{14}O$	benzene	-6.36	-6.01
0086eth	$C_2H_4O_2$	benzene	-4.02	-4.07
0087pro	$C_3H_6O_2$	benzene	-4.75	-4.86
0088but	$C_4H_8O_2$	benzene	-5.30	-5.34
0089pen	$C_5H_{10}O_2$	benzene	-6.01	-5.87
0090hex	$C_6H_{12}O_2$	benzene	-6.94	-6.43
0093met	$C_3H_6O_2$	benzene	-4.04	-3.74
0094met	$C_4H_8O_2$	benzene	-4.58	-4.49
0095eth	$C_4H_8O_2$	benzene	-4.53	-4.36
0097pro	$C_5H_{10}O_2$	benzene	-5.21	-4.95
0098met	$C_6H_{12}O_2$	benzene	-5.83	-5.74
0099but	$C_6H_{12}O_2$	benzene	-5.78	-5.60
0100met	$C_7H_{14}O_2$	benzene	-6.47	-6.41
0101pen	$C_7H_{14}O_2$	benzene	-6.53	-6.27
0103eth	C_2H_7N	benzene	-2.73	-2.40
0104dim	C_2H_7N	benzene	-3.01	-2.32
0106pro	C_3H_9N	benzene	-3.68	-3.16
0107tri	C_3H_9N	benzene	-2.80	-2.66
0111die	$C_4H_{11}N$	benzene	-4.02	-3.48
0115dip	$C_6H_{15}N$	benzene	-5.09	-4.90
0116pyr	C_5H_5N	benzene	-5.28	-4.46
0118ani	C_6H_7N	benzene	-6.88	-5.76
0119met	C_6H_7N	benzene	-5.86	-4.99
0121met	C_6H_7N	benzene	-6.17	-5.13
0122Nme	C_7H_9N	benzene	-6.64	-6.00
0125dim	C_7H_9N	benzene	-6.39	-5.29
0134nit	$C_6H_5NO_2$	benzene	-7.60	-6.74
0150mhy	$C_7H_6O_2$	benzene	-9.29	-8.56
0151phy	$C_7H_6O_2$	benzene	-9.73	-8.54
0215pbr	C_6H_5OBr	benzene	-8.81	-7.94
0216amm	H_3N	benzene	-1.12	-1.74
0217wat	H_2O	benzene	-1.71	-3.08

0220tri	$C_3H_9O_4P$	benzene	-8.02	-6.81
0221tri	$C_6H_{15}O_4P$	benzene	-8.58	-7.47
0222tri	$C_9H_{21}O_4P$	benzene	-9.34	-8.57
0225pipa	$C_5H_{11}N$	benzene	-5.03	-3.82
0228met	CH_5N	benzene	-2.66	-1.89
0229hyd	H_4N_2	benzene	-4.02	-3.99
0236oct	$C_8H_{18}O$	benzene	-8.06	-6.85
0240met	$C_8H_8O_2$	benzene	-6.27	-7.77
0401amia	$C_9H_{12}N_2O$	benzene	-12.18	-10.39
0433pho	$C_4H_7O_4PCl_2$	benzene	-9.09	-7.85
0441pho	$C_8H_{10}NO_5PS$	benzene	-9.21	-8.60
0447pho	$C_{10}H_{14}NO_5PS$	benzene	-8.58	-9.07
0506nit	CH_3NO_2	benzene	-4.50	-3.13
0571dim	C_7H_9N	benzene	-7.64	-5.68
n008	C_7H_7NO	benzene	-9.93	-8.65
n009	C_7H_9N	benzene	-7.37	-6.44
n010	C_7H_9N	benzene	-7.61	-6.47
n011	C_7H_9N	benzene	-7.59	-6.50
n017	H_2O_2	benzene	-4.77	-3.64
0008noc	C_8H_{18}	bromobenzene	-5.02	-4.58
0036tol	C_7H_8	bromobenzene	-5.13	-4.30
0044met	CH_4O	bromobenzene	-2.31	-2.83
0045eth	C_2H_6O	bromobenzene	-3.26	-3.42
0047pro	C_3H_8O	bromobenzene	-3.74	-4.05
0049but	$C_4H_{10}O$	bromobenzene	-4.08	-4.74
0052pen	$C_5H_{12}O$	bromobenzene	-5.06	-5.54
0053phe	C_6H_6O	bromobenzene	-6.87	-6.87
0054hex	$C_6H_{14}O$	bromobenzene	-5.92	-6.29
0055ocr	C_7H_8O	bromobenzene	-7.26	-7.37
0057pcr	C_7H_8O	bromobenzene	-7.12	-7.42
0058hep	$C_7H_{16}O$	bromobenzene	-6.68	-6.91
0062dio	$C_4H_8O_2$	bromobenzene	-5.02	-5.03
0076but	C_4H_8O	bromobenzene	-4.37	-4.37
0093met	$C_3H_6O_2$	bromobenzene	-3.87	-4.44
0095eth	$C_4H_8O_2$	bromobenzene	-4.57	-4.76
0097pro	$C_5H_{10}O_2$	bromobenzene	-4.93	-5.41
0099but	$C_6H_{12}O_2$	bromobenzene	-5.58	-6.16
0101pen	$C_7H_{14}O_2$	bromobenzene	-6.35	-6.93
0103eth	C_2H_7N	bromobenzene	-2.73	-2.84
0106pro	C_3H_9N	bromobenzene	-3.57	-3.70
0110but	$C_4H_{11}N$	bromobenzene	-4.22	-4.57
0118ani	C_6H_7N	bromobenzene	-6.66	-6.78
0186bro	C_6H_5Br	bromobenzene	-6.25	-5.83
0215pbr	C_6H_5OBr	bromobenzene	-8.49	-8.91
0506nit	CH_3NO_2	bromobenzene	-4.25	-4.04
n011	C_7H_9N	bromobenzene	-7.59	-7.32
0044met	CH_4O	bromoform	-2.79	-3.28
0045eth	C_2H_6O	bromoform	-3.24	-3.79
0047pro	C_3H_8O	bromoform	-4.03	-4.40
0049but	$C_4H_{10}O$	bromoform	-4.72	-5.05
0052pen	$C_5H_{12}O$	bromoform	-5.34	-5.84
0053phe	C_6H_6O	bromoform	-6.88	-6.90
0054hex	$C_6H_{14}O$	bromoform	-6.20	-6.68
0055ocr	C_7H_8O	bromoform	-7.45	-7.52
0058hep	$C_7H_{16}O$	bromoform	-7.10	-7.44
0086eth	$C_2H_4O_2$	bromoform	-4.54	-5.04
0179tri	$CHBr_3$	bromoform	-6.21	-6.64
0215pbr	C_6H_5OBr	bromoform	-8.49	-9.03
0093met	$C_3H_6O_2$	bromooctane	-3.35	-4.13
0095eth	$C_4H_8O_2$	bromooctane	-3.97	-4.41
0097pro	$C_5H_{10}O_2$	bromooctane	-4.48	-5.06
0099but	$C_6H_{12}O_2$	bromooctane	-5.11	-5.81
0101pen	$C_7H_{14}O_2$	bromooctane	-5.81	-6.57
0041nap	$C_{10}H_8$	butyl acetate	-7.59	-7.09
0044met	CH_4O	butyl acetate	-3.04	-2.82
0045eth	C_2H_6O	butyl acetate	-3.97	-3.38
0046eth	$C_2H_6O_2$	butyl acetate	-6.27	-6.17
0047pro	C_3H_8O	butyl acetate	-4.52	-3.97
0049but	$C_4H_{10}O$	butyl acetate	-5.23	-4.66
0052pen	$C_5H_{12}O$	butyl acetate	-5.78	-5.46
0053phe	C_6H_6O	butyl acetate	-8.96	-6.91
0054hex	$C_6H_{14}O$	butyl acetate	-6.62	-6.19
0055ocr	C_7H_8O	butyl acetate	-8.90	-7.31
0056mcr	C_7H_8O	butyl acetate	-8.44	-7.33
0057pcr	C_7H_8O	butyl acetate	-9.28	-7.35
0058hep	$C_7H_{16}O$	butyl acetate	-7.14	-6.84
0086eth	$C_2H_4O_2$	butyl acetate	-6.11	-5.45
0099but	$C_6H_{12}O_2$	butyl acetate	-5.52	-6.01
0116pyr	C_5H_5N	butyl acetate	-5.31	-5.18
0118ani	C_6H_7N	butyl acetate	-7.30	-6.91
0215pbr	C_6H_5OBr	butyl acetate	-10.57	-8.95
0217wat	H_2O	butyl acetate	-4.13	-4.83
0236oct	$C_8H_{18}O$	butyl acetate	-8.17	-7.45

n011	C_7H_9N	butyl acetate	-7.81	-7.45
n017	H_2O_2	butyl acetate	-6.76	-5.09
0053phe	C_6H_6O	butylbenzene	-6.76	-5.95
0078pen	$C_5H_{10}O$	butylbenzene	-4.74	-4.04
0080hex	$C_6H_{12}O$	butylbenzene	-5.31	-4.72
0081dim	$C_6H_{12}O$	butylbenzene	-4.77	-4.41
0082hep	$C_7H_{14}O$	butylbenzene	-5.93	-5.42
0094met	$C_4H_8O_2$	butylbenzene	-4.19	-3.89
0098met	$C_6H_{12}O_2$	butylbenzene	-5.37	-5.15
0099but	$C_6H_{12}O_2$	butylbenzene	-5.28	-5.01
0100met	$C_7H_{14}O_2$	butylbenzene	-6.09	-5.82
0529but	$C_{10}H_{14}$	butylbenzene	-6.86	-6.44
0008noc	C_8H_{18}	carbon disulfide	-5.68	-5.52
0036tol	C_7H_8	carbon disulfide	-5.39	-4.88
0045eth	C_2H_6O	carbon disulfide	-2.72	-3.10
0053phe	C_6H_6O	carbon disulfide	-6.27	-6.39
0062dio	$C_4H_8O_2$	carbon disulfide	-4.67	-4.87
0075pro	C_3H_6O	carbon disulfide	-3.14	-3.50
0076but	C_4H_8O	carbon disulfide	-3.85	-4.19
0086eth	$C_2H_4O_2$	carbon disulfide	-2.98	-4.21
0093met	$C_3H_6O_2$	carbon disulfide	-3.67	-4.17
0095eth	$C_4H_8O_2$	carbon disulfide	-4.08	-4.86
0097pro	$C_5H_{10}O_2$	carbon disulfide	-4.63	-5.50
0131nit	$C_3H_7NO_2$	carbon disulfide	-4.50	-5.54
0506nit	CH_3NO_2	carbon disulfide	-3.30	-3.75
0537car	CS_2	carbon disulfide	-3.95	-4.72
n017	H_2O_2	carbon disulfide	-3.14	-3.74
0008noc	C_8H_{18}	carbon tetrachloride	-5.39	-5.50
0024met	C_4H_8	carbon tetrachloride	-2.63	-2.34
0025buta	C_4H_8	carbon tetrachloride	-2.48	-2.58
0028Epe	C_5H_{10}	carbon tetrachloride	-3.46	-3.30
0035ben	C_6H_6	carbon tetrachloride	-4.50	-3.76
0036tol	C_7H_8	carbon tetrachloride	-5.12	-4.43
0037eth	C_8H_{10}	carbon tetrachloride	-5.67	-5.20
0038oxy	C_8H_{10}	carbon tetrachloride	-6.07	-5.12
0039mxy	C_8H_{10}	carbon tetrachloride	-5.71	-5.09
0041nap	$C_{10}H_8$	carbon tetrachloride	-7.55	-6.69
0044met	CH_4O	carbon tetrachloride	-2.25	-1.88
0045eth	C_2H_6O	carbon tetrachloride	-2.96	-2.49
0047pro	C_3H_8O	carbon tetrachloride	-3.64	-3.24
0048pro	C_3H_8O	carbon tetrachloride	-3.15	-2.93
0049but	$C_4H_{10}O$	carbon tetrachloride	-4.20	-4.03
0050met	$C_4H_{10}O$	carbon tetrachloride	-3.40	-3.06
0052pen	$C_5H_{12}O$	carbon tetrachloride	-4.73	-4.79
0053phe	C_6H_6O	carbon tetrachloride	-6.14	-5.89
0054hex	$C_6H_{14}O$	carbon tetrachloride	-5.04	-5.53
0055ocr	C_7H_8O	carbon tetrachloride	-6.51	-6.60
0057pcr	C_7H_8O	carbon tetrachloride	-6.32	-6.53
0058hep	$C_7H_{16}O$	carbon tetrachloride	-6.49	-6.24
0062dio	$C_4H_8O_2$	carbon tetrachloride	-4.97	-4.23
0068ani	C_7H_8O	carbon tetrachloride	-5.49	-5.77
0074ben	C_7H_6O	carbon tetrachloride	-6.11	-6.27
0075pro	C_3H_6O	carbon tetrachloride	-3.35	-2.98
0076but	C_4H_8O	carbon tetrachloride	-4.09	-3.86
0077cyc	C_5H_8O	carbon tetrachloride	-5.26	-4.65
0078pen	$C_5H_{10}O$	carbon tetrachloride	-4.81	-4.74
0080hex	$C_6H_{12}O$	carbon tetrachloride	-5.47	-5.43
0082hep	$C_7H_{14}O$	carbon tetrachloride	-6.12	-6.12
0084met	C_8H_8O	carbon tetrachloride	-7.10	-7.05
0086eth	$C_2H_4O_2$	carbon tetrachloride	-3.64	-3.83
0087pro	$C_3H_6O_2$	carbon tetrachloride	-4.09	-4.66
0088but	$C_4H_8O_2$	carbon tetrachloride	-4.81	-5.28
0090hex	$C_6H_{12}O_2$	carbon tetrachloride	-6.99	-6.47
0093met	$C_3H_6O_2$	carbon tetrachloride	-3.82	-3.71
0094met	$C_4H_8O_2$	carbon tetrachloride	-4.43	-4.60
0095eth	$C_4H_8O_2$	carbon tetrachloride	-4.40	-4.45
0097pro	$C_5H_{10}O_2$	carbon tetrachloride	-5.03	-5.06
0098met	$C_6H_{12}O_2$	carbon tetrachloride	-5.71	-5.85
0099but	$C_6H_{12}O_2$	carbon tetrachloride	-5.59	-5.71
0100met	$C_7H_{14}O_2$	carbon tetrachloride	-6.39	-6.51
0101pen	$C_7H_{14}O_2$	carbon tetrachloride	-6.35	-6.38
0103eth	C_2H_7N	carbon tetrachloride	-2.77	-2.33
0104dim	C_2H_7N	carbon tetrachloride	-2.75	-2.33
0106pro	C_3H_9N	carbon tetrachloride	-3.59	-3.05
0107tri	C_3H_9N	carbon tetrachloride	-3.09	-2.78
0110but	$C_4H_{11}N$	carbon tetrachloride	-5.35	-3.81
0111die	$C_4H_{11}N$	carbon tetrachloride	-4.12	-3.45
0116pyr	C_5H_5N	carbon tetrachloride	-5.01	-4.41
0118ani	C_6H_7N	carbon tetrachloride	-6.10	-5.68
0122Nme	C_7H_9N	carbon tetrachloride	-6.58	-5.92
0129ben	C_7H_5N	carbon tetrachloride	-6.28	-5.90
0131nit	$C_3H_7NO_2$	carbon tetrachloride	-4.49	-4.83
0134nit	$C_6H_5NO_2$	carbon tetrachloride	-6.92	-6.86

0135met	$C_7H_7NO_2$	carbon tetrachloride	-7.49	-7.51
0144thi	C_7H_8S	carbon tetrachloride	-5.66	-6.77
0151phy	$C_7H_6O_2$	carbon tetrachloride	-8.16	-8.57
0157flu	C_6H_5F	carbon tetrachloride	-3.64	-4.16
0174chl	C_6H_5Cl	carbon tetrachloride	-5.21	-4.98
0176pdi	$C_6H_4Cl_2$	carbon tetrachloride	-6.28	-6.18
0186bro	C_6H_5Br	carbon tetrachloride	-5.85	-5.56
0215pbr	C_6H_5OBr	carbon tetrachloride	-7.86	-7.88
0216amm	H_3N	carbon tetrachloride	-1.06	-1.61
0217wat	H_2O	carbon tetrachloride	-0.85	-2.92
0220tri	$C_3H_9O_4P$	carbon tetrachloride	-7.24	-7.06
0221tri	$C_6H_{15}O_4P$	carbon tetrachloride	-7.51	-7.96
0222tri	$C_9H_{21}O_4P$	carbon tetrachloride	-8.60	-9.01
0228met	CH_5N	carbon tetrachloride	-2.53	-1.87
0240met	$C_8H_8O_2$	carbon tetrachloride	-7.19	-7.76
0506nit	CH_3NO_2	carbon tetrachloride	-3.52	-3.12
0525car	C_1Cl_4	carbon tetrachloride	-4.35	-4.28
n008	C_7H_7NO	carbon tetrachloride	-9.13	-8.64
n009	C_7H_9N	carbon tetrachloride	-7.16	-6.35
n010	C_7H_9N	carbon tetrachloride	-7.23	-6.38
n011	C_7H_9N	carbon tetrachloride	-7.24	-6.41
n017	H_2O_2	carbon tetrachloride	-3.14	-3.39
test4001	C_6H_5I	carbon tetrachloride	-6.50	-7.01
0008noc	C_8H_{18}	chlorobenzene	-5.16	-4.61
0036tol	C_7H_8	chlorobenzene	-5.18	-4.35
0044met	CH_4O	chlorobenzene	-2.44	-2.85
0045eth	C_2H_6O	chlorobenzene	-3.30	-3.48
0047pro	C_3H_8O	chlorobenzene	-3.82	-4.10
0049but	$C_4H_{10}O$	chlorobenzene	-4.31	-4.80
0052pen	$C_5H_{12}O$	chlorobenzene	-5.25	-5.61
0053phe	C_6H_6O	chlorobenzene	-6.96	-6.95
0054hex	$C_6H_{14}O$	chlorobenzene	-5.98	-6.36
0055ocr	C_7H_8O	chlorobenzene	-7.33	-7.45
0057pcr	C_7H_8O	chlorobenzene	-7.23	-7.50
0058hep	$C_7H_{16}O$	chlorobenzene	-6.78	-6.99
0062dio	$C_4H_8O_2$	chlorobenzene	-5.08	-5.09
0075pro	C_3H_6O	chlorobenzene	-3.86	-3.65
0076but	C_4H_8O	chlorobenzene	-4.47	-4.41
0078pen	$C_5H_{10}O$	chlorobenzene	-5.29	-5.14
0080hex	$C_6H_{12}O$	chlorobenzene	-5.84	-5.89
0081dim	$C_6H_{12}O$	chlorobenzene	-5.25	-5.37
0082hep	$C_7H_{14}O$	chlorobenzene	-6.46	-6.55
0087pro	$C_3H_6O_2$	chlorobenzene	-4.38	-6.07
0093met	$C_3H_6O_2$	chlorobenzene	-4.00	-4.49
0094met	$C_4H_8O_2$	chlorobenzene	-4.55	-4.99
0095eth	$C_4H_8O_2$	chlorobenzene	-4.63	-4.81
0097pro	$C_5H_{10}O_2$	chlorobenzene	-5.15	-5.46
0098met	$C_6H_{12}O_2$	chlorobenzene	-5.83	-6.41
0099but	$C_6H_{12}O_2$	chlorobenzene	-5.74	-6.22
0101pen	$C_7H_{14}O_2$	chlorobenzene	-6.49	-6.99
0103eth	C_2H_7N	chlorobenzene	-2.73	-2.89
0104dim	C_2H_7N	chlorobenzene	-2.75	-2.59
0106pro	C_3H_9N	chlorobenzene	-3.59	-3.75
0107tri	C_3H_9N	chlorobenzene	-2.82	-2.86
0118ani	C_6H_7N	chlorobenzene	-6.72	-6.86
0174chl	C_6H_5Cl	chlorobenzene	-5.66	-5.03
0215pbr	C_6H_5OBr	chlorobenzene	-8.54	-9.00
0216amm	H_3N	chlorobenzene	-1.22	-3.35
0228met	CH_5N	chlorobenzene	-2.16	-2.30
0506nit	CH_3NO_2	chlorobenzene	-4.32	-4.10
n011	C_7H_9N	chlorobenzene	-7.54	-7.41
0008noc	C_8H_{18}	chloroform	-5.25	-4.61
0018cyc	C_6H_{12}	chloroform	-4.45	-3.97
0035ben	C_6H_6	chloroform	-4.64	-4.20
0036tol	C_7H_8	chloroform	-5.48	-4.72
0037eth	C_8H_{10}	chloroform	-5.84	-5.43
0038oxy	C_8H_{10}	chloroform	-6.23	-5.27
0039mxy	C_8H_{10}	chloroform	-5.86	-5.23
0041nap	$C_{10}H_8$	chloroform	-7.89	-7.25
0044met	CH_4O	chloroform	-3.32	-2.82
0045eth	C_2H_6O	chloroform	-3.94	-3.24
0046eth	$C_2H_6O_2$	chloroform	-5.98	-6.24
0047pro	C_3H_8O	chloroform	-4.41	-3.89
0048pro	C_3H_8O	chloroform	-4.28	-3.51
0049but	$C_4H_{10}O$	chloroform	-5.28	-4.71
0050met	$C_4H_{10}O$	chloroform	-4.48	-3.57
0052pen	$C_5H_{12}O$	chloroform	-5.90	-5.53
0053phe	C_6H_6O	chloroform	-7.14	-6.94
0054hex	$C_6H_{14}O$	chloroform	-6.67	-6.36
0055ocr	C_7H_8O	chloroform	-7.55	-7.56
0056mcr	C_7H_8O	chloroform	-6.70	-7.44
0057pcr	C_7H_8O	chloroform	-7.59	-7.45
0058hep	$C_7H_{16}O$	chloroform	-7.53	-7.22

0062dio	$C_4H_8O_2$	chloroform	-6.21	-5.18
0063die	$C_4H_{10}O$	chloroform	-4.32	-3.74
0068ani	C_7H_8O	chloroform	-6.24	-6.71
0069met	CH_2O	chloroform	0.12	-2.42
0070eth	C_2H_4O	chloroform	-3.65	-3.13
0074ben	C_7H_6O	chloroform	-7.09	-7.06
0075pro	C_3H_6O	chloroform	-4.42	-3.59
0076but	C_4H_8O	chloroform	-5.43	-4.35
0084met	C_8H_8O	chloroform	-7.81	-7.84
0086eth	$C_2H_4O_2$	chloroform	-4.74	-4.96
0087pro	$C_3H_6O_2$	chloroform	-5.37	-5.73
0088but	$C_4H_8O_2$	chloroform	-5.99	-6.27
0089pen	$C_5H_{10}O_2$	chloroform	-6.61	-6.91
0090hex	$C_6H_{12}O_2$	chloroform	-7.51	-7.60
0093met	$C_3H_6O_2$	chloroform	-4.90	-4.49
0094met	$C_4H_8O_2$	chloroform	-5.48	-5.20
0095eth	$C_4H_8O_2$	chloroform	-5.58	-4.97
0097pro	$C_5H_{10}O_2$	chloroform	-6.35	-5.55
0098met	$C_6H_{12}O_2$	chloroform	-6.68	-6.58
0099but	$C_6H_{12}O_2$	chloroform	-6.71	-6.32
0100met	$C_7H_{14}O_2$	chloroform	-7.24	-7.38
0101pen	$C_7H_{14}O_2$	chloroform	-7.36	-7.12
0103eth	C_2H_7N	chloroform	-4.02	-2.71
0104dim	C_2H_7N	chloroform	-3.69	-2.80
0106pro	C_3H_9N	chloroform	-4.73	-3.50
0107tri	C_3H_9N	chloroform	-3.90	-3.19
0111die	$C_4H_{11}N$	chloroform	-5.23	-3.62
0116pyr	C_5H_5N	chloroform	-6.45	-5.32
0117met	$C_5H_6N_2$	chloroform	-6.99	-6.81
0118ani	C_6H_7N	chloroform	-7.34	-6.73
0119met	C_6H_7N	chloroform	-6.98	-5.88
0120met	C_6H_7N	chloroform	-7.35	-6.06
0121met	C_6H_7N	chloroform	-7.50	-6.01
0125dim	C_7H_9N	chloroform	-7.74	-6.01
0126eth	C_2H_3N	chloroform	-4.44	-3.43
0129ben	C_7H_5N	chloroform	-7.22	-6.83
0134nit	$C_6H_5NO_2$	chloroform	-7.78	-7.83
0135met	$C_7H_7NO_2$	chloroform	-8.30	-8.61
0139thi	C_6H_6S	chloroform	-7.61	-6.43
0142die	$C_4H_{10}S$	chloroform	-6.40	-5.26
0144thi	C_7H_8S	chloroform	-5.98	-6.99
0145pro	C_3H_6O	chloroform	-4.34	-4.40
0149mor	C_4H_9NO	chloroform	-6.72	-6.06
0151phy	$C_7H_6O_2$	chloroform	-10.30	-10.13
0157flu	C_6H_5F	chloroform	-4.25	-4.66
0162tri	$CHCl_3$	chloroform	-4.13	-3.83
0174chl	C_6H_5Cl	chloroform	-5.45	-5.55
0176pdi	$C_6H_4Cl_2$	chloroform	-6.32	-6.82
0186bro	C_6H_5Br	chloroform	-6.07	-6.03
0207tri	$C_2H_3OF_3$	chloroform	-3.03	-3.47
0215pbr	C_6H_5OBr	chloroform	-8.59	-8.93
0216amm	H_3N	chloroform	-2.41	-2.91
0217wat	H_2O	chloroform	-2.05	-4.49
0219hyd	H_2S	chloroform	-0.51	-1.99
0220tri	$C_3H_9O_4P$	chloroform	-9.74	-8.84
0221tri	$C_6H_{15}O_4P$	chloroform	-10.90	-9.08
0222tri	$C_9H_{21}O_4P$	chloroform	-11.11	-10.40
0225pipa	$C_5H_{11}N$	chloroform	-6.37	-4.78
0228met	CH_5N	chloroform	-3.17	-2.38
0229hyd	H_4N_2	chloroform	-4.42	-6.35
0230eth	$C_6H_8N_2$	chloroform	-7.72	-7.62
0233ethb	C_2H_5NO	chloroform	-7.05	-6.52
0240met	$C_8H_8O_2$	chloroform	-7.81	-8.83
0242dii	$C_6H_{14}O$	chloroform	-3.78	-4.36
0244tet	$C_5H_{10}O$	chloroform	-5.84	-4.60
0245thi	C_4H_4S	chloroform	-5.83	-5.27
0246eth	$C_8H_{10}O$	chloroform	-7.16	-7.38
0401amia	$C_9H_{12}N_2O$	chloroform	-13.64	-12.32
0402adn	$C_6H_7N_5$	chloroform	-12.51	-14.84
0403thi	$C_6H_8N_2O_2$	chloroform	-9.71	-12.83
0421dfl	CF_2Cl_2	chloroform	-1.55	-2.45
0422ftc	$CFCl_3$	chloroform	-2.62	-3.34
0441pho	$C_8H_{10}NO_5PS$	chloroform	-9.51	-10.78
0506nit	CH_3NO_2	chloroform	-4.68	-4.19
0519dim	C_4H_9NO	chloroform	-8.38	-6.10
0579pyy	C_4H_5N	chloroform	-5.50	-4.28
0582qui	C_9H_7N	chloroform	-10.23	-7.88
n007	CH_4N_2O	chloroform	-8.56	-11.00
n008	C_7H_7NO	chloroform	-11.06	-11.18
n009	C_7H_9N	chloroform	-8.23	-7.29
n011	C_7H_9N	chloroform	-8.01	-7.27
n017	H_2O_2	chloroform	-4.70	-4.60
n186	C_5H_9NO	chloroform	-9.82	-6.65

n191	$C_4H_4N_2O_2$	chloroform	-14.28	-13.42
n200	$C_4H_3N_2O_2F$	chloroform	-14.31	-13.63
n203	$C_4H_3N_2O_2Br$	chloroform	-15.03	-16.79
test4001	C_6H_5I	chloroform	-6.60	-7.26
0075pro	C_3H_6O	chlorohexane	-3.45	-3.67
0076but	C_4H_8O	chlorohexane	-4.10	-4.42
0078pen	$C_5H_{10}O$	chlorohexane	-4.84	-5.15
0080hex	$C_6H_{12}O$	chlorohexane	-5.42	-5.89
0081dim	$C_6H_{12}O$	chlorohexane	-4.98	-5.39
0093met	$C_3H_6O_2$	chlorohexane	-3.66	-4.50
0094met	$C_4H_8O_2$	chlorohexane	-4.20	-5.01
0095eth	$C_4H_8O_2$	chlorohexane	-4.25	-4.82
0097pro	$C_5H_{10}O_2$	chlorohexane	-4.84	-5.48
0098met	$C_6H_{12}O_2$	chlorohexane	-5.41	-6.43
0099but	$C_6H_{12}O_2$	chlorohexane	-5.37	-6.23
0003pro	C_3H_8	cyclohexane	-2.09	-1.67
0004nbu	C_4H_{10}	cyclohexane	-2.86	-2.47
0005npe	C_5H_{12}	cyclohexane	-3.50	-3.26
0008noc	C_8H_{18}	cyclohexane	-5.63	-5.52
0018cyc	C_6H_{12}	cyclohexane	-4.43	-3.82
0035ben	C_6H_6	cyclohexane	-4.19	-3.67
0036tol	C_7H_8	cyclohexane	-4.90	-4.36
0037eth	C_8H_{10}	cyclohexane	-4.97	-5.13
0038oxy	C_8H_{10}	cyclohexane	-5.54	-5.05
0039mxy	C_8H_{10}	cyclohexane	-5.52	-5.03
0041nap	$C_{10}H_8$	cyclohexane	-7.17	-6.64
0044met	CH_4O	cyclohexane	-1.29	-1.83
0045eth	C_2H_6O	cyclohexane	-2.42	-2.44
0047pro	C_3H_8O	cyclohexane	-2.73	-3.15
0048pro	C_3H_8O	cyclohexane	-2.37	-2.88
0049but	$C_4H_{10}O$	cyclohexane	-3.52	-3.85
0050met	$C_4H_{10}O$	cyclohexane	-2.93	-3.03
0052pen	$C_5H_{12}O$	cyclohexane	-3.61	-4.55
0053phe	C_6H_6O	cyclohexane	-5.57	-5.73
0054hex	$C_6H_{14}O$	cyclohexane	-5.31	-5.22
0055ocr	C_7H_8O	cyclohexane	-6.02	-6.43
0056mcr	C_7H_8O	cyclohexane	-5.20	-6.37
0057pcr	C_7H_8O	cyclohexane	-5.89	-6.38
0058hep	$C_7H_{16}O$	cyclohexane	-6.02	-5.90
0062dio	$C_4H_8O_2$	cyclohexane	-4.17	-4.03
0063die	$C_4H_{10}O$	cyclohexane	-3.03	-3.16
0068ani	C_7H_8O	cyclohexane	-5.38	-5.59
0074ben	C_7H_6O	cyclohexane	-5.71	-6.07
0075pro	C_3H_6O	cyclohexane	-2.67	-2.86
0076but	C_4H_8O	cyclohexane	-3.48	-3.75
0078pen	$C_5H_{10}O$	cyclohexane	-4.19	-4.49
0079pen	$C_5H_{10}O$	cyclohexane	-4.30	-4.41
0080hex	$C_6H_{12}O$	cyclohexane	-4.77	-5.14
0081dim	$C_6H_{12}O$	cyclohexane	-4.42	-4.70
0082hep	$C_7H_{14}O$	cyclohexane	-5.47	-5.81
0084met	C_8H_8O	cyclohexane	-6.29	-6.85
0086eth	$C_2H_4O_2$	cyclohexane	-1.73	-3.77
0087pro	$C_3H_6O_2$	cyclohexane	-3.78	-4.56
0093met	$C_3H_6O_2$	cyclohexane	-3.06	-3.55
0094met	$C_4H_8O_2$	cyclohexane	-3.71	-4.34
0095eth	$C_4H_8O_2$	cyclohexane	-3.56	-4.22
0097pro	$C_5H_{10}O_2$	cyclohexane	-4.36	-4.79
0098met	$C_6H_{12}O_2$	cyclohexane	-5.04	-5.55
0099but	$C_6H_{12}O_2$	cyclohexane	-4.94	-5.43
0100met	$C_7H_{14}O_2$	cyclohexane	-5.75	-6.20
0101pen	$C_7H_{14}O_2$	cyclohexane	-5.71	-6.08
0103eth	C_2H_7N	cyclohexane	-2.04	-2.37
0107tri	C_3H_9N	cyclohexane	-2.63	-2.62
0111die	$C_4H_{11}N$	cyclohexane	-3.61	-3.48
0116pyr	C_5H_5N	cyclohexane	-4.30	-4.29
0118ani	C_6H_7N	cyclohexane	-5.52	-5.53
0119met	C_6H_7N	cyclohexane	-5.05	-4.81
0120met	C_6H_7N	cyclohexane	-5.14	-4.96
0121met	C_6H_7N	cyclohexane	-5.23	-4.93
0122Nme	C_7H_9N	cyclohexane	-6.33	-5.82
0125dim	C_7H_9N	cyclohexane	-5.51	-5.12
0126eth	C_2H_3N	cyclohexane	-1.87	-2.45
0129ben	C_7H_5N	cyclohexane	-5.54	-5.65
0131nit	$C_3H_7NO_2$	cyclohexane	-4.06	-4.48
0134nit	$C_6H_5NO_2$	cyclohexane	-6.62	-6.53
0135met	$C_7H_7NO_2$	cyclohexane	-6.71	-7.16
0138pro	C_3H_8S	cyclohexane	-3.12	-3.72
0144thi	C_7H_8S	cyclohexane	-5.66	-6.69
0150mhy	$C_7H_6O_2$	cyclohexane	-6.88	-8.21
0151phy	$C_7H_6O_2$	cyclohexane	-7.19	-8.20
0157flu	C_6H_5F	cyclohexane	-3.59	-4.09
0165tri	$C_2H_3Cl_3$	cyclohexane	-4.08	-4.07
0173tri	C_2HCl_3	cyclohexane	-4.29	-3.90

0174chl	C_6H_5Cl	cyclohexane	-5.10	-4.88
0176pdi	$C_6H_4Cl_2$	cyclohexane	-5.89	-6.02
0186bro	C_6H_5Br	cyclohexane	-5.29	-5.50
0207tri	$C_2H_3OF_3$	cyclohexane	-1.53	-2.49
0215pbr	C_6H_5OBr	cyclohexane	-7.14	-7.70
0217wat	H_2O	cyclohexane	-0.39	-2.73
0220tri	$C_3H_9O_4P$	cyclohexane	-5.67	-6.35
0221tri	$C_6H_{15}O_4P$	cyclohexane	-7.60	-7.12
0222tri	$C_9H_{21}O_4P$	cyclohexane	-7.71	-8.25
0240met	$C_8H_8O_2$	cyclohexane	-7.01	-7.53
0244tet	$C_5H_{10}O$	cyclohexane	-4.41	-3.75
0246eth	$C_8H_{10}O$	cyclohexane	-6.00	-6.41
0421dfl	CF_2Cl_2	cyclohexane	-1.81	-1.64
0422ftc	$CFCl_3$	cyclohexane	-2.63	-2.98
0425dbr	$C_7H_3NOBr_2$	cyclohexane	-6.83	-9.26
0506nit	CH_3NO_2	cyclohexane	-2.86	-2.91
0515dim	C_3H_7NO	cyclohexane	-3.82	-4.02
0579pyy	C_4H_5N	cyclohexane	-3.77	-3.25
0582qui	C_9H_7N	cyclohexane	-7.38	-6.62
n008	C_7H_7NO	cyclohexane	-8.72	-8.12
n009	C_7H_9N	cyclohexane	-6.44	-6.18
n010	C_7H_9N	cyclohexane	-6.47	-6.21
n011	C_7H_9N	cyclohexane	-6.30	-6.22
test4001	C_6H_5I	cyclohexane	-6.26	-6.92
0036tol	C_7H_8	decalin	-4.37	-4.11
0053phe	C_6H_6O	decalin	-5.38	-5.80
0056mcr	C_7H_8O	decalin	-5.11	-6.13
0057pcr	C_7H_8O	decalin	-5.68	-6.14
0068ani	C_7H_8O	decalin	-5.00	-5.55
0084met	C_8H_8O	decalin	-6.23	-6.48
0086eth	$C_2H_4O_2$	decalin	-4.49	-4.10
0087pro	$C_3H_6O_2$	decalin	-4.42	-4.47
0093met	$C_3H_6O_2$	decalin	-2.90	-3.31
0094met	$C_4H_8O_2$	decalin	-3.50	-3.79
0095eth	$C_4H_8O_2$	decalin	-3.47	-3.67
0097pro	$C_5H_{10}O_2$	decalin	-4.05	-4.25
0098met	$C_6H_{12}O_2$	decalin	-4.83	-5.03
0099but	$C_6H_{12}O_2$	decalin	-4.71	-4.90
0100met	$C_7H_{14}O_2$	decalin	-5.51	-5.70
0101pen	$C_7H_{14}O_2$	decalin	-5.44	-5.56
0110but	$C_4H_{11}N$	decalin	-3.72	-3.55
0118ani	C_6H_7N	decalin	-5.78	-5.81
0122Nme	C_7H_9N	decalin	-6.41	-5.94
0129ben	C_7H_5N	decalin	-5.86	-5.28
0134nit	$C_6H_5NO_2$	decalin	-6.36	-6.17
0144thi	C_7H_8S	decalin	-5.54	-6.35
0157flu	C_6H_5F	decalin	-3.44	-3.95
0174chl	C_6H_5Cl	decalin	-4.61	-4.53
0186bro	C_6H_5Br	decalin	-5.25	-5.51
0240met	$C_8H_8O_2$	decalin	-6.76	-7.11
test4001	C_6H_5I	decalin	-5.96	-6.56
0008noc	C_8H_{18}	decane	-5.18	-4.79
0035ben	C_6H_6	decane	-3.80	-3.47
0036tol	C_7H_8	decane	-4.65	-4.11
0037eth	C_8H_{10}	decane	-5.25	-4.86
0044met	CH_4O	decane	-1.29	-1.77
0045eth	C_2H_6O	decane	-2.44	-2.32
0047pro	C_3H_8O	decane	-2.76	-2.83
0049but	$C_4H_{10}O$	decane	-3.77	-3.38
0052pen	$C_5H_{12}O$	decane	-3.92	-4.01
0053phe	C_6H_6O	decane	-5.50	-5.65
0054hex	$C_6H_{14}O$	decane	-4.97	-4.60
0057pcr	C_7H_8O	decane	-6.00	-5.97
0058hep	$C_7H_{16}O$	decane	-5.62	-5.23
0062dio	$C_4H_8O_2$	decane	-3.97	-3.84
0075pro	C_3H_6O	decane	-2.47	-2.65
0076but	C_4H_8O	decane	-3.30	-3.22
0078pen	$C_5H_{10}O$	decane	-3.93	-3.83
0080hex	$C_6H_{12}O$	decane	-4.61	-4.49
0081dim	$C_6H_{12}O$	decane	-4.15	-4.18
0082hep	$C_7H_{14}O$	decane	-5.18	-5.15
0093met	$C_3H_6O_2$	decane	-2.98	-3.20
0094met	$C_4H_8O_2$	decane	-3.49	-3.68
0095eth	$C_4H_8O_2$	decane	-3.43	-3.57
0097pro	$C_5H_{10}O_2$	decane	-4.02	-4.14
0098met	$C_6H_{12}O_2$	decane	-4.77	-4.89
0099but	$C_6H_{12}O_2$	decane	-4.66	-4.77
0100met	$C_7H_{14}O_2$	decane	-5.48	-5.54
0101pen	$C_7H_{14}O_2$	decane	-5.31	-5.41
0103eth	C_2H_7N	decane	-1.92	-2.08
0106pro	C_3H_9N	decane	-2.96	-2.68
0110but	$C_4H_{11}N$	decane	-3.55	-3.34
0157flu	C_6H_5F	decane	-3.48	-3.92

0173tri	C_2HCl_3	decane	-3.84	-3.71
0174chl	C_6H_5Cl	decane	-4.93	-4.52
0186bro	C_6H_5Br	decane	-5.43	-5.47
0233ethb	C_2H_5NO	decane	-2.85	-3.80
0506nit	CH_3NO_2	decane	-2.81	-2.67
0512dec	$C_{10}H_{22}$	decane	-6.53	-6.02
n011	C_7H_9N	decane	-6.05	-5.92
0053phe	C_6H_6O	decanol	-8.58	-7.10
0055ocr	C_7H_8O	decanol	-8.58	-7.38
0056mcr	C_7H_8O	decanol	-8.01	-7.41
0057pcr	C_7H_8O	decanol	-8.91	-7.43
0059dec	$C_{10}H_{22}O$	decanol	-9.58	-8.76
0103eth	C_2H_7N	decanol	-3.91	-3.36
0106pro	C_3H_9N	decanol	-4.59	-4.15
0110but	$C_4H_{11}N$	decanol	-5.22	-4.94
0146met	$C_3H_8O_2$	decanol	-5.41	-5.33
0174chl	C_6H_5Cl	decanol	-4.83	-4.88
0215pbr	C_6H_5OBr	decanol	-10.32	-8.79
0044met	CH_4O	dibromoethane	-2.38	-2.92
0045eth	C_2H_6O	dibromoethane	-2.69	-3.34
0047pro	C_3H_8O	dibromoethane	-3.82	-3.88
0049but	$C_4H_{10}O$	dibromoethane	-4.65	-4.67
0052pen	$C_5H_{12}O$	dibromoethane	-5.44	-5.49
0053phe	C_6H_6O	dibromoethane	-7.22	-6.84
0054hex	$C_6H_{14}O$	dibromoethane	-6.08	-6.33
0057pcr	C_7H_8O	dibromoethane	-7.52	-7.34
0058hep	$C_7H_{16}O$	dibromoethane	-6.64	-7.16
0215pbr	C_6H_5OBr	dibromoethane	-9.01	-8.82
0008noc	C_8H_{18}	dibutyl ether	-5.24	-4.78
0036tol	C_7H_8	dibutyl ether	-4.87	-4.21
0045eth	C_2H_6O	dibutyl ether	-3.51	-2.93
0062dio	$C_4H_8O_2$	dibutyl ether	-4.37	-4.49
0076but	C_4H_8O	dibutyl ether	-3.78	-3.79
0086eth	$C_2H_4O_2$	dibutyl ether	-5.21	-4.80
0087pro	$C_3H_6O_2$	dibutyl ether	-6.11	-5.20
0111die	$C_4H_{11}N$	dibutyl ether	-3.80	-3.49
0116pyr	C_5H_5N	dibutyl ether	-4.65	-4.75
0117met	$C_5H_6N_2$	dibutyl ether	-5.12	-5.51
0119met	C_6H_7N	dibutyl ether	-5.20	-5.05
0230eth	$C_6H_8N_2$	dibutyl ether	-5.87	-6.27
0501but	$C_8H_{18}O$	dibutyl ether	-5.76	-5.95
0506nit	CH_3NO_2	dibutyl ether	-3.67	-3.44
n017	H_2O_2	dibutyl ether	-5.75	-4.40
0008noc	C_8H_{18}	diethyl ether	-5.62	-4.84
0035ben	C_6H_6	diethyl ether	-4.21	-4.00
0036tol	C_7H_8	diethyl ether	-5.23	-4.62
0037eth	C_8H_{10}	diethyl ether	-5.45	-5.36
0038oxy	C_8H_{10}	diethyl ether	-5.58	-5.25
0039mxy	C_8H_{10}	diethyl ether	-5.56	-5.21
0041nap	$C_{10}H_8$	diethyl ether	-7.25	-7.10
0044met	CH_4O	diethyl ether	-3.61	-2.49
0045eth	C_2H_6O	diethyl ether	-4.41	-3.05
0046eth	$C_2H_6O_2$	diethyl ether	-6.20	-6.15
0047pro	C_3H_8O	diethyl ether	-4.90	-3.90
0048pro	C_3H_8O	diethyl ether	-4.44	-3.39
0049but	$C_4H_{10}O$	diethyl ether	-5.69	-4.66
0050met	$C_4H_{10}O$	diethyl ether	-4.80	-3.33
0051cyc	$C_5H_{10}O$	diethyl ether	-6.50	-5.42
0052pen	$C_5H_{12}O$	diethyl ether	-6.11	-5.51
0053phe	C_6H_6O	diethyl ether	-8.75	-6.83
0054hex	$C_6H_{14}O$	diethyl ether	-6.82	-6.34
0056mcr	C_7H_8O	diethyl ether	-7.95	-7.44
0058hep	$C_7H_{16}O$	diethyl ether	-7.51	-7.18
0062dio	$C_4H_8O_2$	diethyl ether	-4.67	-4.93
0063die	$C_4H_{10}O$	diethyl ether	-3.39	-3.64
0068ani	C_7H_8O	diethyl ether	-5.71	-6.45
0070eth	C_2H_4O	diethyl ether	-2.85	-2.91
0071proa	C_3H_6O	diethyl ether	-3.85	-3.90
0074ben	C_7H_6O	diethyl ether	-6.08	-7.00
0076but	C_4H_8O	diethyl ether	-4.09	-4.45
0084met	C_8H_8O	diethyl ether	-6.79	-7.78
0086eth	$C_2H_4O_2$	diethyl ether	-6.26	-4.92
0087pro	$C_3H_6O_2$	diethyl ether	-6.75	-5.75
0088but	$C_4H_8O_2$	diethyl ether	-7.32	-6.30
0089pen	$C_5H_{10}O_2$	diethyl ether	-7.87	-6.93
0090hex	$C_6H_{12}O_2$	diethyl ether	-8.85	-7.62
0103eth	C_2H_7N	diethyl ether	-2.89	-2.55
0104dim	C_2H_7N	diethyl ether	-2.63	-2.53
0106pro	C_3H_9N	diethyl ether	-3.65	-3.38
0107tri	C_3H_9N	diethyl ether	-2.78	-2.89
0110but	$C_4H_{11}N$	diethyl ether	-4.24	-4.29
0111die	$C_4H_{11}N$	diethyl ether	-3.83	-3.41
0115dip	$C_6H_{15}N$	diethyl ether	-4.96	-5.24

0116pyr	C_5H_5N	diethyl ether	-4.81	-5.08
0118ani	C_6H_7N	diethyl ether	-6.51	-6.61
0126eth	C_2H_3N	diethyl ether	-3.59	-3.21
0129ben	C_7H_5N	diethyl ether	-6.36	-6.73
0134nit	$C_6H_5NO_2$	diethyl ether	-6.85	-7.71
0135met	$C_7H_7NO_2$	diethyl ether	-7.21	-8.42
0145pro	C_3H_6O	diethyl ether	-4.87	-4.30
0146met	$C_3H_8O_2$	diethyl ether	-5.12	-5.16
0150mhy	$C_7H_6O_2$	diethyl ether	-11.36	-9.98
0151phy	$C_7H_6O_2$	diethyl ether	-12.07	-9.97
0174chl	C_6H_5Cl	diethyl ether	-5.42	-5.27
0176pdi	$C_6H_4Cl_2$	diethyl ether	-6.18	-6.50
0186bro	C_6H_5Br	diethyl ether	-5.99	-5.93
0216amm	H_3N	diethyl ether	-1.41	-2.77
0217wat	H_2O	diethyl ether	-3.85	-4.27
0219hyd	H_2S	diethyl ether	-0.60	-1.54
0225pipa	$C_5H_{11}N$	diethyl ether	-4.82	-4.52
0228met	CH_5N	diethyl ether	-2.32	-2.10
0229hyd	H_4N_2	diethyl ether	-3.08	-5.42
0233ethb	C_2H_5NO	diethyl ether	-6.16	-6.19
0236oct	$C_8H_{18}O$	diethyl ether	-7.25	-7.94
0506nit	CH_3NO_2	diethyl ether	-4.19	-4.01
0515dim	C_3H_7NO	diethyl ether	-5.31	-5.26
n007	CH_4N_2O	diethyl ether	-9.11	-10.00
n008	C_7H_7NO	diethyl ether	-10.60	-10.69
n017	H_2O_2	diethyl ether	-7.03	-4.63
n127	CH_3NO	diethyl ether	-5.97	-5.88
n191	$C_4H_4N_2O_2$	diethyl ether	-15.03	-12.91
n200	$C_4H_3N_2O_2F$	diethyl ether	-15.56	-13.22
n203	$C_4H_3N_2O_2Br$	diethyl ether	-17.01	-16.47
test4001	C_6H_5I	diethyl ether	-6.64	-7.26
test4003	CH_3I	diethyl ether	-3.51	-3.41
0008noc	C_8H_{18}	diisopropyl ether	-5.38	-4.96
0036tol	C_7H_8	diisopropyl ether	-4.91	-4.45
0041nap	$C_{10}H_8$	diisopropyl ether	-7.24	-7.03
0045eth	C_2H_6O	diisopropyl ether	-3.90	-3.11
0053phe	C_6H_6O	diisopropyl ether	-8.35	-6.61
0062dio	$C_4H_8O_2$	diisopropyl ether	-4.42	-4.77
0069met	CH_2O	diisopropyl ether	-1.04	-1.79
0076but	C_4H_8O	diisopropyl ether	-3.96	-4.21
0086eth	$C_2H_4O_2$	diisopropyl ether	-5.73	-4.93
0087pro	$C_3H_6O_2$	diisopropyl ether	-6.37	-5.54
0088but	$C_4H_8O_2$	diisopropyl ether	-6.85	-6.05
0089pen	$C_5H_{10}O_2$	diisopropyl ether	-7.59	-6.63
0090hex	$C_6H_{12}O_2$	diisopropyl ether	-8.23	-7.25
0107tri	C_3H_9N	diisopropyl ether	-2.74	-2.70
0111die	$C_4H_{11}N$	diisopropyl ether	-3.78	-3.51
0116pyr	C_5H_5N	diisopropyl ether	-4.88	-4.97
0118ani	C_6H_7N	diisopropyl ether	-6.67	-6.52
0151phy	$C_7H_6O_2$	diisopropyl ether	-11.63	-9.39
0217wat	H_2O	diisopropyl ether	-3.58	-4.23
0242dii	$C_6H_{14}O$	diisopropyl ether	-3.97	-4.13
0506nit	CH_3NO_2	diisopropyl ether	-3.90	-3.69
n017	H_2O_2	diisopropyl ether	-6.72	-4.54
0008noc	C_8H_{18}	dimethylpyridine	-4.88	-4.46
0036tol	C_7H_8	dimethylpyridine	-5.03	-4.29
0045eth	C_2H_6O	dimethylpyridine	-4.87	-3.53
0062dio	$C_4H_8O_2$	dimethylpyridine	-4.90	-5.13
0076but	C_4H_8O	dimethylpyridine	-4.34	-4.34
0125dim	C_7H_9N	dimethylpyridine	-6.04	-5.72
0045eth	C_2H_6O	dodecane	-2.06	-2.21
0047pro	C_3H_8O	dodecane	-2.74	-2.73
0049but	$C_4H_{10}O$	dodecane	-3.47	-3.30
0052pen	$C_5H_{12}O$	dodecane	-4.09	-3.94
0054hex	$C_6H_{14}O$	dodecane	-4.28	-4.54
0058hep	$C_7H_{16}O$	dodecane	-5.41	-5.15
0084met	C_8H_8O	dodecane	-6.11	-6.16
0513dod	$C_{12}H_{26}$	dodecane	-7.83	-7.05
0008noc	C_8H_{18}	ethoxybenzene	-4.75	-4.57
0036tol	C_7H_8	ethoxybenzene	-4.99	-4.23
0045eth	C_2H_6O	ethoxybenzene	-3.45	-3.17
0062dio	$C_4H_8O_2$	ethoxybenzene	-4.87	-4.75
0076but	C_4H_8O	ethoxybenzene	-4.28	-3.96
0246eth	$C_8H_{10}O$	ethoxybenzene	-6.75	-6.69
0506nit	CH_3NO_2	ethoxybenzene	-4.45	-3.76
0008noc	C_8H_{18}	ethylacetate	-4.72	-4.72
0036tol	C_7H_8	ethylacetate	-5.05	-4.51
0044met	CH_4O	ethylacetate	-3.37	-2.85
0045eth	C_2H_6O	ethylacetate	-4.24	-3.48
0046eth	$C_2H_6O_2$	ethylacetate	-6.82	-6.51
0047pro	C_3H_8O	ethylacetate	-4.90	-4.23
0049but	$C_4H_{10}O$	ethylacetate	-5.77	-4.93
0052pen	$C_5H_{12}O$	ethylacetate	-6.13	-5.75

0053phe	C_6H_6O	ethylacetate	-8.70	-7.05
0054hex	$C_6H_{14}O$	ethylacetate	-6.92	-6.50
0058hep	$C_7H_{16}O$	ethylacetate	-7.56	-7.21
0062dio	$C_4H_8O_2$	ethylacetate	-5.03	-5.10
0086eth	$C_2H_4O_2$	ethylacetate	-6.46	-5.44
0087pro	$C_3H_6O_2$	ethylacetate	-6.95	-6.13
0088but	$C_4H_8O_2$	ethylacetate	-7.34	-6.69
0095eth	$C_4H_8O_2$	ethylacetate	-4.46	-5.01
0217wat	H_2O	ethylacetate	-4.26	-4.81
0236oct	$C_8H_{18}O$	ethylacetate	-8.41	-7.89
0506nit	CH_3NO_2	ethylacetate	-5.06	-4.21
n011	C_7H_9N	ethylacetate	-7.63	-7.42
n017	H_2O_2	ethylacetate	-7.60	-5.20
n191	$C_4H_4N_2O_2$	ethylacetate	-15.43	-13.47
n200	$C_4H_3N_2O_2F$	ethylacetate	-16.10	-13.96
n203	$C_4H_3N_2O_2Br$	ethylacetate	-17.93	-17.30
0037eth	C_8H_{10}	ethylbenzene	-5.67	-4.97
0044met	CH_4O	ethylbenzene	-1.43	-2.03
0045eth	C_2H_6O	ethylbenzene	-2.49	-2.68
0047pro	C_3H_8O	ethylbenzene	-3.71	-3.28
0049but	$C_4H_{10}O$	ethylbenzene	-3.77	-3.87
0052pen	$C_5H_{12}O$	ethylbenzene	-4.72	-4.55
0053phe	C_6H_6O	ethylbenzene	-6.82	-6.05
0054hex	$C_6H_{14}O$	ethylbenzene	-5.68	-5.22
0055ocr	C_7H_8O	ethylbenzene	-7.25	-6.51
0058hep	$C_7H_{16}O$	ethylbenzene	-6.70	-5.94
0075pro	C_3H_6O	ethylbenzene	-3.41	-2.99
0076but	C_4H_8O	ethylbenzene	-4.12	-3.74
0078pen	$C_5H_{10}O$	ethylbenzene	-4.85	-4.39
0080hex	$C_6H_{12}O$	ethylbenzene	-5.49	-5.08
0081dim	$C_6H_{12}O$	ethylbenzene	-4.92	-4.65
0082hep	$C_7H_{14}O$	ethylbenzene	-6.10	-5.79
0093met	$C_3H_6O_2$	ethylbenzene	-3.74	-3.74
0094met	$C_4H_8O_2$	ethylbenzene	-4.29	-4.24
0095eth	$C_4H_8O_2$	ethylbenzene	-4.31	-4.10
0097pro	$C_5H_{10}O_2$	ethylbenzene	-4.95	-4.70
0098met	$C_6H_{12}O_2$	ethylbenzene	-5.56	-5.51
0099but	$C_6H_{12}O_2$	ethylbenzene	-5.48	-5.37
0101pen	$C_7H_{14}O_2$	ethylbenzene	-6.20	-6.05
0103eth	C_2H_7N	ethylbenzene	-2.59	-2.39
0106pro	C_3H_9N	ethylbenzene	-3.44	-3.19
0107tri	C_3H_9N	ethylbenzene	-2.64	-2.60
0110but	$C_4H_{11}N$	ethylbenzene	-4.43	-3.93
0215pbr	C_6H_5OBr	ethylbenzene	-8.54	-8.05
0217wat	H_2O	ethylbenzene	-1.51	-3.48
0008noc	C_8H_{18}	fluorobenzene	-4.99	-4.72
0036tol	C_7H_8	fluorobenzene	-5.27	-4.45
0045eth	C_2H_6O	fluorobenzene	-3.45	-3.49
0062dio	$C_4H_8O_2$	fluorobenzene	-5.18	-5.10
0076but	C_4H_8O	fluorobenzene	-4.60	-4.44
0157flu	C_6H_5F	fluorobenzene	-4.60	-4.35
0506nit	CH_3NO_2	fluorobenzene	-4.62	-4.11
0093met	$C_3H_6O_2$	fluoroctane	-3.59	-4.00
0094met	$C_4H_8O_2$	fluoroctane	-4.09	-4.50
0095eth	$C_4H_8O_2$	fluoroctane	-4.16	-4.33
0097pro	$C_5H_{10}O_2$	fluoroctane	-4.65	-4.97
0098met	$C_6H_{12}O_2$	fluoroctane	-5.33	-5.87
0099but	$C_6H_{12}O_2$	fluoroctane	-5.22	-5.69
0007nhe	C_7H_{16}	heptane	-4.65	-4.69
0035ben	C_6H_6	heptane	-4.00	-3.57
0036tol	C_7H_8	heptane	-4.78	-4.26
0038oxy	C_8H_{10}	heptane	-5.52	-4.96
0039mxy	C_8H_{10}	heptane	-5.67	-4.94
0040pxy	C_8H_{10}	heptane	-5.52	-4.94
0041nap	$C_{10}H_8$	heptane	-7.02	-6.60
0042ant	$C_{14}H_{10}$	heptane	-10.00	-9.60
0044met	CH_4O	heptane	-1.29	-1.72
0045eth	C_2H_6O	heptane	-2.15	-2.32
0047pro	C_3H_8O	heptane	-3.01	-2.99
0049but	$C_4H_{10}O$	heptane	-3.66	-3.62
0052pen	$C_5H_{12}O$	heptane	-4.09	-4.31
0053phe	C_6H_6O	heptane	-5.32	-5.62
0054hex	$C_6H_{14}O$	heptane	-4.89	-4.96
0055ocr	C_7H_8O	heptane	-6.01	-6.23
0056mcr	C_7H_8O	heptane	-5.01	-6.25
0057pcr	C_7H_8O	heptane	-5.77	-6.26
0058hep	$C_7H_{16}O$	heptane	-5.60	-5.63
0068ani	C_7H_8O	heptane	-5.35	-5.46
0074ben	C_7H_6O	heptane	-5.50	-5.90
0075pro	C_3H_6O	heptane	-2.61	-2.74
0076but	C_4H_8O	heptane	-3.36	-3.62
0078pen	$C_5H_{10}O$	heptane	-4.07	-4.26
0080hex	$C_6H_{12}O$	heptane	-4.55	-4.91

0081dim	$C_6H_{12}O$	heptane	-4.30	-4.54
0082hep	$C_7H_{14}O$	heptane	-5.22	-5.56
0084met	C_8H_8O	heptane	-6.14	-6.69
0087pro	$C_3H_6O_2$	heptane	-4.06	-4.38
0088but	$C_4H_8O_2$	heptane	-5.05	-4.84
0089pen	$C_5H_{10}O_2$	heptane	-5.23	-5.34
0090hex	$C_6H_{12}O_2$	heptane	-6.54	-5.87
0093met	$C_3H_6O_2$	heptane	-2.97	-3.41
0094met	$C_4H_8O_2$	heptane	-3.63	-4.11
0095eth	$C_4H_8O_2$	heptane	-3.50	-4.00
0097pro	$C_5H_{10}O_2$	heptane	-4.09	-4.57
0098met	$C_6H_{12}O_2$	heptane	-4.92	-5.31
0099but	$C_6H_{12}O_2$	heptane	-4.83	-5.19
0100met	$C_7H_{14}O_2$	heptane	-5.63	-5.95
0101pen	$C_7H_{14}O_2$	heptane	-5.42	-5.83
0103eth	C_2H_7N	heptane	-2.09	-2.34
0106pro	C_3H_9N	heptane	-3.03	-3.03
0110but	$C_4H_{11}N$	heptane	-3.55	-3.57
0116pyr	C_5H_5N	heptane	-4.28	-4.24
0118ani	C_6H_7N	heptane	-5.38	-5.46
0126eth	C_2H_3N	heptane	-2.06	-2.22
0129ben	C_7H_5N	heptane	-5.33	-5.44
0134nit	$C_6H_5NO_2$	heptane	-6.14	-6.29
0157flu	C_6H_5F	heptane	-4.13	-3.98
0174chl	C_6H_5Cl	heptane	-5.15	-4.77
0175odi	$C_6H_4Cl_2$	heptane	-6.01	-5.87
0176pdi	$C_6H_4Cl_2$	heptane	-5.81	-5.91
0186bro	C_6H_5Br	heptane	-5.72	-5.47
0187dib	$C_6H_4Br_2$	heptane	-7.55	-7.35
0220tri	$C_3H_9O_4P$	heptane	-5.59	-5.89
0221tri	$C_6H_{15}O_4P$	heptane	-6.67	-6.78
0222tri	$C_9H_{21}O_4P$	heptane	-7.50	-7.89
0239oct	$C_8H_{16}O$	heptane	-5.68	-6.23
0245thi	C_4H_4S	heptane	-4.09	-4.66
0414dcl	$C_{12}H_8Cl_2$	heptane	-9.22	-9.95
0519dim	C_4H_9NO	heptane	-4.80	-4.54
n008	C_7H_7NO	heptane	-7.26	-7.79
n009	C_7H_9N	heptane	-6.28	-5.96
n010	C_7H_9N	heptane	-6.35	-5.99
n011	C_7H_9N	heptane	-6.15	-6.00
n186	C_5H_9NO	heptane	-5.80	-5.14
n200	$C_4H_3N_2O_2F$	heptane	-11.28	-9.05
n203	$C_4H_3N_2O_2Br$	heptane	-12.73	-12.10
test4001	C_6H_5I	heptane	-6.27	-6.82
0001met	CH_4	hexadecane	0.45	-1.31
0002eth	C_2H_6	hexadecane	-0.67	-1.31
0003pro	C_3H_8	hexadecane	-1.43	-1.31
0004nbu	C_4H_{10}	hexadecane	-2.20	-1.86
0005npe	C_5H_{12}	hexadecane	-2.95	-2.41
0006nhe	C_6H_{14}	hexadecane	-3.64	-2.95
0007nhe	C_7H_{16}	hexadecane	-4.33	-3.52
0008noc	C_8H_{18}	hexadecane	-5.02	-4.14
0009nhe	$C_{16}H_{34}$	hexadecane	-10.52	-9.06
0010met	C_4H_{10}	hexadecane	-1.92	-1.74
0011dim	C_5H_{12}	hexadecane	-2.48	-2.11
0012met	C_6H_{14}	hexadecane	-3.48	-2.87
0013dim	C_7H_{16}	hexadecane	-3.87	-3.37
0014tri	C_8H_{18}	hexadecane	-4.24	-4.21
0016cyc	C_3H_6	hexadecane	-1.78	-1.89
0017cyc	C_5H_{10}	hexadecane	-3.38	-3.03
0018cyc	C_6H_{12}	hexadecane	-4.04	-3.56
0019met	C_7H_{14}	hexadecane	-4.43	-3.98
0021eth	C_2H_4	hexadecane	-0.39	-1.31
0022pro	C_3H_6	hexadecane	-1.29	-1.31
0023str	C_4H_6	hexadecane	-2.10	-1.94
0025buta	C_4H_8	hexadecane	-2.03	-1.93
0027pen	C_5H_{10}	hexadecane	-2.79	-2.70
0029hex	C_6H_{12}	hexadecane	-3.51	-3.47
0030eth	C_2H_2	hexadecane	-0.20	-1.31
0031pro	C_3H_4	hexadecane	-1.40	-1.34
0032but	C_4H_6	hexadecane	-2.07	-2.11
0033pen	C_5H_8	hexadecane	-2.74	-2.88
0034hex	C_6H_{10}	hexadecane	-3.42	-3.70
0035ben	C_6H_6	hexadecane	-3.80	-3.99
0036tol	C_7H_8	hexadecane	-4.54	-4.56
0037eth	C_8H_{10}	hexadecane	-5.15	-5.10
0038oxy	C_8H_{10}	hexadecane	-5.37	-4.95
0039mxy	C_8H_{10}	hexadecane	-5.24	-4.92
0040pxy	C_8H_{10}	hexadecane	-5.24	-4.91
0041nap	$C_{10}H_8$	hexadecane	-7.29	-6.77
0042ant	$C_{14}H_{10}$	hexadecane	-10.32	-9.36
0043chr	$C_{18}H_{12}$	hexadecane	-14.10	-11.99
0044met	CH_4O	hexadecane	-1.32	-2.02

0045eth	C_2H_6O	hexadecane	-2.03	-2.29
0046eth	$C_2H_6O_2$	hexadecane	-2.81	-4.04
0047pro	C_3H_8O	hexadecane	-2.77	-2.81
0048pro	C_3H_8O	hexadecane	-2.47	-2.53
0049but	$C_4H_{10}O$	hexadecane	-3.55	-3.38
0050met	$C_4H_{10}O$	hexadecane	-2.74	-2.37
0051cyc	$C_5H_{10}O$	hexadecane	-4.42	-4.42
0052pen	$C_5H_{12}O$	hexadecane	-4.24	-4.03
0053phe	C_6H_6O	hexadecane	-5.14	-5.51
0054hex	$C_6H_{14}O$	hexadecane	-4.92	-4.63
0055ocr	C_7H_8O	hexadecane	-5.78	-5.83
0056mcr	C_7H_8O	hexadecane	-5.91	-5.85
0057pcr	C_7H_8O	hexadecane	-5.88	-5.86
0058hep	$C_7H_{16}O$	hexadecane	-5.62	-5.24
0059dec	$C_{10}H_{22}O$	hexadecane	-7.68	-6.83
0060dim	C_2H_6O	hexadecane	-1.49	-1.60
0061tet	C_4H_8O	hexadecane	-3.60	-3.17
0062dio	$C_4H_8O_2$	hexadecane	-3.82	-3.81
0063die	$C_4H_{10}O$	hexadecane	-2.81	-2.32
0066dim	$C_4H_{10}O_2$	hexadecane	-3.63	-3.29
0068ani	C_7H_8O	hexadecane	-5.35	-5.27
0069met	CH_2O	hexadecane	-0.99	-1.63
0070eth	C_2H_4O	hexadecane	-1.68	-2.27
0071proa	C_3H_6O	hexadecane	-2.48	-2.79
0072but	C_4H_8O	hexadecane	-3.10	-3.42
0073pen	$C_5H_{10}O$	hexadecane	-3.89	-3.94
0074ben	C_7H_6O	hexadecane	-5.44	-5.82
0075pro	C_3H_6O	hexadecane	-2.31	-2.46
0076but	C_4H_8O	hexadecane	-3.12	-2.96
0077cyc	C_5H_8O	hexadecane	-4.39	-4.16
0078pen	$C_5H_{10}O$	hexadecane	-3.76	-3.51
0079pen	$C_5H_{10}O$	hexadecane	-3.83	-3.43
0080hex	$C_6H_{12}O$	hexadecane	-4.45	-4.09
0081dim	$C_6H_{12}O$	hexadecane	-3.94	-3.79
0082hep	$C_7H_{14}O$	hexadecane	-5.13	-4.62
0083hep	$C_7H_{14}O$	hexadecane	-5.20	-4.51
0084met	C_8H_8O	hexadecane	-6.14	-6.10
0085non	$C_9H_{18}O$	hexadecane	-6.46	-5.58
0086eth	$C_2H_4O_2$	hexadecane	-2.39	-3.55
0087pro	$C_3H_6O_2$	hexadecane	-3.12	-3.97
0088but	$C_4H_8O_2$	hexadecane	-3.86	-4.46
0089pen	$C_5H_{10}O_2$	hexadecane	-4.61	-5.00
0090hex	$C_6H_{12}O_2$	hexadecane	-5.35	-5.56
0091met	$C_2H_4O_2$	hexadecane	-1.99	-2.95
0092ethb	$C_3H_6O_2$	hexadecane	-2.59	-3.29
0093met	$C_3H_6O_2$	hexadecane	-2.67	-2.98
0094met	$C_4H_8O_2$	hexadecane	-2.68	-3.39
0095eth	$C_4H_8O_2$	hexadecane	-3.25	-3.26
0096met	$C_5H_{10}O_2$	hexadecane	-4.01	-3.91
0097pro	$C_5H_{10}O_2$	hexadecane	-3.93	-3.77
0098met	$C_6H_{12}O_2$	hexadecane	-4.69	-4.47
0099but	$C_6H_{12}O_2$	hexadecane	-4.61	-4.33
0100met	$C_7H_{14}O_2$	hexadecane	-5.43	-5.04
0101pen	$C_7H_{14}O_2$	hexadecane	-5.20	-4.90
0102eth	$C_{20}H_{40}O_2$	hexadecane	-13.69	-13.04
0103eth	C_2H_7N	hexadecane	-2.29	-2.10
0104dim	C_2H_7N	hexadecane	-2.18	-1.89
0106pro	C_3H_9N	hexadecane	-2.92	-2.67
0107tri	C_3H_9N	hexadecane	-2.21	-2.41
0111die	$C_4H_{11}N$	hexadecane	-3.27	-2.73
0113pen	$C_5H_{13}N$	hexadecane	-4.28	-3.85
0115dip	$C_6H_{15}N$	hexadecane	-4.57	-4.09
0116pyr	C_5H_5N	hexadecane	-4.10	-3.95
0118ani	C_6H_7N	hexadecane	-5.44	-5.49
0119met	C_6H_7N	hexadecane	-4.68	-4.20
0120met	C_6H_7N	hexadecane	-4.91	-4.36
0121met	C_6H_7N	hexadecane	-4.89	-4.33
0122Nme	C_7H_9N	hexadecane	-6.19	-5.58
0123dim	C_7H_9N	hexadecane	-5.52	-4.55
0124dim	C_7H_9N	hexadecane	-5.52	-4.58
0125dim	C_7H_9N	hexadecane	-5.27	-4.40
0126eth	C_2H_3N	hexadecane	-2.37	-1.33
0127pro	C_3H_5N	hexadecane	-2.84	-2.05
0128butb	C_4H_7N	hexadecane	-3.48	-2.74
0129ben	C_7H_5N	hexadecane	-5.51	-4.63
0130nit	$C_2H_5NO_2$	hexadecane	-3.29	-2.85
0131nit	$C_3H_7NO_2$	hexadecane	-3.95	-3.41
0132nit	$C_3H_7NO_2$	hexadecane	-3.47	-3.27
0133nit	$C_4H_9NO_2$	hexadecane	-4.66	-4.02
0134nit	$C_6H_5NO_2$	hexadecane	-6.22	-5.49
0135met	$C_7H_7NO_2$	hexadecane	-6.52	-6.10
0137ethb	C_2H_6S	hexadecane	-2.96	-2.37
0138pro	C_3H_8S	hexadecane	-3.66	-2.93

0139thi	C_6H_6S	hexadecane	-5.61	-5.72
0140dim	C_2H_6S	hexadecane	-3.05	-2.50
0141dim	$C_2H_6S_2$	hexadecane	-4.84	-4.22
0142die	$C_4H_{10}S$	hexadecane	-4.23	-3.51
0143dip	$C_6H_{14}S$	hexadecane	-5.61	-4.49
0145pro	C_3H_6O	hexadecane	-2.73	-3.52
0155flu	C_2H_5F	hexadecane	-0.76	-1.31
0157flu	C_6H_5F	hexadecane	-4.03	-4.29
0158flu	$C_6H_{13}F$	hexadecane	-4.03	-3.51
0159flu	$C_8H_{17}F$	hexadecane	-5.25	-4.74
0161dic	CH_2Cl_2	hexadecane	-2.76	-2.15
0162tri	$CHCl_3$	hexadecane	-3.38	-2.89
0163chl	C_2H_5Cl	hexadecane	-2.29	-1.85
0165tri	$C_2H_3Cl_3$	hexadecane	-3.73	-3.72
0166tri	$C_2H_3Cl_3$	hexadecane	-4.49	-3.79
0167chla	C_3H_7Cl	hexadecane	-2.86	-2.34
0168chl	C_3H_7Cl	hexadecane	-2.69	-2.17
0170chl	C_3H_5Cl	hexadecane	-2.88	-2.50
0171Zdi	$C_2H_2Cl_2$	hexadecane	-3.33	-2.72
0172Edi	$C_2H_2Cl_2$	hexadecane	-3.11	-2.76
0173tri	C_2HCl_3	hexadecane	-4.08	-3.51
0174chl	C_6H_5Cl	hexadecane	-4.99	-4.90
0175odi	$C_6H_4Cl_2$	hexadecane	-6.16	-5.57
0176pdi	$C_6H_4Cl_2$	hexadecane	-6.02	-5.58
0178dib	CH_2Br_2	hexadecane	-3.94	-3.79
0179tri	$CHBr_3$	hexadecane	-5.16	-5.51
0180bro	C_2H_5Br	hexadecane	-2.89	-2.94
0182bro	C_3H_7Br	hexadecane	-3.57	-3.62
0183bro	C_3H_7Br	hexadecane	-3.26	-3.54
0184bro	C_4H_9Br	hexadecane	-4.24	-4.20
0185bro	$C_5H_{11}Br$	hexadecane	-4.93	-4.77
0186bro	C_6H_5Br	hexadecane	-5.51	-5.82
0201bro	$C_2H_1F_3ClBr$	hexadecane	-2.97	-2.71
0203bro	$C_2H_1F_4Br$	hexadecane	-1.87	-1.94
0204tet	C_2Cl_4	hexadecane	-4.88	-4.17
0206tri	$C_2F_3Cl_3$	hexadecane	-2.89	-3.04
0207tri	$C_2H_3OF_3$	hexadecane	-1.67	-2.46
0210dic	$C_3H_4OF_2Cl_2$	hexadecane	-3.90	-3.91
0214tri	$C_4H_5OF_3$	hexadecane	-1.91	-3.05
0216amm	H_3N	hexadecane	-0.93	-2.18
0217wat	H_2O	hexadecane	-0.35	-3.17
0219hyd	H_2S	hexadecane	-0.72	-1.37
0223die	$C_4H_{10}S_2$	hexadecane	-5.74	-5.17
0233ethb	C_2H_5NO	hexadecane	-3.33	-3.92
0236oct	$C_8H_{18}O$	hexadecane	-6.30	-5.79
0237oct	$C_8H_{16}O$	hexadecane	-5.98	-5.55
0239oct	$C_8H_{16}O$	hexadecane	-5.81	-5.15
0240met	$C_8H_8O_2$	hexadecane	-6.31	-6.73
0242dii	$C_6H_{14}O$	hexadecane	-4.02	-3.01
0244tet	$C_5H_{10}O$	hexadecane	-4.08	-3.65
0245thi	C_4H_4S	hexadecane	-4.01	-4.65
0246eth	$C_8H_{10}O$	hexadecane	-5.64	-5.63
0400hyd	H_2	hexadecane	1.64	-1.34
0417brp	C_3H_5Br	hexadecane	-3.42	-3.35
0418bri	C_4H_9Br	hexadecane	-4.04	-4.12
0419brt	C_7H_7Br	hexadecane	-6.36	-6.54
0420pbr	C_7H_7Br	hexadecane	-6.19	-6.29
0423brt	C_1Cl_3Br	hexadecane	-4.46	-4.41
0431pho	$C_3H_9O_3P$	hexadecane	-5.43	-5.01
0471dim	C_7H_9N	hexadecane	-2.58	-4.75
0506nit	CH_3NO_2	hexadecane	-2.58	-2.30
0571dim	C_7H_9N	hexadecane	-2.52	-4.75
0574eth	C_7H_9N	hexadecane	-2.45	-4.87
0939tet	$C_4H_{12}Si$	hexadecane	-2.92	-2.95
n007	CH_4N_2O	hexadecane	-6.37	-6.19
n009	C_7H_9N	hexadecane	-6.08	-5.81
n011	C_7H_9N	hexadecane	-6.04	-5.83
n127	CH_3NO	hexadecane	-2.91	-3.86
test4001	C_6H_5I	hexadecane	-6.25	-6.90
test4002	CH_2I_2	hexadecane	-5.26	-6.27
test4003	CH_3I	hexadecane	-2.88	-3.03
test4004	C_2H_5I	hexadecane	-3.51	-3.70
test4005	C_3H_5I	hexadecane	-4.10	-4.50
test4006	C_3H_7I	hexadecane	-4.27	-4.45
test4007	C_4H_9I	hexadecane	-4.95	-5.02
test4008	$C_5H_{11}I$	hexadecane	-5.63	-5.60
0005npe	C_5H_{12}	hexadecyliodide	-2.59	-2.32
0006nhe	C_6H_{14}	hexadecyliodide	-3.26	-2.92
0007nhe	C_7H_{16}	hexadecyliodide	-3.90	-3.53
0018cyc	C_6H_{12}	hexadecyliodide	-3.66	-3.60
0019met	C_7H_{14}	hexadecyliodide	-4.07	-4.04
0035ben	C_6H_6	hexadecyliodide	-3.71	-4.12
0036tol	C_7H_8	hexadecyliodide	-4.41	-4.72

0161dic	CH_2Cl_2	hexadecyliodide	-2.76	-2.24
0162tri	$CHCl_3$	hexadecyliodide	-3.36	-2.94
0006nhe	C_6H_{14}	hexane	-4.00	-4.09
0008noc	C_8H_{18}	hexane	-5.46	-5.56
0035ben	C_6H_6	hexane	-3.96	-3.66
0036tol	C_7H_8	hexane	-4.84	-4.36
0037eth	C_8H_{10}	hexane	-4.99	-5.14
0038oxy	C_8H_{10}	hexane	-5.22	-5.06
0039mxy	C_8H_{10}	hexane	-4.99	-5.04
0040pxy	C_8H_{10}	hexane	-5.01	-5.04
0044met	CH_4O	hexane	-1.49	-1.71
0045eth	C_2H_6O	hexane	-2.73	-2.34
0047pro	C_3H_8O	hexane	-2.81	-3.07
0049but	$C_4H_{10}O$	hexane	-3.77	-3.76
0052pen	$C_5H_{12}O$	hexane	-4.38	-4.44
0053phe	C_6H_6O	hexane	-5.49	-5.57
0054hex	$C_6H_{14}O$	hexane	-5.14	-5.10
0055ocr	C_7H_8O	hexane	-6.25	-6.27
0057pcr	C_7H_8O	hexane	-5.86	-6.25
0058hep	$C_7H_{16}O$	hexane	-5.75	-5.76
0062dio	$C_4H_8O_2$	hexane	-4.08	-3.92
0074ben	C_7H_6O	hexane	-5.53	-5.98
0075pro	C_3H_6O	hexane	-2.60	-2.77
0076but	C_4H_8O	hexane	-3.48	-3.65
0080hex	$C_6H_{12}O$	hexane	-4.68	-5.05
0081dim	$C_6H_{12}O$	hexane	-4.34	-4.61
0082hep	$C_7H_{14}O$	hexane	-5.36	-5.70
0084met	C_8H_8O	hexane	-6.05	-6.77
0086eth	$C_2H_4O_2$	hexane	-2.83	-3.59
0087pro	$C_3H_6O_2$	hexane	-2.98	-4.37
0093met	$C_3H_6O_2$	hexane	-3.12	-3.43
0094met	$C_4H_8O_2$	hexane	-3.65	-4.25
0095eth	$C_4H_8O_2$	hexane	-3.62	-4.14
0097pro	$C_5H_{10}O_2$	hexane	-4.10	-4.71
0098met	$C_6H_{12}O_2$	hexane	-4.94	-5.45
0099but	$C_6H_{12}O_2$	hexane	-4.86	-5.33
0100met	$C_7H_{14}O_2$	hexane	-5.64	-6.08
0101pen	$C_7H_{14}O_2$	hexane	-5.52	-5.96
0103eth	C_2H_7N	hexane	-2.09	-2.34
0106pro	C_3H_9N	hexane	-3.13	-3.10
0110but	$C_4H_{11}N$	hexane	-3.62	-3.70
0116pyr	C_5H_5N	hexane	-3.81	-4.19
0118ani	C_6H_7N	hexane	-5.43	-5.40
0130nit	$C_2H_5NO_2$	hexane	-3.19	-3.69
0133nit	$C_4H_9NO_2$	hexane	-4.64	-4.99
0134nit	$C_6H_5NO_2$	hexane	-6.09	-6.41
0151phy	$C_7H_6O_2$	hexane	-9.18	-8.00
0157flu	C_6H_5F	hexane	-4.15	-4.07
0162tri	$CHCl_3$	hexane	-3.17	-3.32
0174chl	C_6H_5Cl	hexane	-5.14	-4.87
0176pdi	$C_6H_4Cl_2$	hexane	-5.69	-6.01
0179tri	$CHBr_3$	hexane	-4.38	-5.52
0186bro	C_6H_5Br	hexane	-5.66	-5.47
0215pbr	C_6H_5OBr	hexane	-6.96	-7.57
0220tri	$C_3H_9O_4P$	hexane	-5.82	-6.08
0221tri	$C_6H_{15}O_4P$	hexane	-6.78	-6.93
0222tri	$C_9H_{21}O_4P$	hexane	-7.24	-8.18
0425dbr	$C_7H_3NOBr_2$	hexane	-9.67	-9.06
0506nit	CH_3NO_2	hexane	-2.90	-2.78
n008	C_7H_7NO	hexane	-7.77	-7.81
n011	C_7H_9N	hexane	-6.18	-6.03
0008noc	C_8H_{18}	iodobenzene	-4.72	-4.58
0036tol	C_7H_8	iodobenzene	-4.99	-4.23
0044met	CH_4O	iodobenzene	-2.18	-2.75
0045eth	C_2H_6O	iodobenzene	-3.18	-3.27
0047pro	C_3H_8O	iodobenzene	-3.52	-3.91
0049but	$C_4H_{10}O$	iodobenzene	-4.05	-4.59
0052pen	$C_5H_{12}O$	iodobenzene	-5.02	-5.37
0053phe	C_6H_6O	iodobenzene	-6.76	-6.67
0054hex	$C_6H_{14}O$	iodobenzene	-5.71	-6.10
0055ocr	C_7H_8O	iodobenzene	-7.14	-7.19
0056mcr	C_7H_8O	iodobenzene	-6.04	-7.22
0057pcr	C_7H_8O	iodobenzene	-7.01	-7.24
0058hep	$C_7H_{16}O$	iodobenzene	-6.53	-6.72
0062dio	$C_4H_8O_2$	iodobenzene	-4.94	-4.90
0076but	C_4H_8O	iodobenzene	-4.22	-4.26
0103eth	C_2H_7N	iodobenzene	-2.73	-2.72
0106pro	C_3H_9N	iodobenzene	-3.54	-3.58
0110but	$C_4H_{11}N$	iodobenzene	-4.19	-4.44
0215pbr	C_6H_5OBr	iodobenzene	-8.45	-8.71
0506nit	CH_3NO_2	iodobenzene	-4.10	-3.87
0005npe	C_5H_{12}	isooctane	-3.21	-3.05
0006nhe	C_6H_{14}	isooctane	-3.08	-3.80

0008noc	C_8H_{18}	isooctane	-5.44	-5.27
0022pro	C_3H_6	isooctane	-1.61	-1.42
0025buta	C_4H_8	isooctane	-2.26	-2.17
0027pen	C_5H_{10}	isooctane	-2.36	-2.92
0035ben	C_6H_6	isooctane	-4.01	-3.53
0036tol	C_7H_8	isooctane	-4.68	-4.22
0039mxy	C_8H_{10}	isooctane	-5.12	-4.90
0045eth	C_2H_6O	isooctane	-2.44	-2.33
0047pro	C_3H_8O	isooctane	-3.00	-2.96
0049but	$C_4H_{10}O$	isooctane	-3.56	-3.51
0052pen	$C_5H_{12}O$	isooctane	-4.17	-4.19
0053phe	C_6H_6O	isooctane	-5.30	-5.69
0054hex	$C_6H_{14}O$	isooctane	-5.10	-4.85
0055ocr	C_7H_8O	isooctane	-5.68	-6.18
0057pcr	C_7H_8O	isooctane	-5.59	-6.21
0062dio	$C_4H_8O_2$	isooctane	-4.02	-3.98
0072but	C_4H_8O	isooctane	-3.45	-3.83
0073pen	$C_5H_{10}O$	isooctane	-4.24	-4.57
0075pro	C_3H_6O	isooctane	-2.44	-2.74
0076but	C_4H_8O	isooctane	-3.40	-3.53
0078pen	$C_5H_{10}O$	isooctane	-4.14	-4.14
0080hex	$C_6H_{12}O$	isooctane	-4.72	-4.79
0110but	$C_4H_{11}N$	isooctane	-3.57	-3.46
0118ani	C_6H_7N	isooctane	-5.20	-5.56
0131nit	$C_3H_7NO_2$	isooctane	-3.94	-4.10
0137ethb	C_2H_6S	isooctane	-3.13	-2.68
0138pro	C_3H_8S	isooctane	-3.78	-3.24
0162tri	$CHCl_3$	isooctane	-3.06	-3.25
0240met	$C_8H_8O_2$	isooctane	-6.71	-7.21
0506nit	CH_3NO_2	isooctane	-2.82	-2.76
0045eth	C_2H_6O	isopropylbenzene	-2.90	-2.65
0075pro	C_3H_6O	isopropylbenzene	-3.32	-2.92
0076but	C_4H_8O	isopropylbenzene	-4.02	-3.56
0078pen	$C_5H_{10}O$	isopropylbenzene	-4.84	-4.21
0080hex	$C_6H_{12}O$	isopropylbenzene	-5.39	-4.90
0081dim	$C_6H_{12}O$	isopropylbenzene	-4.81	-4.50
0082hep	$C_7H_{14}O$	isopropylbenzene	-5.99	-5.60
0087pro	$C_3H_6O_2$	isopropylbenzene	-4.23	-4.73
0088but	$C_4H_8O_2$	isopropylbenzene	-4.93	-5.21
0094met	$C_4H_8O_2$	isopropylbenzene	-4.19	-4.06
0095eth	$C_4H_8O_2$	isopropylbenzene	-4.22	-3.93
0097pro	$C_5H_{10}O_2$	isopropylbenzene	-4.78	-4.52
0098met	$C_6H_{12}O_2$	isopropylbenzene	-5.45	-5.32
0099but	$C_6H_{12}O_2$	isopropylbenzene	-5.36	-5.18
0100met	$C_7H_{14}O_2$	isopropylbenzene	-6.19	-6.00
0101pen	$C_7H_{14}O_2$	isopropylbenzene	-6.13	-5.86
0110but	$C_4H_{11}N$	isopropylbenzene	-4.06	-3.81
0217wat	H_2O	isopropylbenzene	-1.41	-3.46
0502pro	C_9H_{12}	isopropylbenzene	-6.04	-5.61
0093met	$C_3H_6O_2$	isopropyltoluene	-3.32	-3.34
0094met	$C_4H_8O_2$	isopropyltoluene	-4.14	-3.83
0098met	$C_6H_{12}O_2$	isopropyltoluene	-5.33	-5.07
0100met	$C_7H_{14}O_2$	isopropyltoluene	-6.06	-5.74
0101pen	$C_7H_{14}O_2$	isopropyltoluene	-6.02	-5.60
0110but	$C_4H_{11}N$	isopropyltoluene	-4.22	-3.61
0053phe	C_6H_6O	mesitylene	-6.80	-5.91
0076but	C_4H_8O	mesitylene	-3.95	-3.50
0078pen	$C_5H_{10}O$	mesitylene	-4.80	-4.14
0080hex	$C_6H_{12}O$	mesitylene	-5.34	-4.82
0081dim	$C_6H_{12}O$	mesitylene	-4.77	-4.43
0082hep	$C_7H_{14}O$	mesitylene	-5.99	-5.51
0531mes	C_9H_{12}	mesitylene	-6.40	-5.44
0008noc	C_8H_{18}	methylenechloride	-5.18	-4.60
0036tol	C_7H_8	methylenechloride	-5.53	-4.94
0045eth	C_2H_6O	methylenechloride	-3.82	-3.73
0053phe	C_6H_6O	methylenechloride	-7.50	-7.43
0057pcr	C_7H_8O	methylenechloride	-7.71	-7.92
0062dio	$C_4H_8O_2$	methylenechloride	-5.33	-5.69
0139thi	C_6H_6S	methylenechloride	-7.11	-6.82
0161dic	CH_2Cl_2	methylenechloride	-3.80	-3.42
0215pbr	C_6H_5OBr	methylenechloride	-9.09	-9.59
0217wat	H_2O	methylenechloride	-2.63	-5.23
0506nit	CH_3NO_2	methylenechloride	-5.05	-4.74
0044met	CH_4O	nonane	-1.29	-1.70
0045eth	C_2H_6O	nonane	-2.15	-2.36
0047pro	C_3H_8O	nonane	-2.76	-2.88
0049but	$C_4H_{10}O$	nonane	-3.77	-3.43
0052pen	$C_5H_{12}O$	nonane	-3.92	-4.06
0053phe	C_6H_6O	nonane	-5.60	-5.66
0054hex	$C_6H_{14}O$	nonane	-4.97	-4.69
0055ocr	C_7H_8O	nonane	-6.20	-6.04
0058hep	$C_7H_{16}O$	nonane	-5.62	-5.36
0076but	C_4H_8O	nonane	-3.20	-3.36

0078pen	$C_5H_{10}O$	nonane	-3.97	-3.97
0080hex	$C_6H_{12}O$	nonane	-4.59	-4.62
0081dim	$C_6H_{12}O$	nonane	-4.19	-4.25
0082hep	$C_7H_{14}O$	nonane	-5.24	-5.29
0093met	$C_3H_6O_2$	nonane	-3.02	-3.34
0094met	$C_4H_8O_2$	nonane	-3.50	-3.82
0095eth	$C_4H_8O_2$	nonane	-3.45	-3.71
0097pro	$C_5H_{10}O_2$	nonane	-4.07	-4.28
0098met	$C_6H_{12}O_2$	nonane	-4.85	-5.03
0099but	$C_6H_{12}O_2$	nonane	-4.69	-4.91
0100met	$C_7H_{14}O_2$	nonane	-5.51	-5.67
0101pen	$C_7H_{14}O_2$	nonane	-5.33	-5.55
0103eth	C_2H_7N	nonane	-1.98	-2.22
0106pro	C_3H_9N	nonane	-2.96	-2.75
0110but	$C_4H_{11}N$	nonane	-3.55	-3.39
0511non	C_9H_{20}	nonane	-5.91	-5.59
0035ben	C_6H_6	nonanol	-3.82	-3.76
0036tol	C_7H_8	nonanol	-4.34	-4.32
0037eth	C_8H_{10}	nonanol	-4.61	-5.00
0053phe	C_6H_6O	nonanol	-8.61	-7.19
0103eth	C_2H_7N	nonanol	-4.02	-3.28
0106pro	C_3H_9N	nonanol	-4.66	-4.07
0110but	$C_4H_{11}N$	nonanol	-5.35	-4.87
0146met	$C_3H_8O_2$	nonanol	-5.61	-5.40
0215pbr	C_6H_5OBr	nonanol	-10.36	-8.96
0518non	$C_9H_{20}O$	nonanol	-9.05	-8.19
0008noc	C_8H_{18}	octane	-5.28	-5.29
0036tol	C_7H_8	octane	-4.82	-4.17
0044met	CH_4O	octane	-1.29	-1.70
0045eth	C_2H_6O	octane	-2.15	-2.31
0047pro	C_3H_8O	octane	-2.76	-2.94
0049but	$C_4H_{10}O$	octane	-3.69	-3.49
0052pen	$C_5H_{12}O$	octane	-4.10	-4.17
0053phe	C_6H_6O	octane	-5.47	-5.64
0054hex	$C_6H_{14}O$	octane	-4.86	-4.83
0055ocr	C_7H_8O	octane	-6.16	-6.14
0056mcr	C_7H_8O	octane	-5.19	-6.15
0057pcr	C_7H_8O	octane	-6.19	-6.17
0058hep	$C_7H_{16}O$	octane	-5.56	-5.50
0075pro	C_3H_6O	octane	-2.46	-2.71
0076but	C_4H_8O	octane	-3.24	-3.51
0078pen	$C_5H_{10}O$	octane	-3.97	-4.12
0080hex	$C_6H_{12}O$	octane	-4.60	-4.77
0081dim	$C_6H_{12}O$	octane	-4.21	-4.40
0082hep	$C_7H_{14}O$	octane	-5.25	-5.43
0093met	$C_3H_6O_2$	octane	-3.06	-3.38
0094met	$C_4H_8O_2$	octane	-3.57	-3.97
0095eth	$C_4H_8O_2$	octane	-3.48	-3.86
0097pro	$C_5H_{10}O_2$	octane	-4.09	-4.43
0098met	$C_6H_{12}O_2$	octane	-4.86	-5.17
0099but	$C_6H_{12}O_2$	octane	-4.80	-5.05
0100met	$C_7H_{14}O_2$	octane	-5.53	-5.81
0101pen	$C_7H_{14}O_2$	octane	-5.36	-5.69
0103eth	C_2H_7N	octane	-2.04	-2.30
0106pro	C_3H_9N	octane	-3.00	-2.89
0110but	$C_4H_{11}N$	octane	-3.44	-3.44
0111die	$C_4H_{11}N$	octane	-3.42	-3.38
0117met	$C_5H_6N_2$	octane	-4.70	-4.63
0118ani	C_6H_7N	octane	-4.84	-5.54
0131nit	$C_3H_7NO_2$	octane	-3.95	-4.08
0230eth	$C_6H_8N_2$	octane	-5.51	-5.31
n009	C_7H_9N	octane	-6.06	-5.92
n010	C_7H_9N	octane	-6.15	-5.95
n011	C_7H_9N	octane	-6.00	-5.96
0093met	$C_3H_6O_2$	pentadecane	-2.82	-2.96
0094met	$C_4H_8O_2$	pentadecane	-3.35	-3.38
0095eth	$C_4H_8O_2$	pentadecane	-3.37	-3.24
0097pro	$C_5H_{10}O_2$	pentadecane	-3.91	-3.75
0098met	$C_6H_{12}O_2$	pentadecane	-4.59	-4.45
0099but	$C_6H_{12}O_2$	pentadecane	-4.49	-4.31
0100met	$C_7H_{14}O_2$	pentadecane	-5.35	-5.06
0101pen	$C_7H_{14}O_2$	pentadecane	-5.18	-4.92
0516pen	$C_{15}H_{32}$	pentadecane	-9.91	-8.62
0005npe	C_5H_{12}	pentane	-3.35	-3.25
0044met	CH_4O	pentane	-1.29	-1.55
0045eth	C_2H_6O	pentane	-2.15	-2.26
0047pro	C_3H_8O	pentane	-2.76	-3.04
0049but	$C_4H_{10}O$	pentane	-3.77	-3.81
0052pen	$C_5H_{12}O$	pentane	-3.92	-4.53
0053phe	C_6H_6O	pentane	-5.67	-5.48
0054hex	$C_6H_{14}O$	pentane	-4.97	-5.22
0058hep	$C_7H_{16}O$	pentane	-5.62	-5.87
0078pen	$C_5H_{10}O$	pentane	-4.16	-4.53

0080hex	$C_6H_{12}O$	pentane	-4.79	-5.17
0081dim	$C_6H_{12}O$	pentane	-4.43	-4.63
0082hep	$C_7H_{14}O$	pentane	-5.40	-5.82
0093met	$C_3H_6O_2$	pentane	-3.13	-3.44
0094met	$C_4H_8O_2$	pentane	-3.69	-4.31
0095eth	$C_4H_8O_2$	pentane	-3.69	-4.23
0097pro	$C_5H_{10}O_2$	pentane	-4.21	-4.84
0098met	$C_6H_{12}O_2$	pentane	-4.96	-5.57
0099but	$C_6H_{12}O_2$	pentane	-4.88	-5.46
0100met	$C_7H_{14}O_2$	pentane	-5.67	-6.20
0101pen	$C_7H_{14}O_2$	pentane	-5.62	-6.09
0103eth	C_2H_7N	pentane	-2.18	-2.22
0106pro	C_3H_9N	pentane	-3.13	-2.98
0118ani	C_6H_7N	pentane	-5.15	-5.32
0162tri	$CHCl_3$	pentane	-3.26	-3.47
0179tri	$CHBr_3$	pentane	-4.83	-5.54
0075pro	C_3H_6O	perfluorobenzene	-3.82	-2.53
0078pen	$C_5H_{10}O$	perfluorobenzene	-5.10	-3.58
0080hex	$C_6H_{12}O$	perfluorobenzene	-5.55	-4.24
0081dim	$C_6H_{12}O$	perfluorobenzene	-5.26	-4.10
0082hep	$C_7H_{14}O$	perfluorobenzene	-6.15	-4.91
0093met	$C_3H_6O_2$	perfluorobenzene	-4.23	-3.03
0095eth	$C_4H_8O_2$	perfluorobenzene	-4.56	-3.31
0097pro	$C_5H_{10}O_2$	perfluorobenzene	-5.06	-3.89
0098met	$C_6H_{12}O_2$	perfluorobenzene	-5.59	-4.65
0099but	$C_6H_{12}O_2$	perfluorobenzene	-5.52	-4.53
0100met	$C_7H_{14}O_2$	perfluorobenzene	-6.21	-5.30
0101pen	$C_7H_{14}O_2$	perfluorobenzene	-6.16	-5.17
0110but	$C_4H_{11}N$	perfluorobenzene	-4.13	-3.28
0505per	C_6F_6	perfluorobenzene	-4.42	-3.81
0506nit	CH_3NO_2	perfluorobenzene	-4.30	-2.60
0008noc	C_8H_{18}	phenylether	-4.38	-4.55
0036tol	C_7H_8	phenylether	-4.86	-4.52
0045eth	C_2H_6O	phenylether	-3.22	-3.02
0062dio	$C_4H_8O_2$	phenylether	-4.83	-4.56
0076but	C_4H_8O	phenylether	-4.08	-3.59
0506nit	CH_3NO_2	phenylether	-4.19	-3.36
0093met	$C_3H_6O_2$	secbutylbenzene	-3.91	-3.40
0095eth	$C_4H_8O_2$	secbutylbenzene	-4.11	-3.76
0097pro	$C_5H_{10}O_2$	secbutylbenzene	-4.62	-4.35
0099but	$C_6H_{12}O_2$	secbutylbenzene	-5.22	-5.01
0101pen	$C_7H_{14}O_2$	secbutylbenzene	-5.98	-5.68
0076but	C_4H_8O	tbutylbenzene	-3.94	-3.40
0078pen	$C_5H_{10}O$	tbutylbenzene	-4.72	-4.04
0080hex	$C_6H_{12}O$	tbutylbenzene	-5.27	-4.73
0081dim	$C_6H_{12}O$	tbutylbenzene	-4.79	-4.42
0082hep	$C_7H_{14}O$	tbutylbenzene	-5.88	-5.43
0093met	$C_3H_6O_2$	tbutylbenzene	-3.57	-3.41
0094met	$C_4H_8O_2$	tbutylbenzene	-4.17	-3.89
0095eth	$C_4H_8O_2$	tbutylbenzene	-4.22	-3.76
0097pro	$C_5H_{10}O_2$	tbutylbenzene	-4.72	-4.35
0098met	$C_6H_{12}O_2$	tbutylbenzene	-5.39	-5.15
0099but	$C_6H_{12}O_2$	tbutylbenzene	-5.25	-5.01
0100met	$C_7H_{14}O_2$	tbutylbenzene	-6.13	-5.83
0101pen	$C_7H_{14}O_2$	tbutylbenzene	-5.92	-5.69
0530tbu	$C_{10}H_{14}$	tbutylbenzene	-6.43	-6.11
0053phe	C_6H_6O	tetrachloroethene	-6.10	-5.88
0075pro	C_3H_6O	tetrachloroethene	-3.09	-3.00
0093met	$C_3H_6O_2$	tetrachloroethene	-3.63	-3.70
0094met	$C_4H_8O_2$	tetrachloroethene	-4.39	-4.44
0095eth	$C_4H_8O_2$	tetrachloroethene	-4.22	-4.31
0097pro	$C_5H_{10}O_2$	tetrachloroethene	-4.80	-4.90
0098met	$C_6H_{12}O_2$	tetrachloroethene	-5.41	-5.70
0099but	$C_6H_{12}O_2$	tetrachloroethene	-5.35	-5.56
0110but	$C_4H_{11}N$	tetrachloroethene	-4.49	-3.91
0204tet	C_2Cl_4	tetrachloroethene	-5.39	-4.58
0008noc	C_8H_{18}	tetrahydrofuran	-5.39	-4.53
0036tol	C_7H_8	tetrahydrofuran	-5.50	-4.57
0045eth	C_2H_6O	tetrahydrofuran	-4.56	-3.37
0061tet	C_4H_8O	tetrahydrofuran	-4.25	-4.05
0062dio	$C_4H_8O_2$	tetrahydrofuran	-5.17	-5.20
0076but	C_4H_8O	tetrahydrofuran	-4.54	-4.66
0506nit	CH_3NO_2	tetrahydrofuran	-5.09	-4.36
0045eth	C_2H_6O	tetralin	-1.54	-2.73
0075pro	C_3H_6O	tetralin	-2.54	-2.95
0076but	C_4H_8O	tetralin	-3.12	-3.52
0078pen	$C_5H_{10}O$	tetralin	-3.99	-4.18
0080hex	$C_6H_{12}O$	tetralin	-4.64	-4.89
0081dim	$C_6H_{12}O$	tetralin	-4.19	-4.58
0082hep	$C_7H_{14}O$	tetralin	-5.33	-5.56
0217wat	H_2O	tetralin	0.07	-3.85
0534tet	$C_{10}H_{12}$	tetralin	-7.55	-7.03
0008noc	C_8H_{18}	toluene	-5.38	-5.16

0036tol	C_7H_8	toluene	-5.12	-4.30
0044met	CH_4O	toluene	-2.18	-2.04
0045eth	C_2H_6O	toluene	-3.33	-2.61
0047pro	C_3H_8O	toluene	-3.71	-3.32
0049but	$C_4H_{10}O$	toluene	-4.31	-3.91
0052pen	$C_5H_{12}O$	toluene	-5.17	-4.64
0053phe	C_6H_6O	toluene	-6.93	-6.02
0054hex	$C_6H_{14}O$	toluene	-6.12	-5.34
0055ocr	C_7H_8O	toluene	-7.43	-6.60
0057pcr	C_7H_8O	toluene	-7.56	-6.63
0058hep	$C_7H_{16}O$	toluene	-6.75	-6.05
0062dio	$C_4H_8O_2$	toluene	-4.91	-4.33
0075pro	C_3H_6O	toluene	-3.59	-3.01
0076but	C_4H_8O	toluene	-4.27	-3.88
0078pen	$C_5H_{10}O$	toluene	-5.02	-4.52
0080hex	$C_6H_{12}O$	toluene	-5.60	-5.21
0081dim	$C_6H_{12}O$	toluene	-5.00	-4.79
0082hep	$C_7H_{14}O$	toluene	-6.30	-5.91
0086eth	$C_2H_4O_2$	toluene	-4.00	-4.21
0087pro	$C_3H_6O_2$	toluene	-4.57	-4.88
0088but	$C_4H_8O_2$	toluene	-5.24	-5.36
0089pen	$C_5H_{10}O_2$	toluene	-5.89	-5.89
0090hex	$C_6H_{12}O_2$	toluene	-6.97	-6.47
0093met	$C_3H_6O_2$	toluene	-3.81	-3.76
0094met	$C_4H_8O_2$	toluene	-4.62	-4.37
0095eth	$C_4H_8O_2$	toluene	-4.41	-4.24
0097pro	$C_5H_{10}O_2$	toluene	-5.00	-4.83
0098met	$C_6H_{12}O_2$	toluene	-5.65	-5.64
0099but	$C_6H_{12}O_2$	toluene	-5.57	-5.49
0100met	$C_7H_{14}O_2$	toluene	-6.38	-6.31
0101pen	$C_7H_{14}O_2$	toluene	-6.41	-6.17
0103eth	C_2H_7N	toluene	-2.67	-2.42
0104dim	C_2H_7N	toluene	-2.68	-2.30
0106pro	C_3H_9N	toluene	-3.51	-3.13
0107tri	C_3H_9N	toluene	-2.71	-2.64
0110but	$C_4H_{11}N$	toluene	-4.33	-3.96
0111die	$C_4H_{11}N$	toluene	-3.75	-3.45
0115dip	$C_6H_{15}N$	toluene	-5.24	-4.96
0116pyr	C_5H_5N	toluene	-5.13	-4.54
0118ani	C_6H_7N	toluene	-6.69	-5.90
0131nit	$C_3H_7NO_2$	toluene	-5.25	-4.64
0215pbr	C_6H_5OBr	toluene	-8.70	-8.01
0216amm	H_3N	toluene	-2.38	-2.12
0217wat	H_2O	toluene	-1.69	-3.38
0228met	CH_5N	toluene	-2.65	-1.90
0240met	$C_8H_8O_2$	toluene	-7.96	-7.72
0506nit	CH_3NO_2	toluene	-4.31	-3.15
0648gbu	$C_4H_6O_2$	toluene	-4.70	-4.58
n011	C_7H_9N	toluene	-7.39	-6.51
n017	H_2O_2	toluene	-3.14	-3.78
0044met	CH_4O	tributyl phosphate	-4.16	-3.34
0045eth	C_2H_6O	tributyl phosphate	-4.57	-3.56
0047pro	C_3H_8O	tributyl phosphate	-5.42	-4.21
0049but	$C_4H_{10}O$	tributyl phosphate	-6.28	-4.88
0052pen	$C_5H_{12}O$	tributyl phosphate	-6.69	-5.57
0054hex	$C_6H_{14}O$	tributyl phosphate	-7.68	-6.20
0058hep	$C_7H_{16}O$	tributyl phosphate	-7.98	-6.84
0086eth	$C_2H_4O_2$	tributyl phosphate	-7.11	-5.57
0087pro	$C_3H_6O_2$	tributyl phosphate	-7.73	-6.05
0088but	$C_4H_8O_2$	tributyl phosphate	-8.29	-6.65
0089pen	$C_5H_{10}O_2$	tributyl phosphate	-8.82	-7.22
0103eth	C_2H_7N	tributyl phosphate	-3.29	-3.74
0106pro	C_3H_9N	tributyl phosphate	-3.98	-4.46
0118ani	C_6H_7N	tributyl phosphate	-7.60	-7.44
0146met	$C_3H_8O_2$	tributyl phosphate	-6.14	-5.47
0217wat	H_2O	tributyl phosphate	-4.69	-5.61
0008noc	C_8H_{18}	triethylamine	-5.62	-5.16
0036tol	C_7H_8	triethylamine	-4.98	-4.34
0045eth	C_2H_6O	triethylamine	-4.02	-2.63
0062dio	$C_4H_8O_2$	triethylamine	-4.41	-4.35
0076but	C_4H_8O	triethylamine	-3.86	-3.90
0506nit	CH_3NO_2	triethylamine	-3.63	-3.18
0510tri	$C_6H_{15}N$	triethylamine	-4.44	-4.38
0076but	C_4H_8O	trimethylbenzene	-3.97	-3.55
0078pen	$C_5H_{10}O$	trimethylbenzene	-4.83	-4.19
0080hex	$C_6H_{12}O$	trimethylbenzene	-5.39	-4.88
0081dim	$C_6H_{12}O$	trimethylbenzene	-4.80	-4.49
0082hep	$C_7H_{14}O$	trimethylbenzene	-6.01	-5.58
0093met	$C_3H_6O_2$	trimethylbenzene	-3.58	-3.56
0094met	$C_4H_8O_2$	trimethylbenzene	-4.14	-4.04
0098met	$C_6H_{12}O_2$	trimethylbenzene	-5.41	-5.31
0100met	$C_7H_{14}O_2$	trimethylbenzene	-6.16	-5.98
0101pen	$C_7H_{14}O_2$	trimethylbenzene	-6.09	-5.84

0532tri	C_9H_{12}	trimethylbenzene	-6.47	-5.46
0035ben	C_6H_6	undecane	-4.05	-3.55
0036tol	C_7H_8	undecane	-4.81	-4.19
0037eth	C_8H_{10}	undecane	-5.44	-4.90
0110but	$C_4H_{11}N$	undecane	-3.55	-3.27
0162tri	$CHCl_3$	undecane	-3.42	-3.09
0165tri	$C_2H_3Cl_3$	undecane	-3.82	-3.83
0172Edi	$C_2H_2Cl_2$	undecane	-3.60	-2.81
0173tri	C_2HCl_3	undecane	-3.87	-3.67
0174chl	C_6H_5Cl	undecane	-5.12	-4.50
0175odi	$C_6H_4Cl_2$	undecane	-6.11	-5.52
0179tri	$CHBr_3$	undecane	-4.84	-5.48
0204tet	C_2Cl_4	undecane	-4.63	-4.40
0520und	$C_{12}H_{26}$	undecane	-7.22	-7.15
0008noc	C_8H_{18}	xylene	-5.29	-4.99
0036tol	C_7H_8	xylene	-5.06	-4.20
0044met	CH_4O	xylene	-1.73	-2.01
0045eth	C_2H_6O	xylene	-3.42	-2.65
0047pro	C_3H_8O	xylene	-3.57	-3.25
0049but	$C_4H_{10}O$	xylene	-4.17	-3.84
0052pen	$C_5H_{12}O$	xylene	-4.72	-4.51
0053phe	C_6H_6O	xylene	-6.83	-6.02
0054hex	$C_6H_{14}O$	xylene	-5.85	-5.19
0055ocr	C_7H_8O	xylene	-7.25	-6.48
0056mcr	C_7H_8O	xylene	-6.32	-6.50
0057pcr	C_7H_8O	xylene	-7.18	-6.51
0058hep	$C_7H_{16}O$	xylene	-6.74	-5.90
0062dio	$C_4H_8O_2$	xylene	-4.86	-4.26
0075pro	C_3H_6O	xylene	-3.26	-2.97
0076but	C_4H_8O	xylene	-4.23	-3.72
0078pen	$C_5H_{10}O$	xylene	-4.87	-4.36
0080hex	$C_6H_{12}O$	xylene	-5.49	-5.05
0081dim	$C_6H_{12}O$	xylene	-4.91	-4.63
0082hep	$C_7H_{14}O$	xylene	-6.15	-5.76
0086eth	$C_2H_4O_2$	xylene	-4.08	-4.25
0087pro	$C_3H_6O_2$	xylene	-4.72	-4.81
0088but	$C_4H_8O_2$	xylene	-5.30	-5.29
0089pen	$C_5H_{10}O_2$	xylene	-5.71	-5.83
0090hex	$C_6H_{12}O_2$	xylene	-6.67	-6.40
0093met	$C_3H_6O_2$	xylene	-3.70	-3.71
0094met	$C_4H_8O_2$	xylene	-4.20	-4.22
0095eth	$C_4H_8O_2$	xylene	-4.26	-4.08
0097pro	$C_5H_{10}O_2$	xylene	-4.87	-4.68
0098met	$C_6H_{12}O_2$	xylene	-5.61	-5.48
0099but	$C_6H_{12}O_2$	xylene	-5.40	-5.34
0100met	$C_7H_{14}O_2$	xylene	-6.26	-6.16
0101pen	$C_7H_{14}O_2$	xylene	-6.19	-6.02
0103eth	C_2H_7N	xylene	-3.01	-2.38
0104dim	C_2H_7N	xylene	-3.36	-2.27
0106pro	C_3H_9N	xylene	-3.69	-3.17
0107tri	C_3H_9N	xylene	-2.63	-2.60
0111die	$C_4H_{11}N$	xylene	-3.93	-3.41
0113pen	$C_5H_{13}N$	xylene	-4.70	-4.60
0115dip	$C_6H_{15}N$	xylene	-5.35	-4.96
0116pyr	C_5H_5N	xylene	-5.12	-4.54
0118ani	C_6H_7N	xylene	-6.10	-5.99
0215pbr	C_6H_5OBr	xylene	-8.69	-8.00
0217wat	H_2O	xylene	-1.56	-3.43
0225pipa	$C_5H_{11}N$	xylene	-5.15	-4.13
0228met	CH_5N	xylene	-3.20	-1.87
0506nit	CH_3NO_2	xylene	-4.20	-3.13
n011	C_7H_9N	xylene	-7.17	-6.43

Part 4. Non-aqueous training II results.

The results provided in this section come from the ESE-EE-DNN version for nonaqueous solutions trained without using the MNSol and extrapolated MNSol data, without CombiSolv-QM data. These correspond to the *nonaqueous training II* in Table 2 in the main text.

Table S10. RMSE of the solvation free energy in kcal/mol for 14 *polar protic* solvents computed using two different trainings of the ESE-GB-DNN model (a total of 467 entries).

**Solvent**[a]		**training I**[b]	**training II**[c]
octanol(247)		1.10	1.91
heptanol(12)		0.95	0.87
m-cresol(7)		1.19	1.12
benzyl alcohol(10)		0.65	0.62
hexanol(14)		0.94	0.87
pentanol(22)		1.07	1.09
sec-butanol(9)		0.71	0.97
isobutanol(17)		1.25	2.19
methoxyethanol(6)		0.57	0.72
butanol(21)		1.12	1.35
isopropanol(7)		0.79	1.20
propanol(7)		0.76	1.15
ethanol(8)		1.08	1.27
methanol	cations(29)	1.13	0.89
	anions(51)	0.85	0.99
	all ions(80)	0.96	0.95
**All neutral solutes (387)**		1.06	1.70
**All polar protic solvents (467)**		1.05	1.60
Slope		1.002	0.993
Intercept		0.17	–0.14
R^2		0.998	0.995
# bad solvents[d]		6	8

[a] The number of entries in the data set is given in parentheses.
[b] Training including the CombiSolv-QM data.
[c] Training excluding the CombiSolv-QM data.
[d] The number of solvents for which RMSE > 1 kcal/mol for neutral solutes.

Table S11. RMSE of the solvation free energy in kcal/mol for 20 *polar aprotic* solvents using two different trainings of the ESE-GB-DNN model (a total of 338 entries).

**Solvent**[a]	**training I**[b]	**training II**[c]
bromoethane(7)	0.56	1.13
2-methylpyridine(6)	0.64	0.79
o-dichlorobenzene(11)	0.88	0.91
dichloroethane(39)	0.58	1.94
4-methyl-2-pentanone(13)	0.90	0.86
pyridine(7)	0.64	0.76
cyclohexanone(10)	1.18	1.50
acetophenone(9)	0.63	1.04
butanone(13)	0.65	0.72
benzonitrile(7)	0.58	0.61
o-nitrotoluene(6)	0.91	1.03
nitroethane(7)	0.40	0.59
nitrobenzene(15)	0.75	0.81
acetonitrile neutral solutes (7)	0.44	0.79
cations(39)	0.46	0.45
anions(30)	0.81	0.78
all ions(69)	0.63	0.54
nitromethane (7)	0.35	0.69
dimethyl formamide (7)	0.78	0.99
dimethyl acetamide (7)	0.80	0.98
sulfolane(7)	0.65	0.75
dimethyl sulfoxide neutral solutes (7)	0.95	1.11
cations (4)	0.53	0.63
anions (66)	0.47	0.57
methyl formamide (7)	1.03	1.37
**All neutral solutes(199)**	0.73	1.20
**All polar *aprotic(338)***	0.67	1.001
Slope	1.004	0.994
Intercept	0.07	–0.34
R^2	1.000	0.999
# bad solvents[d]	2	7

[a] The number of entries in the data set is given in parentheses.

[b] Training including the CombiSolv-QM data.

[c] Training excluding the CombiSolv-QM data.

[d] The number of solvents for which RMSE>1 kcal/mol for neutral solutes.

Table S12. RMSE of the solvation free energy in kcal/mol for 57 non-polar solvents computed using two different trainings of the ESE-GB-DNN model (a total of 1554 entries).

Solvent[a]	training I[b]	training II[c]
pentane(26)	0.40	0.57
hexane(59)	0.45	0.79
heptane(69)	0.48	0.79
isooctane(32)	0.34	0.57
octane(38)	0.30	0.48
nonane(26)	0.22	0.43
decane(39)	0.30	0.46
undecane(13)	0.48	0.80
dodecane(8)	0.32	0.76
cyclohexane(92)	0.63	0.82
perfluorobenzene(15)	1.17	1.41
pentadecane(9)	0.46	2.28
hexadecane(198)	0.68	1.39
decalin(27)	0.41	0.40
carbon tetrachloride(79)	0.53	1.10
isopropyltoluene(6)	0.37	0.50
mesitylene(7)	0.65	1.01
tetrachloroethene(10)	0.35	0.72
benzene(75)	0.81	1.17
sec-butylbenzene(5)	0.34	0.64
tert-butylbenzene(14)	0.40	0.63
butylbenzene(10)	0.48	0.71
trimethylbenzene(11)	0.45	0.67
isopropylbenzene(19)	0.58	0.54
toluene(51)	0.56	0.92
triethylamine(7)	0.63	1.00
xylene(48)	0.60	0.79
ethylbenzene(29)	0.54	0.69
carbon disulfide(15)	0.64	1.95
tetralin(9)	1.40	0.97
dibutyl ether(15)	0.54	0.70
diisopropyl ether(22)	0.93	1.13
hexadecyl iodide(9)	0.34	0.69
phenyl ether(6)	0.45	0.48
fluoroctane(6)	0.41	0.46
ethoxybenzene(7)	0.43	0.45
anisole(8)	0.44	0.44
diethyl ether(72)	0.92	1.77
bromoform(12)	0.42	1.81
iodobenzene(20)	0.41	0.33
chloroform(109)	1.05	1.83
dibromoethane(10)	0.36	0.43
butyl acetate(22)	0.97	1.24
bromooctane(5)	0.66	0.86
bromobenzene(27)	0.39	0.38
fluorobenzene(7)	0.40	0.46
chlorobenzene(38)	0.55	0.53
chlorohexane(11)	0.64	0.75
ethyl acetate(24)	1.00	1.11
acetic acid(7)	0.77	1.06
aniline(10)	1.03	1.13
dimethylpyridine(6)	0.67	0.79
tetrahydrofuran(7)	0.72	0.84
decanol(11)	0.94	0.94
tributyl phosphate(16)	1.17	1.75
nonanol(10)	0.78	0.75
methylene chloride(11)	0.87	0.50
**All non-polar** ***(1554)***	0.68	1.11
Slope	0.87	0.91
Intercept	–0.53	–0.06
R^2	0.892	0.76
# bad solvents[d]	5	16

[a] The number of entries in the data set is given in parentheses.
[b] Training including the CombiSolv-QM data.
[c] Training excluding the CombiSolv-QM data.
[d] The number of solvents for which RMSE > 1 kcal/mol for neutral solutes.

References

1. Marenich, A. V.; Kelly, C. P.; Thompson, J. D.; Hawkins, G. D.; Chambers, C. C.; Giesen, D. J.; Winget, P.; Cramer, C. J.; Truhlar D. G., Minnesota Solvation Database – version 2012, University of Minnesota, November 26, 2012. https://conservancy.umn.edu/bitstream/handle/11299/213300/MNSolDatabase_v2012.zip retrieved on 17/05/2019.
